# Supplementary material for: Two waves of evolution in the rodent pregnancy-specific glycoprotein (Psg) gene family lead to structurally diverse PSGs
Source: BMC Genomics. 2023 Aug 21;24:468. doi: 10.1186/s12864-023-09560-6 (PMC10440875; doi:10.1186/s12864-023-09560-6)
Supplement: Supplementary file 1 — Additional file 1. [file 12864_2023_9560_MOESM1_ESM.docx]

**Supplementary Table 1: Rodent CEACAM families genomic data sources**

| **abbre-viation** | **Latin name** | **common name** | **accession/**  **genomic data source^a^** | **genome coverage**  **sequencing technology** |
| --- | --- | --- | --- | --- |
|  |  |  |  |  |
| Aam | *Arvicola amphibius* | Eurasian water mole | CAJEUE010000000 | 55x PacBio  52x Genomics Chromium  10x BioNano  32x Dovetail Hi-C |
| Aca | *Acomys cahirinus* | Egyptian spiny mouse | PVKX01000000 | 32.7x Illumina HiSeq |
| Ani | *Arvicanthis niloticus* | African grass rat | JAAOME010000000 | 61.0x PacBio Sequel I CLR; Illumina NovaSeq; Arima Genomics Hi-C; Bionano Genomics DLS |
| Aru | *Aplodontia rufa* | Mountain beaver | PVKS010000000 | 30.6x Illumina HiSeq |
| Asp | *Apodemus speciosus* | Large Japanese field mouse | BDUI01000000 | 65x Illumina Hiseq2000 |
| Asy | *Apodemus sylvaticus* | European wood mouse | LIPJ01000000 | 17x Illumina HiSeq |
| Cap | *Cavia aperea* | Brazilian Guinea pig | AVPZ01000000  Ensembl CavAp1.0 | 333x Illumina HiSeq |
| Cca | *Castor canadensis* | American beaver | RPDE01000000  Ensembl C.can_genome_v1.0 | 87x Illumina HiSeq |
| Cga | *Cricetomys gambianus* | Gambian giant pouched rat | PVKD010000000 | 36.7x Illumina HiSeq |
| Cgr | *Cricetulus griseus* | Chinese hamster | FYBK01000000  Ensembl CHOK1GS_HDv1, CriGri_1.0, CriGri-PICR | n.a. |
| Cgu | *Cynomys gunnisoni* | Gunnison's prairie dog | WBRW01000000 | 67.0x PacBio; Illumina |
| Cla | *Chinchilla lanigera* | Long-tailed chinchilla | AGCD01000000  Ensembl ChiLan1.0 | 87x Illumina Hi-Seq |
| Cpa | *Cuniculus paca* | Lowland paca | RJWT010000000 | 23.4x Illumina Hi-Seq |
| Cpi | *Capromys pilorides* | Desmarest's hutia | PVKN010000000 | 26.1x Illumina Hi-Seq |
| Cpo | *Cavia porcellus* | Guinea pig | AAKN02000000  Ensembl Cavpor3.0 | 6.8x Sanger |
| Cso | *Ctenomys sociabilis* | Social tuco-tuco | PVKA01000000 | 26.5x Illumina HiSeq |
| Cts | *Cavia tschudii* | Montane guinea pig | PVKK010000000 | 34.8x Illumina HiSeq |
| Dbr | *Dinomys branickii* | Pacarana | PVLD010000000 | 31.9x Illumina HiSeq |
| Dor | *Dipodomys ordii* | Kangaroo rat | ABRO02000000  Ensembl Dord_2.0 | 181.0x Illumina  2.5x Sanger |
| Dpa | *Dolichotis patagonum* | Patagonian cavy | PVJX010000000 | 30.0x Illumina HiSeq |
| Dpu | *Dasyprocta punctata* | Punctate agouti | RJWM01000000 | 40.0x Illumina HiSeq |
| Dsp | *Dipodomys spectabilis* | Banner-tailed kangaroo rat | JAHHPX010000000 | 23.0x PacBio Sequel |
| Edo | *Erethizon dorsatum* | North American porcupine | SWEC01000000 | 25.0x Illumina HiSeq |
| Elu | *Ellobius lutescens* | Transcaucasian mole vole | LOEQ01000000 | 130.0x Illumina |
| Eta | *Ellobius talpinus* | Northern mole vole | LOJH01000000 | 50.0x Illumina |
| Fda | *Fukomys damarensis* | Damara mole-rat | JAAHWF010000000  Ensembl DMR_v1.0 | 29.0x Illumina X Ten; Hi-C |
| Gdo | *Grammomys dolichurus* | Common thicket rat | JADRCF010000000 | 26.3x Illumina HiSeq |
| Gmu | *Graphiurus murinus* | Woodland dormouse | PVLC010000000 | 35.0x Illumina HiSeq |
| Gsu | *Grammomys surdaster* | African thicket rat | SRMG01000000 | 50.0x Illumina NovaSeq |
| Hal | *Hylomyscus alleni* | Allen's wood mouse | JADRCC010000000 | 31.2x Illumina HiSeq |
| Hbr | *Hystrix brachyura* | Malayan porcupine | QZML01000000 | 137.0x Illumina HiSeq |
| Hcr | *Hystrix cristata* | Crested porcupine | PVJO010000000 | 35.2x Illumina HiSeq |
| Hgl | *Heterocephalus glaber* | Naked mole-rat | RPGA01000000  Ensembl HetGla_1.0  Ensembl etGla_female_1.0 | 77.0x BGISEQ-500 |
| Hhy | *Hydrochoerus hydrochaeris* | Capybara | PVLA01000000 | 28.2x Illumina HiSeq |
| Itr | *Ictidomys tridecemlineatus* | Thirteen-lined ground squirrel | AGTP01000000 | 495.1x Illumina HiSeq |
| Jja | *Jaculus jaculus* | Lesser Egyptian jerboa | AKZC01000000  Ensembl JacJac1.0 | 78.0x Illumina HiSeq |
| Lim | *Lophiomys imhausi* | Crested rat | CAJQZJ010000000 | n.a. |
| Mag | *Microtus agrestis* | Short-tailed field vole | CADCXT010000000 | n.a. |
| Mar | *Microtus arvalis* | Common vole | VIIT010000000 | 77.0x Illumina HiSeq |
| Mau | *Mesocricetus auratus* | Golden hamster | APMT01000000  Ensembl MesAur1.0 | 115x Illumina HiSeq |
| Mav | *Muscardinus avellanarius* | hazel dormouse | PVJB01000000 | 32.8x Illumina HiSeq |
| Mca | *Mus caroli* | Ryukyu mouse | FMAL02000000  Ensembl CAROLI_EIJ_v1.1 | n.a. |
| Mco | *Mastomys coucha* | Southern multimammate mouse | VSBT01000000 | 73.0x Illumina HiSeq |
| Mcy | *Myocastor coypus* | Nutria | PVJA010000000 | 56.5x Illumina HiSeq |
| Mfl | *Marmota flaviventris* | Yellow-bellied marmot | QZWP02000000 | 45.0x Illumina HiSeq |
| Mfo | *Microtus fortis* | Reed vole | NMRL01000000 | 108.0x Illumina HiSeq |
| Mgl | *Myodes glareolus* | Bank vole | MULK01000000 | 90.0x Illumina HiSeq |
| Mma | *Marmota marmota marmota* | Alpine marmot | CZRN02000000  Ensembl marMar2.1 | n.a. |
| Mmi | *Mus minutoides* | Southern African pygmy mouse | CACVCL010000000 | n.a. |
| Mmo | *Marmota monax* | Woodchuck | JAMOFY010000000 | 77.2x Illumina HiSeq; PacBio Sequel |
| Mmu | *Mus musculus* | House mouse | Ensembl GRCm39 | n.a. |
| Mmu_cas | *Mus musculus castaneus* | Southeastern Asian house mouse | LVXN01000000 | 60.0x Illumina |
| Mmu_dom | *Mus musculus domesticus* | Western European house mouse | LVXW01000000 | 60.0x Illumina |
| Mmu_mus | *Mus musculus musculus* | Eastern European house mouse | CAKLHR010000000 | n.a. |
| Mna | *Mastomys natalensis* | African soft-furred rat | JAJTUV010000000 | 53.0x PacBio RSII |
| Moc | *Microtus ochrogaster* | Prairie vole | JAATJU010000000  Ensembl MicOch1.0 | 108.0x Illumina |
| Moe | *Microtus oeconomus* | Root vole | VIIU01000000 | 77.0x Illumina HiSeq |
| Mor | *Microtus oregoni* | Creeping vole | JAGKIF010000000 | 43.0x PacBio Sequel;  Oxford Nanopore PromethION |
| Mpa | *Mus pahari* | Shrew mouse | FMBV02000000  Ensembl PAHARI_EIJ_v1.1 | n.a. |
| Mri | *Microtus richardsoni* | Water vole | JAGDQN010000000 | 120.0x Illumina |
| Msi | *Mus spicilegus* | Steppe mouse | QGOO01000000 | 50.0x 10x Genomics; Illumina HiSeq |
| Msp | *Mus spretus* | Algerian mouse | LVXV01000000  Ensembl SPRET_EiJ_v1 | 60.0x Illumina |
| Mun | *Meriones unguiculatus* | Mongolian gerbil | VFHZ01000000  Ensembl MunDraft-v1.0 | 1.0x Illumina HiSeq |
| Mva | *Marmota vancouverensis* | *Vancouver marmot* | SAYX01000000 | 25.0x Illumina HiSeq |
| Nga | *Nannospalax galili* | Upper Galilee mountains blind mole rat | AXCS01000000  Ensembl S.galili_v1.0 | 86.0x Illumina HiSeq 2000 |
| Nle | *Neotoma lepida* | Desert woodrat | LZPO01000000 | 48.0x Illumina HiSeq |
| Obe | *Otospermophilus beecheyi* | California ground squirrel | n.a. | n.a. Illumina NovaSeq |
| Ode | *Octodon degus* | Degu | AJSA01000000  Ensembl OctDeg1.0 | 80.0x Illumina HiSeq |
| Omi | *Octomys mimax* | Viscacha rat | NDGM010000000 | 10.0x Illumina HiSeqX |
| Oto | *Onychomys torridus;* | Southern grasshopper mouse | PVIT010000000 | 46.8x Illumina HiSeq |
| Ozi | *Ondatra zibethicus* | Muskrat | PVIU01000000 | 43.9x Illumina HiSeq |
| Pat | *Peromyscus attwateri* | Texas deermouse | CABHPP010000000 | n.a. |
| Paz | *Peromyscus aztecus* | Aztec mouse | CABHPQ010000000 | n.a. |
| Pca | *Peromyscus californicus insignis* | California mouse | VALE02000000 | 37.0x Illumina NovaSeq |
| Pcp | *Pedetes capensis* | Springhare | VMDO01000000 | 39.7x Illumina HiSeq |
| Pde | *Praomys delectorum* | Delectable soft-furred mouse | JADRCD010000000 | 26.3x Illumina HiSeq |
| Per | *Peromyscus eremicus* | Cactus mouse | CACRXM010000000 | n.a. |
| Ple | *Peromyscus leucopus* | White-footed mouse | NMRJ02000000 | 60.0x Illumina; PacBio; HiC-Illumina |
| Pma | *Peromyscus maniculatus bairdii* | Northern American deer mouse | AYHN01000000  Ensembl Pman_1.0 | 110.0x FLX 454; Illumina HiSeq |
| Pme | *Peromyscus melanophrys* | Plateau mouse | CABHPR010000000 | n.a. |
| Pnu | *Peromyscus nudipes* | Naked-footed Deermouse | CABHPH010000000 | n.a. |
| Pob | *Psammomys obesus* | Fat sand rat | NESX02000000 | 87.6x Illumina HiSeq |
| Ppo | *Peromyscus polionotus subgriseus* | Oldfield mouse subspecies | RCWS02000000 | 290.0x Illumina |
| Psu | *Phodopus sungorus* | Dzhungarian hamster | MCBN010000000 | 37.0x Illumina HiSeq |
| Pty | *Petromus typicus* | Dassie-rat | PVIR01000000 | 37.7x Illumina HiSeq |
| Rdi | *Rhabdomys dilectus* | Mesic four-striped grass rat | JADRCG010000000 | 55.8x Illumina HiSeq |
| Rno | *Rattus norvegicus* | Norway rat | AAHX01000000  Ensembl mRatBN7.2 | n.a. |
| Rop | *Rhombomys opimus* | Great gerbil | REGO01000000 | 86.0x Illumina HiSeq |
| Rpr | *Rhizomys pruinosus* | Hoary bamboo rat | VZQC01000000 | 205.0x Illumina HiSeq; PacBio RSII |
| Rra | *Rattus rattus* | Black rat | JAAIVD010000000 | 130.0x Illumina HiSeq; Oxford Nanopore |
| Rso | *Rhynchomys soricoides* | Mount Data shrew rat | JADRCH010000000 | 56.4x Illumina HiSeq |
| Sda | *Spermophilus dauricus* | Daurian ground squirrel | AXRT01000000 | 132.3x Illumina HiSeq |
| Shi | *Sigmodon hispidus* | Hispid cotton rat | PVIH01000000 | 43.2x Illumina HiSeq |
| Svu | *Sciurus vulgaris* | Eurasian red squirrel | CACRXH010000000 | 23x PacBio  44x 10x Genomics Chromium |
| Tba | *Tympanoctomys barrerae* | Plains viscacha rat | NDGN010000000 | 10.0x Illumina HiSeqX |
| Tsw | *Thryonomys swinderianus* | Greater cane rat | PVIC010000000 | 58.2x Illumina HiSeq |
| Upa | *Urocitellus parryii* | Arctic ground squirrel | QVIC01000000  Ensembl ASM342692v1 | 40.0 Illumina HiSeq |
| Xin | *Xerus inauris* | South African ground squirrel | PVHX01000000 | 24.4x Illumina HiSeq |

^a^ Ensembl databases; UCSC, UCSC Genome Browser assembly; GenBank

n.a., not available
