# Supplementary material for: Two waves of evolution in the rodent pregnancy-specific glycoprotein (Psg) gene family lead to structurally diverse PSGs
Source: BMC Genomics. 2023 Aug 21;24:468. doi: 10.1186/s12864-023-09560-6 (PMC10440875; doi:10.1186/s12864-023-09560-6)
Supplement: Supplementary file 2 — Additional file 2. [file 12864_2023_9560_MOESM2_ESM.docx]

**Supplementary File 1**

**ITAM-encoding cytoplasmic domain exon nucleotide sequences of rodent Ceacam genes**

ITAM, immunoreceptor tyrosine-based activation motif; TSA, transcriptome shotgun assembly; WGS, whole genome shotgun

>Cla_CEACAM3L_Cyt_ITAM (Chinchilla lanigera; long-tailed chinchilla) WGS AGCD01073029.1

GCCTAGCATCCAGCATGACCTTGGCGAGCACCAGCAACCTGTGTCCATAGCCGGTCTCAAGTTTTGCAGCAGCTTCACCTCCCAGGACACCCTCTTGAGTTCCAAGACAGCAGTTCCCATCTATGAGGATCTACTGAACCCTGACATGAATATTTACTGCAAGATCAACCATAAAGCAGATACTGCTTCATAA

PSIQHDLGEHQQPVSIAGLKFCSSFTSQDTLLSSKTAVPIYEDLLNPDMNIYCKINHKADTAS-

>Cpo_Ceacam3L_Cyt_ITAM (Cavia porcellus; Guinea pig) Ensembl ENSCPOG00000030322.1

GCCCAGCATGCAGCATGACCTCGGAGAGCACTGGCAACCTGTGTCAACCTCCAGCCCCGATTTTTGCAACAGTTTCACAAGCCAGGTCAACCTCCCTGATTCCAAGGAAACCGTTCCTATCTATGAGGAATTACTAATCTCTGACATGAATGTTTACTGCGAGATCAACCACAGAGGAGATACAGCTTCATAA

PSMQHDLGEHWQPVSTSSPDFCNSFTSQVNLPDSKETVPIYEELLISDMNVYCEINHRGDTAS-

>Mmo_Ceacam3L1_Cyt_ITAM (Marmota monax; woodchuck) WGS JAMOFY010000149.1

GGCCAGCGTCCAGCTTGGCCTCAGAGACCACCGCACCCCAGCATCAAGACCCGGCCAAGGTCCCTCTGACACTTCCACCTCCCTGGCCCCACCCCCTGGCCACAGAGCTGCTGTCCCCATCTACCAGGAATTACTAAACCCTGACCTGGACATCTACTGCCGGGTCGACCACAAGGCTGATGTGGATTCTTAG

ASVQLGLRDHRTPASRPGQGPSDTSTSLAPPPGHRAAVPIYQELLNPDLDIYCRVDHKADVDS-

>Mmo_Ceacam3L2_Cyt_ITAM (Marmota monax; woodchuck) WGS JAMOFY010000149.1

GGCCAGCGTCCAGCTTGGCCTCAGAGAACACCACACCCCAGCATCAAGACCCGGCCAAGGTCCCTCTGACAGCTCCACCTCCCTGGCCCCACCCCCTGGCCACAGAGCTGCTGTTCCCATCTACCAGGAATTGCTAAACCCTGACCTGGACATCTATTGCCGGGTCGACCACAAGGCTGATGTGGATTCTTAG

ASVQLGLREHHTPASRPGQGPSDSSTSLAPPPGHRAAVPIYQELLNPDLDIYCRVDHKADVDS

>Mmo_Ceacam3L3_Cyt_ITAM (Marmota monax; woodchuck) WGS JAMOFY010000149.1

GGCCAGTGTCCAGCTTGGCCTCAGAGACCACTGCACCCCAGCATCAAGACCCGGCCAAGGTCCCTCTGACACTTCCACCTCCCTGGCCCCACCCCCTGGCCACAGATCTGCTGTTCCCATCTACCAGGAATTGCTAAACCCTGACCTGGACATCTACTGCCGGGTCGACCACAAGGCTGATGTGGATTCTTAG

ASVQLGLRDHCTPASRPGQGPSDTSTSLAPPPGHRSAVPIYQELLNPDLDIYCRVDHKADVDS-

>Mmo_Ceacam3L4_Cyt_ITAM (Marmota monax; woodchuck) WGS JAMOFY010000149.1

GGCCAGTGTCCAGCTTGGCCTCAGAGACCACCGCACCCCAGCATCAAGACCCGGCCAAGGTCCCTCTGACACTTCCACCTCCCTGGCCCCACCCCCTGGCCACAGATCCGCTGTTCCCATCTACCAGGAATTGCTAAACCCTGACCTGGACATCTACTGCCGGGTCGACCACAAGGCTGATATGGATTCTTAG

ASVQLGLRDHRTPASRPGQGPSDTSTSLAPPPGHRSAVPIYQELLNPDLDIYCRVDHKADMDS-

>Mmo_Ceacam3L5_Cyt_ITAM (Marmota monax; woodchuck) 23.10.22 WGS CABDUW010004955.1 GGCCAGCGTCCAGCTTGGCCTCAGAGAACACCACACCCCAGCATCAAGACCCGGCCAAGGTCCCTCTGACAGCTCCACCTCCCTGGCCCCACCCCCTGGCCACAGAGCTGCTGTTCCCATCTACCAGGAATTGCTAAACCCTGACCTGGACATCTATTGCCGGGTCGACCACAAGGCTGATGTGGATTCTTAG

ASVQLGLREHHTPASRPGQGPSDSSTSLAPPPGHRAAVPIYQELLNPDLDIYCRVDHKADVDS-

>Nga_Ceacam3LCyt1_ITAM (Nannospalax galili; Upper Galilee mountains blind mole rat) XM_029570052.1

GGTCAGGGTCCAGCATGACCTCAGAGAGCCAAAGCCGCCAGTGTCCACTTCAGGTCCTGGAGCCTCAGACAGCTATGCCCTGGTCCCCTTTTCCACCCCCAAGGAAGCCACTCCTGTATACCAGGAGTTACTAAAGCCTAGTGTGAGCATTTATGACCAGATCAACCACACAGAAGATGTGGCTTCTTAG

VRVQHDLREPKPPVSTSGPGASDSYALVPFSTPKEATPVYQELLKPSVSIYDQINHTEDVAS-

>Obe_Ceacam3L1Cyt1_ITAM (Otospermophilus beecheyi Obeecheyi; California ground squirrel) TSA GJUE01021571.1

AGGGCCACTGTCCAGCATGGCCTCAGAGACCACCGCACCCCAGCATCAAGACCCGGCCAAGGTCCCTCGGAGAGCTCCACCTTCCTGGCCCCACCCCCTGGCCACGGAGCTGCTGTTCCCATCTACCAGGAATTACTAAACCCTGACCTGGACATCTACTGCCGGGTGGACCACACAGCAGTTGGAGGTTCCTAG

RATVQHGLRDHRTPASRPGQGPSESSTFLAPPPGHGAAVPIYQELLNPDLDIYCRVDHTAVGGS-

>Obe_Ceacam3L2Cyt1_ITAM (Otospermophilus beecheyi Obeecheyi; California ground squirrel) TSA GJUE01021552.1

AGGGCCAGTGTCCAGCATGGCCTCAGAGACCACCGCACCCCTGCAACAAGACCCGGCCAAGGTCCCTCTGACAGCTCCACCTCCCTGGCCCCACTCCCTGGCCACGGAGCTGCTGTTCCCATCTACCAGGAATTGCTAAACCCTGACCTGGACATCTACTGCAGGGTCAACCACAAGGCTGATGTGGATTCTTAG

RASVQHGLRDHRTPATRPGQGPSDSSTSLAPLPGHGAAVPIYQELLNPDLDIYCRVNHKADVDS-

>Obe_Ceacam3L3Cyt1_ITAM (Otospermophilus beecheyi Obeecheyi; California ground squirrel) TSA GJUE01021556.1

AGGGCTGGTGTCCAGCATGGCCTCAGAGACCACCGCACCCCAGCATCAACAGCCTGTCAAGGTCCCTCTGACAGCTCCACCTCCCTGGCTCCTGTTTCTGGCCACAAAACTGCTGTTCCCATCTACCAGGAATTACTAAACCCTGACCTGGACATCTACTGCCGGGTGGACCACACAGCAGTTGGAGGTTCCTAG

RAGVQHGLRDHRTPASTACQGPSDSSTSLAPVSGHKTAVPIYQELLNPDLDIYCRVDHTAVGGS-

>Upa_CEACAM3L1Cyt1_ITAM (Urocitellus parryii; Arctic ground squirrel) WGS QVIC01002149.1

GGCTGGTGTCCAGCATGGCCTCAGAGACCCCGGCACCCCAGCAACATCACATGGCCAAGGTCCCTCTKACAGCTCCACCTCCCTGTCCCCTGTCCCTGGCCACAGGTCTGCGGTTCCCATCTACCAGGAATTACGAAACCCTAATGTGGACATCTATTGTCGTATTGACCACAAAGCTCACGTGGCTTCATAG

AGVQHGLRDPGTPATSHGQGPSXSSTSLSPVPGHRSAVPIYQELRNPNVDIYCRIDHKAHVAS-
