# Supplementary material for: Two waves of evolution in the rodent pregnancy-specific glycoprotein (Psg) gene family lead to structurally diverse PSGs
Source: BMC Genomics. 2023 Aug 21;24:468. doi: 10.1186/s12864-023-09560-6 (PMC10440875; doi:10.1186/s12864-023-09560-6)
Supplement: Supplementary file 3 — Additional file 3. [file 12864_2023_9560_MOESM3_ESM.docx]

**Supplementary File 2**

**N/N1 exon nucleotide sequences of rodent pregnancy-specific glycoprotein genes**

_Ceacam11l, Ceacam11-like; _P, pseudogene (no open reading frame in N/N1 exon); _WGS, whole genome shotgun; alleles (definition >99% nucleotide sequence identity) are not listed

>Aam_Psg2N1 (Arvicola amphibius; Eurasian water mole) WGS CAJEUE010010886.1

CCTACCTTTTAACCTGTTGGCACCTGCCCACCACTCTACAAGTTATTGTTGATTTAGTGCCACCCCATGTTGTTGAAGGAGAAGATGTCCTTCTTCGTGTCCGCAACCTGCCAGAGGATATTGTAGCCTTTGTCTGGCACAAAGGGGCGACAAAGATGAACCTCGGAATTGTACTTTATTCACTGACCACTAATTTGAACATCACAGGGCCTGGACACAGTGGTAGAGAGATAGTGTACAGAAATGGATCTCTGCGCCTCCAAAATGTCACCCAAAAGGACACAGGATTCTACACGCTACGATCCTTAAATAGGCATAAAGGAATTGTATCAACAACATCTATACACCTGCATGTATACT

>Aam_Psg3N1 (Arvicola amphibius; Eurasian water mole) WGS CAJEUE010010886.1

CCTCCCTTTTCTCCTTCTGGCATCTCCCCACTACTGCTCATGTGTCCACTGAATCAGTGCCACCCCTAGTGGCTGAAGGAGATAACGTCCTTATCCTTGTCAACAATCTTCCAGAGAATCTTTTAGCCTTAGCCTGGTTCAAAGGGCTAACAAATATGAAGCAAGGAATTGCATTATATGCACTGCACAAAAATATAAGTGTTACAGGGCCTGTGCACAGTGGCAGAGAGACAATATATCACAATGGATCTTTGTTGATTGAAAAACTCACCCAGAAGGACACAGGATTCTACACCTTTCGAGCCTATAATAGACGTGGAAGAATTGTATCAACCACAGCTACCTACCTCCATGTGCAAG

>Aam_Psg5N1 (Arvicola amphibius; Eurasian water mole) WGS CAJEUE010014165.1

TCTCCCTTTTAAGCTCCTGGCATCTGTCCACAAATGCCCATACGACTATTGAAAAAGTGCCAACCCTAGTTGCTGAAGGAGATGACATCCTTTTCCATGTTAGTGATCTGCCAGAGAATATTACAACCTTAGCCTGGTTCAAAGGTCTAAGAAATACGACACAAGGAATTGCAGGATATGCACCGCTCTTCAATGTGAGTAGGCCAGGTCCTATGTACAGTGGTAGAGAGACAATATATCGCAATGGATCCCTGCTGATAAAAAATGTCAACCCGTCGGACACTGGATTCTATACCCTACGAACTTATAATAGACATGGAACCAGGATATCAGTAACATCCACGTACCTCCAAGTGCATG

>Aam_Psg6N1_P (Arvicola amphibius; Eurasian water mole) WGS CAJEUE010010885.1 TCTCCCTTTTAACCTCCTGGCACCTGTCCACTGCTGTCCATATAACTACTGAGTCAGTTTCATCCCGAGTGGTTGAAGGAGAAAACGTCCTTTTCCTTGTGCATGATCTGCCAGATAATACTAAATCCTTAGTCTGGTTCAAAGCTCTAAGAAATGTGACAGAAGAAATTGCAGCATATGCACTGCCCTACAATTTAAGTAGGCCAGGTCCTCTGTACAGTGGTAGAGAGACAATATATCGCAATGGATCCCTGATGATAGAAAATATCAACCTCAAGGACACAGGATTCTATATCCTACAAACCTATAACAGACGTAAAAAAAGTCATATCAACAACAACCATGTATCTCCAAGTGAATG

>Aam_Psg9N1 (Arvicola amphibius; Eurasian water mole) WGS CAJEUE010014164.1

CCTTCCTTTCAATCTGTTGTCATTCACCTGCCACTGCTGAAGTCACCATTGAATCAGTGCCGCCCAATGTGTTCGAAGGAGACAGTGTCCTTCTATATGTCCACAATCTGCCAGAGAATCTGCTAGCCTTTGCTTGGTTCAAAGGGCTAACAAATATGAAACGCAGAATTGTACTCTATGAACTGAACAACAATTTAAGTTGCCGGGGGCCTGAATACAGTGGTAGAGAGACAGTGTACCGCAATGGATCCCTGTGGATTTCCAATGTCACCCACGTGGACACAGGATTCTATACCCTACGAACCATAAGTAGACATTCAAGAATTGTGTCAATAACAACCATCCACCTCCCTGTGTACA

>Aam_Psg10N1 (Arvicola amphibius; Eurasian water mole) WGS CAJEUE010010885.1 CCTCCATTTTAAGCTTCTGGCACATGTCCACTACTGCCCATGAGACCACCGAGTCACTGCCACACCAAGTGGTTGAAGGAGAAAACGTCCTTTTGCTGGTCCACAATCTGCCAGAGAATCTTATAGCCTTTGCCTGGTTCAAAGGGCTAACAAATATGACGCAAGGAATTGCAGTATATACACTGCACAACAATTTAAGTACACCAGGACCTGTGCACAGTGGTAGAGAGACAGTGTATAGCAATGGATCTCTGCTGATAGAAAATGTCACCCAGAAAGACACAGGAATCTATACCCTACGAACCTATAATAGAAGTGGAAAAATTGCATCAACAACATCTATGTACCTCCACGTCCACG

>Aam_Psg11N1 (Arvicola amphibius; Eurasian water mole) WGS CAJEUE010014164.1

CCTCCCTTTTGACTTACTGGTATCTACCCACCACTGCCCAAGTTATCATTGAACTAGTGCCACCCAAGGTGTTCCAAGGAGAAAATGTCCTTCTAGAGGTCCACAATCTGCCAGAGGATTTTCTAGCCTTTGCTTGGTACAGAGGGGTGACAACCATGAAGCGCGGAATTGCAGTCTATGCCAAGAGAAACAGTTTAAGAGCATCGGGGCCCGCGTACAGTGGCAGACAGACAGTGTACAGTGACGGGTCACTGTTGCTCCAGCGTGTCATCCTCAAGGACACAGGATTCTACACCCTGCGAGTCATAAGTAGACAAGGAGAAATTGTATCAACAACATCCGTGTTCCTCCATGTGCAGA

>Aam_Psg16N1 (Arvicola amphibius; Eurasian water mole) WGS CAJEUE010010886.1

CCTCCCTTTTAACCTGCCACCTGTCCACCACTGTCAAAATCACAATTGACTCAGTGCCACTCCAAGTGGTTGAAGGAGAAAACGTCCTTCTACGTGTCAACAATCTGCCACAGAATCTTCGAAAATTTGCCTGGTTCAAAGGGGTGACAAATATGAACTTCAGAATTGCACTATATACACTGACCACTAATCTATATGAGATGGGGCCTGAAAATAGTGGTAGAGAAGCTGTGTACAGCAATGGATCCCTATGGCTCAAAAATGTCTCCCAGAAGGACACAGGATTTTATATACTACAAACAATAAATAGAAGTGGAAAAATTGTATCTATAACCACATACCTCCACGTGTATG

>Aam_Psg19N1_P (Arvicola amphibius; Eurasian water mole) WGS CAJEUE010014164.1

CTTGCCTTTTAACCTCCTGGCACCTGTCTTCCACTGCCAATGTGACCATTGAATTACTGCCAACTCCAGTGGCCGAAGGAGATAACATCCTTTTCCTGGTCCACAATCTGCCAGAGGAAATTAAAGCTGTAGCCTGGTTCAAAGGCTGGGAAATAAGAAACAACAAATTGCAGTGTATGTACTGTACAAAAATTTAAGTAAGCCAGGTCCTATGCACAGCGGGAGAGAGATAATATATCACAATGGATCCCTGCTATTGAAAAGGTTACCCAGAAGGATGCAGGATTCTATACCCTACAACCTATGATAGAGGTGGAAAATTTGTATCAACCATACCCATGTACCTCCACGTGCAAG

>Aam_Psg20N1 (Arvicola amphibius; Eurasian water mole) WGS CAJEUE010014164.1

CCTCTATTTTAACCTCCTGGTACCTGTCTTCCACTGCCAATGTGACAATTGAATTACTGCCAACTCCAGTGGCCGAAGGAGATAACGTCCTCTTCCTTGTCCACAATCTGCCAGAGGATATTATAGACGTTGCCTGGTTCAAAGGGCTGGGAAAAAAGAAACAACAAATTGCAGTGTATGTACTGCACAAAAATTTAAGTAAGCCAGGTCCTATGCACAGCGGGAGAGAGATAATATATCACAATGGATCCCTGCTACTTGAAAAGGTCACCCAGAAGGATGCAGGATTCTATACCCTACGAACATATAATAGAGAAAGAAGATTTGTATCAACCATACCCATGTACCTCCACGTGCACG

>Aca_Ceacam9N (Acomys cahirinus; Egyptian spiny mouse) WGS PVKX01035343.1

CCTCCCTCTTAGCCTGTGGGAATGCACCCACCGCCGCCGAGCTCACTATCGAATTGGTGCCGCCCGTGGTGGCCGAAGGTGGAAACTCCGTGCTCTTTGTGCATAAGATGCCCCTGAACACCCAGGCATTTTACTGGTACAAACAGAAGGATCCCACGAAGAGCTACGAAGTCGCGCGCTACTTAACACCCACGAACGCATCCTCGAAGATGCCGCAGCACAGCGGCAGGAAAACGGTTTTCTACAGTGGGTCCCTGCTCATCAAAAACGTCACCCAGGCTGACAGCGGCCTCTACACTTTACTAACGTTTAACACAGAGATGGAAAGCGAATTAACACACGTGCATCTGGATGTACAAA

>Aca_Ceacam11lN1 (Acomys cahirinus; Egyptian spiny mouse) WGS PVKX01026098.1

CTTCACTTTTAACCTACTGGTTGCCTCCTACCACTGCCCAGATCACCATTGAAACAGAACCTCCCATTGCTTTGGAAGGGCAAAATGTTCTTCTTTCTGTGTACGGTCTACCAGAGAGTGTGCAAGCCCTTTCCTGGTTCACAGGAGTTTTGGTGTTCAAGGGCTGTGAAATTGCAAGATATGTGATTGCTAACAATTCTTATGTGCTGGGGATTGCACATAGTGGTAGAGAGACAGTACTCAACAATGGATCTCTGCTGATCAAGAATGTCACTAGGAAGGACTCAGGATACTACACCCTCCAAACATTTAAAGAACATTCAAGTTCGGAAATAACACGTGCTGAGTTCTTTGTACACA

>Aca_Ceacam15N (Acomys cahirinus; Egyptian spiny mouse) WGS PVKX01028227.1

CTTCACTGTTAACCTGCTGGAGCTCACCGGCCCCGGCGACACTAACATCTACAGATTTGCGGTTCTCTGCGGCGGAAGGGGCCAGGGTTCTTCTCCACGTCCCTAACCAGGAAAAGGACCTCCTCTCCTTTTCCTGGTACCAAGGGAAAGATGTACATGAGAATTTTACAATTGCGCATTACGAAAAGGCCAAAGATGTCTTTAAACTTGGACGTAAAGTCAGCGGCAGAGAAGAAATTTATAAGGATGGATCCATGATTCTCGGGCCCGTCACAAAGGAAGACACGGGGATCTACACCCTAGAAACCTTTAAAACACAAAATCAATATGAAACAACGTATGTCCATCTTCAGGTGTACA

>Aca_Psg1N1 (Acomys cahirinus; Egyptian spiny mouse) WGS PVKX01009468.1

CCTGCCTTTTAACCTGCTGGCACCTGTCCACTGCTGATGAAGTCAGCATTGAATTAGTGCCACCCCAAGTGGTTGAAGGAGAAAATGTCCTTTTCCTTGTTCACAATCTGCCAGATAATCTTAGAGCCTTAAGTTGGTTCAAAGGGAGAACAAATACGAGCCACGAAATTGCACTGTATACAATACATGCTAAAGAATGTGTGACAGGACCTGAACACAGCGGTAGAGAGACAGTGCACAGCAGTGGATCTCTGCGGATTCTCAAGGTCACCCAGAAGGACATAGGATACTACACCCTTCGAACATTTAATAAACAATCAGAAACGGTATCAATAACATCCACGTACCTCCATGTGAACA

>Aca_Psg2N1 (Acomys cahirinus; Egyptian spiny mouse) WGS PVKX01008214.1

CTTCTATTTTAACTGGCTGGCACCCTCCCACCACTGCTGATGTCACTGTTGAGACAGTGCCACCTGAAGTGGTTGAAGGACAAAACGTCCTTCTACTAATAAACAGTCTGCCGAAGAACATTATAGCCTTGGCCTGGTATAAAGGGGTACAGAATATGAGCCATGTCATTGCACTGTACTCACTGAAATATGATATAAGTGTGACAGGGCTTGCACACAGTGGTAGAGAGACAATGTACCGCAATGGGTCCTTGCTGATCCAAAATTTTACCCGGAAAGACGACGGATTTTATACCCTACGAACCATAAGTAGTCGTGGAGAATTTGTATCAACAACAACCACGCACGTCCAGGTGTACA

>Aca_Psg3N1 (Acomys cahirinus; Egyptian spiny mouse) WGS PVKX01006019.1

CCTCCCTGGTAACATGCTGGCACCTGTCTACCACTACCTACGTCAACATTGAATTACTGCCACGCAAAGTGTTTGAGGGAGAAAACGTCCTTCTACTTCCTAAAAATCTGCCAAACAATCTTAAATCCTTTGTCTGGTTCAAAGGAATGGTAGAAAGGGGCCATGAGATTGCACTGTATGTGGTGAATGATAAAGGAAGTGTCACAGGGCCTGAACACAGTGGCAGAGAGACAATCTACAGTGATGGATCCCTGCAGATCCGCAAGGTCACCCAGAAGGACATAGGATTCTACATTCTACAAACGTATAATATGCAAGAAAAAATTGTATCAACATCATCCGTATACCTCTACATGTACA

>Aca_Psg4N1 (Acomys cahirinus; Egyptian spiny mouse) WGS PVKX01008214.1

TTTCCATTTTAACCTGCTGTCATCCTCCCACCACTGCTGATGTCACTGTGGAGACAGTGCCACCTGAAGTAGTTGATGGAGAAAACATCTTCCTACTAATTAACGGTCTGCCAAAGAATATTATAGCCTTTGCCTGGTACAAAGGAGTACAGAATATGAGCCATGTCATTGCACTATATTCACTGAAATATAATACGACTGTGACAGGGCCTCTACACAGTGGTAGAGAGATACTGTACCGTAATGGGTCCTTGTTGATCCAAAATTTTACCCAAAAAGACAAAGGATTGTATACCCTACGAACCAAAAATAGTCGTGGAGAATTTGTATCAACAACAACCACGTACCTCCGCGTGCAAG

>Aca_Psg5N1 (Acomys cahirinus; Egyptian spiny mouse) WGS PVKX01008214.1

CTTTTATTTGTACCTGCTGGCACCCTTCCGTCACTAACTCTGTTACTGTTGAGCCAGTGCCACTCTATGTCCTTAACGGGGAAAACGTGCTTCTTCTCGCTACCAATCTGCTAGATGACATTATAGCTTTAGTGTGGCACAAAGGGGTGAAGAATCTGGACCATGGAATTGCACTTTATTCACTGCAATATAACGTGAGTGTGACAGGACCTGCACACAGTGGTAGAGAGACAATTTACCGCAACGGGTCCTTGTTGATCCAAAATGTTACCCTGAATGACAAAGGATTTTATACTCTACGAACCATAAATAGTAGTGGATATATTGTATCAACATCACAGCTGCACCTCCTAGTACTCG

>Aca_Psg6N1 (Acomys cahirinus; Egyptian spiny mouse) WGS PVKX01008214.1

CTTCTATTTGTACCTGCTGGCACCCTTCCATCACTAACTCTGTTACTATTGAGCCAGTGCCACTCTATGTCATTGAGGGGGAAAACGTGCTTCTTCTAACTACCAATCTGCAAGACGATATTATAGCCTTAGTCTGGCACAAAGGGGTGGAGAATCTGGACCATGGAATTGCACTTTATTCACTGCAACATAATGTGAGTGTGACAGGACCTGCACACAGTGGTAGAGAGACAATTTACCGCAATGGGTCTTTGTTGATCCAAAATGTTACTCTGAATGACAAAGGATTTTATACCCTACGAACTATAAATAGTAGTGGATATATTGTATCAACATCACACCTGTACCTCCTAGTACTCG

>Aca_Psg7N1 (Acomys cahirinus; Egyptian spiny mouse) WGS PVKX01008214.1

CTTCTATTTTAACTGGCTGGCACCCTCCCACCACTGCCGATGTCACTGTGGAGACAGTGCCACCGGAAGTGGCTGATGGAGACAACGTCCTTATACTTACTAACGGTCTGCCGAAGAATATAATAGGCTTCGCCTGGTACCAAGGGATACATAATTTGAACCTTACAATTATAATATATTCACTGAGATATAACGTGAGTATGGCAGGAACTGTATACAGTGGTAGAGAGACAATTTACCGCAACGGGTCCTTGTTGATCCAAAATGTTACCCGGAAAGACACAGGATTTTATACCCTACGAACCGTAAATAGTGATGGAGGATTTGCATCAACAACAACCATGCACCTCCAGGTGTACC

>Aca_Psg10N1 (Acomys cahirinus; Egyptian spiny mouse) WGS PVKX01014167.1

CCTCCCTTGTTACATGCTGGCACCTGTCTACCACTACCTATGTCAAGATCGAAATACTGCCACGCAAAGTGTTTGAGGGAGACAATGCCCTTCTATTTGCTAAAAATCTGCCAAAGAATCTTAAATCCTTTGTCTGGTTCAAAGAGAAGGTAGAAAAGAGCCATCAGATTGCACTGTATTATATGAATACTGAAGAAACTGTGACAGGGCCTGAACACAGTGGCAGAGAGACAACCAACAGTGATGGATCTCTGCAGATCCGCAAGGTCACCCAGAAGGACATAGGATTCTACATTCTACAAACGTATAATTGGCAAGAAAAAATTGTATCAACATCACCCATATACCTCTACGTGTACA

>Aca_Psg11N1 (Acomys cahirinus; Egyptian spiny mouse) WGS PVKX01013370.1

CTTCTATTTTAACCTGCTGGCACTCTCCCACCACTGCCTATGTTATGGTTGAGCCAGTCCCACGCCATGTGATTGACGGGGAAAACGTCCTTCTACTAACTACCAATCTGCCAGAGAATATTATAGCCTTTGTCTGGCACAAAGGGGTGAAGATTATGACCCATGGAATTGCACTCTATTCACTGCAATATAACGTGAGTGTGACAGGGCCTGCGCACAGTGGTAGAGAGAAAATTTACCGCAACGGGTCCTTGTTGATCCAAAATGTTACCCTGAATGACATTGGATTTTATACCCTCCGCACCATAAATAGTAGTGGACATATTGCATCAACAACGACCATGTTCCTCCTCGTATACG

>Aca_Psg15N1 (Acomys cahirinus; Egyptian spiny mouse) WGS PVKX01013370.1

CCTCCATTTTAACTTTCTGGTACCCACCCACAACTGATGAAATAACCGTTGAATTAGTGCCACCTCAAGTGGTTGAAGGAGAAAATGTCCTTTTCCTTGTTAATGATCTGCCAAATAATCTTATAACCTTCGCCTGGTTCAAACGGATGACAGATATGAACCTCAGAATTGCATTATATGCACTGAACAGTGATGCAAATGTGATGGGTGCTATATACAGTGGTAGAGCAACTGTGTACAGAAATGGATCTCTTGAGATCCACAGTGTCACCCAGAACGATATAGGATACTACACACTAAAAACCTTTAATAGACATGAAGAAATTGTATCAATAACATTCACCTACCTCCACGTGAACA

>Aca_Psg16N1 (Acomys cahirinus; Egyptian spiny mouse) WGS PVKX01022413.1

CGTCCCTTTTAACCTGCTGGCACCTGTCTACCACTACCAACGTCACCATTAAAGTAGTGCCTCCGTATGTTTTTGAAGGAGAAAATGTCCTTCTCTTTGCTAACAATCTGCCAGACAATCTTCTCGCCTTTGTCTGGTTCAAAGGGATGACAGAAAAGAGCCACATGATTGCAGTGTATGCAGTGAACACTAAAAGAAGTCTGATGGGGCGTGCACACAGTGGCAGAGAAACAATCTACAGTGATGGATCTCTGCAGATCTGCAATGTCACCCAGAAGGATATAGGATCCTACATTCTTAAAACCTATAATAGGCAAGCAGAGCCAGTATCAACAACATCCATATACCTCCACGTGTACA

>Aca_Psg17N1 (Acomys cahirinus; Egyptian spiny mouse) WGS PVKX01037727.1

CCTCCCTTTTAACATGTTGGCACCTGTCTACCACTACCAATGTCACCATTAAATTAGTGCCACAGCACGTATTTGAAGGAGAAGACGTCCTTCTCCTTCCTAAAAATCTGCCAGACAATCTTCTAGCCTTTGTCTGGTTCAAAGACATAACAAAGAAGAGCCACATGATTGCACTATATGCAGGGAACATTGAAGGAAATATGACAGGACGTGCAGACAGTGGCAGAGAAACAATCTACAGTGACGGATCTCTGCAGATCCGCAATGTCACCCAGAAGGACACAGGATTCTACATTCTTAAAACCTATAATTGTCAAGCAGAAACAGCATCAACAACATCCGTATACCTCCAAGTGTACA

>Aca_Psg18N1_P (Acomys cahirinus; Egyptian spiny mouse) WGS PVKX01037727.1

CCTCCCTTTTAAGCTGTTGGCACCCAAAACCACTACCCACATGACCATTACTTTTGTGCCATTTCTTTTGTTTGAAGGAGCAACTGTCATTTTCCATGTTAAAAATTTTGCAGACACTCATACATCCTTTGGCTGGTTCAAAGAGATAACAGGAAAGAGTCATGAGATTTCACTGTATGCAGCGAATGCTAAAGGAAATATGACAGGGCGTGCACACAGTGACAGAGAGACAATCTACAGTGATGGATCTCTGCAGATCCACAAGGTCACCCGGAAGGACACAGGATACTATATTCTAGAAGGCTATAATAGGCAAGCAGAACTTGTATCAGTAGCACCCATGTACCTCCTCGTCTACT

>Aca_Psg19N1_P (Acomys cahirinus; Egyptian spiny mouse) WGS PVKX01006019.1

CCTCTCTTTTAATATGCTGGCACCTGTCTACCACTTCCTATGTCACCATTAAATTAGTGCCACACCACGTGTTTGAATGAGAAAACGTCCTTCTCCTTGTTAAAAATCTGCCAGACAATATTATTGCCTTTATCTGGTTCAAAGGGATGACAGAAATGAGCCACGCATTTGCACTGTATTTCATGACTGCTAAAGGAAATGTAACAGGGTTTGCATACAGTGGCAGAGAGAGAATCTACAGTGATGGATCTCTGCAGATCCACAAGGTCACCCAGAAGGACACAGGATTCTACATTCTACAAACCTATAATGAGCAAGCAGAAATTGTATCAACATCATCCATATACCTCATTGTTTATC

>Aca_Psg20N1 (Acomys cahirinus; Egyptian spiny mouse) WGS PVKX01040301.1

CCTCCCTTTTAAGCTGCTGGCACCTGTCTACCACTATGAATGTCACCATTAAAGTAGTGCCTCCCCATGTGTTTGAAGGAACAAACATCCTTCTCCGTCCTAACAATCTGCCAGCCAATCTTTCAGCCTTTTCATGGTTCAAAAGGATGACACAAGAGAGCCATGCGATTGCAGTGTATGCGGTTTCTGCTAATAGAAATATGACGGGGCGCGCGCACAGCGGCAGAGAGACAATCTACAGCGATGGATCTCTGCAGATCCGCAACGTCACCTGGGAGGACACAGGATTCTACATTCTACAAACCCTTAATTGGCTAGGAGAAATTGTATCAACACCCATATACCTCCGTGTGTACA

>Ani_Ceacam9N (Arvicanthis niloticus; African grass rat) WGS JAAOMG010000027.1

CCTCCCTCTTAACCTGCTGGAATGCACTCGCCGCTGCTGAGCTCACTATTGAGTTAGTGCCACCCATGGTTGCAGAAGGCGGAAACTCCGTTCTGTTTGTGCACGAAATGCCGCTGAATGTCCAGGCGTTTTACTGGTACAAACAGAGAGGTCCGACCAAGAGCTATGAAGTCGCGCGGTACTTAACACCCACTAACGAAAGTTCGAAGATGCCTCAGCACAGTGGTAGGAAAACCGTATTCTACAGTGGATCCCTGCTGATCAGAAATGTCACCCAGGCCGACAGCGGAGTCTACACCTTACTAACATTCAACACAGAAATGGAAAGCGAATTAACACACGTGCATCTGGAAGTACGCG

>Ani_Ceacam11N1 (Arvicanthis niloticus; African grass rat) WGS JAAOME010000001.1

TCTCCCTTTTAACCTGCTGGCTGCTTCCCACTACTGCCAAGATCACCATTGAATCAATGCCTCCCATTGCTGTTGAAGGGGAAGATGTTCTTCTGTTTGTGCATAACTTGCCAAAGCAAGTTAAAGCCCTTTCCTGGTACACAGGAGTTGCAGCACTCAAGAGTTGTGAAATTGCAAGACATGTGATAGCTACCAATTCTAATGTGGTGGGACTTGCACACAGTGGTAGAGAGACAATACTCAACAGTGGATCTCTGGTGATCAAGAGTGTCACCAGAAAAGACTCTGGATACTATACCCTACAAATACTTCTTGATTCAATCTCAAGACCTAAAGTAATGCATGCAGAATTCTTTGTACACA

>Ani_Ceacam12N1 (Arvicanthis niloticus; African grass rat) WGS JAAOMG010000027.1

TTTCCCTTTTAACCTGCTGGCTGCTTCCCACTACTACTCAGCTCATCATTGAATCAGTGCCTCCCATTGCTGTTGAAGGGGAAAATGTTCTTTTGTTAGTGCATAATTTGCCAAAGAAGGTTAACGCCCTTTCCTGGTACATAGGAGATAAAGTGCTCAAGAGTTGTGAAATTGCAAGACATACGATAGCTACCAATTCTAGTGTGGTGGGACTTGCACACAGTGGTAGAGAGACAGTACTCAACAATGGATCTCTGCTGATTAAAAGTGTCACCAGAAAAGACTCAGGATACTACACTCTGGAAATACTTGATTCAGCCTCAAGACTTGAAATAATACATGCAGAATTCTTTGTACACA

>Ani_Ceacam13N1 (Arvicanthis niloticus; African grass rat) WGS JAAOME010000001.1

TTTCCCTTTTAACTTGCTGGCTTCTTTCCACTACTGCCCAGCTCACCATTGAATCAGTGCCTCCCATTGCTATTGAAGGGGAAAATGTTCTTGTGTTTGTGCAAAACCTTCCAAAAAATGTTAAAGCCCTTTCCTGGTACAGAGGAGCTAAAGCACTCAAGACTTTTGAAATTGCAAGACATGATATAGCTACCAATTCTAGTGTAGTGGGACCTGCAAACAGTCATAGAGAGACAGTACTCAACAGTGGATCTCTGCTGATCAAGAGTGTAACCAGAAAAGACTCTGGATACTACACCCTACAAATACTTCATACAACCTCAAAACCTGAAATAATGCGTGCAGAATTCTTCGTACAGA

>Ani_Ceacam15N (Arvicanthis niloticus; African grass rat) WGS JAAOMG010000027.1

CCTCACTTTTAACCTGCTGGAACTCCCCTGCCGCAGCACTGCTAACATCTAAAGAAATGCGGTTCTCGGCTGCTGAAGGGGCAAAGGTTCTTCTCTATGTTCCTGACCAGGAAGAGAACCTCCTCTCCTTTTCCTGGTACAAAGGGAAAGATGTAAATGAAAATTTTACCATTGCGCATTATAAAAAATCCAGTGGTTCACTTCAACTTGGAAAGAAAGTCAGCGGCAGGGAAGAAATCTATAAGGACGGCTCCATGATGCTCCAGGATGTCACCCAGGAAGACACGGGATTCTACACTTTAAAAACCTTTAAAGAACACAATCAACAGGAAATAACATATGCCCATCTCCAAGTATATA

>Ani_Psg36N1 (Arvicanthis niloticus; African grass rat) WGS JAAOMG010000027.1

CCTCCCTCTTAACCTGCTGGTTTCTGCCCACCACTGCCCAAGTTGCCATCGAACCCTTACCTCCCCAAGTGGTTGAAGGAGAAAATGTTCTTCTACGTGTTAACAATCTGCCAGAGAATCTTCTAGCCTTTGTCTGGTATAGAGGGGTGACAAATATGAGTCTCGGAATTGCACTGTATTCACTGACCTACAGCGTAACTGTGACGGGGCCCATGCACAGCGATAGAGAGACGTTGTACAGCAATGGATCCCTGTGGATCCACAATGTCACCCAGAAGGACACAGGATTCTACACTTTTCGAACCATAAGTAAACGTGGAGAAATTGTATCAAATACAACAGTGCATCTTCAAGTGTACT

>Ani_Psg38N1 (Arvicanthis niloticus; African grass rat) WGS JAAOMG010000027.1

CCTCCCTTTTAACCTGCTGGCTCCTGCCCGCCACTGCCCAGGTCACCATTGACTCAGTGCCATCAAATGTGGTTGAAGGAGAAAATGTCCTTCTTCTTGTTGACAATCTGCCACAGGATCTCATAGCCTTAGCCTGGTTCAGAGGAATGAAGAAAATTGTCGTATATATACTGAACACTAAAGTAAGTGTGACAGGGTCCATGTACAGTGGTAGAGAGACAGTGTCCAGCAACGGGTCCCTGTGGATCCACAATGTCACCCAGAAGGACACAGGATTCTACACCCTACGAACTGTAAATAGACGTGGAGAAATTGTATCAACAACATCCATGCATCTCTATGTGTACA

>Ani_Psg42N1 (Arvicanthis niloticus; African grass rat) WGS JAAOME010000001.1

CCTCCCTTTTAACCTGTTGGCACCTGTCTACCACTTCCAAAGTCACCATTGAATTAGTGCCACCACAAGTGGTTGAAGGGGAAGATGTTCTTTTCCTTGTTCATAATCTGCCAGAGAATCTTACAGCCTTTGCTTGGTTTAAAGGCAGGACAAATATGAAACGAGGAATTGCACTGTATGCATTGGCCTCTAACATACATGTACACAGTGATAGAGAGACACTGTATAGCAATGGATCCCTGATAATCCACAATATCACCCAGAAGGACAGAGATTATTATACCCTACGAACCTTCAATGGACATTTAAAGGCTGTATCAACAACAACCACATTCCTCCATGTGAACC

>Ani_Psg44N1 (Arvicanthis niloticus; African grass rat) WGS JAAOME010000001.1

CCTCCCTTTTAACCTGCTGGCACCTGCCTACCACTGCACAAATAACCATTGAATTAGTGCCACCCCAAGTGATTGAAGGAGAAAATGTTCTCATACGTATTGACAATCTGCCAGAGAATCTTGTAACCTTAGCCTGGTTCAGAGGAACAAGGATTAAGAGCCCGCAAATTGGACAATATACCCTGGCCACTAATACTACTGTGCTGGGGCCTGGTCACAGTGGTAGAGAGACTTTGTACAGCAATGGATCCCTGCAGATCTACAATGTCAACCAGGAGGATATAGGGTTCTACAGCCTACGAATCATAAACAGACATGCAGAGATTTTATCCATAACATCCATATATCTCAACGTGTACT

>Ani_Psg3N1 (Arvicanthis niloticus; African grass rat) WGS JAAOME010000001.1

CCTCCTTCTTAACCTGCTGGCTTCTGCCAACCACTGCCCAAGTCACCATCGAATCTTTACCATTCAAAGTGGTTGAAGGAGAAAATGTTCTTTTACGCGTGGACAATCTGCCAAAGAATCTTATAGGCTTCGCCTGGTACAGAGGGGTGAAAAATTTGAAGTTTGGAATTTCACTGTATTCACTGACCTATAGCATAAATGTGGAAGGGCCTACACACAGCGGTAGAGAGACATTGTACAGCAACGGGTCCCTGTGGATACAAAATGTCACCAAGGAGGACACAGGATATTACACCTTTCGAACCATAAGTAGACGTGGAGAAATTATATCAAATACATCCCTCCAACTTCATGTGTACT

>Ani_Psg5N1 (Arvicanthis niloticus; African grass rat) WGS JAAOMF010000002.1

CCTCCCTTTTAACCTGCTGGCTTTTGCCCACCACTGCTGGAATCACCATCAAATCCTTACCACCCAAAGTGGTTGAAGGAGAAAATGTTCTTCTACGTGTTGACAATCTGCCAGAGAATCTTCTAATGTTTGCCTGGTACAGAGGGGTGAGAAATTTGACACATGCAATTGCATACTATTCACTGCACCATAGCGCAAGTGTGAAGGGGCTGACCCACAGAGGTAGAGAGACATTGTACAGCAATGGGTCCCTGTGGATCCCAAATGTCACCCAGAAGGACATAGGATTCTACACTTTTCAAACCATAAGTAGACATGGAGAAATTGTATCAAATACATCAATGTTCCTTCTCGTGnnn

>Ani_Psg6N1_P_partial (Arvicanthis niloticus; African grass rat) WGS JAAOME010000001.1

CCTCGCTTTTAACCTGCTGGTTTCTGCCCACCATTGCCCGAGTCACCATTGAATCCTTTCCGATCAAGGTGGTTGAAGGAGAAAATGTTCTTCTACGTGTTGACAATATGCCAGAAAATCTTCTAGGCTTTGCCTGGTATAAAGGGTTGTCAAATATGAGGCTTGGAATTATACTGTATTCTGTGGTCTATAGCATAAGTGTAACAGGGCCCGAGCACAGTGGTCGAGAGATATTGTACAGCAACGGGTCCCTGTGGATCCCAAATATCACCCGGAAGGACACAGGATTCTACACTTTTTGAnnnnnnnnnnnnnnnnnnnnnnnnnnnnnnnnnnnnnnnnnnnnnnnnnnnnnnnnnn

>Asp_Ceacam9N (Apodemus speciosus; large Japanese field mouse) WGS BDUI01014618.1

CCGCCCTCTTAACCTGCGGGAACGCACCGGCCGCTGCCGAGCTCACTATTGAGTTAGTGCCGCCCGTGGTTGCCGAAGGCGGAAACTCGGTCCTGTTTGTGCATGCAATGCCACTGAATGTCCAGGCGTTTTACTGGTACAAACAGAGAGACTCAACGAAGAGCTACGAAGTCGCACGGTTCTTAACGCCCACTAACGAAAGTTCGAAGATGCCTCAGCACAGTGAGAGGAAAACCGTCTTCTACAGCGGATCCCTGCTGATCAGAAAYGTCACCCAGGCCGACAGTGGAGTCTACACCCTWCTAACGTTTAACACAGAAATGGAAAGCGAATTAACACACGTGCATCTGGAAGTACGGG

>Asp_Ceacam11N1_P_partial (Apodemus speciosus; large Japanese field mouse) WGS BDUI01033684.1

TCTCTCTTTTAACCTGCTGGCTCCTTCCCACTAGTGTCCAAATCACCATTGAGCCAGTGCCTCCCATTGCTGTTGAAGGGGAGAATGTTCT GTTTGTGCATGACCTGTGGGAGAATGTTAAAGCCCTTTCCTAGGACACAGGAGTTAAACCTCTCAAGGCTTGTGAAATTGCAAGACATGTGATAGCTACCAATTCTACTGTGGTGGGACTTGCACACAGTGGTAGAGAGACAGTACTCAACACTGGATCTCTGCTGATCAAGAGTGTCACCAGAAAAGAnnnnnnnnnnnnnnnnnnnnnnnnnnnnnnnnnnnnnnnnnnnnnnnnnnnnnnnnnnnnnnnnnnnnnnnnnnnnn

>Asp_Ceacam12N1 (Apodemus speciosus; large Japanese field mouse) WGS BDUI01133201.1

TCTCCCTTTTAGCCTACTGGCTGCTTCCATCTACTGCCCAGCTCACCATTGAATCAGTGCCTCCGATTGCTGTTGAAGGGGAAAATGTTCTTGTGTTTGTGCAAAACCTGCCAGAGAATGTTCAAGCCCTTTCCTGGTATACAGGAGGTAAACCTCTCAAGAGATTTGAAATTGCAAGACATGTGATAGCTACCAATTCTACTGTGGTGGGACCTGCACACAGTGGTAGAGAGACAGTACTCCCTAATGGATCTCTGCTGATCAAGAGTGTCACCAGAAAAGACTCAGGATATTACACTCTAMAAATACAGGATACAACCTCAAGACGTGAAATAATGCATGCAGAATTCTTTGTACAGA

>Asp_Ceacam13N1 (Apodemus speciosus; large Japanese field mouse) WGS BDUI01014547.1

TCACCCTTTTATCATCCTGGCTGCTTCCCACTACTTCAAARCTCACTATTAAATCAGTACCTCCCATTGCTGTTGAAGGGGAAAATGTTCTTCTGTTTGTGCAWAACCTGCCGAAGAATGTCAAAACCTTTTCCTGGTACACAGGAGTTGCACCATTCAAGTGTAGTGAAATTGCAAGTCATGCCATAGCTACCAATTTTACTGTGGTGGGACTTGCATACAGTGGTAGAGAGACAGTACTCAATAATGGATCTCTGCTGATCAAGAGTGTCACCAGAAAAGACTCAGGATACTACACTCTAAGAACACTTGATGCAACCTCAAGACCTGAAATAATTCGTGCAGAATTCTTTGTACACA

>Asp_Ceacam14N1 (Apodemus speciosus; large Japanese field mouse) WGS BDUI01247059.1

TCTCCCTTTTAGCCTGCTGGCTGCTACCCGCTACTGCCCAGCTCACCATTAAATCAGTACCTCACATTGCTGTTGAAGGAGAAAATGTTCTTCTGTTTGTGAATGACCTACCAAAGAATGTTAAAGCCTTTTCCTGGTACACAGGAGTTACAGCACTCAAGAGTTGTGAAATTGCAAGTCACACGATAGCTACCAATTTTAGTGTTGTAGGACTTGCACACAGTGGTAGAGAGACAATACTCAACRATGGATCTTTGCTGATCAAGAGTGTCACCAGAAAAGACTCAGGATACTACACTCTACGAATACTTGATGCAACCTCAAGACCTGAAATAATACATKCAGAATTCTTTGTACACA

>Asp_Ceacam15N (Apodemus speciosus; large Japanese field mouse) WGS BDUI01046038.1

CTTCACTTTTGATCCTCGGGAACTCGCCCGCTGCAGCACTGCAAACGTCTAAAGAAATGCGCTTCTCAGCTGCCGAAGGGGCAAAGGTTCTTCTCTATGTTCCTGACCAGGAAGAGAACCTCCTCTCCTTTTCCTGGTACAAAGGGAAGGATGCGAATGAAAATTTTACAATCGCACATTATAAAAAGTCCAGCGATTCACTTCAACTTGGAAAGAAAGTCAGCGGCAGGGAAGAAATCTTTAAGGACAGCTCCATGATGCTCCGGGCCGTCACCCAGGAAGATACAGGATTCTACACGTTAGAAACCTTTAAAGCACACGATCAACAGGAAATAACATATGTCCATCTCCAAGTGTACA

>Asp_Psg31N1 (Apodemus speciosus; large Japanese field mouse) WSG BDUI01123278.1

CCTCTTTTTTAACCTGCTGGCACCTGCCTACCACTGCCCAAATAACCATTGAATTAGTGCCACCCCAAGTGATTGAAGGAGAAAATGTCCTCATAGGTATCAACAATCTGCCAGAGAATCTTGTAGCCTTAGCCTGGTTCAGAGGAACAAGGATTAAGAGCCCTCAAATTGGACAATATATACTGGCCACTAATGTTACTGTGCTGGGGCCTGGTCACAGTGGTAGAGAGATTTTGTACAGCGATGGATCCCTGCAGATCTACAATGTCACMCAGGAGGATATAGGATACTACAGCCTACGGATGATAAATAGACATGCGGAAATTGCATCAATAGCATCCATATACCTCAACGTGTACT

>Asy_Ceacam9N (Apodemus sylvaticus; European wood mouse) WGS LIPJ01001818.1

CTGCCGCCCTCTTAACCTGCTGGAATGCACCCGCCACTGCCGAGCTCACCATCGAGTTAGTGCCGCCCATGGTTGCCGAGGGCGGAAACTCCGTTCTGTTTGTGCATGCAATGCCGCTGAATGTCCAGGCGTTTTACTGGTACAAACAGAGAGACTCAACGAAGAGCTATGAAGTCGCACGCTACTTAACGCCCACTAACGAAAGTTCGAAGATGCCTCAGCACAGTGATAGGAAAACCGTATTCTACAGTGGATCCCTGCTGATCAGAAACGTCACCCAGGCCGACAGTGGACTCTACACCTTACTAACGTTTAACACAGAAATGGAAAGCGAATTAACGCACGTGCATCTGGAAGTACAGG

>Asy_Ceacam11N1_P_partial (Apodemus sylvaticus; European wood mouse) WGS LIPJ01073102.1

TTCCCCTTTTAACCTGCTGGCTGCTTCCCACTAATGCCCAAATCACCATTGAATCAGTGCCTCCCATTGCAGTTGAAGGGGAGAATGTTCTGTTTGTGCATAACCTTTGAGAGAATGTTAAAGCCCTTTCCTAGTACCCAGGAGTTCAACCTCTCAAGACTTGTAAAACTGCAAGACATGTGATAGCTACCACTTCTACTGTGGTGGGACTTGCACACAGTGCAGAGAGACAGTACTCAACAATGGATCTCTGCTGATCACAAGTGTCACCAGAAAAGACTCTCGCTGATGCCTAGAAGCTCTTGCTGACAGGAGCCTGATATCACAATTCCTTGAGAGGCTCTGCnnnnnnnnnn

>Asy_Ceacam12N1 (Apodemus sylvaticus; European wood mouse) WGS LIPJ01011559.1

TTTCCCTTTTAGCCTGCTGGATGCTTCCGGCTACTGCCCAGTTCACCATTGAATCAGTGCCTCCCATTGCTGTTGAGGGGGAAAATGTTCTTGTCCTTGTGCAAAACCTGCCAGAGAATGTTCAAGCCCTTTCCTGGTATGCAGGAGGCAAACCTCTCCAGAGATTTGAAATCGCAAGACATGTGATAGCAACAAATTCTACCGTGGTGGGGCCTGCGCACAGTGGTAGAGAGACAGTACTCCCCAATGGATCTCTGCTGATCAAGAGTGTCACCAGAAAAGnnnnGGGATACTACACTCTAAAAATGCTTGATACAACCTCAAGACGTGAAATAATGCGTGCGGAATTCTTTGTACAGA

>Asy_Ceacam13N1 (Apodemus sylvaticus; European wood mouse) WGS LIPJ01023278.1

TCACCCTTTTAACATCCTGGCTGCTTCCTACTACTTCCAAGCTCACCATTAAATCAGTGCCTCCCATTGCCGTTGAAGGGGAAAACGTTCTTTTGTTTGTGCACAACCTGCCGAAGAATGTTAAAGCCGTTTCCTGGTACACAGGAGTTGCACCATTCAAGTGTAGTGAAATTGCAAGTCATGTGGTAGCTACCAATTTTACTGTGGTGGGACTTGCGCACAGTGGTAGAGAGACAGTTTTCAATAATGGATCTCTGCTGATCAAGAGTGTCACCAGAAAAGACTCAGGATACTACACTCTAAGAACAATTGATTCAACCTCAAGACCTGAAATGATTCGTGCAGAATTCTTTGTCCACA

>Asy_Ceacam14N1 (Apodemus sylvaticus; European wood mouse) WGS LIPJ01063045.1

TCTCCATTTTAACCTGCTGGCTGCTTCCCACTACTGCCAAGATCACCATTAAATCAATGCCTCCCATTGCTGTTGAAGGGGAACATGTTCTTCTGTTTGTGGATAACCTACCGAAGAATGTTAAAGCCTTTTCCTGGTACACAGGAGTTACAGCGCTCAAGAGTTGTGAAATTGCAAGTCACACCATAGCTACCAATTTTACTGTGGTGGGACTTGCACACGGTGGTAGAGAGACAGTATTCAACAATGGATCTCTGTTGATCAAGAGTGTCACCAGAAAAGACTCAGGACACTACACTCTACAAATACGTGATGCAACCTTAAGACCTGAAATAATACATGCAGAATTCTTTGTACACA

>Asy_Ceacam15N_partial (Apodemus sylvaticus; European wood mouse) WGS LIPJ01001818.1

CGTCACTTTTAATCCTCCGGAACTTGCCAGCTGCAGCACTGCCAGCATCTAAAGAAATGCGCTTCTCAGCTGCGGAAGGGGCAAAGGTTCTCCTCTATGTTCCTGACCAGGAAGAGAACCTCCTCTCCTTTTCCTGGTACAAAGGGAAGGATGCAAATGAAAATTTTACAATTGCACACTACAAAAAGTCCAGCGATTCACTTCAACTTGGAAAGAAAGTCAGCGGCCGGGAAGAAATCTTTAAGGATACTTCCAnnnnnnnnnnnnnnnnnnnnnnnnnnnnnnnnnnnnnnnnnnnnnnnnnnnnnnnnnAAGCACACGATCAACAGGAAATAACATATGTCCATCTCCAAGTATACA

>Asy_Psg16N1 (Apodemus sylvaticus; European wood mouse) WGS LIPJ01010956.1

CCTCCATTTTAACCTGCTGGCTCCTGCCCACCGCCCAGGTCACCATTGAACCGGTGCCGTTCAATGTGATTGAAGGAGAAGATGTCCTTCTTCTTGTCGACAATCTGCCAGAGAGTCTTGTAGTCTTAGCCTGGTTCAGAGGGCTGGGGAAAATTGTTGTATACATACCGAATATTAATGTAAGTGTGACAGGGCCCATGTACAGCGGTAAAGAGGCAGTGTCCAGCAATGGCTCCCTGTGGATCCGCAATGTCACCCAGAAGGACACAGGATTCTATACCCTACGAACCGTGAACAGAAATGGAGAAATTTTATCAACAACATCCATATACCTCCATGTGAACC

>Asy_Psg24N1_partial (Apodemus sylvaticus; European wood mouse) WGS LIPJ01010956.1

nnnnnnnnnnnnnnnnnnnnnnnnnnnnnnnnnnnnnnnnnnnnnnnnnnnnnnnnnnnnnnnnnnnnnnnnTTGAAGGAGAAAATGTTCTTCTACGTGTTGACAATCTGCCAGAGAATCTTCTAGCCTTTGTCTGGTACAAAGGGGTGACAGACATGAGCCTTGGAATTGCACTGTATTCGCTGGCCTATAGTGTAAGTGTGACGGGTCCTGCACACAGTGGCAGAGAGACATTATACAGCAACGGGTCCCTATGGATCCAAAATGTCACTCAGGAGGACACAGGATTCTACACCCTACGAACCATAAGCAAACGTGGAGAAATTGTATCAAATACATCCACGCACCTTCTTGTGTACT

>Asy_Psg29N1 (Apodemus sylvaticus; European wood mouse) WGS LIPJ01034300.1

TCTCCCTTTTAGCCTGCTGGCACCTGTCTACCACTTCTGAAGTCACCATTGAATTAGTGCCACCACAAGTGGTTGAAGGGGAAGATGTCCTTTTCCTTGTCCGTAATCTGCCAGAAAATCTTACAGCTTTTGTCTGGTCTAAAGGGAGGACAAATAGGAAACGTGGAATTGCACTCTATACAGTGGCCTCTGATATACATGTACACAGTGATAGAGAGACATTGTATAGCAATGGATCCCTGATGATCCACAATGTCACCCAGAAGGACAGAAATTATTATACTCTACGAACCTTCAATAAACATGCAGAAACTGTATCAACAACATCCACATTCCTCCATGTGAACC

>Asy_Psg31N1 (Apodemus sylvaticus; European wood mouse) WGS LIPJ01011559.1

CCTCCTTTTTAACCTGTTGGCACCTCCCTACCTCTGCCCAAATAACCATTGAATTAGTGCCACCCCAAGTGATTGAGGGAGAAAATGTTCTCATACGTATCAACAATCTGCCAGAGAATCTCATGACCTTAGCCTGGTTCAGAGGAATGAGGATTAAGAGCCCTCAAATTGGACAATATACACTGGCCACTGATGCTACTGTGCTGGGGCCTGGTCACAGTGGTAGAGAGATTTTGTACAGCGATGGATCCCTGCAGATCTACAATGTCACCCAGGAGGATACAGGATACTACAGCCTACGAATGATAAATAGACAAGAAGAAATTGTGTCAATAACATCCATATACCTCAACGTGTACT

>Cga_Ceacam9 (Cricetomys gambianus; Gambian giant pouched rat) WGS PVKD010020451.1

CCTTACTCTTGACCTGCTGGAATGCACCTGCCGCCGAGCTCACTGTGGAATTGGTACCACCCAGGGTGGCTGAAGGCGGAAACTCCATTCTCTTTGTGCATAAGATGCCGTTGAACATCCAGGCCTTCCACTGGTACAAACAGAAAGATCCTACGAAGAGCTATGAAGTTGCGAGATACTTAACACCCACCAACACAACTTCGCGGGTGCCGAAACACAGCGGTAGGAAAACAGTATTCTACAGCGGATCCCTGCTGATCAGAAACGTCACCCGGGCCGACAGCGGACTCTACACTTTACTGACATTTAATACTGAAATGGAAAGTGAATTAACGCACGTGTATCTGGAAGTACACA

>Cga_Ceacam15N (Cricetomys gambianus; Gambian giant pouched rat) WGS PVKD010035102.1

CCTCATTTTTAACCTGCTGGAACTCACCCACCGTGGCCCTACGAACCACTAAAGAAATGCGGTTCTCTGCGGCTGAAGGGGCCAAGGTTCTGCTCCATGTCCCTAATCAGGCAGAGGACCTTCAGTCCTTCCACTGGTACAAAGGCAAAGAGGAGAATAAAGAATTTACAATCGCATATTACAAAAAGACCGCAGATATAGTTGAACTTGGGAAGAAGATCAGCGGCAAGGAAGAAATATACAAGGATGGATCCATGATGGTCCAGAACGTCACCAAGCAAGACACAGGATTCTACACATTAGTCTTTGAGACACATGATCAGGAAGATATAACACATGTCTCCCTCCAAGTGCACA

>Cga_Psg1N1 (Cricetomys gambianus; Gambian giant pouched rat) WGS PVKD010016125.1

CCTCCCTGATGACCTGCCTATACCTGTCTACCACGGCCCTAGTCAGCATTGAGTCAGTGCCGGCCCAAGTGGTGGAAGGAGACAGTGTCCGTCTACGGGTCCACAGTCTGCCAGAGAATCTTATAGCCTTCGCCTGGTACAAAGGGATGACGAATTCGAGCGTCGTGATAGCACTATACACGCTGTCTGTGGGAAAGGATCCTGGTCCTGTACACACTGGTAGACATTCCATATACAGTGATGGATCCCTGTGGATCCGCAATGTCACCCAGAAGGACACAGGATTCTACACTCTACGGACCTTTAATAGAGATGGAAAAGTTGAATCAACAACGACCAAGTACCTCAAGGTGTATG

>Cga_Psg2N1 (Cricetomys gambianus; Gambian giant pouched rat) WGS PVKD010028963.1

CCTCCCTGATGACCTGCTGGCACCTGCCCACCATGGCCCTAGTGAGCATTGAATCAGTGCCTTCCCATGTGGCTGAAGGACACACTGTCCATCTACGGGTCCACAGTCTGCCCGATAATCTTATAGCCTTTGCCTGGTTCAAAAGGGTGGCGAACGTGAGCCTTGGCATCGCACTGTACGCACTGACCACCAAGGTAAACGTTTCTGGTCCTGTACACACTGGTAGAGAATCCATATACAGCGATGGATCCCTGTGGATCCGCAATGTCACCCAGAAGGACACAGGATTCTACACTCTACGGACCTTTAATAGATATGGAAAAGTTGAATCAACAACGACCAAGTACCTCAACGTGTATGGTAA

>Cga_Psg3N1 (Cricetomys gambianus; Gambian giant pouched rat) WGS PVKD010051549.1

CCTCCCTGATGACCTGCTGGCACCTGCCCACCACGGCCCTAGTGAGCATTGAATCAGTGCCTCCCCAGGTGGTTGAAGGAGACAATGTTCTTCTACAGGCCCACAATCTGCCTGATCATCTTATAGCCTTTGCCTGGTTCAAAAGGGTGGCGAACGTGAGCCTTGGCATCGCACTGTACGCACCGACCACCAAGGTAAACGTTTCTGGTCCTGTACACACTGGTAGAGAGTCCATATACAGCGATGGATCCCTGTGGATCCGCAATGTCACCCAGAAGGACACAGGATTCTACACTCTACGGACCTTTAATAGAGACGGAAAACTTGAATTAATAGCGACCATGTACGTCAAGGTGTATG

>Cgr_Ceacam9N (Cricetulus griseus; Chinese hamster) WGS RAZU02000010.1

CCTTCCTCTTAACATGCTGGAATACACCCACCACTGCCGAGCTCACTATTGAGCTAGTGCCCCCCAAGGTGGCTGAAGGCGGAAACTCGGTTCTATTTGTGCATCAAATGCCATTCAACGTCCAGGCATTTTACTGGTACAAACAGAAAGACCCAACCAAGAGCTATGAAGTTGCCAGGTACTTAACACCCGATAACACAACCTCAAAGATGCCTCAACACAACGGTAGGAGAACGGTATTCTACAGCGGCTCCCTGCTGATCAGAAACGTCACCCAGGCCGACAGTGGAGTCTACACCTTACTAACATTCAACACAGAAATGGAAACGGAACTAACACACGTGCATCTGGAAGTACACG

>Cgr_Ceacam11lN1 (Cricetulus griseus; Chinese hamster) WGS RAZU02000010.1

CCTCCCTTTTAATCCTCTGGCTGCCTTCTACTGTTGCCCAGCTCACCATTGAATCAGTGCCGCCAATTTCTGCTGAAGGGGATAATGTTCTTCTTTTTGTACATAATCTGCCTGAGAATGTTCAAGCCTTTTCCTGGTACACAGGAGTTATGGCGCTCAAGAGCCGTGAAATTGCAAGGTATGAGATAGCTACCAATTCACGTACTCTCGGAACTGCACACAGCGGCAGAGCGACAGTATTCAATAATGGATCTCTGCTGATCAAGAATGTCACCAGGAAGGACTCAGGATACTACATCCTACAAACACTCGATACACATTTGCGATCTGAAATAACACGTGTGGAATTTTTTGTACATG

>Cgr_Ceacam15N (Cricetulus griseus; Chinese hamster) WGS FYBK01051609.1

CCTCACTTTTAGCTTGCTGGACCTCACCCACCAAGGCGCTAAGAACTACTAAAGAAATTCGGTTCTCTGCTGCCAAAGGAGCCAGGGTTCTTCTCTCTGTTCCTAGCCAGGCAGTGAACCTTCTCTCCTTTCACTGGTACAAAGGGAAAGATGCAAACGAAGATTTTACAATTGCCCATTATGAAAAGGCCAAGGGTTCACTTAAACTTGGGAATCAAATCAGCGGCAGGGAAGAAATATATATGGATGGATCCATGCTGCTCCAGAATGTCACCCAGGAAGACACCGGGATCTACACCCTAGAAATCTTTGGATCAGATGACCTCTATGAAATAACATATGTCCACCTTCAAGTGTACA

>Cgr_Psg1N1 (Cricetulus griseus; Chinese hamster) WGS RAZU02000031.1

CCTCCCTTCTAAACTTCTGGCTCCTGTCCACCGGTGCCCACATGACCACTGAATTAGTGCCACTCCAAGTGGCTGAAGGAGAAAACGTCCTTTTTCTTGCTCACGATCTTCCTGAGAGTCTGACAGCCTTCGCCTGGTTCAAAGGGCTAAGAAGCACAAAAAAAGGAATTGCACTGTATTCACTGTACCAGAATGTAAGTGGGCCAGGGCCTGTGCACAGTGGTAGAGAGACAATATATCGCAATGGATCCCTGCTGCTGGAAAAGGTCACCCAGAAGGACTCAGGATTCTATACCCTACGAACCTATAATATACGTGGAAAACTCATAGCAACCACATCTATATACCTTCACGTGCACG

>Cgr_Psg2N1 (Cricetulus griseus; Chinese hamster) WGS RAZU02000010.1

CCTCTCTTTTAACCTGCTGGCACCTCTCTGCCACAGCCCACGTCACCATTGAATCTGTCCCACCCCAAGTGGTTGAAGGAGAAAATGTCCTTTTCCTTGTCCACAATCTACCAGATGATGTTCTAAACTTGGCTTGGGCCAAAGGGGTGAATACTATGCATCTTGGAATTGGAACATATTTGCAGAGTGAAAATTTAAGTGTGCCAGgtactgaaagcactggtagagAATCAGTGTACAGTAACGGATCCCTGCTGCTCAGAAATGTGACAAAGAAGGACACAGGATTCTATACCCTAAGAGTCTTCAATAGTCGTGTGTCTATTGTGTCAACAACAACCATTTACCTTCATGTGCACA

>Cgr_Psg3N1 (Cricetulus griseus; Chinese hamster) WGS RAZU02000010.1

CCTACCTGCTATCCTTCTGGCACCTGCCCACTGTTGCCCAAGTGGCCACTGAATTAACACCACCCCTAGTGGCCGAAGGAGATAACGTCCTTGTCCTTGTCCACAATCTGCCAGAGAATCTTCTAGCCTTAGCCTGGTTCAAAGGACTAACAGACAAGAAAAAAGGAATTGCAATATATGGATTTCACAAGAATTTAAGTGCAACAGGGCCTGGGCACAGTGACAGAGAGACAATATATCGCAATGGATCTCTGCTGTTCGAAAAGCTCACCAAGAAGGACACAGGATTCTATACCTTGAGAACCTATAATAGACTCGGAAAAATCGTATCAACAACATCCATATACCTCCACGTGCATG

>Cgr_Psg4N1 (Cricetulus griseus; Chinese hamster) WGS RAZU02000031.1

CCTCACTTTTAACCTTCTACCACCTGTCCACCACTGCCCATGTGATCATTGAATCAGTGCCACCCCTAGTGGCCAAAGGAGATGATGTCCTTTTCCTGGTCCGAGATCTGCCAGAGAATATTCAAACCTTAGCCTGGTTCAAAGGGCTAACAAATACAACAGACAAAATTGCAGAATATGGACTGTTCAACAATGTAACTGAGCCAGGGTCTGTACACAGTGGTAGAGAGACAATATATCTCAACGGATCCCTGCTGATTGAAAAGCTCACTGAGAAGGACATAGGATTCTATACCCTACGAACCTATGATGAACATGCAAAAATTGTATCAACAACACCCACATACCTCCATGTGCAGG

>Cgr_Psg5N1 (Cricetulus griseus; Chinese hamster) WGS RAZU02000031.1

CCTCCCTTTTAAACTGCTGGCATCTCTCCACCACGGACTACATCACTCTTCGATCTGTCCCACCCCACGTGGCCAGTGGAGAAAACGTCCTTCTCTTTGTCCACAACATGCCAGAGGATATTCTAGCCTTTGCCTGGTTCAAAGGCGTGACAAGCATGAAGCATGGAATTGCAATGTATGAACTGCACAAGAATTTAAGTGTCACAGGGCCCACACACAGTGGAAGAGAGACAGTATATCGCAACGGATCCCTGCTCCTGGAACGTGTCACTGAGAGGGACTCAGGATTCTACACCCTACAAACCTTAGACAGACAGGGAGAGATTGTGTCAACAACAAACATGCGCCTTCACGTGTACC

>Cgr_Psg6N1 (Cricetulus griseus; Chinese hamster) WGS RAZU02000031.1

CCTCCTTTTTAACCTTCTGCCACCTGTCCACCACTGCCCATGTGACTGTGACCACTGAATCAGTGCCACCACTAGTGGCCAAAGGAGATGACGTCCTTTTCCTTGTCCACGAACTGCCAGAAAATATTGAAGCCATAGCCTGGTTCAAAGGGCTAACAAATATGAAAGACGCAATTGCAGTATATGGACTGTTCAACAATGTAAGTGGGCCAGGGCCTGTGCACAGTGGTAGAGAGACAATATATCGCAATGGATCCCTGCTGATTGAAAAACTCACTGAGAAGGACACGGGATTCTATACCCTACGAACGTATAATAGACATGTAAAAATTGTATCAACAACATCCACGTACCTCCATGTGCAGG

>Cgr_Psg7N1 (Cricetulus griseus; Chinese hamster) WGS RAZU02000031.1

CCTCCCTTTTAACCTGCTGGCACCTGACCACCACTGCCCAAGTCGCCATTGAATCAGTGCCGCCCCAAGTGGTTGAAGGAGAAGACGTCCTCCTACGTGTTCATCATCTACCAGACAATATTCTAGCCTTTGTCTGGCACAAAGGGGTGAGCAATATGAGCCTTGGGATTGCACTCTATTCACTGGCCAAGGATGGAAGTGTGACAGGGCCCGAACACAGTGGTAGAGAGACGGTGTACAGCAACGGATCCCTGCAGATCCGCAATGTCACCCGGAAGGACACCGGATACTACACCTTCCGAACCATAAATGGACAAGTAGGTATAGTATCAACGACAACCATATACCTTCACGTGTACG

>Cgr_Psg8N1 (Cricetulus griseus; Chinese hamster) WGS APMK01151744.1

CCTCTCTTTTAGCATGCTGGCACTTCTCCACCAGCACTGCACAGGTCACCATTGAATCTGTCCCACCCCAAGTGGTCGAAGGGGAAAACGTCCTTTTTCTTGTCCACAATCTGCCAGAGAATCTTATAGCCTCAGTCTGGTTCAGGGGGCTGAAAATTACAGAAAATGTAATTGCAATCTATGATCTGAAGCAAGATTTTAGTGCTCCAGGGCCTATACATAGTGGTAGAGAGACAGTGTACCGCAATGGATCTCTGTTGCTCAGAAATGTCACGATGAAGGACACAGGACGCTATACCCTGAGAACCATAAATAGACATGGAGATATCGTGTCAACAACAATCATGAACCTTCATGTGCACA

>Cgr_Psg9N1 (Cricetulus griseus; Chinese hamster) WGS RAZU02000010.1

CCTGCCTTTTAACCTCCTGGCATCTGTCCACGGCTCATGTGACCACTGAATTAGTGCCACGCCAAGTTGCCGAAGGAGATAACGTCTTTTTCCTTGTCCATGGTCTGCCTGAGAATATTATAGGCTTAGCCTGGTTCAAAGGGCTAAGAAATATGAAGCAAGGAATTGCAACATATGCACTGGACAGGAATCAAAGTGAGCCAGGGACGGTGTACAGTGGTCGAGAGACAATATATCACAATGGATCTTTGCTGTTGAAAAATGTTAACCATAAGGATTCAGGATTCTATACCTTACGGACCTATGATAGAAATGGAAAAATTGTATCAACAACATCCACATATCTCGATGTGCAGG

>Cgr_Psg10N1 (Cricetulus griseus; Chinese hamster) WGS RAZU02000010.1

CCTCCCTTCTAACCTGCTGGCATCTGTCCACCACTGCCAAAGTCACCATTGAGTCAGTGCCATCTCCAGTGATTGAAGGGGAAAACGTTCTTCTAAGTGCCAAAAATCTGCCAAACAATCTTGTAGCTTTTGCCTGGTTCAAAACCGTGAAGACAATGAGGCGTGGAATTGCACTATATACTCTGACCACTAATGTAAGTGCGGCAGGGCCTCAATACAGTGGCAGAGAAACTTTGTTTAGCAATGGATCCCTGTGGCTCACAAATGTCACCCCGAAGGACAAAGGATTCTATACCCTAAGGACAATACATAAAAGTGGAAAAATTGTATCTACAACAAGGATGTACCTCCATGTGTACA

>Cgr_Psg11N1 (Cricetulus griseus; Chinese hamster) WGS RAZU02000010.1

CCTCCCTTCTAACCTGCTGGCATCTGTCCACCACTGCCAAAGTCACTATTGAGTCAGTGCCATCTCCAGTGATTGAAGGGGAAAATGTTCTTTTCAGTGCCAAAAATCTACCAGAGAATCTTCTTGCTTTTGCCTGGTACAAAAGAGTGAAGTCAATGAGGTTCGGAATTGGACTATTTTCCCTGACCTCTAATCTAAGTGCAACGGGGCCTGGATACAGTGGTAGAGAAACTTTGTATCGCAATGGATCCCTGTGGCTCACAAATGTCACCACGAAGGACAAAGGATTCTATACCCTAAGAACAATAAATAGAAGTGGAAAAGTTGTATCTACAACAAGGATGTTCCTACGTGTGTACA

>Cgr_Psg12N1 (Cricetulus griseus; Chinese hamster) WGS RAZU02000010.1

CCTCTCTTGTAACATTCTGGCACTACCCCACCACAGCTGAACATGTCACTATTGAATCTGTCCCACCCCATGTGGTCGAAGGAGAAAATGTCCTTCTTCTCGTCCAGAATCTGCCAGAGAATCTTGCAACCTTGGTCTGGTCCAAAGGGGTAAAAATATCAAACAATGTAATTGGATTATATGACCTGAACAAAGATGTAAGTGCTCCAGGGCCTCTACACAGTGGTAGAGAGACAGTGTACAGTAACGGGTCTCTGCTGCTCAGAAATGTCACATGGAAGGACACAGCACTGTATACCCTAAAATGCTTAAATAGACATGGAGATACTGCATCAACAACCACGATTTACCTTGAAGTGCACA

>Cgr_Psg13N1 (Cricetulus griseus; Chinese hamster) WGS RAZU02000010.1

CCTCCCTTTTAGCATGCTGGCACTTCCACACCACCAGTGCAAGTGTCAACATTGAGTCCGTCCCACCCCATGTGGTTGAAGGAGAAAACGTCCTTCTTCTTGTCCACAATCTTCCAGAGAATCTTTCAACCTTGGTCTGGTCCAAAGGGGAGAATATTAGAGACAATGCGATTGGATTATATACACTGAGCCAAGATGTAAGTGCTCCAGGGCCTCTACACAGTGGTAGAGAGACAGTGTACCACAATGGATCTCTGCTGCTCAGAAATGTCACAAGGAAGGACACAGGATTCTATACCCTACAAAGTTTAAATAGTCATGAAGATATTGCATCAAGAACAACAATTTACCTTCATGTGCACA

>Cgr_Psg14N1 (Cricetulus griseus; Chinese hamster) WGS RAZU02000010.1

CCTCCCTTTTATCCTTATGGCACCTGCCCACCACCGAACAGTTTCCCAGCGATTCAGTGCCAGCTCTAGTGGCTGAAGGAGACAATGTTCTTCTCCCTGTACGTAGTCTGCCGGCGAAAATTAAATCTATAACCTGGTACAAAGAGATAAGAAATGAGACAAAAGAAATTGCAGTATATACACTGCGCAATAATTTAAATAAGCCAGGGCCGGCACACAGCGGTAGAGAGACAATATATCGCAATGGAACTCTGCTGATTGAAAAGGTGAACACGAAGGACACAGGATTTTATACTCTACGCACCCGTAATAGACACGGGAAAATTGTATCAACGTCAGTCAAGTACCTCGACGTAATAC

>Cgr_Psg15N1 (Cricetulus griseus; Chinese hamster) WGS RAZU02000010.1

CCTCCCTTTTATCCTTCTGGCACATGTCCACCACTGCCCATGTGACCACTGAATTAGTGCCACCCCATGTGGCGGAAGGAGAAAACGTCCTTTTGGCTGTCCATGGTCCCCCAAAGAATCTTAGAGCCTATGCCTGGTTCAAAGGGCTACCAAACACAACTCGAGGAATTGCGGTGTATACACTGCATAACAATTTAAGTGCTCCTGGGCCTCTACACAGTGGTAGAGAGACAGTGTATCACAATGGATCCCTACTGCTTGAAAATGTCACCCAGAAGGACACAGGAATCTATACCCTACAAACCTATAATACACATGGGGAAATCTTATCAACAACATCCAGGTACCTCCGAGTACACG

>Cgr_Psg16N1 (Cricetulus griseus; Chinese hamster) WGS RAZU02000010.1

CCTCCTTTTTAACCTCCTGGCACCTGTTCACGACTGCCCATGTGACCACTGAAGCAGTGCCACCCCTAGTGGCTGAAGGAGATAATGTCCTTTTCCTAGTCCATGATCTGCCAAAGAAAATTAAAGCCTTAACCTGGTTCAAAGGTCTAACAACTATGACAGAAGATATTGCAACATATGAACTGCGCAAAAATTTCAGTCAGCCAGGGTCTGTGCACAGTGGTAGAGAGACAATATATCACAACGGATCCCTGCTGCTTGAAAAGGTTAACCTGAAGGACACAGGATTCTATACCCTACGGACCTATAATAGACGTGGAAAAATCTTAACAACAGCAACCCTGTACCTCCGTGTGCACA

>Cgr_Psg17N1 (Cricetulus griseus; Chinese hamster) WGS RAZU02000031.1

CCTCCCTTTTAACCTGCTGGCACCTGACCACCACTGCCCAAGTCGCCATTGAATCAGTGCCGCCCCAAGTGGTTGAAGGAGAAGACGTCCTCCTACGTGTTCATCATCTACCAGACAATATTCTAGCCTTTGTCTGGCACAAAGGGGTGAGCAATATGAGCCTTGGGATTGCACTCTATTCACTGGCCAAGGATGAAAGTGTGACAGGGCCCGAACACAGTGGTAGAGAGACGGTGTACAGCAACGGATCCCTGCAGATCCGCCATGTCACCCGGAAGGACACCGGATACTACACCTTCCGAACCATAAATGGACAACTAGGTATAGTATCAACGACAACCATATACCTTCACGTGTACG

>Cgr_Psg1P1N1 (Cricetulus griseus; Chinese hamster) WGS FYBK01051247.1

CCTCCCTTTTAACCTGCTGGCACCTGACCACCACTGCCCAAGTCGCCATTGAATCAGTGCCACCCCAAGTGGTTGAAGGAGAAGACGTCCTCCTACGTGTTCATCATCTACCAGACAATATTCTAGCCTTTGTCTGGCACAAAGGGGTGAGCAATATGAGCCACTCTATGCACTGGCCAAGGATGGAAGTGTGACAGGGCCCGAACACAGTGGTAGAGAGACGGTGTACAGCAACGGATCCCTGCAGATCCGCAATGTCACCCGGAAGGACACCGGATAATACACCTTCCGAACCATAAATGGACAAGTAGGTATAGTATCAACGACAACCATATACCTTCACGTGTACG

>Elu_Ceacam9N (Ellobius lutescens; transcaucasian mole vole) WGS LOEQ01004837.1

CCTTCCTCTTAACCTGCTGGAATGCACCCACCACTGCCGAACTCACTATTGAATTAGTGCCCCCCATGGTTGCTGAAGGTGGAAACTCCGTCCTATTTGTGCATAAAATGCCGTTGAACGTCCAGGCATTTTACTGGTACAAACAGAAAGATCCGACCAAGAGCTATGAAGTTGCACGCTACTTAACACCCGATAACACAACGTCGAAGATGCCTCAACACAGCGGTAGGAAAACAGTATTCTACAGTGGATCCCTGATGATCAGAAATGTCACCCAGGCTGACAGTGGATTCTACACCTTACTAACGCTCAACACAGAAATGCAAAGTGAACTCACACACGTACATCTGGAAGTATACA

>Elu_Ceacam11lN1_P (Ellobius lutescens; Transcaucasian mole vole) WGS LOEQ01013171.1

CCTCACTTTTAACCTGATGGCTGCCTCCTACTGTTGCCTGTCTCACCATTGAATCAGTGCCACCCATTTCCGCTGAAAAAGATATGTTTGTGCACAATCTACCTGAAAATGTTCAAGTCTTTTCCTGGTTCACAGGAGTGACGGTAATCAAGAGCCATGAGATTTCAAGATATGTGGTAGCTACCAGTTCATGTATTCTGGGGCCTGCACACAATGGTAGAGAGACAGTTTTCAATAATGTATCTCTGCTGATCAAGATGTCACCAGGAAGGACTACTCATGGTTCTACATCCTACAAACACTTAGTACAAATTTGATATCTGAAATAATGTGTGGAGTTTTTGTATATT

>Elu_Ceacam15N_P (Ellobius lutescens; Transcaucasian mole vole) WGS LOJG01153097.1

GCATATTTTGGGCCTACACCGGACCTCATGCACCATGGCACTAAGAAATACTAAATAAATGTGGCTGTCTGCTGCCGAAGGGGAGAAGGTTCTTCTCTATGTTCCTAATCAGGCAGAGAACCTGCTCTCCTTCCACTGGTACAAAAGGAAAGATGTATCAAAGATTTTACAATTGCCCATTATGAAAAGGCCATGGACTTACTTAAACTTGGGAATAAAACCTGCAGCAGGGAAGAAATATCTAAGGGTGGCTCCATGATGATCCAGAATGTCAACCAGGGAGAAACAGGGATCTACACCCTAGAAAGCTTGGGAACACATAATCTCTATGAAATAATACGTGTTCACCTCCAAGGGTAA

>Elu_Psg1N1 (Ellobius lutescens; Transcaucasian mole vole) WGS LOEQ01009529.1

CCTTCCTTTTAACCTGTTGGCACCTGCCCACCACTGCCCAAGTCACCATTGACTCAGTGCCGCCCCAAGTGGTTGAAGGAGAAAACATTCTTCTACGTGTTCATAATCTACCAGAGAATCTTCTAGCCTTTGTCTGGCACAAAGGGGTGAGGAATATGAGCCTTGGAATTGCTCTATATTCACTGGCCAAGGGTTTAAGTGTGACAGGACCTGTACACAGTGGTAGAGAGACAGTGTACAGCAATGGATCCCTGAAGATCTACGATGTCACCCAGAAGGACACAGGATTCTACACCTTTCGAACCATAAATGGACATGTAGGAGTTGTATCAATAACAACCATGTACCTTCACGTGTACA

>Elu_Psg2N1_P_partial (Ellobius lutescens; Transcaucasian mole vole) WGS LOEQ01018097.1

CCTACCTTTTAACCTGTTGGCACCTGCCCACCACTGTCCAAGTCATTGTTGATTTAGTGCCACCTCATATTGTTGAAGGAGAAGATGTCCTTCTTCGTGTCCACAATCTGCCTGAGGATCTTGTAGCCTTTGTCTGGCACAAAGAGGTGACAAAGATGAACCTGGGAATTGTACTTTATTCACTGACCACTAATTTAAACATCATGGGGCCTGGACATAGTGGTAGAGAGATAGTGTACAGAAATGGATCTCTGCACCTCCAAAATGTCACCCAGAAGGACACAGGATTCTACACACTATGATCTTTAAATAAGCATAAAGGAATTGTAnnnnnnnnnnnnnnnnnnnnnnnnnnnnnnn

>Elu_Psg3N1 (Ellobius lutescens; Transcaucasian mole vole) WGS LOJG01018903.1

CCTCCCTTTTCTCCTCCTGGCATCTCCCCACTACTGCTCATGTGTCCACTGAATCAGTGCCACCCCTAGTGGCTGAAGGAGATAACATCCTTATCCTTGTCAACAATCTGCCAGAGAATCTTTTAGCCTTAGCCTGGTTCAAAGGGCTAACAAATATGAAACAAGGAATTGCATTATATGCACTGCACAAAAATGTAAGTGCTACAGGGCCTGTGCACAGTGGCAGAGAGACAATATATCACAATGGATCCTTGTTGATTGAAAAACTCACCCAGAAGGACACAGGATTCTACACCTTTAAAGCCTATAATAGACATGGAAGAATTGTATCAACCACATCCACCTACCTCCATGTGCAAG

>Elu_Psg4N1 (Ellobius lutescens; Transcaucasian mole vole) WGS LOEQ01015434.1

cctcacTTTTAGGCTGCTGCCTATCCACCACTGACTATATCACCATTAAATCTGTCCCACCCCATGTGGCCAGTGGAGAAGACGTCTTTATCCATGTCCACAATCTGCCAGAGGATCTTTTTGCCTTCGCCTGGTTCAAAGGGCCGACAAGCATGAACCACGGAATTGCAGTATATGCACTGAACAAAAATTTAAGTGCGACAGGGCCTGCACATAGTGGTAGAGAGACAGTGTACCACAATGGATCCATGCTGCTCCGAAGTGTCACCCAGAAGGACACAGGATTCTATACCCTACGAACCTTAGGCAGACACGGAGAGATTGTATCAAAAACAATCACGTTCCTCTATGTGCATC

>Elu_Psg6N1 (Ellobius lutescens; Transcaucasian mole vole) WGS LOEQ01015114.1

TCTCCCTTTTAACCTCCTGGCACCTGTCCACCGCTGTCCATATAACTACTGAGCCAGTGCCAACCCGAGTGGTTGAAGGAGAAAACGTCCTTTTCCTTGTACATGATCTGCCAGATAATACTAAATCCTTAGTCTGGTTGAAAGCTCTAAGAAATATGACAGAAGAAATTGCAGCATATGCACTGCCCTACAATTTAAGTAGGCCAGGTCCTCTGTACAGTGGTAGAGAGACAATATATCTCAATGGATCCCTGATGATAGAAAATGTCAACCTCAAGGACACAGGATTCTATATCCTACGAACCTATAACAGATGTGAAAAAGTCATATCAACAACAACAATGTACCTCCAAGTGAATG

>Elu_Psg7N1 (Ellobius lutescens; Transcaucasian mole vole) WGS LOEQ01013819.1

CCTCCCTTTTAACCCTCTGGCACCTGTCCATCACTGCCTCTGTGACCATTGAATCAGTGCCACCCCTGATGGCCGAAGGAGATAACATTCTTTTCCTTGTCGACAATCTGCCGGAGAAGACGGTAACCTTAGTCTGGTTCAAAGGGCTAACAAATATGAAAGCTGTAATTGCAATATATGGACGGCACATCAATTTAAGTGCATCTGGGCCTTTGCACAGTGGTAGAGAGACAATATATTACAACGGATCCCTGCTGATTAAAAAGGTTGTCCAGAAAGACACAGGATTCTATACCCTACGAAGCTATGATAAGTATTTAAACATCATATCAACAACATCCACATACATCCATGTTCATG

>Elu_Psg8N1 (Ellobius lutescens; Transcaucasian mole vole) LOEQ01001997.1

CCTTCCTTTTAACCTGTTGGCACCTGCCCACCACTGCCCAAGTCACCATTGACTCAGTGCCGCCCCAAGTGGTTGAAGGAGAAAACATTCTTCTACGTGTTCATAATCTACCAGAGAATCTTCTAGCCTTTGTCTGGCACAAAGGGGTGAGGAATATGAGCCTTGGAATTGCACTATATTCACTGGCCAAGGGTTTAATTGTGACAGGACCTGTACACAGTGGTAGAGAGAGAGTGTACAGCAATGGATCCCTGAAGATCTACAATGTCACCCAGAAGGACACAGGATTCTACACCTTTCGAACCATAAATGGACAAGTAGGAGTCTCATCAAAAACAACCACATACCTTCACGTGTATA

>Elu_Psg11N1 (Ellobius lutescens; Transcaucasian mole vole) WGS LOEQ01001997.1 CCTCCCTTTTAACTTACTGGTACCTACCCACCACTACCCAAGTCATCATTGAATTAGTGCCACCCAACGTGTTCCAAGGAGAAAATGTCCTACTAGAGGTCCACAATCTGCCAGAGGATTTTCTAGCCTTTGCTTGGTACAGAGGGGTGACAAACATGAAATGCGGAATTGCAGTCTATGCCAAAAGAAACAGTTTAAGAGCACTGGGGCCTGCACGCAGTGGTAGACAGGCAGTGTACAGTGATGGATCACTGCTGCTTCAGCGTGTCATCCTCAAGGACACAGGATTCTACACCCTACAAGTCATAAGTAGACAAGGAGAAATTGTATCAACAACATCCATGTTCCTCCATGTGCAGA

>Elu_Psg13N1 (Ellobius lutescens; Transcaucasian mole vole) 29.5.21 WGS LOEQ01013819.1

CCTTCCTTTTAACCTTTTGGCACCTGTCCACCACTGTCCATGTGACCACTGTGTCAGTGCCACCCATAGTGTCTGAAGGAGATGACGTCCTGTTCCTTGTCCACAATCTTCCAGAGGAAATTGAATCCTTAGCCTGGTTCAAAGGGCTAGGAGATGAGGCAGAAGAAATTGCAACATATGCACTGCACAGCGGTTTAAGTAGGCTAGGTCCTGCGCACAGCAGTAGAGAGACAATATATCACAACGGATCCATGCTATTTGAGAAGGTCAACCTGAAGGACACAGAATTCTATACCCTACGAGCTTATAATACAAGTGGAAAAATCGTATCAACAGCAAACGTGTACCTCAATGTGTATG

>Elu_Psg14N1 (Ellobius lutescens; Transcaucasian mole vole) WGS LOEQ01014926.1

CCTCCCTTTTAACCTTCTGGCACCTGTCCACCACCGCCCATGAGACCACTGTATCAGTGCCACCCCGAGTGGCTGAAGGAGATGATGTCCTGTTCCTTGTCCACAATCTGCCAGAGAAAATTAGATCCTTAGCCTGGTTCAAAGGGCAAGGAAACAGAACAGAAAAAATTGCAGCATATGCACTGCACAACAATTCAAGTAGACCAGGTCCTGCTTACAGCAATAGAGAGACAATATATCACAATGGATCCATGCTGTTTGAGAAGGTCATCCTGAAGGACACAGGGTTCTATACGCTACAAACCTATAACAGACGTGGAAAAATTGTATCAACAACAAATGTGATCCTCAATGTGCATG

>Elu_Psg15N1 (Ellobius lutescens; Transcaucasian mole vole) WGS LOEQ01015329.1

CCTTCCTTTTAACCTTCTGGCACCTGTCCACCACTGCCTTTGTGACCACTGTATCAGTGCCACCCCGAGTGGCCGAAGGAGATGACGTCCTATTCCTTGTCCACAATCTGCCAGAGAAAATTAAAACCATAGCCTGGTTCAGAGGGTCCTCAAATATGACTGCAATATATGGACTGCCCAACAATTTAAGTAGGCCAGGTCCTGCACACACTGGTAGAGAGACAATATTTCACAATGGATCCATGCTCCTTCAAAAGGCCAACCTGAAGGACACGGGCTTCTATACTGTACGAACCTACAATAGACATGGAAATGCCATATCAACAACATACACATACCTCAACGTGTATG

>Elu_Psg16N1 (Ellobius lutescens; Transcaucasian mole vole) WGS LOJG01036454.1

CCTCCATTTTAACCTGCTGGCACCTGTCCACCACTGTCAAAATCACAATTGACTCAATGCCACTTCAAGTGGTTGAAGGAGAAAATTTCCTTCTACGTGTCAACAATCTGCCACAGAATCTTCTAACTTTTGCCTGGTTCAAAGGAGTGACAAATATCAACTCCAGAATTGCACTATATACACTGACCACTAAGCTATGTGTGATGGGGCCCGAAAATAGTGGTAGAGAAGCTGTGTACAGCAATGGATCCCTGTGGCTCAAAAATGTCTCCCAGAAGGACACAGGATTTTATGTACTACAAACAGTAAATAGAGGTGGAAAAATTGTATCTACAACAACCACATACCTCCACGTGTATG

>Elu_Psg19N1 (Ellobius lutescens; Transcaucasian mole vole) WGS LOEQ01009747.1

CCTCCCTTTTAACCTCCTGGCACCTGACTACCACTGCCAATGTGACCGTTGAATTACTGCCAACTCCAGTGGTCGAAGGAGATAACGTCCTTTTCCTTGTCCACAATCTGCCAGAGGAAATTAAAGCCGTAGCCTGGTTCAAAGGGCTGAGAAACAAGAAACAACAAATTGCAGTGTATGTACTGCACCAAAGTTTAAGTATGCCAGGTCCTATGCACAATGGGAGAGAGACAATATATCACAATGGATCTCTGTTTCTTGAAAAGGTCACCCAGAAGGATGCAGGATTCTATACCCTGCGAACCTATGATAGAGGTGGAAAAACTGTATCAACCATACCCATGTACCTCCACGTGCACG

>Elu_Psg20N1 (Ellobius lutescens; Transcaucasian mole vole) WGS LOJG01154727.1

CCTCCCTTTTAACAAGCTGGCACCTGCCCACAGCCGTCCAAGTCAGAATTGAATCTGTCCCACCCCAAGTGGTTGAAGGAGAAAACGTCCTTCTTGTTGTCCACAATCTGCCAGAGAATCCTCAAAATTTAATCTGGTCCAAAGGCATGGAAAGTATGAACCATGAAATTGGAACATATTTACTGAACAAAGGTTTAAGTTTACCAGGGCCTTTTCACAGTGGTAGAGAGACAGTGTACAGCAATGGATCCCTGCTGCTGAGAAATGTCACCAAGAAGGATACAGGATTCTATACCCTACGAATCTTAAATAGACATGTAATTGTTGTGTCAACAACAACCATGTACCTTCATGTGCACA

>Eta_Psg1N1_P_partial (Ellobius talpinus; Northern mole vole) 9.7.21 WGS LOJH01244771.1

CCTTGCTTTTAACCTGTTGGCACCTGCACCCTACTGCCCAAGTCACCATTGAATCAGTGCCGCCCCAAGTGGTTGAAGGAGAAAACATTCTTCTACGTGTTCATAATCTACCAGAGAATCTTCTAGCCTTTGTCTGGCACAAAGTGGTGAGGAATATGAGCCTTGGAATTGCACTATATTCACTGGCCAAGGGTTTAAGTGTGACAGGACCTGTACACAGTGGTAGAGAGACAGTGTGCAGCAATGGATCCCTGAAGATCTACAATGTCACCCAGAAGGACACAGGATTCTACACCTTTCGAACCATAAATGGACAAGTAGGAGTCTCATTATAAnCAACCACGTACCTTCACGTGTATA

>Eta_Psg2N1_P (Ellobius talpinus; Northern mole vole) WGS LOJH01107468.1

CCTACCTTTTAACCTGTTGGCACCTGCCCACCACTGTCCAAGTCATTGTTGATTTAGTGCCACCTCATGTTGTTGAAGGAGAAGATGTCCTTCTTCATGTCCGCAATCTGCCTGAGGATCGTGTAGCCTTTGTCTGGCACAAAGAGGCGACAAAGATGAACCTCGGAATTGTACTTTATTCACTGACCACTAATTTAAACATCACGGGGCCTGGACACAGTGGTAGAGAGATAGTGTACAGAAATAGATCTCTGCACCTCTAAAATCTCACCCAGAAGGACACAGGATTCTACACACTACGATCTTTAAATAGGCATAAAGGAATTGTATCAACAACATCTATATACCTGCATGTATACT

>Eta_Psg3N1 (Ellobius talpinus; Northern mole vole) WGS LOJH01020394.1

CCTCCCTTTTCTCCTTCTGGCATCTCCCCACTACTGCTCATGTGTCCACTGAATCAGTGCCACCCCTAGTGGCTGAAGGAGATAATGTCCTTATCCTTGTCAACAATCTGCCAGAGAATCTTTTAGCCTTAGCCTGGTTCAAAGGGCTAACAAATATGAAACAAGGAATTGCATTATACGCACTGCACAAAAATGTAAGTGCTACAGGGCCTGTGCACAGTGGCAGAGAGACAATATATCACAATGGATCCTTGTTGATTGAAAAACTCACCCAGAAGGACACAGGATTCTACACCTTTCGAGCCTATAACAGACATGGAAGAATTGTATCAACCACATCCACCTACCTCCATGTGCAAG

>Eta_Psg4N1 (Ellobius talpinus; Northern mole vole) 29.5.21 WGS LOJH01128794.1

CCTCACTTTTAGGCTGCTGCCTATCCACCACTGACTATATCACCATTAAATCTGTCCCACCCCATGTGGCCAGTGGAGAAGACGTCTTTCTCCATGTCTACAATCTGCCAGAGGATCTTCTAGCCTTTGCCTGGTTCAAAGGGGCGACAAGCATGAACCATGGAATTGCAGTATATGCACTGAACAAAAATTTAAGTGCGACAGGGCCTGCACATAGTGGTAGAGAGAAAGTGTACCACAATGGATCCCTGCTGCTCCAAAGTGTCACCGAGAAGGACACAGGATTCTATACCCTACGAACCTTAGACAGACACGGAGAGATTGTATCAACAACAACCATGCACCTCTATGTGTATC

>Eta_Psg5N1 (Ellobius talpinus; Northern mole vole) 29.5.21 WGS LOJH01018125.1

TCTCCCTTTTAAGCTCCTGGCATCTGTCCACAAATGCCCATATGACTATTGAAAAAGTGCCAACCCTAGTTGCTGAAGGAGATGACATCCTTTTCCATGTCAATGATCTGCCAGAGAATATTACAACCTTAGCCTGGTTCAAAGGTCTAAGAAATACGACACAAGGAATTGCAGCATATGCACTGCTCTTAAATTTGAGTAGACCAGGTCCTATGTACAGTGGTAGAGAGACAATATATCGCAATGGATCCCTGCTGATAAAAAATGTCAACCCGATGGACACTGGATTCTATACCGTACGAACTTATAATAGGCATGGAACTAGGATATCAATAACATCTGCGTACCTCCAAGTGCATG

>Eta_Psg6N1 (Ellobius talpinus; Northern mole vole) WGS LOJH01033788.1

TCTCCCTTTTAATCTCCTGGCACCTCTCCACTGCTGTCCATATAACTACTGATTCAGTGCCAACCCGAGTGGTTGAAGGAGAAAACGTTCTTTTCCTTGTGCATGATCTGCCAGATAATACTAAATCCTTAGTCTGGTTCAAAGCTCTAAGAAATATGACAGAAGAAATTGCAGCATATGTACTGCCCTACAATTTAAGCAGGCCAGGTCCTCTGTACAGTGGTAGAGAGACAATATATTACAATGGATCCCTGATGATAGAAAATGTCAACCTCAAGGACACAGGATTCTATATCCTACGAACCTATAACAGACGTGAAAAAGTCATATCAACAACAACCATGTACCTCCAGGTGAATG

>Eta_Psg7N1 (Ellobius talpinus; Northern mole vole) WGS LOJH01086927.1

CCTCCCTTTTAACCCTCTGGCACCTGTCCATCACTGCCTCTGTGACCATTGAATCAGTGCCACCCCTGATGGCCGAAGGAGATAACATTCTTTTCCTTGTCGACAATCTGCCGGAGAAGATGGTAACCTTAGTCTGGTTCAAAGGGCTAACAAATATGAAAGCTGTAATTGCAATATATGGACGGCACATCAATTTAAGTGCATCTGGGCCTTTGCACAGTGGTAGAGAGACAATATATTACAATGGATCCCTGCTGATTAAAAAGGTTATCCAGAAAGACACAGGATTCTATACCCTACGAAGCTATGATAAGTATTTAAACATCATATCAACAACATCCACATACATCCATGTTCATG

>Eta_Psg8N1_P (Ellobius talpinus; Northern mole vole) 21.6.21 WGS LOJH01244771.1

CCTTGCTTTTAACCTGTTGGCACCTGCACCCTACTGCCCAAGTCACCATTGAATCAGTGCCGCCCCAAGTGGTTGAAGGAGAAAACATTCTTCTACGTGTTCATAATCTACCAGAGAATCTTCTAGCCTTTGTCTGGCACAAAGTGGTGAGGAATATGAGCCTTGGAATTGCACTATATTCACTGGCCAAGGGTTTAAGTGTGACAGGACCTGTACACAGTGGTAGAGAGACAGTGTGCAGCAATGGATCCCTGAAGATCTACAATGTCACCCAGAAGGACACAGGATTCTACACCTTTCGAACCATAAATGGACAAGTAGGAGTCTCATTATAAnCAACCACGTACCTTCACGTGTATA

>Eta_Psg10N1 (Ellobius talpinus; Northern mole vole) WGS LOJH01039303.1

CCTCCATTTTAAGCTTCTGGCATATGTCTGCTATTGCCCACGAGACCACTGAGTCACTGCCACACCAAGTGGTTGAAGGAGAAAATGTCCTTTTGATTGTCCACAATCTGCCAGAGAATCTTATAGCCTTTTCCTGGTTCAAAGGTCTAACAAATATGATGCAAGAAATTGCAATATATACACTGAACAACAATTTAAGTGCACCAGGGCCTGTGCACAGTAATAGAGAGACAGTGTATAGCAATGGATCCCTGCTGATAGAAAATGTCACCCAGAAAGACACAGGAATCTATACCCTACAAACCTATAATAGAAGTGGAAAAATTGCATCAACAACATCTATGTACCTTCATGTGCACG

>Eta_Psg11N1_P (Ellobius talpinus; Northern mole vole) WGS LOJH01075796.1

CCTCCCTTTTAACTTACCCACCACTGCCCAAGTCATCATTGAATTAGTGCCACCCAACATGTTCCAAGGAGAAAATGTCCTTCTAGAGGTCCACAATCTGCCAGAGGATTTTCTAGCCTTTGCTTGGTACAGGGGTGACAAAAATGAAACGTGGAATTGCAGTCTATGCCAAAAGAAACAGTTTAAGAGCATCGGGGCCTGCGTACAGTGGTAGACAGGCAGTGTACAGTGATGGATCACTGCTGCTCCAGCGTGTCATCCTCAAGGACACAGGATTCTACACCCTATGAGTTATAAGTAGACAAGGAGAAATTGTATCAACAACATCCGTGTTCCTCCATGTGCAGA

>Eta_Psg12N1_P (Ellobius talpinus; Northern mole vole) WGS LOJH01043030.1

CCTCCCTTTTAACCTTTGGCACCTGTCCACCACTGCCCGTGTGATCACTGAATCAGTACCACCCCAAGTGGCTGAAGGAGAAAATGTTCTTTTCATTGTCCACAATCTGCCAGAGAATGTTAAATCCTTTGCCTGGTTCAAAGGGCTAAAAATCGAGAAACAAGGAATTGCAATGTATAGACGGCACAAGAATTTAGTTACAAATGGGCCTATGCACAGTGGCAGAGAGACCATATATCACAATGGATCCCTGCTGCTTGAAAAGGTCTCCCATAATGACACAGGATTCTATATCCTACAAACCTATAATAGACATGCAAAAATCCTATCAACAACTGCTGTGTATCTCCATGTGCATG

>Eta_Psg13N1 (Ellobius talpinus; Northern mole vole) WGS LOJH01034294.1

CCTCCCTTTTAACTGTCTGGCACATGTCCACCACTGCCCAGGTGACCACTGTGTCAGTGCCACCCATAGTGTCTGAAGGAGATGACGTCCTGTTCCTTGTCCATAACCTGCCAGAGGAAATTGAAACCTTAGCCTGGTTCAAAGGGCTAGGAGATGAAGCAGAAGAAATTGCAGCATATGCACCGCACAGCGGTTTAGGTAGGCCAGGTCCTGCGCACAGCAGCAGAGAGACAATGTATCACAACGGATCCATGCTGTTTGAGAAGGTCCACCTGAAGGACTCAGAGTTCTACACCCTACGAGCCTATAATGGAAGTGGAAAAATCATATTAACAGCAAACGTGTACCTCAATGTGTATG

>Eta_Psg14N1 (Ellobius talpinus; Northern mole vole) WGS LOJH01042066.1

CCTCCCTTTTAACCTTCTGGCACCTGTCTACCACCGCCCATGAGACCACTGTATCAGTGCCACCCCGAGTGGCTGAAGGAGATGAAGTCCTGTTCCTTGTCCACAATCTGCCAGAGGAAATTAAATCTTTAGCCTGGTTCAAAGGGCAAGGAAACACAACAGAAAAAATTGCAGCATATGCACTGCACAACAATTTAAGTAGACCAGGTCCTGCGTACAGCAATAGAGAGACAATATATCACAACGGATCCATGCTGTTTGAGAAGGTCATCCTGAAGGACTCAGGATTCTATACGCTACGAACCTATAACAGACGTGGAAAAATTGTATCAACAACATATGTGATCCTCAATGTACATG

>Eta_Psg15N1 (Ellobius talpinus; Northern mole vole) WGS LOJH01111403.1

CCTTCCTTTTAACCTTCTGGCACCTGTCCACCACTGCCTTTGTGACCACTGTATCAGTGCCACCCCGAGTGGCCGAAGGAGATGACGTCCTATTCCTTGTCCACAATCTGCCAGAGAAAATTAAAACCATAGCCTGGTTCAGAGGGCCCTCAAATATGACTGCAATATATGGACTGCCCAACAATTTAAGTAGGCCAGGTCCTGCACACAGTGGTAGAGAGACAATATTTCACAATGGATCCATGCTCCTTGAAAAGGCCAACCTGAAGGACACAGGCTTCTATACCGTACGAACCTACAATAGACATGGAAATGTCATATCAACAACATACACATACCTCAACGTGTATG

>Eta_Psg16N1 (Ellobius talpinus; Northern mole vole) WGS LOJH01043170.1

CCTCCCTTTTAACCTGCTGGCACCTGTCCACCACTGTCAAAATCACAATTGACTCAGTGCCACTCCAAGTGGTGGAAGGAGAAAACGTCCTTCTACGTGTCAACAATCTGCCACAGAATCTTCTAACTTTTGCTTGGTTCAAAGGGGTGACAAATATGAACTTCAGAATTGCACTATATACAATGACCACTAATCTATGTGTGATGGGGCCTGAAAATAGTGGTAGAGAAGCTGTGTACAGCAATGGGTCCCTGTGGCTCAAAAATGTCTCCCAGAAGGACACAGGATTTTATATACTACAAACAGTAAATAGAGGTAGAAAAATTGTATCTACAACAACCACATACCTCCATGTGTACG

>Eta_Psg19N1 (Ellobius talpinus; Northern mole vole) WGS LOJH01204808.1

CCTCCCTTTTAACCTCCTGGCACCTGTCTTCCACTGCCAATGTGACCGTTGAATTACTGCCAACTCCAGTGGCTGAAGGAGATAACGTCCTTTTCCTTGTCCACAATCTGCCAGAGGAAATTAAAGCCGTAGCCTGGTTCAAAGGGCTGAGAAACAAGAAACAACAAATTGCAGTGTATGTACTGCACAAAAATTTAAGTATGCCAGGTCCTATGTACAGCGGGAGAGAGATAATATATCACAATGGATCCCTGCTTCTTGAAAAGGTCACCCAGAAGGATGCAGGATTCTATACCCTACGAACCTATGATAAAGGTGGAAAATTTGTATCAGCCATACCCATGTACCTCCACGTGCACG

>Eta_Psg20N1 (Ellobius talpinus; Northern mole vole) LOJH01053430.1

CCTCCCTTTTAACATGCTGGCACCTGCCCACAGCCGTCCAAGTCAGAATTGAATCTGTCCCACCCCAAGTGGTTGAAGGAGAAAACGTCCTTCTTCTTGTCCACAATCTGCCAGAGAATCCTCTAACTTTAGTCTGGTCCAAAGGCATGGAAAGTATGGACCATGTAATTGGAACATATTTACTGAACAAAGATTTAAGTGTACCAGGGCCTTTTCACAGTGGTAGAGAGACAGTGTACAGCAATGGATCCCTGCTGCTGAGAAATGTCACCAAGAAGGATACAGGATTCTATACCCTACGAATCTTAAATAGACATGTAGTTATTGTGTCAACAACAACCATGTACCTTCATGTGCACA

>Gdo_Ceacam11N1 (Grammomys dolichurus; common thicket rat) WGS JADRCF010450127.1

TCTCCCTTTTAACCTGCTGGCTGCTTCCCACTACTGCCAAGCTCACCATTGAATCAGTGCCTCCCATTGCTGTTCAAGGGGACAATGTTCTTCTGTTAGTGCATAATTTGCCAAAGAAGGTTAAAACCGTTTCATGGTACACAGGAGTTGCAGCGCTCAAGAGTTGTGAAATTGCAAGACATGTGATAGCTACCAATTCTAGTGTGGTGGGACTTGCACACAGTGGTAGAGAGACAGTACTCAACAATGGATCTCTGCTGATCAAAAGTGTCACTAGAAAAGACTCAGGATACTACACCCTACAAATACTTGATTCAGCCTCAAGACCTGAAATAATACATGCAGAATTCTTTGTACACA

>Gdo_Ceacam12N1 (Grammomys dolichurus; common thicket rat) WGS JADRCF010482624.1

TTTCCCTTTTAACCTGCTGGTTGCTTCCCATTACTACTCAGCTCACCATTGAATCAGTGCCTCCCATTGCTGTTGAAGGGGAAAATGTTCTTTTGTTAGTGCATAATTTGCCAAAGAAGGTTAAAGCCCTTTCGTGGTACACAGGAGATAAACTTTTCAAGAGTTGTGAAATTGCAAGACATGTGATAGCTACCAATTCTAGTGTGGTGGGACTTGCACACAGTGGTAGAGAGACTGTATTCAATAATGGATCTCTGCTGATTAAGAGTGTCACCAGAAAAGACTCAGGATACTACACCCTACAAATACTTGATTCAACCTCAAGATCTGAAATAATACATGCAGAATTCTTTGTACATG

>Gdo_Ceacam13N1 (Grammomys dolichurus; common thicket rat) WGS JADRCF010505608.1

TTTCCCTTTTAACCTGCTGGCTGCTTCCCACTACTGCCCGGCTTACCATTGAATCAGTGCCTCCCATTGCTATTGAAGGGGAAAATGTTCTTGTGTTTGTGAAAAACCTGCCAAAGAATGTTAAAGCCCTTTCCTGGTACAGAGGAGATAAACCACTCAAGACTTTTGAAATTGCAAGACATGATATAGCTACCAATTCTAGTGTGGTGGGACCTGCAAACAGTCATAGAGAGACAGTACTCAACAGTGGATCTCTGCTGATCAAGAGTGTAACCAGAAAAGACTCTGGATACTACACCCTACAAATACTTCATACAAGCTCAAGACCTGAAATAATGCGTGCAGAATTCTTTGTACAGA

>Gdo_Ceacam15N (Grammomys dolichurus; common thicket rat) WGS JADRCF010498844.1

CCTCTCTTTTAACCTGCTGGAACTCGCCCACTGCAGCACTGCTAACACACAAAGAGATGCGGTTCTCGGCTGCCGAAGGGGCAAAGGTTCTTCTCTATGTTCCTGACCAGGAAGAGAACCTCCTCTCCTTTTCCTGGTACAAAGGGAAAGATGTAAATGAGAATTTTACAATTGCGCATTATAAAAAATCCAGCGACTCACTTCAACTTGGAAAGGAAGTCAGCGGCAGGGAAGAAATCTATAAGGACGGCTCCATGCTGCTCCACGCCATCACCCAGGAAGACACGGGATTCTACACTTTAAAAACCTTTAAAGCACATAACCAACAGGAAATAACATATGCCCATCTCCAAGTATATA

>Gdo_Psg37N1_P (Grammomys dolichurus; common thicket rat) WGS JADRCF010301968.1

CCCCCCTCTTAACCCGCTAGTTTCTGCCCACCATTGTCTGAGTAAGCATTGCCTCCTTACTGTCCAAAGTGGTTAAAGGAGAAAATGTTCTTCCACATGTTGACAATCTGCCAGAGAACCTTCCAGCATTTGCCTGGTACAAAAAAAGGTGTCAAATATGAGGCTTGTAATTCCACTGCACTCATTGGACTACAGCTTGAGTGTGATGGGGCCAGAGCACAGTGGTAGAGAGACATTGTACTGTAATGGGTCCTGTGGATCCAAAATGTCACAAAGGAGTACACCGGATTCTACAGTTTTCAAACCATAAGTGAACATGGAGAAATTGTATCAAATACATCCATGTTCATTTATATGTATT

>Gdo_Psg38N1 (Grammomys dolichurus; common thicket rat) WGS JADRCF010428531.1

CCTCCCTTTTAACCTGCTGGCTCCTGCCCGCCACTGCCCAGGTCACCATTGAATCGGTGCCACCCAATGCAGTTCAAGGAGAAAATGTCCTTCTTTTTGTCAACAATCTGCCAGAGGACATTATAGCCTTAGCCTGGTACAGAGGGCTGAAGAAAATTGTCATATACATACTGAACACTAACGTAAGTGTGACAGGGTCCATGTACAGCGGTAGAGAGATAACGTCCAGCAACGGGTCCCTGTGGATCCACAATGTCACCCAGAAGGACACAGGAGTCTACACCTTGCGAACTGTAAATAGACGTGGAGAAATTGTATCAACAACATCCATGTATCTCTACGTGCACA

>Gdo_Psg39N1 (Grammomys dolichurus; common thicket rat) WGS JADRCF010470998.1

CCTCCCTTTTAACCTGCTGGCTTCTGCCCACCACTGCCCAAGTCACCATCGAATTTTTACCACTCAAAGTGGTTGAAGGAGAAAATGTTCTTTTACGTGTTGACAATTTGCCAGAAAATCTTCTAGGCTTTGCCTGGTACAGAGGGATAAAAAATTTGAGGCAAGGAATTTCACTATATTCACTGGCCTATGGCACAAATGTGCAAGGTCCTACACACAGCGGTAGAGAGACATTGTACAGCAACGGCTCCCTCTGGATACAAAATGTCACCAAGGAGGACACAGGATATTACACCTTTCGAACCATAAGTAGACGTGGAGAAATTATATCAAATACATCCCTCCAACTTCATGTGTACT

>Gdo_Psg40N1 (Grammomys dolichurus; common thicket rat) WGS JADRCF010502069.1

CCTCCCTTTTAACCTGCTGGCACCTGTCTACCACTTCCCAAGTCACCATTGAATTAGTGCCACCCCAAGTGGTTGAAGGAGAAGATGTCCTATTCCTTGTCCACAAACTGCCAGAAAATCTTGCGGCCTTTGTCTGGTTCAAAGGGTTGAGAGTTGTAAAACATGCGATTGCACTGCATGCAACAGACACTAGAAAAACTGTGATGAGGCACCTGTTCGGTGCTAGAGAGATCTTGTACAGAAATGGGTCCCTGTTGATCCAAAACGTCACCCAGAAGGACGCAGGATTCTACATCCTACAGACCTTAAATAGAAATGGATCTACTGTGTTAACATCACTGTTCCTTCATGTGAACT

>Gdo_Psg42N1 (Grammomys dolichurus; common thicket rat) WGS JADRCF010396355.1

CCTCCCTTTTAACCTGCTGGCACCTGTCTACCACGTCTGAAGTCACCATTGAATTAGTGCCACCACAAGTGGTCAAAGGGGAAGATGTTCTTTTCCTTGTCCATAATCTGCCAGAGAATCTTACAGCCTTTGCTTGGTTTAAAGGCAGGACAAATATGAAACGAGGAATTGCACTGTATGCATTGGCCTCTGACATACATATACACAGTGATAGAGAGACACTGTATAGCAATGGATCCCTGATAATACACAATGTCACCCGGAAGGACAGAGATTATTATACTCTACGAACCTTCAATAGACATGCAAAAACTGTATCATCAACAACCACATTTCTCCATGTGAACC

>Gdo_Psg43N1 (Grammomys dolichurus; common thicket rat) WGS JADRCF010343058.1

CTTTTCTTTTAATCTCCTGGTTCCTGCCCACCACTGTCCAGGTCACCATTGAATTAGTGCCACCCCAAGTGACTGAAAGAGAAAATGTCCTTCTTTTTGTCTACAATCTGCCAGAGAATCTTATAGGCATAGCCTGGTTCAAAGGAGTGACAAATATGAGCCTTGGAATTGCAATGTATGCACTGGCCTTTAACATAAGTTTTTTAGGGCCTGACCACAGTGGTAGAGAGACAGTGTACAGAAATGGATCCTTGCTGTTCCACAATGTCACCAAGAAGGACACAGGATTTTATACTCTACGAACCTTAAATATACATAAAAAAATTGTATCAACAACATCCATATATCTGCATGTGTACA

>Gdo_Psg44N1 (Grammomys dolichurus; common thicket rat) WGS JADRCF010142819.1

CCTCCCTTTTAACCTGCTGGTACCTGCCTACCACTGCAGAAATAACCGCTGAATTAGTGCCACCTCAAGTGATTGAAGGAGAAAATGTTCTCATACGTATTGACAATCTACCAGAGAATCTTGTAACCTTAGTCTGGTTAAGAGGAGCGGGGATTAAGAGCCCTCGAATTGGACAATATACACTGCCCACTAATGCTACTGTGCTGGGGCATGGTCACAGTGGAAGAGAGACTTTGTACAGCAATGGATCCCTGCAGATCTACAATGTCACCCAGGAGGATATAGGGTTCTACAGCCTACAAATCATAAATAGATATGCAGAAATTGTATCAATAATATCCATATATCTCAATGTGTACT

>Gsu_Ceacam9N (Grammomys surdaster; African thicket rat sp.) WGS SRMG01000223.1

CCTCCCTCTTAACCTGCTGGCTCCTGCCCACCACTGCCCAAGTCACCATCGAATCCTTACCGCCCCAAGTGGTTGAAGGAGAAAATGTTCTTCTACGTGTTGACAATCTGCCAGAGAATCTTCTAGCCTTTGTCTGGTACAGAGGGGTGACAAATATGAGTCTCGGAATTGCACTGTATTCACTGAAGTACAGTGTAATTGTGACGGGGCCCGTGCACAGTGGTAGAGAGACATTGTACAGCAACGGGTCCCTGTGGATCCACAATGTCACCCAGAAGGACACAGGATTCTACACCCTACGAACCATAAGTAAACGTGGAGAAATTGTATCAAACACAACAATACACCTTAAAGTGTACT

>Gsu_Ceacam11N1 (Grammomys surdaster; African thicket rat sp.) WGS SRMG01000958.1

TCTCCCTTTTAACCTGCTGGCTGCTTCCCACTACTGCCAAGCTCACCATTGAATCAGTGCCTCCCATTGCTGTTCAAGGGGACAATGTTCTTCTGTTAGTGCATAATTTGCCAAAGAAGGTTAAAACCGTTTCATGGTACACAGGAGTTGCAGCGCTCAAGAGTTGTGAAATTGCAAGACATGTGATAGCTACCAATTCTAGTGTGGTGGGACTTGCACACAGTGGTAGAGAGACAGTACTCAACAATGGATCTCTGCTGATCAAAAGTGTCACTAGAAAAGACTCAGGATACTACACCCTACAAATACTTGATTCAGCCTCAAGACCTGAAATAATACATGCAGAATTCTTTGTACACA

>Gsu_Ceacam12N1 (Grammomys surdaster; African thicket rat sp.) WGS SRMG01000958.1

TTTCCCTTTTATCCTGCTGGCTGCTTCCCATTACTACTCAGCTCACCATTGAATCAGTGCCTCCCATTGCTGTTGAAGGGGAAAATGTTCTTTTGTTAGTGCATAATTTGCCAAAGAAGGTTAAAGCCCTTTCGTGGTACACAGGAGATAAACTTTTCAAGAGTTGTGAAATTGCAAGACATGTGATAGCTACCAATTCTAGTGTGGTGGGACTTGCACACAGTGGTAGAGAGACTGTATTCAATAATGGATCTCTGCTGATTAAGAGTGTCACCAGAAAAGACTCAGGATACTACACCCTACAAATACTTGATTCAACCTCAAGATCTGAAATAATACATGCAGAATTCTTTGTACATG

>Gsu_Ceacam13N1 (Grammomys surdaster; African thicket rat sp.) WGS SRMG01000958.1

TTTCCCTTTTAACCTGCTGGCTACTTCCCACTACTGCCCGGCTTACCATTGAATCAGTGCCTCCCATTGCTATTGAAGGGGAAAATGTTCTTGTGTTTGTGAAAAACCTGCCAAAGAATGTTAAAGCCCTTTCCTGGTACAGAGGAGATAAACCACTCAAGTCTTTTGAAATTGCAAGACATGATATAGCTACCAATTCTAGTGTGGTGGGACCTGCAAACAGTCATAGAGAGACAGTACTCAACAGTGGATCTCTGCTGATCAAGAGTGTAACCAGAAAAGACTCTGGATACTACACCCTACAAATACTTCATACAAGCTCAAGACCTGAAATAATGCGTGCAGAATTCTTTGTACAGA

>Gsu_Ceacam15N (Grammomys surdaster; African woodland thicket rat) WGS SRMG01000223.1

CCTCACTTTTAACCTGCTGGAACTCGCCCACTGCAGCACTGCTAACACACAAAGAGATGCGGTTCTCGGCTGCCGAAGGGGCAAAGGTTCTTCTCTATGTTCCTGACCAGGAAGAGAACCTCCTCTCTTTTTCCTGGTACAAAGGGAAAGATGTAAATGAGAATTTTACAATTGCGCATTATAAAAAATCCAGCGACTCACTTCAACTTGGAAAGGAAGTCAGCGGCAGGGAAGAAATCTATAAGGACGGCTCCATGCTGCTCCACGCCATCACCCAGGAAGACACGGGATTCTACACTTTAAAAACCTTTAAAGCACATAACCAACAGGAAATAACATATGCCCATCTCCAAGTATATA

>Gsu_Psg36N1 (Grammomys surdaster; African thicket rat sp.) WGS SRMG01000223.1

CCTCCCTCTTAACCTGCTGGCTCCTGCCCACCACTGCCCAAGTCACCATCGAATCCTTACCGCCCCAAGTGGTTGAAGGAGAAAATGTTCTTCTACGTGTTGACAATCTGCCAGAGAATCTTCTAGCCTTTGTCTGGTACAGAGGGGTGACAAATATGAGTCTCGGAATTGCACTGTATTCACTGAAGTACAGTGTAATTGTGACGGGGCCCGTGCACAGTGGTAGAGAGACATTGTACAGCAACGGGTCCCTGTGGATCCACAATGTCACCCAGAAGGACACAGGATTCTACACCCTACGAACCATAAGTAAACGTGGAGAAATTGTATCAAACACAACAATACACCTTAAAGTGTACT

>Gsu_Psg37N1 (Grammomys surdaster; African thicket rat sp.) WGS SRMG01000958.1

CCCCCCTCTTAACCCGATAGTTTCTGCCCACCATTGTCTGAGTAACCATTGCCTCCTTACTGTCCAAAGTGGTTAAAGGAGAAAATGTTCTTCCACATGTTGACAATCTGCCAGAGAACCTTCCAGCATTTGCCTGGTACAAAAAAAGGTGTCAAATATGAGGCTTGTAATTCCACTGCACTCATTGGACTACAGCTTGAGTGTGATGGGGCCAGAGCACAGTGGTAGAGAGACATTGTAATGGGTCCTGTGGATCCAAAATGTCACAAAGGAGTACACTGGATTCTACAGTTTTCAAACCATAAGTGAACATGGAGAAATTGTATCAAATACATCCATGTTCATTTATATGTACT

>Gsu_Psg38N1 (Grammomys surdaster; African thicket rat sp.) 9.10.20 WGS SRMG01000223.1

CCTCCCTTTTAACCTGCTGGCTCCTGCCCGCCACTGCCCAGGTCACCATTGAATCGGTGCCACCCAATGCAGTTCAAGGAGAAAATGTCCTTCTTTTTGTCAACAATCTGCCAGAGGATATTATAGCCTTAGCCTGGTACAGAGGGCTGAAGAAAATTGTCATATACATACTGAACACTAACGTAAGTGTGACAGGGTCCATGTACAGCGGTAGAGAGATAACGTCCAGCAACGGGTCCCTGTGGATCCACAATGTCACCCAGAAGGACACAGGAGTCTACACCTTGCGAACTGTAAATAGACGTGGAGAAATTGTATCAACAACATCCATGTATCTCTACGTGCACA

>Gsu_Psg39N1 (Grammomys surdaster; African thicket rat sp.) WGS SRMG01000958.1

CCTCCCTTTTAACCTGCTGGCTTCTGCCCACCACTGCCCAAGTCACCATCGAATCTTTACCGCTCAAAGTGGTTGAAGGAGAAAATGTTCTTTTACGTGTTGACAATTTGCCAGAAAATCTTCTAGGCTTTGCCTGGTACAGAGGGATAAAAAATTTGAAGCAAGGAATTTCACTATATTCACTGGCCTATGGCACAAATGTGCAAGGTCCTACACACAGCGGTAGAGAGACATTGTACAGCAACGGCTCCCTCTGGATACAAAATGTCACCAAGGAGGACACAGGATATTACACCTTTCGAACCATAAGTAGACGTGGAGAAATTATATCAAATACATCCCTCCAACTTCATGTGTACT

>Gsu_Psg40N1_partial (Grammomys surdaster; African thicket rat sp.) WGS SRMG01000223.1

CCTCCCTTTTAACCTGCTGGCACCTGTCTACCACTTCCCAAGTCACCATTGAATTAGTGCCACCCCAAGTGGTTGAAGGAGAAGATGTCCTATTCCTTGTCCACAAACTGCCAGAAAATCTTTCGGTCTTAGTCTGGTTCAAAGGGTTGACAGTTGCAAAACATGCAATTGCACTGTATGCAACAGACATAAGAGAAATTGTGAAGGGGCTCATGTACAGTGATAGAAAGACCTTGTACAGAAACGGGTCCCTGTTGATCCACAATGTCACCCAGAAGGACACAGGATACTACACCCTACAAACCTTAAATAGAAATGGAACTATTGTGTCAAAnnnnnnnnnnnnnnnnnnnnnnnnnn

>Gsu_Psg42N1 (Grammomys surdaster; African thicket rat sp.) WGS SRMG01000223.1

CCTCCCTTTTAACCTGCTGGCACCTGTCTACCACGTCTGAAGTCACCATTGAATTAGTGCCACCACAAGTGGTCAAAGGGGAAGATGTTCTTTTCCTTGTCCATAATCTGCCAGAGAATCTTACAGCCTTTGCTTGGTTTAAAGGCAGGACAAATATGAAACGAGGAATTGCACTGTATGCATTGGCCTCTGACATACATATACACAGTGATAGAGAGACACTGTATAGCAATGGATCCCTGATAATACACAATGTCACCCGGAAGGACAGAGATTATTATACTCTACGAACCTTCAATAGACATGCAAAAACTGTATCATCAACAACCACATTTCTCCATGTGAACC

>Gsu_Psg43N1 (Grammomys surdaster; African thicket rat sp.) 15.9.20 WGS SRMG01000958.1

CTTTTCTTTTAGCCTCCTGGTTCCTGCCCACCACTGTCCAGGTCACCATTGAATTAGTGCCACCCCAAGTGGCTGAAGGAGAAAATGTCCTTCTTCTTGTTTACAATCTGCCAGAGAATCTTATAGGCATAGCCTGGTTCAAAGGAGTGACAAATATGAACCTTGGAATTGCAATGTATGCACTGACCTTTAACATAAGTTTTTTAGGGCCTGAACACAGTGGTAGAGAGACAGTGTACAGAAATGGATCCTTTCTGCTCCGCAATGTCACCAAGAAGGACACAGGATTTTATACTCTACGAACCTTAAATATACATAAAAAAATTGTATCAACAACATCCATATATCTCCATGTGTACA

>Gsu_Psg44N1 (Grammomys surdaster; African thicket rat sp.) WGS SRMG01000958.1

CCTCCCTTTTAACCTGCTGGTACCTGCCTACCACTGCAGAAATAACCGCTGAATTAGTGCCACCTCAAGTGATTGAAGGAGAAAATGTTCTCATACGTATTGACAATCTACCAGAGAATCTTGTAACTTTAGTCTGGTTAAGAGGAGCGGGGATTAAGAGCCCTCGAATTGGACAATATACACTGCCCACTAATGCTACTGTGCTGGGGCATGGTCACAGTGGAAGAGAGACTTTGTACAGCAATGGATCCCTGCAGATCTACAATGTCACCCAGGAGGATATAGGGTTCTACAGCCTACAAATCATAAATAGATATGCAGACATTGTATCAATAATATCCATATATCTCAATGTGTACT

>Hal_Ceacam11N1 (Hylomyscus alleni; Allen's wood mouse) WGS JADRCC010341433.1

TCTCCCTTTTAACCTGCTGGCTGCTTCAGATTACTGCAGAGCTCACCATTGAATCAGTGCCTCCCATTGCTGTTGAAGGGGAAAATGTTCTTCTGTTTGTGCATAACCTGCCAAAGAATGTTAAAACCCTTTCCTGGTACACAGGAGGTAAACCACTCAAGAGTTGTGAAATTGCAAGTCATGTGATAGCTACGAATTCTAGTGTGGTGGGATTTGCGCACAGTGGTAGAGAGACAGTACTCAACAATGGATCTCTGCTGATTAAGAGTGTTACCAGAAAAGACTCAGGATACTATACCCTAAAAACACTTGATACAACCTCGAGACCTGAAATAATTCATGCAGAATTCTTTGTTCAGA

>Hal_Ceacam13aN1 (Hylomyscus alleni; Allen's wood mouse) WGS JADRCC010373705.1

TCTCTCTTTTAACCTGCTGGCTGCTTCCCACTACTTCCCAGCTCACCATAAAATCAGTGCCTCCAATTGCTATTGAAGGGGAAAATGTTCTTCTGTTTGTGCATAACCTGCCGAAGAATGTTAAAGCCTTTTCCTGGTACACAGGAACTGCACCATTCAAGTGTTGTGAAATTGCAAGCCATGTGATAGCAACCAATTTTACTGTGGTAGGACTTGCACACAGTGGTAGAGAGACAGTACTCAACAATGGATCTCTGCTGATCAAAAGTGTCACCAGAAAAGACTCAGGATACTACACTCTGCGAACACTTGATTCAACCTCAAGACCTGAAATAATACATACAGAATTCTTCGTACACA

>Hal_Ceacam13bN1 (Hylomyscus alleni; Allen's wood mouse) WGS JADRCC010578809.1

TCTCCCTTTTAACCTGCTGGCTGCTTCCCACTACTTCCCAGCTCACCATAAAATCAGTGCCTCCAATTGCTATTGAAGGGGAAAATGTCCTTCTGTTTGTGCATAACCTGCCGAAGAATGTTAAAGCCTTTTCCTGGTACACAGGACCTGCACCATTCAAGTGTTGTGAAATTGCAAGCCATGTGATAGCAACCAATTTTACTGTGGTAGGACTTGCACACAGTGGTAGAGAGACAGTACTCAACAATGGATCTCTGCTGATCAAAAGTGTCACCAGAAAAGACTCAGGATACTACACTCTACGAACACTTGATTCAACCTCAAGACCTGAAATAATACATACAGAATTCTTTGTACACA

>Hal_Ceacam14N1 (Hylomyscus alleni; Allen's wood mouse) WGS JADRCC010552834.1

TCTCCCTTTTAATCTGCTGGCTGCTTCCCACTACTTCCCAGCTCACCATTAAATCAGTGCCTCCCATTGCTGTTGAAGGGGAAAATGTTCTTCTGTTTGTGCATAACCTGCCGAAGAATGTTAAAGCCTTTTCCTGGTACACAGGAGTTACAGCTCTCAAGAGTTGTGAAATTGCAAGTCATGTGATTGCTACCAAATTTACTGTGGTGGGACTTGCACACAGTGGTAGAGAGACACTATTCAACAATGGATCTTTGCTGATCAAGAGTGTCACCAGAAGAGACTCAGGATACTACACTCTACAAATACTTGATGCAACCTCAAGACCTAAAATAATACGTGCAGAATTCTATGTGCACA

>Hal_Ceacam15N (Hylomyscus alleni; Allen's wood mouse) WGS JADRCC010200502.1

CCTCGCTTTTATCCTGCTGGAGCTGGCCCACGGCCGCACTGCTAACATCTAAAGAGATGCGCTTCTCAGCTGCCGAAGGGGCGAAGGTTCTTCTCTCTGTCCCTGACCAGGAAGGGGACCTCCTCTCCTTTTCCTGGTACAAAGGGAAGGATGTAAATAAAAATTTTACAATTGCCCATTACAAGAAGTCCAGCGATTCACTTCAACTTGGAAAGAGCGTCAGTGGCAGGGAAGAAATCTACAAGGATAGCTCCATGATGCTCCAGGCCGTCACGCAGGAAGACACGGGATTTTACACTTTAGAAACCTTTAAGGCACACGATCAACAGGAAATAACATATGTCCATCTCCAAGTATACA

>Hal_Psg36N1 (Hylomyscus alleni; Allen's wood mouse) WGS JADRCC010485818.1

CCTCCCTCTTAACGTGCTGGCTCCTGTCCACCACTGCCCAAATTGACATCGAATCCTTACCACCCCAAGTGGTTGAAGGAGAAAATGTTCTTCTATGTGTTGACAATCTGCCAGAGGATATTATAGCCTTTGCCTGGTACAAAGGGGTGACAAACATGAGCCTCGGAATTGCACTGTATTCACTGACCTACAGCGTAAGTGTGACGGGGCCTGTGCACAGTGGTAGAGAGACACTGTACCTCAATGGATCCCTGTGGATCCAAAATGTCACCCAGGAGGACACAGGATTCTACACCCTACGAACCATAAGTAAACGTGGAGAAATTGTATCAAATACGACCATGCACCTTCACGTGTACT

>Hal_Psg37N1 (Hylomyscus alleni; Allen's wood mouse) WGS JADRCC010466963.1

CCTCCCTCTTAACCTGCTGGCTCCTGCCCAACACTGCCAGTGTCACCATCGAATCCTTACCGCCCAAAGTGGTTGAAGGAGAAAATGTTCTTCTACACGTTGACAATCTGCCAAAGAATCTTCTAGTCTTTGCCTGGTACAGAGAAGTGACAAATTTGAAGCTTGGAATTGCACTGTATTCACTGGATTACAGCACAAGTGTGACGGGGCCTGAGCACAGTGGTAGAGAGACATTGTACAGCAACGGGTCCCTGTGGATCCAAAATGTCACCCAGGAGGACACAGGATATTACACTCTTCGAACCATAAGTAAACGTGGAGAAATGGTATCAAATACATCCGTGTTCCTTCAGGTGTACT

>Hal_Psg37L1N1 (Hylomyscus alleni; Allen's wood mouse) WGS JADRCC010523855.1

CCTCCCTCTTAACTTGCTGGCTCGTGCCCACCACTGCCAGTGTCACCATCGAATCCTTACCGCCCAAAGTGGTTGAAGGAGAAAATGTTCTTCTGCGTGTTGACAATCTGCCAGAGAATCTTCTAGCCTTGACCTGGTACAAAGGGATGACAGATATGAGACTCAGAATTGCACTGTATTCACTGGCCTATAGCATAAGTGTGAAGGGGCTGAAGTACACCGGTAGAGAGATAGTGTACAGCAACGGGTCCCTGTGGATCCGAAATGTCACCCAGGAGGACACAGGATTGTACATTCTTCGAACTATAAGTAAAAATGGAGAATTTGCGTCAAATACATCCCTGTACCTTCAAGTGTACT

>Hal_Psg37L2N1 (Hylomyscus alleni; Allen's wood mouse) WGS JADRCC010439334.1

CCTCCCTCTTAACCTGCTGGCTCCTGCCCACCACTGCCAGAGTCACCATTGAATCCTTACCGCCACAAGTGATTGAAGGAGAAAATGTTCTTCTACGTGTTGAAAATCTGCCAGAGAATCTTCTAGTCTTTGCCTGGTATAGAGGGGTGACAAATTTGAGGCTGTCAATTGCATTCAATTCACTGTACTATAGAGCAAGTGTGAAGGGGCCGAAGCACAGCGGCAGAGAGACATTGTACAGCAACGGGTCCCTATGGATCCAAAATGTCACCCTGAAAGACTCAGGATATTACACTTTTCGAACCATAAGTAAACATGGAGAATTCATATCAAATACATCCCTGTACCTTCATGTACACT

>Hal_Psg39N1 (Hylomyscus alleni; Allen's wood mouse) WGS JADRCC010579797.1

CCTCCCTCTTATCCTGCTGGCTCCTGCCCACCACTGCCAGAGTCACCTTGGAATCCTTACCGCCCAAAGTGGTTGAAGGAGAAAATGCTCTTCTACTTGTTGATGGTCTGCCACAGAATCTTATAGCCTTTGCCTGGTACAAAGGGGTGATAGACATGAGCCTGGGAATTGCACTGTATTCACTGACCTACAGAGTAAGTGTGACGGGGCCTGTGCACAGTGGTAGAGAGATAGTGTACAGCAACGGGTCCCTGTGGATCCAAAATGTCACCAAGGAGGACACAGGATTCTACACTCTTCGAACCATAAGTAAACGTGGAGAAATGGTATTAAATGTATCCACGTACCTTGAGGTGTACT

>Hal_Psg40N1_P (Hylomyscus alleni; Allen's wood mouse) WGS JADRCC010018210.1

CCTCCCTTTTAACCTACTGGTGCTTTTCTACCACGTCCCAAGTCACCATTGAATTAGTGCCACCCCAAGTGGTTGAAGGAGAAGATGTCCTATTCCTTCTCCACAACCTGCCAGAAATTCTTATTGTCCTTAGGCTGGTTCAAAGGGATGACAGTTATAAAACATGGAATTGCACTGTAGGCCACAAACACTAAAGTATGACAGGGCCCATGCACAGTGCTAGAGAGACCTTGTACAGAAATGGGTCCCTGTTGATCCACAATCACCCGAAAGGACACAGGATTCTATACCCTATGAACCTTAAATAGACTTGGAGACATTGTGTCGACATCCACGTTCCTCTAGATGAAGC

>Hal_Psg41N1 (Hylomyscus alleni; Allen's wood mouse) WGS JADRCC010154545.1

CTTCCTTTTTAACCTGCTTGCTCCTGGCCACCACCGCCCAGGTCACCATTGAGTCAGTGCCACCCCAAGTGGTTGAAGGAGAAAATGTTCTTTTTCTTGTACACAAGTTGCCAGAGAATCTTATAGCCTTAGTCTGGTTAAGAAGACTGAGGAAAATGAACCTCGCAATTGGCCTATATGCAATGAACACTAAAACAAGTGTGATGGGGCCCATGCACAGCGGTAGAGAGACAGTATCCAGCAACGGGTCCCTGTGGATCCGCAATGTCACCCAGAAGGACACAGGATTCTACATCCTACAAACCGTAAACAGACGTGGAGAAATTGTGTCGAGAACACCCATGTATCTCTATGTGCACC

>Hal_Psg42N1 (Hylomyscus alleni; Allen's wood mouse) WGS JADRCC010421346.1

CCTCCCTTTTAACATGCTGGCACCTATCTACCACTTCCAAAGTCACCATTGAATTATTGCCACCACATGTGGTTGAAGGGGAAGATGTTCTTTTCCTTGTCCAAAATCTGCCAGAGGATCTTGCAGTCTTTGCCTGGTTTAAAGGGAGGACAAATAAGAAACGTGGAATTGCACTGTATGCAGTGGCCCCTGATATACATGTACACAGCGAAAAAGAGACATTGTACAGCAATGGATCCCTCATGATCCACAATATCACCCAGAAGGACAGAGGTTACTACACCCTACGAACCTTCAATAAACATGCAGAAACTGTATCAACAACATCCATATTCCTTCATGTGAACC

>Hal_Psg43N1 (Hylomyscus alleni; Allen's wood mouse) WGS JADRCC010302962.1

CTTTTCTTTTAACCTCCTGGTTCCTGCCCAACACTGTCCAAGTCACCATTGAATTAGTGCCACCCCAAGTTGCTGAAGGAGAAAATGTCCTTCTTCTTGTTTACAATCTGCCAGAGAATCTTATAGCCATAGCTTGGTTCAAAGGAGTGATAAATATGAACCTCGGAATTGTGTTGTATGCACTGGCCTCTAACATTAGTGTGACCGGGCCTGAACACAGCAGTAGAGAGACAATGTACAGCAATGGATCCCTGCTGCTTCACAATGTCACCCAGAAGGACACAGGATTCTATACTCTGCGGACCTTAAATAGACATGGAAAAATTGTATCAACAACATCCATATATCTCCATGTGTACA

>Hal_Psg44N1 (Hylomyscus alleni; Allen's wood mouse) WGS JADRCC010387900.1

CCTCCTTTTTAGCCTGCTGGCATCTGTCTACCACTGCCCCAATAACCATCGAATTAGTGCCACCCCAAGTGATTGAAGGAGAAAATGTTCTCATACGTATCAACAATCTGCCAGACAATCTTCTAACCTTAGCCTGGTTCAGAGGAATGAGGATTAGGAGCCCTCAAATTGGACAATATACACTGACCACTAATATTACTGTGGTGGGGCCTGCTCACAGTGGTAGAGAGACTTTGTACAGCAATGGATCCCTGCAGATCTACAATGTCACCCAGGAGGATATAGGATTCTACAGCCTACGGATCATAAACAGACACGCAGAAATTGTGTCAATAATGTCCATATACCTCAACGTGTACT

>Hal_Psg45N1 (Hylomyscus alleni; Allen's wood mouse) 20.5.22 WGS JADRCC010272374.1

CATCCTTCTTAACCTGCTGGCACCTGCCTACCACTGCCCAAATAACCATTGAATTAGTGCCATCCCAAGTGATTGAAGGAGAAAATGTTCTCATATGTACCAAAAATCTGCCAGACAACTTTATAACCTTAGCCTGGTTCAGAGGAAAGAAGATTGAGAGCCCTCAAATTGGACAATATACACTGGCCACTAATGTTACTGTGCTGGGGCCTGCTCACAGTGGTAGAGAGACTTTGTACAGCAATGGATCCCTGCAGATCTACAATGTCACCCAGGAGGATATTGGATTCTTCAGTCTACGAATCATAAACAGACACGCAGAAATTGTGTCAATAACATCCATATACCTCAACGTGTACT

>Hal_Psg46N1 (Hylomyscus alleni; Allen's wood mouse) 20.5.22 WGS JADRCC010508713.1

CTTCCCTTTTAACATGCTGGCACCTGTCCACCACAGCCAAGATCACCATTGAATTATTGCCACCCCATGTGGTTGAAGGAGAAGATGTCCTTTTTCTTGTCCACAGTCTGCCAGAGAATCTTACAGCCTTTGCCTGGTTTAAAGGGAGGACAAATAAGAAACGTGGGATTGCACTATATGCAGTGGCCTCTGACATACGTATACACAGTGATAGAGAGACATTGTACAGCAATGGATCCCTGATTATCCACAATATCACCCAGAAGGACAGAGGTTACTACACCCTACGAACCTTCAATAAACATGCAGAAACTGTATCAACAACATCCACATTCCTTCATGTGAACC

>Lim_Ceacam11N1 (Lophiomys imhausi; crested rat) WGS CAJQZJ010026257.1

CCTCCCTTTTAACCTGCTGGCTGCCTCCTACCACTGCCCAGCTCACCATTGAATCAGTGCCGCCTATTGCCGAAGAAGGGGAAAGTGTTGTTCTGTTTGTGCATAACCTGCCAGGGAACGTTCAAGCCATTTCCTGGTACACAGGAGTTATGGTGCTCAAGAGTCGCGAAATTGTGAGATATGTGGTAGCTACCAATTCCTACAGGCTGGGGCCTGCGTCCACCGGTAGAGAGTCAGTACTCAACAACGGATCTCTATTGATCAAGAATGTCACCAGGAAAGACTCGGGATACTACATACTACAAACACTTACCACAGATTTGCGATCTGAAATAATACGTGTGGAATTCTTTGTACACA

>Lim_Psg1N1 (Lophiomys imhausi; crested rat) WGS CAJQZJ010026257.1

CCTCCCTTATAACCTGCTGGCACCTGTCCACCACTGATGAAGTCACCATTGAATTAGTGCCACCCCAAGTAGCTGAAGGAGAAAATATCCTTTTCCTTGTCCACAATCTGCCAGAGAATCTTATAGCCTTAACCTGGCTCAAAGACATGACAAATGTGAGCCTCAGAATTGCACTACATGCACTGGCTACTAAGGCAAGTGTGATGGGGCCTGCATACAGTGGTAGAGAGACAGTGTACAGTAATGGGTCTCTGCGTATCCACAATGTCACCAAGAAGGACATAGGATTCTACACCTTACGAACTTTTAATAGAAAAGCAGAAACTGTATCAATAACATCCACGTACCTCCACGTGAACA

>Lim_Psg2N1 (Lophiomys imhausi; crested rat) WGS CAJQZJ010026257.1

CCTCCCTTTTAACCTGCTGGCACCTGTCCACCACTGATGACGTCACCATCGAATTAGTGCCACCCCAAGTGGTCGAAGGAGAAAATCTCCTTTTCCTTGTCCACAATCTGCCAGAGAATCTTATAGCCTTAGCCTGGTTCAAAGGGGTGAAGAGCACGAACTTTGGAATTGCACTATATGCACTGAACGCTAAAGCAAGCGTGATGGGGCCTGTACACAGTGATAGAGAGACAGTGTACAGCAATGGATCTCTGTGGATACAGAATGTCACCCAGAAGGACATAGGATACTACACCCTACGAACCTTTAATAGACAAGCAGAAACTGTATCAACAACATCTGTATACCTCCATGTGTACT

>Lim_Psg3N1 (Lophiomys imhausi; crested rat) WGS CAJQZJ010026257.1

CCTTCCTTTTAGCCTGCTGGCACCTGCCTACCACTGCCAAAATCACCATTGAATCAGTGCCGCCTCAAGTGCTGGAAGGAGAAAGCGTCCTTCTAAGTGTGGGCAATCTACCAGAGAATCTTATAGCTTTTGTGTGGTTCAAAGGAATGAGGCAAAAGAGCCCAGGAATTGCACTACATGCACTGGCAACTAAAGCAAGTGTGCTGGGGCCTGCGTACAGTGGCAGAGAGACAGTGTACAGCAATGGGTCCCTGTTGCTACAGAATGTCACCCAGGAGGACACAGGGTTCTACAGTCTACGAACCATAAATAGACATGCTGAAATTGTATCAATAACATCGATGTATATCCATGTGTACA

>Lim_Psg4N1_P (Lophiomys imhausi; crested rat) WGS CAJQZJ010026257.1

CCTCTATTATAACCTGTTGGTACTCCCCCCCACCTCTGCCCAAATCACCATCGAGCTGGTGCCACCCCACGTGGTTGATGGAGAAGATGCCCTTCTCCTTATCCAGAATCTACCAGAGGATCTTATTGCTTTAGCCTGGTTCAAAGGGGTGAGGGATATGAGCTTTGGAATTGCACTATATGCACTGACCACTAATATAAGTGTGACAGGGCCTGCACACAGCAGTAGAGAGACAATGTACAGCAATGGGTCCTTGTGAGTACACAATGTCACCCAGAAAGACAGAGGATTTTATACCCTACGAACCATAAATAGACATGGAGAAATTGTATCAACAACCATGTACCTCTACCTGTACAGTAAGTGATTCTTACTTGTGAACTCCGGGTCCTGGGTGGGGGTCCATTCCACTGGACACA

>Lim_Psg5N1 (Lophiomys imhausi; crested rat) WGS CAJQZJ010028334.1

CATCCCTTTTAACCTGCTGGTGCCTGTCCACCACTGATGACGTCACCATTGAATTAGTGCCACCCCAAGTGGTCGAAGGAGAAAACATCCTTTTCCTTGTCCACAATCTGCCAGAGAATCTTATAGCCTTAGGCTGGTTCAAAGGTGTGAGGGACACAAGCCTTGGAATTGCACTATATGCACTGAACGCTAAAGCAAGTGTGATGGGGCCTGTACACAGTGATAGAGAGACAGTGTACAGCAACGGATCTCTGCAAATCCACAATGTCACCCAGAAGGACATAGGATACTACACCCTACGAACCTTTAATAGACAAGCAGAAACTGTATCAATAACATCCACATACCTCCACGTGAACA

>Mag_Ceacam9N (Microtus agrestis; short-tailed field vole) WGS CADCXT010002153.1

CCTTCCTCTTAACCTGCTGGAATGTACCCACCACTGCCGAACTCACTATTGAATTAGTGCCCCCCATGGTTGCTGAAGGTGGAAACTCCGTCCTATTTGTGCATAAAATGCCGCTGAACGTCCAGGCATTTTACTGGTACAAACAGAAAGATGCGACCAAGAGCTATGAAGTTGCACGCTACTTAACACCCGATAACACAACGTCGAAGATGCCTCAACACAGTGGTAGGAAAACGGTATTCTACAGTGGATCCCTGCTGATCAGAAACGTCACCCAGGCTGACAGCGGATTCTACACCTTACTGACGTTCAACACAGAAATGCAAAGTGAACTCACACACATACATCTGGAAGTATACA

>Mag_Psg1N1 (Microtus agrestis; short-tailed field vole) WGS CADCXT010001870.1

CCTTCCTTTTAACCTGTTGGCACCTGCCTACCACTGCCCAAGTCACCATCGAATTAGTGCCGCCCCAAGTGGTTGCAGGAGAAAACGTTCTTCTACGTGTTCATAATCTACCAGAGAATCTTCTAGCCTTTGTCTGGCACAAAGGGGTGAGGAATATGAGCCTTGGAATTGCACTACATTCATTGGACAAGGGTTTAAGTGTGACAGGGCCCATACACAGTGGTAGAGAGACGGTGTACAGCAATGGATCCCTGCAGATCTACAATGTCACCCAGAAGGACACAGGATTCTACACCTTTCGAACCATAAATGGACAAGTAGGAGTTGCATCAATAACAACCACGTACCTTCACGTGTACA

>Mag_Psg2N1 (Microtus agrestis; short-tailed field vole) WGS CADCXT010002299.1

CCTACCTTTTAACCTGTTGGCACCTGCACACCACTGTCCAAGTCATTGTTGATTTAGTGCCACCCCATGTTGTTGAAGGAGAAGATGTCCTTCTTCGTGTCCGCAATCTGCCAGAAGATCTTGTAGCCTTTGTCTGGCACAAAGGGGCGACAAAGATGGACCTCGGAATTGTACTTTATTCACTGACCACTAATTTAAAAATCACAGGGCCTGGACACAGTGGTAGAGAGATAGTGTACAGAAATGGATCTCTGCACCTCCAAAATGTCACCCAGAAAGACACAGGATTCTACACACTACGATCCTTAAATAGGCATAAAGGAATTGTATCAACAACATCTATATACCTGCATGTATACT

>Mag_Psg3N1 (Microtus agrestis; short-tailed field vole) WGS CADCXT010002299.1

CCTCCCTTTTCTCCTTCTGGCATCTCCCCACTACTGCTCATGTGTCCACTGAATCAGTGCCACCCCTAGTGGCTGAAGGTGATAATGTCCTTATCCTTGTCAACAATCTGCCAGAGAATCTTTTAGCCTTAGCCTGGTTCAAAGGGCTAACAAATATGAAGCAAGGAATCGCATTATATGCACTGCACAAAAATGTAAGTGTTACAGGGCCTGTGCACAGTGGCAGAGAGACAATATATCACAATGGATCCTTGTTGATTGAAAAACTCACCCAGAAGGACACAGGATTCTACACCCTTCGAGCCTATAATAGACGTGGAAGAACTGTATCAAGCACATCCACCTACCTCCATGTGCAAG

>Mag_Psg4N1 (Microtus agrestis; short-tailed field vole) WGS CADCXT010002393.1

CGTCACTTTTAGGCTGCTGCCTATCCACCACTGACTATATCACCATTAAATCTGCCCAACCCCATGTGGCCAGTGGAGAAGACGTCCTTCTCCTTGTCCACAATCTGCCAGAGGATATTCTAGCCTTCGCCTGGTTCAAAGGGGCGACAAGCATGAAACATGGAATTGCAGTATATGCACTGAACAAAAATTTAAGTGCGACAGGGCCTGCACATAGTGGTAGAGAGACAGTGTACCGCAATGGATCCCTGCTACTCCAAAGTGTCACCGAGAAGGACACAGGATCCTATACCCTACGAACCTTAGATAGACACGGAGAGATTGTATCAACAACAACCATGCGCCTCTATGTGTACC

>Mag_Psg5N1 (Microtus agrestis; short-tailed field vole) WGS CADCXT010002299.1

TCTCCATTTTAAGCTCCTGGCATCTGTCCACAAATGCCCATATGACTATTGAAAAAGTGCCAGCCCTAGCTGCTGAAGGAGATGACATATTTTTCCATGTCAATGATCTGCCAGAGAATACTACAACCATAGCCTGGTTCAAAGGTCTAAGAAATACGACACAAGGAATTGGAGCATATGCACCGCTCTTAAATTTAAGTAGGCCAGGTCCTATGTACAGTGGTAGAGAGACAATATATCGCAATGGATCCCTGCTGGTAAAAAATGTCAACCCGACGGACACTGGATTCTATACCCTACGAACTTATAATAATCATGGAACTAGGACATCAATAACATCCGCGTACCTCCAAGTGCATG

>Mag_Psg6N1 (Microtus agrestis; short-tailed field vole) WGS CADCXT010002299.1

TCTCCCTTTTAACCTCCTGGTACCTGTCCACTGCTGTCCATATAACTACTGAGTCAAGCCGAGTGGTTGAAGGAGAAAACATCCTTTTCCTTGTGCATGATCTGCCAGAAAATACTAAATCCTTAGTCTGGTTCAAAGCTCTAAGAAACGTGACAGAAGAAATTGCAGCATATGCACTGCCCTACAATTTGAGTAGGCCAGGTCCTCTGTACAGTGGTAGAGAGACAATATATCGCAATGGATCCCTGATGATAGAAAATATCAACCTCAAGGACACAGGATTCTATATTCTACAAACCTATAACAAACGTAAAAAAGTCATATCAACAACAACCACGTACCTCCAAGTGAATG

>Mag_Psg7N1 (Microtus agrestis; short-tailed field vole) WGS CADCXT010001870.

CCTCCCTTTTAGCCCTCTGGCACCTGTCCATCACTGCCTCAGTGACCATTGAATCAGTGCCACCCCTGATGGCCGAAGGAGATAACATTCTTTTTCTTGTTGACAATCTGCCGGAGAAGACTGTAACCTTAGTCTGGTTCAAAGGGCTAACAAATATGAAAGCTGTGATTGCAATATATGGACGGCACATCAATTTAAGTGCATCTGGGCCTTTGCACAGCGGTAGAGAGACAATATATTACAACGGATCCCTGCTGATTAAAAAAGTTACCCAGAAAGACACAGGATTCTATACCCTACGAAGCTATGATAAGTATTTAAACATCATATCAACAATATACACATATGTCCATGTTCACG

>Mag_Psg8N1 (Microtus agrestis; short-tailed field vole) WGS CADCXT010002153.1

CCTTCCTTTTAACCTGTTGGCACCTGCCCACCACTGCCCAAGTCACCATCGAATTAGTGCCGCCCCAAGTGGTTGAAGGAGAAAACGTTCTTCTACGTGTTCATAATCTACCAGAGAATCTTCTAGCCTTTGTCTGGCACAAGGGGGTGAGGAATATGAGCCTTGGAATTGCACTATATTCACTGGCCAAGGGTTTAAGTGTGACAGGGCCCATACACAGTGGTAGAGAGACAGTGTACAGCAACGGATCCCTGCAGATCCACAATGTCACCCAGAAGGACACAGGATTCTACACCTTTCGAACCATAAATGGACAAGTAGGAGTTGCATCAATAACAACCACATACCTTCACGTGTACA

>Mag_Psg9N1_P (Microtus agrestis; short-tailed field vole) WGS CADCXT010002153.1

CCTTCCTTTTAAGCTGTTGTCATTCACCCGCCACTGCTGAAGTCACCATTGAATCAGTGCAGGCCCAATGTGTTCGAAGGAGACAGTGTCCTTATATATGTCCACAGTCTGCCAGAGAATCTGCTAGCCTTTGCTTGGTTCAAAGGGCTAACAAATATGAAACGCAGAATTGTACTCTATGAACTGAACAACAATTTAAGTTTGCTGGGGCCTGAATACAGCGGTAGAGAGACAGTCTACTGCAATGGATCCCTGTGGATTTCCAATGTCACCCACGTGGACACAGGATTCTATACCCTACGAACCATCAATAGACATTCAAGAATTGTGTCAGTAACAACCATCCACCTCCCTGTATACA

>Mag_Psg10N1 (Microtus agrestis; short-tailed field vole) WGS CADCXT010002299.1

CCTCCATTTTAGGCTTCTGGCACATGTCCACTACTGCCCATGAGACCACTGTGTCACTGCCACGCCAAGTGGTTGAAGGAGACAACGTTCTTTTGCTTGTCCATAATCTGCCAGAGAATCTTATAGCCTTTGCCTGGTTCAAAGGGCTAACAAATATGACGCAAGGAATTGCAGTATATACACTGCACAACAATTTAAGTGCACCAGGGCCTGTGCACAGTAGTAGAGAGACAGTTTATAGCAATGGATCCCTGCTGATAGAAAATGTCACCCAGAAAGACACAGGAATCTATACCCTACGAACCTATAATAGAAGAGGAAAAATTGCATCAACAACATCTATGTACCTCCACGTGCACG

>Mag_Psg12N1 (Microtus agrestis; short-tailed field vole) WGS CADCXT010001870.1

CCTCCCTTTTAATCTTTGGGCACCTGCCCACCACTGCCCGTGTGATCACTGAATTAGTACCACCCGAAGTGGCTGAAGGAGAAAATGTTCTTTTTATTGTCCACAATCTGACAGAGAATGTTAAATCCTTTGCCTGGTTCAAAGGGCTAAAAATGGAGAAACAAGGAATTGTAACGTATAGACGGCAGAAGAATTTAGTTACAAATGGGCCTATGCACAGTGGCAGAGAAACCATATATCGCAATGGATCCCTGCTGCTCCAAAAGGTCTCCCACAATGACACAGGATTCTTTACCCTACAAACCTATGATACACATGAAAAAATCCTATCAACAACTTCTGTGTATCTCCATGTGCATG

>Mag_Psg13N1 (Microtus agrestis; short-tailed field vole) WGS CADCXT010001870.1

CCTTCCTTTTAACCTTCTGGCTCCTCTCCACCACTGCCCATGAGACCACTGTATCAGTGCCACCTATAGTGTCTGAAGGAGATGACGTCCTGTTCCTTGTCCACAATCTGCCAGGGGAAATTGAATCCTTAGCCTGGTTCAAAGGACTAGGAGATGAGGCAGAAGAAATTGCAACATATGAACTGCACAGAGGTTTAAGCAGGCTAGGTCCTGCACACAGCAGTAGAGAGACAATATATCACAACGGATCCATGCTGTTTGAGAAGGTCAACCTGAAGGACACAGAATTCTATACCCTACGGACCTATAACAGAAGTGGAAAAATCATATCAACAGCAAACGTGTACCTCAATGTGTATG

>Mag_Psg14N1 (Microtus agrestis; short-tailed field vole) WGS CADCXT010001870.1

CCTCCCTTTTAACCTTCTGGCACCTCTCCACCACCGCCCGTGAGACCACTGTGTCAGTGCCACCCCAAGCGGCTGAAGGAGATGACGTCCTGTTTCTTGTCCACAATATGCCAGAGGACATTAAATCCTTAGCCTGGTTCAAAGGGCAAGGAAACACAACAGAAAAAATTGCAACATATACACTGCACAACGATTTAAGTAGGCCAGGTCTTGCATACAGCAATAGAGAGACAATATATCACAATGGATCTATGCTGTTTGAGAAGGTCACCCTGAAGGACTCAGGATTCTATACACTACAAACCTATAACAGACATGGAAAAATTGTATCAACAACATACGTGATCCTCAATGTGAAAG

>Mag_Psg15N1 (Microtus agrestis; short-tailed field vole) WGS CADCXT010001870.1

CCTTCCTTTTAACTTTCTGGCACCTGTCCACCACTGCCTTTGTGACCACTGTATCAGTGCCATCCCGAGTGGCCGAAGGAAATGACGTCCTATTCCTTGTCCACAATCTGCCAGAGAAATTTAAAACCGTTGCCTGGTTCAGAGGGCCCTCAAATATGACTGCAATATATGGACTGCCAGACAATTTAAACAGTCCAGGTCCTGCACACAGCGGCAGAGAGACAATATTTCACAATGGATCCATGCTCCTTGAAAAGGTCAACCTGAAGGACACAGGCTTCTATACCGTACGAACCTATAATATACATGGAAATGTCATATCAACAACATACACATACCTCAACGTGTATG

>Mag_Psg16N1 (Microtus agrestis; short-tailed field vole) WGS CADCXT010002299.1

CCTCCCTTTTAACCTGCTGGCACCTGTCCACCACTGTCAAAATCACAATTGACTCAGTGCCACTCCAAGTGGTTGAAGGAGAAAGCGTCCTTCTACATGTCAACAATCTGCCACAGAATCTTCGAAATTTTGCCTGGTTCAAAGGGGTGACAGATATGAAATTCAGAATTGCATTATATTCACTGACCAGTAATCTATGTGTGATGGGGCCTGAAAATAGTGGTAGAGAAGCTGTGTACAGCAATGGATCCCTGTTTCTCAAAAATGTCTCCCAGAAGGACACGGGATTTTATATACTACGAACAGTAATTGGAGGTGGAAAAATTGTATCTACAACCACATACCTCCACGTGTATG

>Mag_Psg19N1 (Microtus agrestis; short-tailed field vole) WGS CADCXT010002153.1

CCTCCCTTTTAATCTCCTGCCACCTCTCTTCTACTGCCAATGTGACCATTGAATTACTGCCAACTCCAGTGGCCGAAGGAGATAACGTCCTTTTCCTCATTCAAAATCTGCCAGAGGAAATAAAAGCTGTAGCCTGGTTCAAAGGGCTTGGAAATAAGAAACAACAAATTACTGTGTATGTACTGGACAAAAAATTAAGTAAGCCAGGTCCTATGCACAGCGGGAGAGAGACAATATATCACAATGGATCCCTGCTTCTTGAAAAGGTCACCCAGAAGGATGCAGGATTCTATACCCTACGAACCTATGATAGAGGTGGAAAATTTGTATCAACAATAACCATATACCTCTACGTGCAAG

>Mar_Ceacam9N (Microtus arvalis; common vole) 27.8.21 WGS VIIT010034676.1

CCTTCCTCTTAACCTGCTGGAATGCACCCACCACTGCCGAACTCACTATTGAATTAGTGCCCCCCATGGTTGCTGAAGGTGGAAACTCCGTCCTGTTTGTGCATAAAATGCCGCTGAACGTCCAGGCATTTTACTGGTACAAACAGAAAGATGCGACTAAGAGCTACGAAGTTGCACGCTACTTAACACCCGATAACACAACGTCGAAGATGCCTCAACACAGCGGTAGAAAAACGGTATTCTACAGTGGATCCCTGCTGATCAGAAATGTCACCCAGGCTGACAGTGGATTCTACACCTTACTGACGTTCAACACAGAAATGCAAAGTGAACTCACACACGTATATCTGGAAGTATACA

>Mar_Psg1N1 (Microtus arvalis; common vole) WGS VIIT010018833.1

CCTTCCTTTTAACCTGTTGGCACCTGCCCACCACTGCCCAAGTCACCATCGAATTAGTGCCACCCCAAGTGGTTGAAGGAGAAAACGTTCTTCTACGTGTTCATAATCTACCAGAGAATCTTTTAGCCTTTGTCTGGCACAAGGGGGTGAGGAATATGAGCCTTGGAATTGCACTACATTCATTGGACAAGGGTTTAAGTGTGACAGGGCCCATACACAGTGGTAGAGAGACAGTGTACAGCAACGGATCCCTGCAGATCCACAATGTCACCCAGAAGGACACAGGATTCTACACCTTTCGAACCATAAATGGACAAGTAGGAGTTGCATCAATAACAACCACGTACCTTCACGTGTACA

>Mar_Psg2N1 (Microtus arvalis; common vole) WGS VIIT010005732.1

CCTACCTTTTAACCTGTTGGCACCTGCCCACCACTGTCCAAGTCATTGTTGATTTAGTGCCACCCCATGTTGTTGAAGGAGAAGATGTCCTTCTTCGTGTCCGCAATCTGCCAAAAGATCTTGTAGCCTTTGTCTGGCACAAAGGGGCGACAAAGATGGACCTCGGAATTGTACTTTATTCACTGACCACTAATTCAAACATCACGGGGCTTGGACACAGTGGTAGAGAGATAGTGTACAGAAATGGATCTCTGCGCCTCCAAAATGTCACCCAGAAGGACACAGGATTCTACACACTACGATCCTTAAATAGGCATAAAGGAATTGTATCAACAACATCTATATACCTGCACGTATACT

>Mar_Psg3N1 (Microtus arvalis; common vole) WGS VIIT010005732.1

CCTCCCTTTTCTCCTTCTGGCATCTCCCCACTACTGCTCATGTGTCCACTGAATCAGTGCCACCCCTAGTGGCTGAAGGTGATAATGTCCTTATCATTGTCAACAATCTGCCGGAGAATCTTTTAGCCTTAGCCTGGTTCAAAGGGCTAACAAATATGAAGCAAGGAATCGCATTATATGCACTGAACAAAAATTTAAGTGTCACAGGGCCTGTGCATAGTGGCAGAGAGACAATATATCACAATGGATCCTTGTTGATTGAAAAACTCACCCAGAAGGACACAGGATTCTACACCTTTCGAGCCTATAATAGACGTGGAAGAATTGTATCAAGCACATCCACCTACCTCCATGTGCAAG

>Mar_Psg4N1 (Microtus arvalis; common vole) WGS VIIT010027407.1

CCTCACTTTTAGGCTGCTGCCTATCCACCACTGACTATATCACCATTAAATCTGTTCAACCCCATGTGGCCAGTGGAGAAGACGTCCTTCTCAATGTCCACAATCTGCCAGAGGATATTCTAGCTTTCGCCTGGTTCAAAGGGGCAACAAGCATGAAACATGGAATTGCAGTATATGCACTGAACAAAAATTTAAGTGCGACAGGGCCTGGACATAGTGGTAGAGAGACAGTGTACCACAATGGATCCTTGCTGCTCCAAAGTGTCACCGAGAAGGACACAGGATTCTATACTCTAAGAACTTTAGATAGACACGGAGAGATTGTATCAACAACAACCATGCGCCTCTATGTGTACC

>Mar_Psg5N1 (Microtus arvalis; common vole) WGS VIIT010018233.1

TCTCCCTTTTAAGCTCCTGGCATCTGTCCACAAATGCCCATATGACTATTGAAAAAGTGCCAGCCCTAGCTGCTGAAGGAGATGACATATTTTTCCATGTCAATGATCTGCCAGAGAATACTACAACCATAGCCTGGTTTAAAGGTCTAAGAAATACGACACAAGGAATTGGAGCATATGCACCGTTCTTAAATTTGAGTAGGCCAGGTCCTATGTACAGTGGTAGAGAGACAATATATCGCAATGGATCCCTGCTGGTAAAAAATGTCAACCCGACGGACACTGGATTCTATACCCTACGAACTTATAATAATCATGGAACTAGGACATCAATAACATCCGCATACCTCCAAGTGCACG

>Mar_Psg6N1 (Microtus arvalis; common vole) WGS VIIT010023848.1

TCTCCCTTTTAACCTCCTGGCACCTGTCCACCGCTGTCCATATAACTACTGAGTCAAGTCGAGTGGTTGAAGGAGAAAACATCCTTTTACTTGTGCATGATCTGCCAGATAATACTAAATCCTTAGTCTGGTTCAAAGTTCTAAGAAATGTGACAGAAGAAATTGCAGCATATGCACTGCCCTACAATTTAAGTAGGCCAGGTCCTCTGTACAGTGGTAGAGAGACAATATATCGCAATGGATCCCTGATGATAGAAAATGTCAACCTCAAGGACACAGGATTCTATATTCTACAAACCTATAACAGACGTAAAAAAGTCATATCAACAACAACCACGTACCTCCAAGTGAATG

>Mar_Psg7N1 (Microtus arvalis; common vole) WGS VIIT010016954.1

CCTCCCTTTTAGCCCTCTGGCACCTGTCCATCACTGCCTCAGTGACCATTGAATCAGTGCCACCCCTGATGGCTGAAGGAGATAACATTCTTTTGCTTGTCGACAATCTGCCGGATAAGACTGTAACCTTAGTCTGGTTCAAAGGGCTAACAAATATGAAAGCTGTGATTGCAATATATGGACGGCACATCAATTTAAGTGCATCTGGGCCTTTGCACAGCGGTAGAGAGACAATATATTACAACGGATCCCTGCTGATTAAGAAGGTTACCCATAAGGACACAGGATTCTATACCCTACGAAGCTATGATAAGTATTTAAACATCATATCAACAACATACACATACATCCATGTTCACG

>Mar_Psg8N1 (Microtus arvalis; common vole) WGS VIIT010013118.1

CCTTCCTTTTAACCTGTTGGCACCTGCCCACCACTGCCCAAGTCACCATCGAATTAGTGCCGCCCCAAGTGGTTGAAGGAGAAAACGTTCTTCTACATGTTCATAATCTACCAGAGAATCTTTTAGCCTTTGTCTGGCACAAAGGGGTGAGGAATATGAGCCTTGGAATTGCACTATATTCACTGGCCAAGGGTTTAAGTGTGACAGGGCCCATATACAGTGGTAGAGAGACAGTGTACAGCAACGGATCCCTGCAGATCCACAATGTCACCCAGAAGGACACAGGATTCTACACCTTTCGAACCATAAATGGACAAGTAGGAGTTGCATCAATAACAACCACGTACCTTCACGTGTACA

>Mar_Psg9N1_P (Microtus arvalis; common vole) WGS VIIT010024669.1

CCTTCCTTTTAAGCTGTTGTCATTCACCTGCCACTGCTGAAGTCACCATTGAATCAGTGCCGCCCAATGTGTTCGAAGGAGACAGTGTCCTTCTATATGTCCACAGTCTGCCAGAGAATCTTCTAGCCTTTGCTTGGTTCAAAGGGCTAACAAATATGAAATTCAGAATTGTACTCTATGAACTGAACAACAATTTAAGTTTGCCGGGGCCTGAATACAGCAGTAGAAAGACAGTCTACCACAATGGATCCCTGTGGATTTCCAATGTCACCCACGTGGACACAGGATTCTATACCCTACGAACCATCAGTTGACATTCAAGAATTGTGTCAATAACAACCATCCACCTCCCTGTATACA

>Mar_Psg10N1 (Microtus arvalis; common vole) WGS VIIT010013065.1

CCTCCATTTTAGGCTTCTGGCACATGTCCACTACTGCCAATGAGACCACTGAGTCACTGCCACGCCAAGTGGTTGAAGGAGACAACGTTCTTTTGCTTGTCCACAATCTGCCAGAGAATCTTATAGCCTTTGCCTGGTTCAAAGGGCTAACAAATATGACACAAGGAATTGCAGTATATACAGTGCACAACAATTTAAGTGCACCAGGGCCTGTGCACAGTAGTAGAGAGACAGTTTATAGCAATGGATCCCTGCTGATAGAAAATGTCACCCAGAAAGACACAGGAATCTATACCCTACGAACCTATAATAGAAGAGGAAAAATTGCATCAACAACATCTATGTACCTCCATGTGCACG

>Mar_Psg11N1 (Microtus arvalis; common vole) WGS VIIT010045491.1

CTTCCCTTTTAACATACTGGTATCTACCCACCACTGCCCAAGTCACCATTGAATTAGTGCCACCCAACGTGTTCCAAGGAGAAAATGTCCGTCTAGAGGTCCACAATCTGCCAGAGGACTTTCTAGCCTTTGCTTGGTACAGAGGGGTGACAAACATGAAACGCGGAATTGCAGTCTATGCCAAAAGAAAAGGTTTAAATGCAACGGGGCCTGCGTACAGTGGTAGACAGACAGTGTACAGTGATGGATCACTGCTGCTCCAGCGTGTCATCCTCAAGGACACAGGATTCTACACCCTACGAGTCATAAGTAGACAAGGAGAAATTATATCAACAACATCCATGTTCCTCCATGTGCAGA

>Mar_Psg12N1 (Microtus arvalis; common vole) WGS VIIT010018950.1

CCTCCCTTTTAATCTTTGGGCACCTGCCCACCACTGCCGGTGTGATCACTGAATTAGTACCACCTGAAGTGGCTGAAGGAGAAAATGTTCTTTTTATTGTCCACAACCTGCCAGAGAATGTTAAATCCTTTGCCTGGTTCAAAGGGCTAAAAATTGAGAAACAAGGAATTGCAACGTATAGACGGCGCAAGAATTTAGTTACAAATGGGCCTATGCACAGTGGCAGAGAGACCATATATCGCAATGGATCCCTGCTGCTCCAAAAGATCTCCCATAATGACACAGGATTCTTTACCCTACAAACCTATGATAGACATGAAAAAATCCTATCAACAACTTCTGTGTATCTCCATGTGCACG

>Mar_Psg13N1 (Microtus arvalis; common vole) WGS VIIT010026754.1

CCTTCCTTTTAACCTTCTGGCTCCTCTCCACCACTGCCCATGAGACCACTGTATCAGTGCCACCCATAGTGTCTGAAGGAGATGATGTCCTGTTCCTTGTCCACAATCTGCCAGGGGAAACTGAATCCTTAGCCTGGTTCAAAGGGCTAGGAGATGAGGCAGAAGAAATCGCAACATATGCACTGCACAGAGGTTTAAGCAGGCCAGGTCCTGCGCACAGCAGTAGAGAGACAATATATCACAACGGATCCATGCTGTTTGAGAAGGTCAACCTGAAGGACACAGAATTCTATACCCTACGAACCTATAATAGAAGTGGAAAAATCATATCAACAGCAAACGTGTACCTCAATGTGTATG

>Mar_Psg14N1 (Microtus arvalis; common vole) WGS VIIT010028229.1

CCTCCCTTCTAACCTTCTGGCTCCTCTCCACCACCGCCCGTGAGACCACTGTGTCAGTGCCACCCCAAGTGGCTGAAGGAGATGACGTCCTGTTCCTTGTCCACAATCTGCCAAAGAACGTTAAATCCTTAGCCTGGTTCAAAGGGCAAGGAAACACAACCAAAAAAATTGCAACATATACACTGCACAATGATTTAAGTAGGCGAGGTCTTGCGTACAGCAATAGAGAGACAATATATCACAACGGATCTATGCTGTTTGAGAAGGTCACCCTGAAGGACTCAGGATTCTATACACTACAAACCTATAACAGACATGGAAAAAATGTATCAACAACATCCGTGATCCTCAATGTGGAAG

>Mar_Psg15N1 (Microtus arvalis; common vole) WGS VIIT010037449.1

CCTTCCTTTTAACCTTCTGGCACTTGTCCACCACTGCCTTTGTGACCACTGTATCAGTGCCATCCCGAGTGGCCGAAGGAAATAACGTTCTATTCCGTGTCCACAATCTGCCAGAGAAATTTAAAACCGTTGCCTGGTTCAGAGGGTCCTCAAATATGACTGCAATATATGGACTGCCCGACAATTTAAGTAGGCCAGGTCCTGCACACAGCGGCAGAGAGACAATATTTCACAATGGATCCATGCTCCTTGAAAAGGTCAACCTGAAGGACACAGGCTTTTATACTGTACGAACCTATAACATACCTGGAAATGTCATATCAACAACATACACATTCCTCAACGTATATG

>Mar_Psg16N1 (Microtus arvalis; common vole) WGS VIIT010005732.1

CCTCCCTTTTAACCTGCTGGCACCTGTCCACCACTGTCAAAATCACAATTGATTCAGTGCCACTCCAAGTGGTTGAAGGAGAAAGCGTCCTTCTACATGTCAACAATCTGCCACAGAATCTTCGAAATTTTGCCTGGTTCAAAGGGGTGACAAATATGAACTTCAGAATTGCATTATATTCACTGACCAGTAATCTATGTGTGATGGGGCCTGAAAATAGTGGTAGAGAAGCTGTGTACAGCAATGGATCCCTGTTTCTCAAAAATGTCTCCCAGAAGGACACGGGATTTTATATACTACGAACAGTAATTGGAGGTGGAAAAATTGTATCTACAACCACATACCTCCACGTGTATG

>Mar_Psg19N1 (Microtus arvalis; common vole) WGS VIIT010040764.1

CCTCCCTTTTAACCTCCTGGTACCTGTCTTCCACTGCCAATGTGACCATTGAATTACTGCCAACCCCAGTGGTGGAAGGAGATAACATCCTTTTCCTTGTCCACAATCTGCCAGAGGAAATAAAAGCTGTAGCCTGGTTCAAAGGGCTGGGAAATAAGAAACAACAAATTGCAGTGTATGTACTGGACAAAAAATTAAGTAAGCCGGGTCCTATGAACAGCGGGAGAGAGACAATATATCACAATGGATCACTGCTTTTTGAAAAGGTCACTCAGAAGGATGCAGGATTCTATACCCTACGGACCTATGATAGAGGTGGAAAATTTGTATCAACAATAACCATGTACCTCTACGTGCAAG

>Mau_Ceacam9N (Mesocricetus auratus; Golden hamster) WGS AFVMI010000538.1

CCTTGCTCTTAGCATGCTGGAACACACCCACCACTGCCCAGCTCACTATTGAACTAGTGCCCCCCATGGTGGCTGAAGGTGGAAACTCCGTTCTATTCGTGCATAAAATGCCATTGAACGTGCAGGCATTTTACTGGTACAAACAGAAAGATCCCACCAGGAGCTATGAAGTTGCGCGGTACTTAACACCCGATAACACAACGTCGAAGATGCCTCAACACATTGGCAGGAGGACGGTATTCTACAGTGGATCCCTGCTGATCAGAAACGTCACCCAGGCTGACAGTGGACTCTACACCTTGCTAACATTTAACACAGAAATGGAAAGTGAGCTAACACACGTGCATCTGGAAGTACACA

>Mau_Ceacam11N1 (Mesocricetus auratus; Golden hamster) WGS JAFVMI010000582.1

CCTCCCTTTTAATCTTCTGGCTGACTTCTACATTTGCCCAGCTCACCATTGAATCAATGCCACCAATTTCTGCTGAAGGCGATAATGTTCTTCTGTTTGTTCATAATCTGCCTGAGAATGTTCAAGCCTTTTCCTGGTACACAGGAGTTATGGTGCTCAAGAGCCGTGAGATTGCAAGATACGCGAAAGCTACCAATTCATGTGTGCTGGGAACTGCACACAGTGGTAGAGAGACAATATTCAGTAACGGATCTCTGCTGATCAAGAATGTCACCAGGAAGGACTCGGGTTACTACCTCCTACAGACTCTTGATACAAGCTTGAAATCTGAAATAACACGTGTGGAGTTTTTTGTACATA

>Mau_Ceacam15N (Mesocricetus auratus; Golden hamster) WGS AFVMI010000538.1

CTGCCCTTTCGGCCTGCTGGACCTCAAAGGCTGTGGCGGGCCCATCTGTCGAGGCTGTGCCACCCTCTGTTCCTGAGGGGGAAAATGTTCTTCTGCGCGTTGATAACCAGGCAGAGAAGTTCCAGGCCTTTTTCTGGTACAAAGGGAAACGTGCCTTCGAAGAATTTAAGATCGCACATTATGAAACAGCCTCTCAGACACTTAAACGGGGGCAGAAATACAGCGGGAGGGAGAGGGTGTACAGCAACGGATCCCTGCTGCTCCAGAACGTCACCCAGGAAGACACCGGGATCTACACCCTAGAAACCTTTGGAACACATTATCAATGTGAAATAACACATGTCCACCTCCAGGTGTACA

>Mau_Psg1N1 (Mesocricetus auratus; Golden hamster) WGS APMT01151843.1

CTTCCCTTTTAACCTGCTGGCACCTGACCACCACTGCCCAAGTCAGCATTGAGTCAGTGCCACCCCAAGTGATTGAAGGAGAAAATGTTCTACTACAGGTTCATAATCTACCAGAGAATCTTCTAGCCTTTGTCTGGCAAAAAGGGGTGAATGAGATGAACCTTGGAATTGCACTATATTCACTGGCTGAGAATGAAAGTATGACAGGGCCCTCACACAGTGGCAGAGAGACAGTGTACAGCAATGGATCCCTGCAGATCCGCAATGTCACCCAGAAGGACACAGGATTCTACACCTTCCGAACCATGAATCGACATGAAAAAGTATTGTCAGTGACAACCACATACCTTCATGTGCACA

>Mau_Psg2N1 (Mesocricetus auratus; Golden hamster) WGS JALRLW010003051.1

CCTCTCTTTTAACCCTCTGGCACTTGCCCACCACTGCCCATGTGACCACTGAATTGGCACCACTTCAAGTGGCTGAAGGAGAAAATGTCCTTTTTCTTCCTCATGATTTTCCTGAGAATGTTACAGCCTTTGCCTGGTTCAAAGGACTAAGAAACATGAAACAAGGAATCGCAGTTTATATACTGGGCCACAATTTAAGTGGTCCGGGGCCTGTGCACAGCAGTAGAGAGACAATATATCCCAATGGTTCCCTGCTGCTGGAGAAGGTCACACAGAAGGATTCAGGATACTATACCCTACGAACCTATAATGGACATGCAAAAATCATATCAACCACATCTATATACCTTCATGTGCATG

>Mau_Psg3N1 (Mesocricetus auratus; Golden hamster) WGS JALRLW010003051.1

CCTCCCTTTTAATGTTCTGTCGCCTGTCCAACACTGCCTATGTGACCACAGAATCAGTGCCACCCCTAGTGACCAAAGGAGATGATGTCCTTCTCCTTGTCCACAATCTGCCAGAAAATGTACAAGCCTTTGCCTGGTTCAAAGGGCGAACAAACATGGAAGACAAAATTGCAGTATATGAATTGTTCAACAATGTAAGTGGGCCAGGGCCTGTGCACAGTGGGAGAGAGACAATATATCACAATGGATCCCTGCTGATCGAAAAGCTCACCAAGAAGGACACAGGATTCTATACCATACGAACCTATAATGAACATGCAAAAATCATATCAACAACATCCATGTACCTCCACGTGCAGG

>Mau_Psg4N1 (Mesocricetus auratus; Golden hamster) WGS JALRLW010003051.1

CCTCCCTTTTAATCTTCTGCCACCTCTCCAACACTGCCCATGTGACTGTGACCATTGAATCAGTGCCACCCCTAGTGGCCAAAGGAGATGATGTCCTTTTTCTTGTCCATGATCTGCCAGAAAATGCTCAAATATTAGCCTGGTTCAAAGGGCTAACAAACATGAAAGACGCAATTGCACTATATGGACTGTTCAACAATGTAAGTGGGCCAGGGCCTGTGCACAGTGGTAGAGAGACAATATATCGCAATGGATCCCTGCTGATCGAAAAGCTCACTGAGAAGGACACGGGATTCTATACCATACGAACCTATAATGAACATATAAAAATTGTATCAACAACATCCACGTACCTCCACGTGCAGG

>Mau_Psg5N1 (Mesocricetus auratus; Golden hamster) WGS JALRLW010003051.1

CCTCTCTTTTAACCTGTTGGCATCTGTCCACCACTGTCAAAATCTCCATTGAGTCAGTGCCATCTCAAGCGATTGAAGGAGAAAACGTTCTTCTACGTCCCAGCAACCTGCCAGAGGATATTCTAGCTTTTGCCTGGTTCAAAAGAGTGAAGAGAACGAGGCATGGAATTGCACTATATTCACTGACCAAAAATCTAAGTAGGCCAGGGCCTCAATACAGTGGTAGAGAAACTTTGTATAGCAATGGATCCTTGTGGCTCCAAAATGTCACTCAGAGGGACAAAGGATTCTACGTCCTACGAACAATAAATAGAAGTGGAAAAATTGCATCTACGACAGCCATGTACCTCCATGTGTACA

>Mau_Psg6N1 (Mesocricetus auratus; Golden hamster) WGS JALRLW010003051.1

CCTACCTTTTATCTTTCTGGCACATGTCCATTACTGCCCATGTGATCATTGAATTAGTGCCACCCCAAGTGGCAGAAGGAGAAAACATCCTTTTGGCTGTCCACGATCTGCCAAAGGATCTTAAAGCCTATGCCTGGTTCAAAGAGATAACGAACAAGACGCAAGGAATTGCGCTGTATACACTGCATAACAATTTAAGTGCTCCTGGGCCTCTACACAGTGGGAGAGAGACAGTGTATCACAATGGATCCCTGCTGCTTGAAAATGTCACCGAGAAGGACACAGGAATCTATACCCTACAAACCTATAATATACATGGGAAAATCTTATCAACAACATCCAGTTACCTCCATGTACACG

>Mau_Psg7N1_P (Mesocricetus auratus; Golden hamster) WGS JALRLW010003051.1

CCTCCCTTTTAATCTGTTGGCACCTGCCCACCACTGTCCAAGTAATTGTTGAATTAGTCCCACCCCATGCTGTCGAAAGAGAAAGTTTCCTTCTCCATGTCTGAAATATGCCAGAGAATCTTGTACCCTTTGCCTGGTACAAAGGTGTGACAAAATGAACCTTGGAATTATATTTTATTCACTGACTGCTATTTTTAACAGTGATGGGGCCTGAACAGGGGTAGAGAGACAGTGTACAGAAATGGACCCCTCTACCTCCAAAATGTCACACAGAAGGACACAGTGTTCTATGCTCTATGATCCATAAATACAATAAAGGAATTGTATCAACAACATCCATATACCTTCATGTGTACT

>Mau_Psg8N1 (Mesocricetus auratus; Golden hamster) WGS APMT01188643.1

CCTGCCTTTTAACCTCCTGTCACCTGTCCACCACTGCTCATGTGACCACTGAATTAGTACCACGCCTAGTTGCTGAAGGAGATAACGCCCTTTTCCTTGTCCATGATCTGCCTGAGAATATTACAAGCTTAGCCTGGTTCAAAGGGGTAAGAAATATGAAACAAGAAATTGCAATATATGCACTGGACAAGAATTTAAGTCAGCCAGGGACTGTGCACAGTGGTCGAGAGACAATATGTCGCAATGGATCTTTGTTGCTCAAAACTGTCAACCAAAATGATGCGGGATTCTATACCTTACGGACCTATAATAGAGATGGAAAAATCATATCAACAACATCCATGTATCTCCATGTGCAGG

>Mau_Psg9N1 (Mesocricetus auratus; Golden hamster) WGS APMT01188646.1

TTTCCCTTTTAATCTGTTGGCACCTCTCTACCACAGCCCATGTCACCATTGAATCTGTCCCACCCCATGTGGTAGAAGGAGATAATGTCCTTTTCCTTGTCCACAATCTGCCAGACAATGTTCTAGAATTAGCTTGGGCCAAAGGGGTGAACACTATGAACCTTGGAATTGGAACATATAAGCGGAATAAAAACTTATGTGTgccaggaattgcaaatagTGGTAGAGAATCAGTGTACAGCAATGGAACCCTGCTGCTCAGAAATGTGACAAAGAAGGACACAGGATTCTATACCCTAAGAGCCTTCAGTAGACGTGCAATGATTGAGTCAACAACAACCATTTACCTTCATGTGCACA

>Mau_Psg10N1 (Mesocricetus auratus; Golden hamster) WGS JALRLW010002286.1

CCTCCTTTTTAACCTCCTGGCACCTGCTCACGACTGCCCATGTGATTACTGAAGTAGTGCCACCCTTGGTGGCTGAAGGAGACAACGTCCTTTTCCTAGTCCATTATCTGCCAAAGAAAATTAAAGCCTTAACCTGGTTCAAAGGTGTAACAAATACGACACAAAGCATTGTAACATATGCTCTGAACAACAAATTCAGTCAGCCAGGGCCCATGCACAGTGGTAGAGAGACAGTATATCACAATGGATCCCTCCTGATTGAAAACGTTAACCTGAAGGACACAGGATTTTATACCCTACGGACCTATAATAGACGTGGAAAAATCTTAACAACAGCATACCTGTACCTTCTCGTGCACA

>Mau_Psg11N1 (Mesocricetus auratus; Golden hamster) WGS APMT01188647.1

CCTCCCTTTTAACATGCTGGCACGTCTCCACCTCCACTGCACATGTCACCATTGAATCTGTCCCACCCCACGTGGTTGAAGGAGAAAATGTCCTTCTTCTTGTCCGCAATCTGCCAGAGAATATTATATCCTTGGTCTGGTTCAAGGATTTGAAAATTATAAGAAGTGCAATTGGATTATATAATGTGAGATACAATGTAAGTGCTCCAGGGCGTTTACATAGTGGTAGAGAGACAATGTACCGCAACGGATCTCTGCTGCTCAGAAACGTCACGGCGAAGGACACAGGATACTATATCCTGAGAACCTTAAATAGACATGTAGATATTGTGTCAACAACAGTCACACACCTTCTTGTGCACA

>Mau_Psg12N1_P (Mesocricetus auratus; Golden hamster) WGS APMT01215043.1

CCTCCCTTTTAACATGCTGGCACGTCTCCACCACCACTGCCCATGTCACCATTGAGTCTGTCCCATTGAAAGTGGTTGAAGGAGAAAACGTCCTTCTTCTTGTCCACAATCTGCCAGAGAATCTTGTAGCCCTAGTCTGGTTCAAAGGGGTGGAAATTACAGACAATGTAATTGGATTATATACAGTGAACCAAGATTTAAGTGTTCCAGAGCTTGTATACAGTGGTAGAGAGACAGTGTACTGCAATGGATCTTTGCTGATCAGAAATGTCATGAAGAATGACACGGGATTCTATACCCTATGAACCTTAAATAAACATGTATATATTGCAACAACAACCATGTACCTTTTCGTGCACA

>Mau_Psg13N1 (Mesocricetus auratus; Golden hamster) WGS APMT01188653.1

CATCCCTTTTAACATGCTGGCACATCTCCACCTCCACTGCACATGTCACCATTGAATCTGTCCCACCCCATGTGGTTGAAGGAGAAAATGTCCTTCTTCTTGTCGACAATCTGCCAGAGAATCTTGCAACGTTAGTTTGGTCCAAAGGGGTGAAAATTACGGACAATATAATTGGTTTGTATGCACTGAACAAAGGTTTAAGTGCTCCAGGGCCTCTACACAGTGGTAGAGAGACACTATACCACAATGGATCCCTATTGCTCAGAAATGTCACATGGAAGGACACAGGCTCATATACCCTACAAACCTTAAATAGACAAGAAGATATTGTATCAACAACAACCATTGACCTTCATGTGCACA

>Mau_PSG14N1 (Mesocricetus auratus; Golden hamster) WGS JALRLW010002286.1

CGTCCCTTTTAATATGCTGGCATCCAACCTCCACTGCCGAAGTCACCATTGAATCAGTGCCGTCCCAAGTGGTCAAAGGAGAAAATGTCCTTTTCCTTGTCCATGATCTGCCAGACCATGTTTTATCCCTAACCTGGTTCAAAGGCGAGGCAATAATAGACAATGCAATCGCATTGTATGCACCGAATGGCAATGTACGTGCACTCGGGCCTTTACACAGTGGTAGAGAGACAATGTACCAAAATGGATCCCTGTGGATTCAAAATGTCACCCAGAATGACACAGGAGTCTATACTCTAGGAATCTTAAGAACACCAACAGAAAATGTATCAACTGCATCCACGTACCTTCAAGTGCTCT

>Mau_Psg15N1 (Mesocricetus auratus; Golden hamster) WGS JALRLW010003051.1

TCTCCCTTTTAACCTGCTGGGATCTCTCCACCACTGACCATATCACGATCAGATCAGTTCCACCTCAAGTGGTCAGTGGAGAAAACGTCCTTTTCCTTGTCCACAATGTGCCAGAAGATATTCTGGCCTTTGCCTGGTACAAAGGCAAGGCAATCATGAAACATGGAATTTCACTATATTCACGGCACATGAATTTAAGTGTGACAGGGTTTGCACACAGTGGAAGAGAGACAATATATCGCAATGGATCCCTGCTCCTGGAACGTGTCACTGAGCAGGACTCAGGAATCTACACCCTACAAACCATAGATAGACAGCTAAACATTGGGTCAACAACAATCATGCGCCTTCACGTGTACC

>Mau_Psg16N1 (Mesocricetus auratus; Golden hamster) WGS JAFVMI010000582.1

CCTCCCTTTTAACCTGCTGGCATCTCTCCACCACTGATCATATCACCATTCGATCAGTTCCACCCCAAGTGGCCAGTGGAGAAAACGTCCTTTTCCTTGTCCACAATATGCCACAGGATATTCTGGCCTTCCTCTGGTACAAAGGCAAGGCAAAAATGAAACATGGAATTGCACTGTATGCACTGCACATGAATTTAAGTGTGACAGGGTTTGCACACAGTGGAAGAGAGACAATATATCGCAACGGATCCCTGCTCCTGGAACATGTCACTGAGCAGGACTCAGGAATCTACACCCTACGAACCATAGATAGAAGGCTAAAGATTGTATCAACAACAATCATGAACCTTCATGTGTACC

>Mau_Psg17N1_P (Mesocricetus auratus; Golden hamster) WGS JAFVMI010000582.1

CCTCTCTTTTTACCTGCTGGCATCTCTCCACCACTGACTATATCACCATATGATCAGTTCTACCCCAAGTGGCCAGTGGAGAAAACATCCTTTTCCTTGTCTACAATCTGCCAGAGGATATTATGGCCTTTGCCTGGTACAAAGGCAAGGCAATCATGAAACATGGAATTTTGCTGTATGCACTGCACAAGAATTTAAGTGTGACAGGGTTTGCATACAGTGGAAGAGAGAAAATATTTCACAATGGATCCCTGCTCTTGTAACGTGTCACTGAGCAGGACTCAGGATTCTACACCCTACAAACCATAGATAGACATCTCGAGATTGTGTCAACAACAATCATGCGCCCTCACGTGTACC

>Mau_Psg18N1_P (Mesocricetus auratus; Golden hamster) WGS JAFVMI010000582.1

ACTGCCTTCTAACCTCCTGGCACCTGTTCACCACTGCCCATGTGACCACTGATCCAGTGCTACTCCATGTGGTCAAAGGAGATAAAGTCCTTTTCCCTGCCCACGATCTGGCAGAGAATCATATAGTCTTAGCCTGGTTCCAAGGTCTAAAAAATATGACGCGGAGAATTGTAATATATAGCCTTGAACAACAATTTAAGTGTGCTGGGACTTGTGCGCGGCTGTAGAGAGACAGTGTACCACAATGTATCCCTGCTGCTTGAAAAGCTCAACCTGAAGGAAACAGGATTCTGTACCATTTGAACCTATAGCAAATGTGCCAGCATCACATGCACAACAGCCATGTACCTCCACATGAACT

>Mau_Psg19N1 (Mesocricetus auratus; Golden hamster) WGS JALRLW010003051.1

TTTGCCTTATATCCTTCTGGCCCCTGCCCACCACTGCCCAGTATTCCAGCGAAACAGTGCCAACTCTAGTGGCTGAAGGAGATAATGTTCTTATCCCCGTACATAATCTTCCAAAGAAAATTAAATCTATAACCTGGTACAAAGAGATAAAAAATGAGACAAAAGCAATCGCAATATATGAACTGCGCAAGAATTCAAGTCAACCCGGGCCTGCACACACTGGCAGAGAGACAATTTTTTACAATGGAACCCTGCTGTTTGAAAAAGTGAACATGAATGACAGAGGATTTTATACTCTACGCACCCATAACAGACGTGGAAAAATTGTATCAACAACAAAGAAGTATCTCAACGTACTCC

>Mau_Psg20N1 (Mesocricetus auratus; Golden hamster) WGS JALRLW010003051.

CCTCCCTTTTAACCTGCTGGCATCTCTCCACCACTGACCATATCACCATTCGATCATTTCCACCCCAAGTGACCAGTGGAGAAAACGTCCTTTTCCTTGTCTACAGTCTGCCAGAGGATATTATAGCCTTTGCCTGGTACAAAGGCAAGGCAAGCATGAAACAAAGAATATTACTGTATGCACTGCAGAAGAATTTAAGTGAGACAGGGTTTGCACACAGTGGAAGAGAGACAGTATATCGCAACGGATCCCTGCTCCTGGAACGTGTCACTGAGCAGGACTCAGGACTCTACATCCTACAAACCATAGATAGACATCTAGAGATTGTGTCAACAACAATCATGCACCTTCATGTGTACC

>Mau_Psg21N1 (Mesocricetus auratus; Golden hamster) WGS JAFVMI010000542.1

CCTCCCTTTTAGCCTGCTGGCATCTCTCCACCACTGACCATATCACCATTCGATCATTTCCACCCCAAGTGGCCAGTGGAGAAAACGTCCTTTTCCTTGCCTACAATCTGCCAGAGGATATTATAGCCTTTGCCTGGTACAAAGGCAAGGCAAGCATGAAACATGGAATTTCACTGTATGCACTGCAGAAGAATATAAGTGTGACAGGGTTTGCACACAGTGGAAGAGAGACAATATATCGCAACGGATCCCTGCTCCTGGAACGTGTCACTGAGCAGGACTCAGGACTCTACATCCTAGTAACCATAGATAGACATCTAGAGATTGTGTCAACAACAATCATGCGCCTTCATGTGTACC

Mca_Ceacam9N (Mus caroli; Ryukyu mouse) WGS FMAL02019644.1

CCTTCCTCTTAACCTGCTGGAATGCACCCGCCACTGCCGAGCTCACTATTGAATTACTGCCACCCATGGTTGCCGAAGGTGGAAACTCCGTTCTGTTTGTGCATGAAATGCCACTGAACGTCCAGGCGTTTTACTGGTACAAACAGAGAGATTCGACAAAGAGCTACGAAGTCGCACGGTACTTAACACCCACGAACCAAAGTTCGAAGATGCCTCAGCACAGTGATAGGAAAACCGTATTCTACAGTGGATCCCTGCTGATCAGAAACGTCACCAAGGCTGACAGTGGAGTCTATACCTTACTGACATTTAACACGGAAATGGAAAGCGAATTAACACATGTGCATCTGGAAGTGCACG

>Mca_Ceacam11N1 (Mus caroli; Ryukyu mouse) WGS FMAL02028168.1

TCTCCCTTTTAACCTGCTGGCTGATTCCAACTACCACCCAGATCACCATTGAATCAGTGCCTCCCATTGCTGTTGAAGGGGAAAATGTTCTTCTGTTTGTGCATAACTTGCCAGAGAATGTTAAAGCCCTTTCCTGGTACACAGGAGTTAAACCACTCAAGAGTTGTGAAATTGCAAGTCATGTGATAGCTACCAATTCTACTGTGGTGGGACCTGCACACAGTGGTAGAGAGACTGTACTCAAAAATGGATCTCTGCTTATCAAGAGTGTTACCAGAAAAGACTCAGGTTACTACACTCTACAAATACTTGATACATCTTCAAGACCTGAATTAATACGTGCAGAATTCTTTGTTCACA

>Mca_Ceacam12N1 (Mus caroli; Ryukyu mouse) WGS FMAL02028751.1

TCTCCATTTTAACATGCTGGCTGCTTCCCACTACTGCCCAGATCACTATTGAATCAGTGCCTCCCATTGCTGTTGAAGGGGATAATGTTCTTCTGTTTGTGCAAAATTTGCCAGAGAATGTTCAAACCCTTTCCTGGTACAGAGGAGGTAAACTGCTCAAGATGTTTGAAATTGCAAGACATGTGATAGCTACCAATTCTAGTGTGATGGGACCTGCACACAGTGGTAGAGAGACAGTGCTCAATAATGGATCTCTGATGATCAAGAATGTCACCAGAAAAGTCTCGGGATACTACACTCTACAAATACTTGATACAACCTCAAGACGTGAAATAACACGTGCAGAATTCTTTGTACAGA

>Mca_Ceacam13N1 (Mus caroli; Ryukyu mouse) WGS FMAL02028449.1

TCTCCCTTTTAACATGCTGGCTGCATCCCACTACTTCTCAGCTCACCATTAAATCAGTGCCTCCCATTGCTGTTGAAGGGGAAAACGTTCTTCTGTTTGTGCATAACCTGCCAAAGAATGTTAAAGCCTTTTCCTGGTACTCAGGAGTTGCACCGTTCAAGTGTTGTGAAATTGCAAGTCATGTGATAGCTACCAATTTTACTGTAGTGGGACTTGCACACAGTGGTAGAGAAACAGTACTCAACAATGGATCTCTGTTGATCAAGAGTGTTACCAAAAAAGACTCAGGATACTACACTCTACGAACAATTGATTCAACCTCAAGACCTGAAATAATACGTGCAGAATTCTTTGTACACC

>Mca_Ceacam14N1 (Mus caroli; Ryukyu mouse) WGS FMAL02028489.1

TCTCCCTTTTAACCTGCTGGCTGATTCCCACTACTTCCCAGCTCACCATCAAATCAGTGCCTCCCATTGCTGTTGAAGGGGAAAATATTCTTCTGTTTGTGCATAACCTGCCGAAGAATGTTAAAGTCTTTTCCTGGTTCACAGGAGCTAGAGTGCTCAAGAGTTGTGAAATTGCAACTCATGTGATAGCTGTCAATGCTACTGTGATCGGATTTTCACATAGTGGTAGAGAGACATTGTTCAAAAATGGATCTCTGCTGATCAAGAGTGTCACCAGTAAAGACTCAGGATACTACACTCTACGAATAATTGATGCAACCTCAAGACCTGAAATAATACGTACAGAATTCTTTGTACACA

>Mca_Ceacam15N (Mus caroli; Ryukyu mouse) WGS FMAL02019644.1

CCTCACTTTTAATCTGCTGGAACTGGTCCACTGCAGCACTGCTGACCTCTAAAGAAATGCGCTTCTCAGCTGCTGAAGGGGCAAAGGTTCTTCTCTCTGTTCCTGACCAGGAAGAGAACCTCCTCTCCTTTTCCTGGTACAAAGGGAAGGATGTAAATGAGAATTTTACAATTGCACATTATAAAAAGTCCAGCGATTCACTTCAACTTGGAAAGAAAGTCAGTGGCAGGGAAGAAATCTATAAGGATGGCTCCATGATGCTCCGGGCCATCACCCCGGAAGACACGGGATTCTACACGTTACAAACCTTTAAAGCACACGGTCAACAGGAAGTAACACATGTCCATCTCCAAGTATACA

>Mca_Psg16N1 (Mus caroli; Ryukyu mouse) WGS FMAL02019648.1

CCTCCCTTTTAGCCTGCTGGCTCCTGTCCACCACTGCCCAGGTCACCATTGAATCAGTGCCATTCAATGTGGTTGAAGGAGAAAATGTCCTTCTTCTTGTTGACAATCTGCCAGAGAATCTTATAGCCTTAGCCTGGTACAGAGGGCTGAGGAAAATTGTTGTATACACACTGAACACTAAAGTAAGTGTGATGGGGCAAATGTACAGTGGTAGAGAGATAGTGTCCAGCAACGGGTCCCTGTGGATCCACAATGTCACCCGGAAGGACACAGGATTCTACACCCTACGGACCGTAAATAGACGTGGAGAAATTGTATCAACATCATCCATGTACCTCTACGTGTACA

>Mca_Psg18N1 (Mus caroli; Ryukyu mouse) WGS FMAL02029928.1

CCTCCCTCTTAACCTGCTGGCTCCTGCCCACCACTGCCAGAGTCACCATTGAATCCTTACCACCCCAAGTGTTTGAAGGAGAAAATGTTCTTCTACGTGTTACCAATATGCCAGAGAATCTTCTAGTGTTTGGCTGGTACAGAGGAATGACAAATTTGAGGCAAGCAATTGCACAGCATTGGCTGTACTACTATAGTGTAATGGCGAAGGGGCTGAATCACAGCGGCAGAGAGAAATTATACATCAACGGGACCCTGTGGATCCAAAATGTCACACAGGAGGACACAGGATATTACACTTTTCAAACCATAAGTAAACGAGGAGAAATAGTATCAAATACATCCTTGTACTTGCACGTGTACT

>Mca_Psg19N1 (Mus caroli; Ryukyu mouse) WGS FMAL02019672.1

CCTCCCTCTTAACCTGCTGGCTCCTGTCCACCACTGCTCGAGTCACCATTGAATCCGTACCACTTAAATTGGTTGAAGGAGAAAATGTTCTTCTACGAGTCTACAATCTGCCAGAGAATCTTCGAGTCTTTGCCTGGTACAGAGGGGTAATAAAATTTAAGCTTGGAATTGCACTGTATTCACTGGACTATAACACAAGTGTGACAGGACCTGAGCACAGTGGTAGAGAGACATTGCACAGCAACGGGTCCCTGTGGATCCAAAATGTCACCCGGGAAGACACAGGATATTACACGTTTCAAACCATAAGTAAAAATGGAAAACTGGTATCAAATACATCCACATTTCTTCAGGTGTACT

>Mca_Psg20N1 (Mus caroli; Ryukyu mouse) WGS FMAL02029056.1

CCTCCCTCTTTACCTGCTGGCTTCTGTCCACCACTGCCAAGGTCACTATCCATTCACCGCTCCAAGTGGTTGAAGGACAAAACGTTTTTCTACGAGTTGACAATCTTCCAGAGAATCTTCTAGCTTTTGCCTGGTACAGAGGACTGACAAATTGGAGGGTCGCAATTGCACTGCATTTAGTGGAGTATAATGCAAGTATGACAAAGCCTGAGCACAGTGATAGAGAGATATTGCACAGCAACGGGTCCCTGTGGATCCAAAATGTTACTCAAGAGGACACAGGATATTACACTTTTCAAACCATAAGTAAACATGGAAAACTGGTATCAAATACATCCACATTTTTTCAGGTGCGCT

>Mca_Psg21N1 (Mus caroli; Ryukyu mouse) WGS FMAL02029056.1

CCTCCCTTTTGACCTGCTGGCTTCTGTCCACCACTGCTAGTGTCACCATCCAGTCACCACAACACGTAGTTGAAGGAGAGAATATTCTTCTACAAGTTGACAATCTGCCAGAGAATCTTCTAGCTTTTGCCTGGTACAGAGGACTGACAAATTGGAGGCTCACAATTGCTCTGCATTTCCTGGAGTATAGCACAAGTATGACAGGGCCTGAGCACAGGGATAGAGAGATATTGTACAGCAACGGGTCCCTGTGGATCCAAAATGTCACTCAGGAGGACACAGGATATTACACTTTTCAAACTATAAGTAAACATGGAGAACTGGAATCAAATACATCCACATTTCTTCAGGTATACTGTAA

>Mca_Psg22N1 (Mus caroli; Ryukyu mouse) WGS FMAL02019672.1

CCTCCCTCTTAACCTGCTGGCTCTTGCCCATCACTGCCGGAGTCACCATTGAATCCGTACCACCCAAATTAGTTGAAGGAGAAAATGTTCTTCTACGAGTGGACAATCTGCCAGAGAATCTTCGAGTCTTTGTCTGGTATAGAGGGGTGACAGACATGAGCCTCNGAATTGCATTGTATTCACTTGACTATAGCACAAGTGTGACAGGACCTGAGCACAGTGGTAGAGAGACATTGTACAGCAACGGGTCCCTGTGGATCCAAAATGTCACCCNGGAAGACACAGGATATTACACTCTTCAAACCATAAGTAAAAATGGAGAACTGGTATCAAATACATCCACATTCCTTCATGTGTACT

>Mca_Psg24N1 (Mus caroli; Ryukyu mouse) WGS FMAL02019650.1

CCTCCCTCTTAACCTGCTGGCTCCTGCCCATCACTACTCAAGTTGACATCGAATCCTTACCGCCCCAAGTGGTTGAAGGAGAAAATGTTCTTCTACGGGTTGACAATCTGCCAGAGAATCTTCTAGGCTTTGTCTGGTATAAAGGTGTGACAGACATGAGCCTCGGAATTGCACTGTATTCACTGACCTATAGCAGAGGTGTGACAGGACCTGTGCACAGTGGTAGAGAGATATTGTACCGAAATGGGTCCCTGTGGATTCAAAATGTCACCCAGGAGGACACAGGATTCTACACCCTACGAACCATAAGTAAACGTGGAGAAATTATATCAAATACATCCATGCATCTTCATGTGTACT

>Mca_Psg25N1 (Mus caroli; Ryukyu mouse) WGS FMAL02028777.1

CCTCCATCTTAACCTACTGGCTCCTGCCCACCAGTGCCAGAGTCATCATCCATTCTTTACCACTCCAAGTGGTTGAAGGAGAAAATGTTCTTCTACATGTTTACAATCTGCCAGAGAATCTTCTAGCCCTTGTCTGGTACAGAGGGTTGCTAAATTTGAAACTTGGAATTGCACTGTATTCACTGCAATATAATGTAAGTGTGACTGGGCCTGAGCACAGCGGTAGAGAGACATTGCATAGAAATGGGTCTCTGTGGATCCAAAATGTCACCCAGGAGGACACAGGATATTACACTCTTCGTACCATAAGTAAAAATGGAGAACTGGTATCAAATACATCCACATTTCTTCATGTGTACT

>Mca_Psg27N1 (Mus caroli; Ryukyu mouse) WGS FMAL02030370.1

CCTCCCTCTTTACCTGCTGGCTCCTCTCCACTACTGCCAGAGTCATCATCCATTCACCACTCCAAGTGGTTGAAGGAGAAAGTGTTCTTCTACGAGTTGACAATCTGCCAGAGAATCTTCTAGCCTTTTCCTGGTACAGAGGACTGACAAATTGGAGGCTTGCAATTGCACTGCATTTACTGGACTATAACACGAGTATGACAGGGCCTGAGCACAGTGATAGAGAGATATTGTACAGCAATGGATCCCTATGGATCCAAAATGTCACCCAGGAGGACACAGGATATTACACTCTTCAAACCATAAGTAAACATGGAGAACTGGTATCAAATACATCCACATTTCTTCAGGTGTACT

>Mca_Psg28N1 (Mus caroli; Ryukyu mouse) WGS FMAL02030476.1

CCTCCCTCTTAACCTGCTGGCTCCTGCCCACCACTGCCAGAGTCACCATTGAATCCTTACCACCCCAAGTGGTTGAAGGAGAAAATGTTCTTCTACGTGTTGATAATATGCCAGAGAATCTTCTAGTGTTTGGCTGGTACAGAGGAATGACAAATATGAGGCATGCAATTGCACTACATTCACTGTACTATAGTGTAACGGCGAAGGGGCTGAAGCACAGCGGCAGAGAGACATTATACATCAACGGGACCCTGTGGATCCAAAATGTCACACAGGAGGACACAGGATATTACACTTTTCAAACCATAAGTAAACAACGGGAAATGGTATCAAATACATCCCTGTACTTGCACGTGTACT

>Mca_Psg29N1 (Mus caroli; Ryukyu mouse) WGS FMAL02030010.1

CCTCCCTCTTAACCTGCTGGTATCTGTCTACCACTTCCAAAGTCACCATTGAATTATTGCCACCTCAAGTGGTTGAAGGAGAAGATGTTCTTTTCCTCGTCAATAATCTGCCAGAGAATCTTACAGCCTTTGCCTGGTTTAAAGGGAGGACAAATAGGAAACATGGAATTGCACTGTATGCAGTGGCCTCTGACATATATGTACACAGCGATAGAGAGACATTGTACAACAATGGATCCCTGATGATCCACAATATTACCCAGAAGGACAGAGATTATTACACCCTACGAACCTTCAATAAACATGCAGAAACTGTGTCAACAACATTCACATTCCTCCATGTGAACC

>Mca_Psg30N1 (Mus caroli; Ryukyu mouse) WGS FMAL02019659.1

CCTCCTTTTTAACTTGCTGGCAGCTGCCTACCACTGCACAAATAACCATGGAATTAGTGCCACCCCATGTGATTGAAGGAGAAAATGTTCTCATACGTATCGACAATCTGACAGAGAATCTTATAACCTTAGCCTGGTTCAGAGGAATGAGGATTAAGAGCCCTCAAATTGGACAATATACACTGGCCACTAATGTTACTGTGCTGGGGCCTGGACACAGTGGTAGAGAGACTTTGTACAGCAATGGATCCCTGCAGATCAACAATGTCACCCAGGAGGACATAGGATTCTACAGCCTACGAATCATAAATAGACATGCAAAAATTGTGTCAATAATATCCATATACCTCAACGTGTACT

>Mca_Psg31N1 (Mus caroli; Ryukyu mouse) WGS FMAL02019660.1

CCTCATTTTTAACCTGTTGTCACCTGCCTGCCACTGCCCAAATAACTATTGAATTAGTGCCACCACATGTGATTGAAGGAGAAAATGTTCTCATACGTGTCAACAATATGCCAGAGAATCTTACAACCTTAGCCTGGTTCAGAGGAATGAGGATTAAGAGCCCTCAAATTGGACAATATACACTGGCCACTAATGTTACTGTGCTGGGGCCTGGTCACAGTGGTAGAGAAACTTTGTACAGCAATGGATCCCTGCAGATCTACAATGTCACCCAGGAGGATGTAGGATTCTACAGCCTACGAATCATGAATAAACATGCACAAATTGTGTCAATAACATCCATATACCTCAACGTGTACT

>Mca_Psg32N1 (Mus caroli; Ryukyu mouse) WGS FMAL02019659.1

CTTTTCTTTTAACCTTTTGGTTCCTGCCCACCACTGTCCAAGTCACCATTGAATTAGTGCCACCACAAGTGGCTGAAGGAGAAAATGTCCTTATTATTGTTTACAGTCTGCCAGAGGATCTTACAGCCATAGCCTGGTTCAAAGGAGTGACAAATATGAACCTCAGAATTGCATTGTATGCACTGGCCTCTAACACCAGCGTGAAAGGGCCCGAACACAGTGGTAGAGAGACAGTGTTCAGCAATGGATCACTGCTGCTTCACAATGTCACCCAGAAGGACACAGGATTCTATACTATACGAACCTTAAATAGACATGGAAAAATTGTATCCACAACATCCATATACCTCCACGTGTACA

>Mco_Ceacam9N (Mastomys coucha; southern multimammate mouse) WGS VSBT01000021.1

CCTCCCTCTTAACCTGCTGGAATGCACCCGCCGCTGCCGAGCTCACTATTGAATTAGTGCCACCCATGGTTGTGGAAGGTGGAAACTCCGTTCTGTTTGTACATGAAATGCCACTGAATGTCCAGGCGTTTTACTGGTACAAACAGAGAGATTCGACGAAGAGCTATGAAGTTGCACGGTACTTAACACCCACTAACGAAAGTTCCAAGATGCCTCAGCACAGTGATAGGAAAACCGTATTCTACAGTGGATCCCTGCTGATCAGAAATGTCACCCAGGCCGACAGTGGAGTCTACACCTTACTAACATTCAACACAGAAATGGAAAGCGAATTAACACACGTACATCTGGAAGTACGC

>Mco_Ceacam11N1_P (Mastomys coucha; southern multimammate mouse) WGS VSBT01000021.1

TCTCCCTTTTAATCTGTTGGCTGCTTTCCACTACTTCCCAGTTCACCATTAAATCAGTGTCTCCCATTGCTGTTGAAATGGAAAATTTTCCTCTGTTTGTGTATAACCTGAAGAATGTTAAAGCCTTTTCCTGGTACACAGGAGTTACCATGCTCAAGGACTATGAAATTGCAAGTCATGTGATAGCTACCAATTTTACTGTAGTGGGGCTTGCACACAGTGGTAGAGAGACAGTGCTCAACAATGGCTCTCTGCTGATCAAGAGTGTCACCAGAAAAGATTCAGGATACTGCACTCTATGATCACTTGATGCAACCTCAAGACCGGAAGTAATAAGTGCAGAATTCTTTGTACACA

>Mco_Ceacam12N1 (Mastomys coucha; southern multimammate mouse) WGS VSBT01000021.1

TCTCCCTTTTAATCTGCTGGCTGCTTTCCACTACTGACCAGCTCACTATTGAATCAGTGCCTCCCATTGCTGTTGAAGGGGAAAATGTTTTGCTATTTGTGCATGACCTGCCAGAGAATGTTCAAGCCCTTTCCTGGTACACAGGAGGTAAACCACTCAAGAGGTTTGAAATTACAAGACATGTGATTGCTACCAATTCTAGTGTGATGGGGCCTGCACACAGTGGTAGAGAGACAATACTCAACAATGGATCTTTGCTGATCAAGAGTGTCACCAGAAAAGACTCAGGATACTACACTCTGCAAATACGTGATACGACCTCAAGACGTAAAATAACACGTGCAGAATTCTTTGTACAGG

>Mco_Ceacam13N1 (Mastomys coucha; southern multimammate mouse) WGS VSBT01000021.1

TCTCCCTTTTAACCTGCTGGCTGCTTCCCACCACTTCCAAGCTCACCATAAAATCAATGCCTCCCATTGCTGTTGAAGGGGAAAATGTTCTTCTGTTTGTGCATAACCTGCCGAAGAATGTTAAAGCCTTTTCCTGGTACACAGGACCTGCACCATTCAAGTGTTGTGAAATTGCAAGTCATGTGATAGCAACCAATTTTACTGTGGTAGGACTTGCACACAGTGGTAGAGAGACAGTACTCAACAATGGATCTCTGCTGATCAAAAGTGTCACCAGAAAAGACTCAGGATACTACACTCTACGAACACTTGACTCAACCTCAAGACCTGAAATAATACATACTGAATTCTTTGTACACA

>Mco_Ceacam14N1 (Mastomys coucha; southern multimammate mouse) WGS VSBT01000021.1

TCTCACTTTTAATCTGCTGGCTGCTTCCCAGTACTTCCCAGCTCACCATTAAATCAGTGCCTCCAATTGCTGTTGAAGGGGAAAATGTTCTTCTGTTTGTGCATAACCTGCCGAAGAATGTTAAAGCCTTTTCCTGGTACACAGGAGTTACAGCTATCAAGAGTTGTGAAATTGCAAGTCATGTGATTGCTACCAAATTTACTGTGGTGGGACCTGCACACAGTGGTAGAGAGACACTATTCAACAATGGATCTTTGCTGATCAATAGTGTCACCAGAAAAGACTCAGGATACTACACTCTACAAATACTTGGTGCAACCTCAAGACGTAAAATAATACGTGCAGAATTCTTTGTGCACA

>Mco_Ceacam15N (Mastomys coucha; southern multimammate mouse) WGS VSBT01000021.1

CCTCACTTTTATACTGCTGGAGCTCACCCACGGTGGCACTGCAAACATCTAAAGAAATGCGCTTCTCGGCTGCTGAAGGGGCAAAGGCTCTTCTCTCTGTTCCTGACCAGGAAGAGGATCTCCTCTCCTTTTCCTGGTACAAAGGGAAGGATGTAAATAAAAATTTTACAATTGCACATTACAAAAAGTCCAGCGATTCACTTCAGCTTGGAAAGAATGTCAGCGGCAGGGAAGAAATCTATAAGGATGGCTCCATGATGCTCCAGGCCGTCACCCAGGAAGACACGGGATTCTACACTTTAGAAACCTTTAAAGCACACGATCAACAGGAGATAACATATGCCCATCTCCAAGTATACA

>Mco_Psg36N1 (Mastomys coucha; southern multimammate mouse) WGS VSBT01000021.1

CCTCCCTCTTAACCTGCTGGCTCCTGCCCACCACTGCCCAAATTGACATCAAATCTTTACCACCCCAAGTGGTTGAGGGAGAAAATGTTCTTCTATGCGTTGAAAATCTGCCAGAGGATCTTATAGCCTTTGTCTGGTACAAAGGGGTGACAGACATGAGCCTCGGAATTGCACTGTATTCACTGACCTACAGCGTAAGTGTGACGGGGCCTGTGCACAGTGGTAGAGAGACACTGTACAGCAACGGGTCCCTGTGGATCCAAAATGTCACCCAGGAGGACACAGGATTCTACACCCTACGAACCATAAGTAAACGTGGAGAAATTGTATCAAACACGTCGATGCACCTTCTCGTGTACT

>Mco_Psg37N1 (Mastomys coucha; southern multimammate mouse) WGS VSBT01000021.1

CCTCCCTCTTAACCTGCTGGCTCCTGTCCACCACTACCAGTGTCACCATCGAATCCTTACCTCCCAAAGTGGTTGAAGGAGAAAATGTTCTTCTACACGTTGACAATCTGCCAGAGAATCTTCTAGTCTTTGCCTGGTACAGAGAAGTGACAAGTATGAAGCTTGTAATTGGACTGTATTTACCAGATTACAGAACAAGTGTAAAGGGGCCTGAGCACAGTGGTAGAGAGATATTGTACAGCAATGGGTCCCTGTGGATCCAAAATGTCACCAGAGAGGACACAGGATATTACACTCTTCGAACCAAAAGCAAACATGGAGAAATTGTATCAAATACATCTGTGTTCCTTCAGGTGTACT

>Mco_Psg38N1 (Mastomys coucha; southern multimammate mouse) WGS VSBT01000021.1

CCTCCCTTTTAACCTGCTGCCTCCTGACCACTGCCCAGGTCACCATTGAATCGGTGCCATTCAATGTGGTTGAAGGAGAAAACGTTCTTCTTCTTGTCGACAATCTGCCAGAGAATCTTCTAGCCTTAGCATGGTACAGGGGGCTGAGGAAAATCATTGTATACACACTGAACACTAAAGTAAGTGTGATGGGGCCTATTAACAGTGGCAGAGAGACAGTGTCCAGCAACGGGTCCCTGTGGATCCACAATGTCACCCAGAAGGACACAGGATTCTACACCTTACGAACCGTAAATAGACGTGGAGAAATTGTGTCAACCACATCCACGTACCTCTACGTGTACT

>Mco_Psg40N1_P (Mastomys coucha; southern multimammate mouse) WGS VSBT01000021.1

TCCCTTTTAACCTACTGGTACTTTTCTACCACTTCCCAAGTCACCATTGAATTAGTGCCACCCCAAGTGGTTGAAGGAGAAGATGTCCTATTCCTTCTCCACAAACTGCCAGAAATTCTTATGTCCTTAGGCTGGTTCAAAGGGATGACAGTTATAAAATGTGGAATTGCACTGTATGCAACAAACACTAAAGTATGACAGGGCCCATGTACAGTGCTAGAGAGACCTTGTACAGAAACGGGTCCCTGTTGATCCACAATGTCACCCAAGGACACAGGATTCTATAACTTATGAACCTTAAATAGACTTGGAGATATTGTGTCAACATCCACGTTCCTCTA

>Mco_Psg41N1 (Mastomys coucha; southern multimammate mouse) WGS VSBT01000021.1

CTTCCCTTTTCACCTGCTTGCTCCTGCCCACCACTGCCCAGGTCACCATTGAATCAGTGCCACCCCACGTGGTTGAAGGAGAAAATGTTCTTTTTCTCGTACACAATTTGCCAGAGAATCTTATAGCCTTAGTCTGGTTAAGAAGACTGGGGAAAATGAACCATGCAATTGGCCTATATGCAATGAACACTAAAATAAGTGTGATGGGGCCCATGAACAGCGGCAGAGAGACAGTGTCCAGCAACGGGTCCCTGTGGATCCGCAATGTCACCCAGAAGGACACAGGATTCTACATCTTACAAACCATAAATAGACGTGGAGAAATTGTCTCAAGAACACCCATGTACCTCTACGTGTACA

>Mco_Psg42N1 (Mastomys coucha; southern multimammate mouse) WGS VSBT01000021.1

CTTCCCTTTTAACATGCTGGCACCTGTCTACCACTTCCAAAGTCACCATTGAATTATTGCCACCACACGTGGTTGAAGGGGAAGATGTTCTTTTCCTTGTCCAAAATCTGCCAGAGGATCTTTCAGCCTTTGCCTGGTTTAAAGGGAGGACAAATAAGAAACATGGAATTGCACTATATGCAGTGGCCTCTGACATACATGTACACAGCGATAAAGAGACATTGTATAGCAATGGATCCCTGATGATCCACAACATCACCCAGAAGGACAGAGGTTACTATACCCTACGAACCTTCAATAAACATTCAGAAACTGTATCAACAACATCCACATTCCTCCATGTGAACC

>Mco_Psg43N1 (Mastomys coucha; southern multimammate mouse) WGS VSBT01000021.1

CTTTTCTTTTAATCTCCTGGTTCCCACCCAACACTGTCCAAGTCACCATTGAATTAGTGCCACCCCAAGTGGCTGAAGGAGAAAATGTCCTTCTTCTTGTTTACAATCTGCCAGAGAATCTTATAGCCATAGCCTGGTTCAAAGGAGTGACAAATATGAACCTCGGAATTGTGTTGTATGCACTGGCCTCTAACATTAGTGTGACAGGGCCTGAACACAGTGGTAGAGAGACAATGTACAGGAATGGATCCCTGATTCTTCACAATGTCACCCAGAAGGACACAGGATTCTATACTCTACGGACCTTTAATAGACATGGAAAAATTGTATCAACAACATCCATTTACCTCCATGTGTACA

>Mco_Psg46N1 (Mastomys coucha; southern multimammate mouse) WGS VSBT01000021.1

CCTCCCTTTTAACATGCTGGCACCTGTCTACCACAGCCAAGATCACCATTGAATTATTGCCACCCCACGTGGTTGAAGGAGAAGATGTCCTTTTTCTTGTCCACAATCTGCCAGAGAATCTTACAGCCTTTGCCTGGTTTAAAGGGAGGACAAATGAGAAACATGGAATTGCACTATATGCAGTGGTCTCTGATTTACATATACACAGCGATAGAGAGACATTATACAGCAATGGATCCCTGATGATCCACAATATCACCCAGAAGGACAGAGATTATTACACCCTACGAACCTTCAATAAACATGCAGAAACTGTATCAACAACATCCACATTCCTCCATGTGAACC

>Mfo_Ceacam9N (Microtus fortis; reed vole) WGS NMRL01000322.1

CCTTCCTCTTAACCTGCTGGAATGCACCCACCACTGCCCAACTCACTATTGAATTAGTGCCCCCCATGGTTGCTGAAGGTGGAAACTCCGTCCTATTTGTGCATAAAATGCCGCTGAACGTCCAGGCATTTTACTGGTACAAACAGAAAGATGCGACCAAGAGCTACGAAGTTGCACGCTACTTAACACCCGATAACACAACGTCGAAGATGCCTCAACACAGTGGTAGGAAAACGGTATTCTACAGTGGATCCCTGCTGATCAGAAACGTCACCCAGGCTGACAGTGGATTCTACACCTTACTGACGTTCAACACAGAAATGCAAAGTGAACTCACACACGTACATCTGGAAGTATACA

>Mfo_Psg1N1 (Microtus fortis; reed vole) WGS NMRL01000258.1

CCTTCCTTTTAACCTGTTGGCACCTGCCTACCACTGCCCAAGTCACCATCGAATTAGTGCCGCCCCAAGTGGTTGAAGGAGAAAATGTTCTTCTACGTGTTCATAATCTACCAGAGAATCTTCTAGCCTTTGTCTGGCACAAGGGGGTGAGGAATATGAGCCTTGGAATTGCACTACATTCATTGGACAAGGGTTTAAGTGTGACAGGGCCCATACACAGTGGTAGAGAGACAGTGTACAGCAATGGATCCCTGCAGATCTACAATGTCACCCAGAAGGACACAGGATTCTACACCTTTCGAACCATAAATGGACAAGTAGGAGTTGCATCAATAACAACCACGTACCTTCATGTGTACA

>Mfo_Psg2N1 (Microtus fortis; reed vole) WGS NMRL01000244.1

CCTACCTTTTAACCTGTTGGCACCTGCCCACCACTGTCCAAGTCATTGTTGATTTAGTGCCACCCCATGTTGTTGAAGGAGAAGATGTCCTTCTTCGTGTCCGCAATCTCCCGGAAGATCTTGTAGCCTTTGTCTGGCACAAAGGGGCGACAAAGATGGACCTCGGAATTGTACTTTATTCACTGACCACTAATTTAAAAATCACAGGGCCTGGACATAGTGGTAGAGAGATTGTGTACAGAAATGGATCTCTGCACCTCCAAAATGTCACCCAGAAGGACACAGGATTCTACACGCTACGATCCTTAAATAGGCATAAAGGAATTGTATCAACAACATCTATATACCTGCATGTATACT

>Mfo_Psg3N1 (Microtus fortis; reed vole) WGS NMRL01000244.1

CCTCCCTTTTCTCCTTCTGGCATCTCCCCACTACTGCTCAAGTGTCCACTGAATCAGTGCCACCCCTAGTGGCTGAAGGTGATAATGTCCTTATCCTTGTCAACAATCTGCCAGAGAATCTTTTAGCCTTAGCCTGGTTCAAAGGACTAACAAATATGAAGCAAGGAATTGCATTATATGCACTGCACAAAAATGTAAGTGTTACAGGGCCTGTGCACAGTGGCAGAGAGACAATATATCACAATGGATCCTTGTTGATTGAAAAACTCACCCAGAAGGACACAGGATTCTACACCTTTCGAGCCTATAATAGACGTGGAAGAATTGTATCAAGCACATCCACCTACCTCCATGTGCAAG

>Mfo_Psg4N1 (Microtus fortis; reed vole) WGS NMRL01000244.1

CCTCACTTTTAGGCTGCTGCCTATCCACCACTGACTATATCACCATTAAATCTGTCCAACCCCATGTGGCCAGTGGAGAAGACGTCCTTCTTCATGTCCACAATCTGCCAGAAGATATTCTAGCCTTCGCCTGGTTCAAAGGGGCGACAAGCATGAAACATGGAATTGCGGTATATGCACTGCACAAAAATTTAAGTGCGACAGGGCCTGCACATAGTGGTAGAGAGACAGTGTACCACAATGGATCCCTGCTGCTCCGAAGTGTCACCGAGAAGGACACAGGATCCTATACCCTAAGAACCTTTGATAGACAAGGAGAGATTGTATCAACAACAACCATGCGCCTCTATGTGTACC

>Mfo_Psg5N1 (Microtus fortis; reed vole) WGS NMRL01000453.1

TCTCCCTTTTAAGCTCCTGGCATCTGTCCACAAATGCCCATATGACTATTGAAAAAGTGCCAGCCCTAGCTGCTGAAGGAGATGACATCTTTTTCCATGTCAATGATCTGCCAGAGAATACTACAACCATAGCCTGGTTCAAAGGTCTAAGAAATACGACAAAAGGAATTGGAGCATATGCACCGCTCTTAAATTTGAGTAGGCCAGGTCCTATGTACAGTGGTAGGGAGACAATGTTTCGCAATGGATCCCTGCTGATAAAAAATGTCAACCCAACGGACACTGGATTCTATACCCTACGAACTTATAATAATCATGGAACTAGGACATCAATAACATCCGCGTACCTCAAAGTGCACG

>Mfo_Psg6N1 (Microtus fortis; reed vole) WGS NMRL01000453.1

TCTCCCTTTTAACCTCCTGGTACCTGTCCACTGCTGTCCATATAACTACTGAGTCAAGCCGAGTGGTTGAAGGAGAAAACATCCTTTTCCTTGTGCATGATCTGCCAGATAATACTAAATCCTTAGTCTGGTTCAAAGCTCTAAGAAACGCCACAGAAGAAATTGCAGCATATGCACTGCCCTACAATTTAAGTAGGCCAGGTCCTCTGTACAGTGGTAGAGAGACAATATATCGCAATGGATCCCTGATGATAGAAAATATCAACCTCAAGGACACAGGATTCTATATTCTACAAACCTATAACAGACGTAAAAAAGTCATATCAACAACAACCATGTACCTCCAAGTGAATG

>Mfo_Psg7N1 (Microtus fortis; reed vole) WGS NMRL01000258.1

CCTCCTTTTTAGCTCTCTGGCACCTGTCCATCACTGCCTCAGTGACCATTGAATCAGTGCCACCCCTGATGGCCGAAGGAGATAACATTCTTTTTCTTGTCGACAATCTGCCGGAGAAGACTGTAACCTTAGTCTGGTTCAAAGGGCTAACAAATATGAAAGCTGTGATTGCAATATATGGACGGCACATCAATTTAAGTGCATCTGGGCCTTTGCACAGCGGTAGAGAGACAATATATTACAACGGATCCCTGCTGATTAAAAATGTTACCCAGAAAGACACAGGATTCTATACCCTACGAAGCTATGATAAGTATTTAAACATCATATCAACAACATACACATACGTCCATGTTCACG

>Mfo_Psg8N1 (Microtus fortis; reed vole) WGS NMRL01000394.1

CCTTCCTTTTAACCTGTTGGCACCTGCCCACCACTGCCCAAGTCACCATCGAATTAGTGCCGCCCCAAGTGGTTGAAGGAGAAAACGTTCTTCTACATGTTCATAATCTACCAGAGAATCTTCTAGCCTTTGTCTGGCACAAAGGGGTGAGGAATATGAGCCTTGGAATCGCACTATATTCAGTGGCCAAGGGTTTAAGTGTGACAGGGCCCATACACAGTGGTAGAGAGACAGTGTACAGCAATGGATCCCTTCAGATCCACAATGTCACCCAGAAGGACACAGGATTCTACACCTTTCGAACCATAAATGGACAAGTAGGAGTCTCATCAATAACAACCACGTACCTTCACGTGTACA

>Mfo_Psg9N1_P (Microtus fortis; reed vole) WGS NMRL01000394.1

CCTTCCTTTTAATCTGGTGTCATTCCCGCCACTGCTGAAGTCACCATTGAATCAGTGCCGCCCAATGTGTTCGAAGGAGACAGTTGTCCTTCTATATGTCCACAGTCTGCCAGAGAATCTGCTAGCCTTTGCTTGGTTCAAAGGGCTAACAAATATGAAACGCAGAATTGTACTCTATGAACTGAACAACAATTTAAGTTTGCCGGGGCCTGAATACAGCGGTAGAGAGACAGTCTATCGCAATGGATCCCTGTGGATTTCCAATGTCACCCATGTGGACACAGGATTCTATACCCTACGAACCATCGGTAGACATTCAAGAGTTGTGTCACTAACAACCATCCACCTCCCTGTGTACA

>Mfo_Psg10N1 (Microtus fortis; reed vole) WGS NMRL01000552.1

CCTCCATTTTAGGCTTCTGGCACATGTCCACTACTGCCCATGAGACCACTGAGTCACTGCCACGCCAAGTCGTTGTAGGAGACAACGTTCTTTTGTTTGTCCACAATCTGCCAAAGAATCTTATAGCCTTTGCCTGGTTCAAAGGGCTAACAAATATGACTCAAGGAATTGCAGTATATACACTGCACAACAATTTAAGTGCACCAGGGCCTGTGCACAGTAGTAGAGAGACAGTTTATAGCAATGGATCCCTGCTGATAGAAAATGTCACCCAGAAAGACACAGGAATCTATACCCTACGAACCTATAATAGAAGAGGAAAAATTGTATCAACAACATCTATGTACCTCCATGTGCACG

>Mfo_Psg12N1 (Microtus fortis; reed vole) WGS NMRL01000258.1

CCTCCCTTTTAATCTTTGGGCACCTGCCCACCACTGCCCGTGTGATCACTGAATTAGTACCACCCGAAGTGGCTGAAGGAGAAAACGTTCTTTTTATTGTCCACAATCTGCCAGAGAATGTTAAATCCTTTGCCTGGTTCAAAGGGCTAAAAACCGAGAAACAAGGAATTGCAACGTATAGACAGAGCAAGAATTTAGTTACAAATGGGCCTATGCACAGTGGCAGAGAAACCATATACCACAATGGATCCCTGCTTCTCCAAAATGTCTCCCATAATGACACAGGATTCTTTACCCTACAAACCTATGATACACATGAAAAAATCCTATCAACAACTTCTGTATATCTCCATGTGCATG

>Mfo_Psg13N1 (Microtus fortis; reed vole) WGS NMRL01000258.1

CCTTCCTTTTAACCTTCTGGCTCCTCTCCACCACTGCCCATGAGACCACTGTGTCAGTGCCACCCATAGTGTCTGAAGGAGATGACGTCCTGTTCCTTGTCCACAATCTGCCAGGGGAAATCGAATCCTTAGCCTGGTTCAAAGGGCTAGGAGATGAGGCAGAAGAAATTGCAACATATGCACTGCACAGAGGTTTAAGCAGGCCAGGTCCTGCGCACAGCAGTAGAGAGACAATATATCACAACGGATCCATGCTGTTTGAGAAGGTCAACCTGAAGGACACAGAATTCTATACCCTACGAACCTATAATAGAAGTGGAAAAATCATATCAACAGCAAACGTGTACCTCAATGTGTATG

>Mfo_Psg14N1_P (Microtus fortis; reed vole) WGS NMRL01000688.1

CCTCCCTCTTAACCTTCTGGCTCCTCTCCACCACCGCCCGTAAGACCACTGTGTCAGTGCCACCCCAAGTGGCTGAAGGAGATGATGTCCTGTTCCTTGTCCACAATCTGCCAAAGGACATTAAATCCTTAGCCTGGTTCAAAGGGCAAGGAAACACAACCAAAAAAATTGCAACATAAACACTGCACAATGATTTAAGTAGGCGAGGTCTTGCGTACAGCAATAGAGAGACAATATATCACAACGGATCTATGCTGTTTGAGAAGGTCACCCTGAAGGACTCGGGATTCTATACACTACAAACCTATAACAGACATGGAAAAAATGTATCAACAACATCCGTGATCCTCGATGTGAAAG

>Mfo_Psg15N1 (Microtus fortis; reed vole) 7.5.21 WGS NMRL01000244.1

CCTTCCTTTTAACCTTCTGGCACCTGTCCACCACTGCCTTTGTGACTACTGTATCAGTGCCATCCCAAGTGGCTGAAGGAAATGACGTCCTATTCCTTGTCCACAATCTGCCAGAGAAATTTAAAACCATTGCCTGGTTCAGAGGGTCCTCAAATACGATTGCAAAAAATGAACTGCCTGACAATTTAAATAGGCCAGGTCTTGCACACAGCGGCAGAGAGACAATATTTCACAATGGATCCATGCTGCTTAAAAAGGTCAACCTGAAGGACACAGGCTTCTATACCGTACGAACCTATAATATACATGGAAATGTCATATCAACAACATACACATACCTCAACGTGTATG

>Mfo_Psg16N1_P (Microtus fortis; reed vole) WGS NMRL01000244.1

CTTCCCTTTTAACCTGCTGGCACCTGTACAACACTGTCAAAATCACAATTGACTCAGAGCCACTCCAAGTGGTTGAAGGAGAAAGCGTCCTTCTACAGGTCAACAATCTGCCACAGAATCTTCGAAATTTTGCCTGGTTCAAAGGGGTGACAAATATAAACTTCAGAATTGCATTATATTCACTGACCAGTAATCTATGTGTGATGGGGCCTGAAAATAGTGGTAGAGAAGCTGTGTACAGCAATGGATCCCTGTTTCTCAAAAATGTCTCCCAGAAGGACACAGGATTTTATATACTACGAACAGTAATTGGAGGTGGAAAAATTGTATCTACAACCAAATAACTCCACGTGTATG

>Mfo_Psg19N1 (Microtus fortis; reed vole) WGS NMRL01000453.1

GCCTCCATTTTAACCTCCTGGCACCTGTCTTCCACTGCCAATGTGACCATTGAATTACTGCCAGCTCCAGTGGCCGAAGGAGATAACGTCCTTTTCCAAGTCCACAATCTGCCAGATGAAATAAAAGCTGTAGCCTGGTTCAAAGGGCTGGGAAATAAGAAACAACAAATTGCTCTGTATGTACTGGACAAAAATTTAAGTAAGCCAGGTCCTATGCACAGCGGGAGAGAGACAATATATCACAATGGATCCCTGCTTCTTGAAAAGGTCACCCAGAAGGATGCAGGATTCTATACCCTACGAACCTATGATAGAGGTGGAAAATTTGTATCAACAATAACCATGTACCTCTACGTGCAAG

>Mgl_Ceacam9N (Myodes glareolus; Bank vole) WGS MULK01040603.1

CCTTCCTCTTAACCTGCTGGAATGCACCCACCACTGCCGAACTCACTATTGAATTAGTGCCCCCCATGGTTGCTGAAGGTGGAAACTCCGTCCTATTTGTGCATAAAATGCCGCTGAACGTCCAGGCATTTTACTGGTACAAACAGAAAGATTCGACCAAGAGCTACGAAGTTGCACGCTACTTAACACCCGATAACACAACGTCGAAGATGCCTCAACACAGCGGTAGGAGAACGGTATTCTACAGTGGATCCCTGCTGATCAGAAACGTCACCCAGGCTGACAGTGGATTCTACACCTTACTAACGTTCAACACAGAAATGCAAAGTGAACTCACACACGTACATCTGGAAGTATACC

>Mgl_Ceacam11lN1_P (Myodes glareolus; Bank vole) WGS MULK01043214.1

CCTCCCTTTTAACCTGCTGGCTGCCTCCCACTATTGCCCAGCTCACCACTGAATCAGGGCCACCCATTTATGCTGAAGGAGATAATGTTCTTATGTTTGTGCACAACATGCCTGAAAATGTTCAAGTCTTTTCCTGGTTCACAGGAGTGATGGTACTCAAGAGCCATGAAATGTCAAGATATGTGGTATCTACCAGCTCATGTATGTTGGAGCCTACACACAGTGGTAGAGAGACAGCAGTCAATAATGGATCTCTCCTGATCAAGATGCCACCAGGAAAGACTCGTGGTTATACGTCCTGCAAACACTTGGTACAAATTTGATACCTGAGATAACATATGTGGATTTTTTTTGTACACT

>Mgl_Psg1N1 (Myodes glareolus; Bank vole) WGS MULK01019059.1

CCTTCCTTTTAACCTGTTGGCACCTGCCCACCACTGCCCAAGTCACCATTGAATTAGTGCCGCCCCAAGTGGTTGAAGGAGAAAATGTTCTTCTACGTGTTCATAATCTACCAGAGAATCTTCTAGCCTTTGTCTGGCACAAAGGGGTGAGGAATATGAGCCTTGGAATTGCACTATATTCACTGGCCAAGGATTTAAGTGTGTCAGGGCCCATACACAGCGGTAGAGAGACAGTGTACAGCAATGGATCCCTGAAGATCTACAATGTCACCCAGAAGGACACAGGACTCTACACCTTACGAACCATAAATGGACAAGTAGGAGTTGGATCAATAACAACCACGTACCTTCACGTGTACA

>Mgl_Psg2N1 (Myodes glareolus; Bank vole) WGS MULK01043214.1

CCTATCTGTTAGCCTGCTGGCATCTGCCTATCACGGTCCAAGTCATTGTTGATTTAGTGCCACCCCATGTTGTTGAAGGAGAAGATGTCCTTCTTCGTGTCCACAATCTTCCAGAGGATCTTGTAGCCTTTGTCTGGCATAAAGGGGTGACAAAAATGAACCTCGGAATTGTACTTTATTCACTGACCACTAATTTAATCATCATGGGGCCTGGACACAGTGGTAGAGAGATTGTGTACAGAAATGGATCTCTGAGCCTCCAAAATGTCACCCAGAATGACACAGGATTCTACACTCTACGATCCTTAAATAGGCATAAAGGAATTGTATCAACAACATCTATATACCTGCATGTATACT

>Mgl_Psg3N1 (Myodes glareolus; Bank vole) WGS MULK01043214.1

CCTTCCTTTTCTCCTTCTGGCATCTCCCCACTATTGCTCAACTGTCGACTGAATCAGTGCCACCCCTAGTGGCTGAAGGAGATAACGTCCTTATCCTTGTCAACACTCTGCCAGAGAATCTTTTAGCCTTAGCCTGGTTCAAGGGGCTAACAGATATGAAACAAGGAATTGCATTATATGCACTGCACAAAAATGTAAGTGCTACAGGGCCTGTGCACAGTGGCAGAGAGACAATATATCACAATGGATCCTTGTTGATTGAAAAACTCACCCTGAAGGACACAGGATTCTACACCTTTCGAGCCTATAATAGACGAGGAAGAGTTGTAGCAACCACATCCACCTACCTCCATGTGCAAG

>Mgl_Psg4N1 (Myodes glareolus; Bank vole) WGS MULK01019059.1

CCACACTTTTAGGCTGCTGCCTATCCACCACTGACTATATCACCGTTAAATCTGTCCCACCCGATGTGGCCAGTGGAGAAGATGTCCTTCTCCATGTCCACAATCTGCCAGAGGATCTTCTAGCCTTCGCCTGGTTCAAAGGGGCAACAAGCATGAAGCATGGAATTGCAGTATATGCGCTGAACAAAAATTTAAGTGCGACAGGGCCTGCACATAGTGGTAGAGAGACAGTGTACCACAATGGATCCATGCTGCTCCAAAGTGTCACCGAGAAGGACACAGGATTCTATACCCTACGAACCTTAGACAGACATGGAGAGATTGTGTCAACAACAACCATGCACCTCTATGTGTACC

>Mgl_Psg5N1 (Myodes glareolus; Bank vole) WGS CADCXP010049679.1

TCTCCCTTTTAAGCTCCTGGCATCTGTCCACAAATGCCCATATGACTATTGAAAAAGTGCCAACCCTAGTTGCTGAAGGAGATGATGTCCTTTTCCATGTCAATGATCTGCCAGAGGATATTACAACCATAGCCTGGTTCAAAGGTCTAAGAAATACAACACAAGGAATTGGAGCATATGCACCGCACTTAAATTTGAGTAGGCCAGGTCCTATGTACAGTGGTAGAGAGACAATATATCGCAATGGATCCCTGCTGATAAAAAATGTCAACCCGATGGACACTGGATTCTATACCCTACGAACTTATAGTCATGGAACTATGATATCAATAACATCCGCGTACCTCCAAGTGTACG

>Mgl_Psg6N1 (Myodes glareolus; Bank vole) WGS MULK01047579.1

TCTCCCTTTTAACCTCCTGGCACCTGTCCACCACTGTCCATATAACTACTGACTCAGTGACAACCCGAGTGGTGGAAGGAGAAAACGTCCTTTTCCTTGTGCATGATCTGCCAGTTAAAACTAAATCCTTAGTCTGGTTCAAAGCTCTAAGAAATATGACAGAAGAAATTGCAGCATATGCACTGCCCTACAATTTAAGTAGGCCAGGTCCTCTGTACAGTGGTCGAGAGACAATATATCACAATGGATCCCTGATGATAGAAAATGTCACCCTCAAGGACACAGGATTCTATATCCTACGAACCTATAACAGACGTAAAAAAATCATATCAACAACAACCATGTACCTCCAAGTGAATG

>Mgl_Psg7N1 (Myodes glareolus; Bank vole) WGS MULK01019059.1

CCTCCCTTTTAACCCTCTGGCACCTGCCCATCACTGCCTCTGTGACCATTGAATCAGTGCCACCCCTGATGGCCGAAGGAGATAACATTCTTTTTCTTGTTGACAATCTGCCGGAGAAGACTGTAACCTTAGTCTGGTTCAAAGGACTAAAAAATATGAAAGCTGTAATTGCAATATATGGACGGCACATCAACTTAAGTGCATCTGGGCCTCTGCACAGTGGTAGAGAGACAATATATTACAACGGATCCCTGCTGATTAAAAAGGTTACCAAGAAAGACACAGGATTCTATACCCTACGAAGCTATGATAAGTATTTAAACATCATATCAACAACATCCACATACATCCATGTTTACG

>Mgl_Psg8N1 (Myodes glareolus; Bank vole) WGS MULK01041290.1

CCTTCCTTTTAACCTGTTGGCACCTGCCCACCACTGCCCAAGTCACCATTGAATTAGTGCCGCCCCAAGTGGTTGAAGGAGAAAATGTTCTTCTACGTGTTCATAATCTACCAGAGAATCTTCTAGCCTTTGTCTGGCACAAAGGGGTGAGGAATATGAGCCTTGGAATTGCACTATATTCACTGGCCAAGGATTTAAGTGTGTCAGGGCCCATACACAGCGGTAGAGAGACAGTGTACAGCAACGGATCCCTGAAGATCTACAATGTCACCCAGAAGGACACAGGATTATACACCTTTCGAACCATAAATGGACAAGTAGGAGTCTCATCAAAAACAACCACGTACCTTCATGTGTATA

>Mgl_Psg9N1 (Myodes glareolus; Bank vole) WGS MULK01041290.1

CCTTGCTTTTAATCTGCTGTCATTCACTGGTCACCGCTGAAGTCATCATTGAATCAGTGCCGCCCAGTGTGTTTGAAGGAGACAATGTCCTTCTATATGTCCACAGTCTGCCAGAGAACCTGCTAGCTTTTGCTTGGTTCAAAGGGCTAACAAATATGAAACGCAGAATTGTACTCTACAAACTGAACAACAATTTAAGTTTGCCGGGGCCTGAATACAGCGGTAGAGAGACAGTTTACCGCAATGGATCCCTGTGGATTTCCAATGTCACCCACGTGGACACAGGATTCTATACCCTAAGAACCATAAGTAGACATTCAAGAATTGTGTCAATAGCAACCATCCACCTCCCTGTGTACA

>Mgl_Psg10N1 (Myodes glareolus; Bank vole) WGS MULK01016118.1

CCTCTATTTTACGTTTCTGGCACATGTCCACAACTGCCCATGAGATCACCGAGTCACTGCCACACCAAGTGGTTGAAGGAGAAAACGTCCTTTTGCTTGTCCACAATCTTCCAGAGAATCTTATAGCCTTTGCCTGGTTCAAAGGGCTAACAAATATGACACAAGGAATTGCAGTATATACAATGCATAATAATTTAAGTGCACCAGGGCCTATGCACAGTAGTAGAGAGACAGTATATAGCAATGGATCACTGCTGATAGAAAATGTCACCCAGAAAGACACAGGAATCTATACGCTACGAACCTATAATAGAAGTGGAAAAATTGCATCAACAACATCTATGTACCTCCACGTGCATG

>Mgl_Psg11N1 (Myodes glareolus; Bank vole) WGS MULK01041290.1

CCTCCCTTTTAACTTACTGGTATCTACCCACCACTGCCCAAGTCATCATTGAATTAGTGCCTCCCAACGTGTTCCAAGGAGAAAATGTCCTTCTAGAGGTCCACAATCTGCCAGAGGATTTTCTAGCCTTTGCTTGGTACAGAGGGGTGACAAGCATGAAACGCGGAATTGCAGTCTATGCCAAAAGAAACAGTTTAAGAGCCCCGGGGCCTGCATACAGTGGTAGACAGACGGTGTACAATGACGGATCACTGCTGCTCCAGCGTGTCATCCTCAAGGACACAGGATTCTACACCTTACGAGTCATAAGTAGACAGGGAGAAATTGTATCAACAACATCCGTGTTCCTCCATGTGCGCA

>Mgl_Psg12N1 (Myodes glareolus; Bank vole) WGS MULK01019059.1

CCTCCCTTTTAACCTTTGGGCACCAGTCCACCACTGCCCATGTGATCACTGAATCAGTACCACCCCAAGTGGCTGAAGGAGAAAACGTTCTTTTCATTGTCCACAATCTGCCAAAGGATGTTAAATCCTTTGGCTGGTTCAAAGGGCTAAAAATCGAGAAACAAGGAATTGCAATGTATAGACGGCGCAAGAATTTAGTTACAAATGGGCCTATGCACAGTGGCAGAGAGACCATATATCGCAATGGATCCCTGCTGCTCGAAAAGGTCTCCCGTAATGACACAGGGTTCTATACCCTACAAACGTATAATAGACATGCAGAAATCCTATCAACAACTGCCGTGTATCTCCATGTGCATG

>Mgl_Psg13N1 (Myodes glareolus; Bank vole) WGS MULK01019059.1

CCTTCCTTTTAACCTTCTGGCACCTGTCCACCACTGCCCATGAGACCACTGTATCAGTGCCACCCATAGTGTCTGAAGGAGATAATGTTCTGTTCCTTGTACACAATCTGCCAGAGGAAATTGAATCCTTAGCCTGGTTCAAAGGGCTAGGAGATGCGGCAGAAGAAATTGCAGCATATACACTGCACAGCGGTTTAAGTAGGCCAGGTCCCGCGCACAGCAGTAGAGAGACAATATATCACAACGGATCCATGCTGTTTGAGAAGGTCAACCTGAAGGACACAGAATTTTATACCCTACGAACCTATAATAGAAGTGGAAAAATCGTATCAACAGCAAACGTGTACCTCAACGTGTATG

>Mgl_Psg14N1 (Myodes glareolus; Bank vole) WGS MULK01019059.1

CCTCCCTTTTAACCTTCTGGCACCTGTCCACCACTGCCCATGTGATCACTGTATCAGTGCCACCCCTAGTGGCTGAAGGAGATGACGTCCTGTTCCTTGTCCACAATCTGTCAGAGGAAATTAAATCCTTAGTCTGGTTCAAAGGGCTAGGAAATGCAACAGGAAAAATTGCAACATATGCACGGCACAGAAATTCAAGAAGGCCAGGTCCTGCGTACAGCAATAGAGAGACAATATATCAAAATGGATCCATGCTGTTTGAGAAGGTCATCCTGAAGGACTCAGGATTCTATACGCTACAAGCCTATGACAGACTTGGAAATATTGTATCAACAACACATGTGACCCTCAATGTGCACG

>Mgl_Psg15N1 (Myodes glareolus; Bank vole) WGS MULK01019059.1

CCTTCCTTTTAACCTTCTGGCATCTGTCCACCACTGCCCTTGTGACCACTGTATCTGTGCCATCCCGAGTGGCTGAAGGAGATGATGTCCTATTCCTTGTCCACAATCTGCCAGAGAAAATTGAAGCCATAGCCTGGTTCAGAGGGCCCTCAGATATGACTGCAATATATGGACTGCCCAACAATTCAAGTAGTCCAGGTCCTGCACACAGCGGCAGAGAGACAATATTTCACAATGGATCCCTGCTCCTTGAAAGGGCCAACCTGAAGGACACAGGCTTCTATACCGTACGAATCTATAATAGACATGGAAATGTCATATCAACAATATACACATACATCAACGTGTATG

>Mgl_Psg16N1 (Myodes glareolus; Bank vole) WGS MULK01043214.1

CCTCCCTTTTAACCTGTTGGCACCTGTCCACCACTGTCAAAATCACAATTGACTCAGTGCCATCCCAAGTGGTTGAAGGAGAAAACGTCCTTCTACGTGCCAACAATCTGCCACAGAATCTTCTAACTTTTGCCTGGTTCAAAGGGGTGACAAATATGAACTTCAGAATTGCATTATATACACTGACCACTAATCTAAGTATGGTGGGGCCTGAAAATAATGGTAGAGAAGTTGTGTACAGCAATGGATCCCTGTGGATCAAAAATGTCTCCCAGAATGACACAGGATTTTATATACTACAAACAGTAAATATAGGTGGAAAAATTGTATCTACAGCAACCACATACCTCCACGTGTACG

>Mgl_Psg17N1 (Myodes glareolus; Bank vole) WGS MULK01019059.1

CCTTCCTTCTAACCTTTTGGCACCTGTCCACCACTGCCCATGTGACCACTGTATCAGTGCCACCCATAGTGTCTGAAGGAGATGATGTCCTGTTCCTTGTCCACAATCTGCCAGAGGAAATTGAATCCTTAGCCTGGTACAAAGGGCTAGGAAACACAACAGAAGAAATTGCAGCATACGCACTGCACAGCGGTTTAAGTAGGCCAGGTCCCGTGCACAGCAGTAGAGAGACAATATATCACAATGGATCCATGCTGTTTGAGAAGGTCATCCTGAAGGACACAGGATTCTATACGCTACAAACTTATAACAGACATGGAAAAATTGTATCAGCAGCATACGTGTACCTCAACGTGCATG

>Mgl_Psg19N1 (Myodes glareolus; Bank vole) WGS MULK01050433.1

CCTCCCTTTCAACCTCCTGGCACCTGTCTACCATGGCCAGTGTCACCATTGAATTACTGCCAACACCAGTAGCTGAAGGAGATAACGTCCTTTTCCTGGTCCGCAATCTGCCAGAGGATATTATAGACGTAGCCTGGTTCAAAGGGCTGAGAAATAAGAAACAACAAATTGCAGTGTACGTACTGCACAAAAATTTAACTATGCCAGGTCCTATACACAGCGGGAGAGAGATAATATATCACAATGGATCCCTGCTTCTTGAAAAGGTCACCCAGAAGGATACAGGATTCTATACCCTACGAACCTATAATAGAGGAAGAAAATTTATATCAACCATGCCCATATACCTCCATGTGCACG

>Mmi_Ceacam9N (Mus minutoides; Southern African pygmy mouse) WGS CACVCL010024911.1

CCTTCCTCTTAACCTGCTGGAATGCACCCGCCGCTGCCGAGCTCACTATTGAGTTAGTGCCACCCATGGTTGCCGAAGGCGGAAACTCCGTTCTGTTTGTGCATGAAATGCCAGTGAATGTCCAGGCGTTTTACTGGTACAAACAGAGAGATTCGACGAAGAGCTACGAAGTCGCACGGTACTTAACACCCACGAACCAAAGTTCGAAGATGCCTCAACACAGCGATAGGAAAACCATATTCTACAGTGGATCCCTGCTGATCAGAAACGTCACCAAGGCTGACAGTGGAGTCTACACCTTACTAACATTTAACACGGAAATGGAAAGCGAATTAACACATGTGCATCTGGAAGTGCATG

>Mmi_Ceacam11N1 (Mus minutoides; Southern African pygmy mouse) WGS CACVCL010003112.1

TCTCCCTTTTAACCTGCTGGATGATTCCAACTACCGCCCAGATCACCATTGAATCAGTGCCTCCCATTGCTGTTGAAGGGGAAAATGTTCTTCTGTTTGTGCATAACGTTCCAGAGAATGTTAAAGTCCTTTCCTGGTACACAGGACTTAAACCACTCAAGAGTTGTGAAATTGCAAGTCATGTGATAGCTACCAATTCTACTGTGGTGGGACTTGCACATAGTGGTAGAGAGACTGTACTCAAAAATGGATCTCTGCTGATCAAGAGTATCACCAGAAAAGACTCAGGTTACTACACTCTACAAATACTTGATACAACCTCAAGACCTGAATTAATACGTGCAGAATTTTTTGTTCACA

>Mmi_Ceacam13N1 (Mus minutoides; Southern African pygmy mouse) WGS CACVCL010003112.1

TCTCCCTTTTATCATGCTGGCTGCATCCCACTACTTCTCAGCTCACCATTAAATCAGTGCCTCCCATTGCTGTTGAAGGGGAAAGCGTTCTTCTGTTTGTGAATAACCTGCCAAAGAATGTTAAAGCCTTTTCCTGGTACTCAGGAGTTGCACCATTCAAGTGTTGTGAAATTGCAAGTCATGTGATAGCTACCAATTTTACTGTGATGGGACTTGCACACAGTGGTAGAGAGACAGTACTCAACAATGGATCTCTGTTGATCAAGAGTGTTACCAGAAAAGACTCAGGATACTACACTCTACGAACACTTGATTCAACCTCAAGACCTGAAATAATACATGCAGAATTCTTTGTACATG

>Mmi_Ceacam14N1 (Mus minutoides; Southern African pygmy mouse) WGS CACVCL010003113.1

TCTCCCATTTAACCTGCTGGCTGATTCCCACTACTTCCCAGCTCACCATTAAATCAGTGCCTCCCATTGCTGTTGAAGGGGAAAACATTCTTCTGTCTGTGCATAACCTGCCGAAGAATGTTAAAGTCTTTTCCTGGTACTCAGGAGCTAGAGTACTCAAGAATTGTGAAATTGCAGCTCATGTGATAGCTATCAATTTTACTCTGATGGGATTTTCACATAGTGGTAGAGAGACACTGTTCAAAAATGGATCTCTGCTGATCAAAAGTGTCACCAGGAAAGACTCAGGATATTACACTCTACGAATACTTGATTCAACCTCAAGACTTGAAATAAAACGTACAGAATTCTTTGTACACA

>Mmi_Ceacam15N (Mus minutoides; Southern African pygmy mouse) WGS CACVCL010024912.1

CCTTACTTTTAATCTGCTGGAGTGTGTCCACTGCAGCACAGCTGAGCTCTAAAGAAATGCGCTTCTCGGCTGCTGAAGGGGCAAAGGTTCTTCTCTCGGTTCCTGACCAGGAAGAGAACCTCCTCTCCTTTTCCTGGTACAAAGGGAAGGATACAGATGAAAATTTTACAATTGCACATTATAAAAAGTCCAGCGATTCGCTTCAACTCGGAAAGAATGTCAGCGGCAGGGAAGAAATCTTTAAGGATGGCTCCATGATGCTCCGAGCTATCACCCTGGCAGACACGGGATTCTACACATTGCAAACCTTTAAAGCACAGGGTCAACAGGAAGTAACACACGTCCATCTCCAAGTATACA

>Mmi_Psg16N1 (Mus minutoides; Southern African pygmy mouse) WGS CACVCK010043193.1

CCTCCCTTCTAGCATTCTGGCTCCTGTCCACCACTGCCCAGGTCACCATTGAATCGGTGCCATTCAATGTGGTTGAAGGAGAAAATGTCCTTCTTCTTGTCGACAATCTGCCAGAGAATCTTACAGCCTTAGCCTGGTACAGAGGGCTGAGGAAAATTGTTGTTTACACACTGAACACTAAAGTAAGTGTGATGGGGCAAATGCACAGTGGTAGAGAGATAGTGTCCAGCAACGGGTCCCTGTGGATCCACAATGTCACCCGGAAGGACACAGGATTCTACACCCTACGGACCGTAAATAGGCGTGGAGAAATTGTATCAACATCATCAGTGTACCTCTACATGTACA

>Mmi_Psg19N1 (Mus minutoides; Southern African pygmy mouse) WGS CACVCK010020651.1

CCTCCCTCTTAGCCTGCTGGCTCCTGCCCACCACTGCCCAAATCACCATTGAATCCGTACCACCCAACTTGGTTGAAGGAGAAAATGTTCTTCTACGAGTTGACAATCTGCCAGAGAATCTTCGAGTCATTGTCTGGTACAGAGGGGTAATAAATGTTAAGCTTCGAATTGCTCTGTATTCACTGGACTATAACACAAGTGTGACGGGACCTGAGCACAGCGGTAGAGAGACATTGTACCGCAACGGGTCCCTGGGGATCCAAAATGTCACCCGGGAGGACACAGGATATTACACTCTTCAAACCATAAGTAAAAATGGAAAACTGGTATCAAATACACCCATGTTCCTTCAGGTGTACT

>Mmi_Psg20N1 (Mus minutoides; Southern African pygmy mouse) WGS CACVCL010003105.1

CCTCCCTCTTGACCTGCTGGCTCCTGTCCACCACTGCTCACATCACCATCCATTCACCGTTCCACATTGTTGAAGGAGAAAATATTCTTCTACAAGTTGACAATCTGCCGGAGCATCTTCTAGCCTTTTCCTGGTACAGAGGACTGACAAATTGGAGGCTAGCAATTGCACTGCATGTAGTGGATTATAATACAAGTATGACAGGGCCTGATCACAGCAATAGAGTGATACTGTACAGCAATGGGTCCCTGTGGATCCAAAATGTCACGCAGGAGGACACAGGATATTACACTCTTCAAACCATAAGTAAACATGGAGGACTGGTATCAAATACATCCACATTCCTTCAGGTGTACT

>Mmi_Psg21N1 (Mus minutoides; Southern African pygmy mouse) WGS CACVCK010020666.1 +

CCTCCCTCTTGACCTGCTGGCTTCTGCCTACCACTGCCAAAGTCACCATCCAGTCACCACTCAAAGTGGTTGAAGGAGAAAATATTCTTCTACGAGTTGATAATCTGCCAGAGAATCTTCTAGCTTTTGCCTGGTACAGAGGACTGGCAAATTGGAAGCTCACAATTGCTATGCATTTACTGGACTATAACGCAAGTATGACAGGGCCAGAGCACAGTGATAGAGAGCTATTGTACAGCAATGGGTCCCTGTGGATCCAAAATGTCACCCAGGAGGACACAGGATATTACACTCTTCGAACCATAAGTAAACATGGAGAACTGGAATCAAATACATCCACATACCTTCAGGTGTACT

>Mmi_Psg22N1 (Mus minutoides; Southern African pygmy mouse) WGS CACVCK010020655.1

CCTCCCTCTTAACCTGCTGGCTCCTGCCCACCACTGCCCGAGTCACCATCGATTCTGTTCCATCCAACTTGGTTGAAGGAGAAAATGTACTTCTACGGGTTGACAATTTGCCAGAGAATCTTCGAATGTTTCTCTGGCACAAAGGGGTGACAGGCACGAGCCTCGGAATTGCACTGTATTCAGTGGACTATAGCACAAGTGTGACAGGACCTTTGCACAGCGGTAGAGAGACACTGTACAGCAATGGGTCCCTGTGGATAAAAAATGTCACCCAGGAGGACACAGGATATTACACTCTTCTAACCATAAGTAAAAATGGAAAACTGGTATCAAATTCATCCATATTCCTTCAGGTGGACT

>Mmi_Psg24N1 (Mus minutoides; Southern African pygmy mouse) WGS CACVCL010027201.1

CCTCCCTCTTAACCTGCTGGCTCCTGCCCACCACTGCCCAAGTTGACATCAAATCCTTACCGCCCCAAGTGGTTGAAGGAGAAAATGTTCTTCTACGCGTTGACAATCTGCCTGAGAATCTTCGAGTCATTGTCTGGTACAGAGGGGTGACAGACGTGAGTCTTGGAATTGCTCTGTATTCACTGGACTATAGCGCAAGTGTGACTGGACCTGAGCATAGCAGTAGAGAGACATTGTACAGCAATGGGTCCCTGTGGATCCAAAATGTCACCCGGGAGGACACAGGATATTACACTCTTCTAACCACAAGTCAACGTGGAGAACTGGTATCAAATACATCCATGTTCCTTCACGTGTACT

>Mmi_Psg25N1 (Mus minutoides; Southern African pygmy mouse) WGS CACVCL010003105.1

CCTCCATCTTAACCTGCTGCCTCCTGCCCTCCACTGCCAGAGTCATCATCCATTCCTTACCACTCCAAGTGGTTGAAGGAGAAAATGTTCTTCTACATGTTTACAATCTGCCAGAGAATCTTCTAGCCTTTGTTTGGTACAGAGGGTTGATAAATTTGAATCATGGAATTGCACTGTATTCACTGCGCTATAATGTAAGTGTGACTGGTCCTAAGCACAGTGGCAGAGAGACACTGTACAGCAACGGTTCCCTGTGGATCCAAAATGTCACCCGGCAGGACACAGGATATTACACTCTTCGAACCATAAGTAAAAATGGAGAACTGGTATCAAATACATCCATGTTCCTTCAAGTGCACT

>Mmi_Psg27N1 (Mus minutoides; Southern African pygmy mouse) WGS CACVCL010003105.1

CCTCCCTCTTGACCTGCTGGCTCTTGTCCACCACTGCCCAAGTCATCATCCATTCATCCCTCCAAGTGATTGAAGGAGAAAGTGTTCTTCTACAAGTTGACAATCTGCCAGAGAATCTTCTAGCCTTTTCCTGGTACAGAGGACTGACAAATTGGAGGCTCGCAATTGCACTGCATTTACTGGACTATAACACAAGTATGACAGGGCCTGACCACAGTGATAGAGAGATATTGTACAGCAATGGATCCCTATGGATCCAAAATGTTACCCAGGAGGACACAGGATATTACACTCTTCGAACCATAAGTAAACACGGAGAACTGGTATCAAATACATCCACATTCCTTCAGGTGTACT

>Mmi_Psg29N1 (Mus minutoides; Southern African pygmy mouse) WGS CACVCL010027200.1

CCTCCCTTTTAACCTGCTGGTACCAGTCTACCACTTCCAAAGTCACCATTGAATTATTGCCACCCCAAGTGGTTGAAGGAGAAGATGTTCTTTTCCTTGTCAATAATCTGCCAGAGAATCTTACAGCCTTTGCCTGGTTTAAAGGGAGGACAAATAAGAAACGTGCAATTGCGCTGTATGCAGTAGCCTCTGACATACATGTACATAGCGATAGAGAGACATTGTACAACAATGGATCCCTGATGATCCACAATGTCACCCAGAAGGACAGAGGTTATTATACTCTACAAACCTTCAATAAACATGCAGAAACTGTATCAACAACATCCACATTCCTCCACGTGAACC

>Mmi_Psg30N1 (Mus minutoides; Southern African pygmy mouse) WGS CACVCL010003114.1

CTTTCTTTTTAACTTGCTGGCACCTTCCTACCACTGCACAAATAACCATTGAATTAGTGCCACCCCAAGTGATTGAAGGAGAAAATGTTCTCATACGAATCGGCAATCTGACAGAGAATCTTATAACCTTAGCCTGGTTCAGAGGAACGAGGATTAAGAGCCCTCAAATTGGACAATATACACTGGCCACTAATGTTACTGTGTTGGGGCCTGGTCACAGTGGTAGAGAGACTTTGTACAGCAATGGATCCCTGCAGATCTACAATGTCACCCAGGAGGACATCGGATTCTACAGCCTACGAATCATGAATAGACATGCAGAAATTGTTTCAATAACATCCATATACCTCAACGTGTACT

>Mmi_Psg31N1 (Mus minutoides; Southern African pygmy mouse) WGS CACVCL010003112.1

CCTCATTTTTAACCTGTTGTTACCTGTCTAGTGCTGCCCAAATAACAATTGAATTAGTGCCACCTCACGTGATTGAAGGAGAAAATGTTCTCATACATATCAACAATCTGCCACAGAATCTTACAACCTTAGCCTGGTTCAGAGGAATGAGCATTCAGAGCCCTCAAATTGGACAATATACACTGGCCACTAATGTTACTGTGTTGGGGCCTGGTCACAGTGGTAGAGAGACTTTGTACAGCAATGGATCCCTGCAGATCTACAATGTCACCCAGGAGGACATAGGATTCTACAGCCTACGAATCATGAATAGACATGCAGAAATTGTGTCAATAGCATCCATATACCTCAACGTGTACT

>Mmi_Psg32N1 (Mus minutoides; Southern African pygmy mouse) WGS CACVCL010003114.1

CTTTTCTTTTAACCTCTTCGTTCCTGCCCACCACTGTCCAAGTCAGCATTGAATTAGTGCCACCACAAGTGGCTGAAGGAGAAAATGTCCTTTTTATTGTTTACAGACTGCCAGAGAATCTTACAGCCATAGCCTGGTTCAAAGGAGTGACAAATACGAACCTCAGAATTGCATTGTATGAACTGGCCTCTAACATCAGTGTGAAAGGGCCTGAACACAGTGGTAGAGAGACAGTGTTCAGCAATGGATCTCTGCTGCTTCATAATGTCACCCAGAAGGACACAGGATTCTATACTATACGAACCTTAAATAGACATGGAAAAATTGTATCTACAACATCCATATACCTCCATGTTTACA

>Mmu_Ceacam9N (Mus musculus; house mouse) WGS LXEJ02002068.1

CCTTCCTCTTAACCTGCTGGAATGCACCCGCCGCTGCCGAGCTCACTATTGAATTAGTGCCACCCATGGTTGCCGAAGGCGGAAACTCCGTTCTGTTTGTGCATGAAATGCCACTGAACGTCCAGGCGTTTTACTGGTACAAACAGAGAGATTCGACGAAGAGCTACGAAGTCGCACGGTACTTAACACCCACGAACCAAAGTTCGAAGATGCCTCAGCACAGTGATAGGAAAACCGTATTCTACAGTGGATCCCTGCTGATCAGAAACGTCACCAAGGCTGACAGTGGAGTCTATACCTTACTAACATTTAACACGGAAATGGAAAGCGAATTAACACATGTGCATCTGGAAGTGCAGG

>Mmu_Ceacam11N1 (Mus musculus; house mouse) ensembl WGS CAKLHV010000014.1

TCTCCCTTTTAACCTGCTGGCTGATTCCAACTACCACCCAGATCACCATTGAATCAGTGCCTCCCATTGCTGTTGAAGGGGAAAATGTTCTTCTGTTTGTGCATAACTTGCCAGAGAATGTTCAAACCCTTTCCTGGTACACAGGAGTTAAACCACTCAAGAATTGTGAAATTGCAAGTCATGTGACAGCTACCAATTCTACTGTGGTGGGACCTGCACACAGTGGTAGAGAGATTGTACTCAAAAATGGATCTCTGCTGATCAAGAGTACCACCAGAAAAGACTCAGGTTATTACACTCTACAAATACTTGATACAACCTCAAGACCTGAATTAATACGTGCAGAATTCTTTGTTCACA

>Mmu_Ceacam12N1 (Mus musculus; house mouse) ensembl WGS CAKLHV010000014.1

TCTCCATTTTAACATGCTGGCTGCTTCCCACTACTGCCCAGATAACTATTGAATCAGTGCCTCCCATTGCTGTTGAAGGGGATAATGTTCTTCTGTTTGTGCAAAACTTGCCAGAGAATGTTCAAACCCTTTCCTGGTACAAAGGAGGTAAACTGCTCAAGATGTTTGAAATTGCAAGACACGTGATAGCTACCAATTCTAGTGTGATGGGACCTGCACACAGTGGTAGAGAGACGATGCTCAATAATGGATCTCTGATGATCAAGAATGTCACCAGAAAAGACTCGGGATACTACACTCTACAAATACTTGATACAACCTCAAGACGTGAAATAATGCGTGCAGAATTCTTTGTACAGA

>Mmu_Ceacam13N1 (Mus musculus; house mouse) ensembl WGS CAKLHV010000014.1

TCTCTCTTTTAACATGCTGGCTGCATCCCACTACTTCTCATCTCACCATTAAAGCAGTGCCTCCCATTGCTGTTGAAGGGGAAAACGTTCTTCTGTTTGTGCATAACCTGCCAAAGAATGTTAAAGCCTTTTCCTGGTACTCGGGAGCTGCACCGTTCAAGTGTTGTGAAATTGCAAGTCATGTGATAGCTACCAATTTTACTGCAGTGGGACTTGCACACAGTGGTAGAGAGACAGTACTCAACAATGGATCTCTGTTGATCAAGAGTGTTACCAGAAAAGACTCAGGATACTACACTCTACGAACACTTGATTCAACCTCAAGACCTGAAATAATACGTGCAGAATTCTTTGTACACC

>Mmu_Ceacam14N1 (Mus musculus; house mouse) ensembl WGS CAKLHV010000014.1

TCTCCCTTTTAACCTGCTGGCTGATTCCCACTACTTCCCAGCTCACCATCAAATCAGTGCCTCCCATTGCTGTTGAAGGGGAAAACGTTCTTCTGTTTGTGCATAACCTGCCGAAGAATGTTAAAGTCTTTTCCTGGTTCACAGGAGCTAGAGTGCTCAAGAGTTGTGAAATTGCAACTCATGTGATAGCTATCAATGCTACTGTGATCGGACTTTCACATAGTGGTAGAGAGACAGTGTTCAAAAATGGATCTCTGCTGATCAAGAGTGTCACCAGTAAAGACTCAGGATACTACACTCTACGGATACTTGATGCAAACTCAAGACCTAAAATAATACGTACAGAATTCTTTGTACACA

>Mmu_Ceacam15N (Mus musculus; house mouse) WGS LXEJ02002068.1

CCTCACTTTTAATCTGCTGGAACTGGTCCACTGCAGCACTGCTGACCTCTAAAGAAATGCGCTTCTCAGCTGCTGAAGGGGCAAAGGTTCTTCTCTCTGTTCCTGACCAGGAGGAGAACCTCCTCTCCTTTTCCTGGTACAAAGGGAAGGATGTAAATGAAAATTTTACAATTGCACATTATAAAAAGTCCAGCGATTCGCTTCAACTTGGAAAGAAAGTCAGCGGCAGGGAAGAAATCTATAAGGATGGCTCCATGATGCTCCGGGCCATCACCCTGGAAGACACGGGATTCTACACGTTACAAACCTTTAAAGCACACGGCCAACAGGAAGTAACACATGTCCATCTCCAAGTATACA

>Mmu_Ceacam-ps1N (Mus musculus; house mouse) WGS CAKLHV010000014.1

CCTGCCAGAACTCAAGCGCTGCCATGGCCCACCTATCTATCGAGGCTCTGCCGCCCTCTCTTCCTGAAGGGGGAAATGTTCTTCTGTGAGTTGATAATCAGTCGGAGAGGCCTCGGGCCTTTTTCTGGTACAAGGGGAAACGTGCTTTTGAAGAATTTAAAATTGCACACTATGAAACAGCCTCTCAGTCGCTTAAGTGGGGGCGAAAATACAGCGGCAGGGAGAGAGTTTTCACCAATGGATCCCTGCTGCTCCAGAAAGTCACCCAGGAAGACACAGGGATCTACACCCTAGAGAGCTTTGATGCATATTATCAGTGTGAAATAGCACATGTCCGCCTCCAAGTGTTCa

>Mmu_Psg16N1 (Mus musculus; house mouse) WGS CAKLHV010000014.1

CCTCCCTTTTAGCCTGCTGGCTCCTGTCCACCACTGCCCAGGTCACCATTGAATCAGTGCCTTTCAATGTGGTTGAAGGAGAAAATGTCCTTCTTCGTGTTGACAATCTGCCAGAGAATCTTATAACCTTAGCCTGGTACAGAGGGCTGAGGAAAATTGTTGTATACACACTGAACACTAAAGTAAGTGTGATGGGGCAAATGTACAGTGGTAGAGAGATAGTGTCCAGCAACGGGTCCCTGTGGATCCACAATGTCACCCGGAAGGACACAGGACTCTACACCCTACGGACCGTAAATAGACGTGGAGAAATTGTATCAACATCATTCACGTTCCTCTACGTGTACA

>Mmu_Psg17N1 (Mus musculus; house mouse) WGS CAKLHV010000014.1

CCTCCCTCTTATCCTGCTGCCTCCTGCCCACCACTGCCAGAGTCACTGTGGAATTCTTACCTCCCCAAGTGGTTGAAGGAGAAAATGTTCTTCTACGCGTTGATAATCTGCCAGAGAATCTTCTAGGTTTTGTGTGGTACAAAGGGGTGGCAAGTATGAAGCTTGGAATTGCACTGTATTCACTGCAATATAATGTAAGTGTGACAGGGCTTAAGCACAGCGGTAGAGAGACACTGCACAGAAATGGGTCCCTGTGGATCCAAAATGTCACCTCGGAGGACACAGGATATTACACCCTTCGAACCGTAAGTCAACGTGGAGAACTGGTATCAGATACATCCATATTCCTTCAGGTGTACT

>Mmu_Psg18N1 (Mus musculus; house mouse) WGS CAKLHV010000014.1

CCTCCCTCTTAACCTGCTGGCTCCTGCCCACCACTGCCAGAGTCACCATTGAATCTTTACCACCCCAAGTGTATGAAGGAGAAAATGTTCTTCTACGTGTTGACAATATGCCAGAGAATCTTCTAGTGTTTGGCTGGTACAGAGGAATGACAAATTTGTGGCAAGCAATTGCACAGCATTGGCTGTACTACTATAGTGTAATGGTGAAGGGGCTGAATCACAGCGGCAGAGAGATATTATACATCAACGGGTCCCTGTGGATCCAAAATGTCACACAAGAGGACACAGGATATTACACTTTTCAAACCATAAGTAAACGAGGAGAAATAGTATCAAATACATCCCTGTACTTGCACGTGTACT

>Mmu_Psg19N1 (Mus musculus; house mouse) WGS CAKLHV010000014.1

CCTCCCTCTTAACCTGCTGGTTCCTGCCCATCACTGCCCGAGTCACCATCGAATCCGTACCACCCAAATTGGTCGAAGGAGAAAATGTTCTTCTACGAGTGGACAATCTGCCAGAGAATCTTCGAGTCTTTGCCTGGTACAGAGGGGTAATAAAATTTAAGCTTGGAATTGCACTGTATTCACTGGACTATAACACAAGTGTGACAGGACCTGAGCACAGTGGTAGAGAGACATTGCACAGCAACGGGTCCCTGTGGATCCAAAGTGCCACCCGGGAAGACACAGGATATTACACGTTTCAAACCATAAGTAAAAATGGAAAAGTGGTATCAAATACATCCATGTTCCTTCAGGTGTACT

>Mmu_Psg20N1 (Mus musculus; house mouse) WGS CAKLHV010000014.1

CCTCCCTCTTTACCTGCTGGCTTCTGTCCACCACTGCCAAGGTCACTATCCATTCACCGCTCCAAGTGGTTGAAGGACAAAACGTTTTTCTACGAGTTGACAATCTGCCAGAGGATCTTCTAGCTTTTGCCTGGTACAGAGGACTGAGAAATTGGAGGGTCGCAATTGCACTGCATTTAGTGGAGTATAATGCAAGTATGACAGGGCCTGAGCACAGTGATAGAGAGATATTGCACAGCAACGGGTCCCTGTGGATCCAAAATGTCACCCAGGAGGACACAGGATATTATACTCTTCAAACCATAAGTAAACATGGAAAACTGGTATCAAATACATCCACATTTCTTCAGGTGTACT

>Mmu_Psg21N1 (Mus musculus; house mouse) WGS CAKLHV010000014.1

CCTCCCTCTTGACCTGCTGGCTTCTGTCCACCACTGCTAGTGTCACCATCCAGTCACCAAAACACGTAGTTGAAGGAGAGAATATTCTTCTACAAGTTGACAATCTGCCAGAGAATCTTCTAGCTTTTGCCTGGTACAGAGGACTGATAAATTGGAGGCTCACAATTGCTCTGCATTTTCTGGACTATAGCACAAGTATGACAGGGCCTGAGCACAGTGATAGAGAGATATTGTACAGCAACGGGTCCCTATGGATCCAAAATGTCACCCAGGAGGACACAGGATATTACATTTTTCAAACCATAAGTAACCATGGAGAACTGGAATCAAATACGTCCACATTTCTTCAGGTCTACT

>Mmu_Psg22N1 (Mus musculus; house mouse) WGS CAKLHV010000014.1

CCTCCCTCTTAACCTGCTGGCTCTTGCCCATCACTGCCGGAGTCACCATCGAATCCGTACCACCCAAATTGGTTGAAGGAGAAAATGTTCTTCTACGAGTGGACAATCTGCCAGAGAATCTTCGAGTCTTTGTCTGGTATAGAGGGGTGACAGACATGAGCCTCGGAATTGCATTGTATTCACTTGACTATAGCACAAGTGTGACAGGACCTAAGCACAGTGGTAGAGAGACATTGTACAGAAACGGGTCCCTGTGGATCCAAAATGTCACCCGGGAAGACACAGGATATTACACTCTTCAAACCATAAGTAAAAATGGAAAAGTGGTATCAAATACATCCATATTCCTTCAGGTGAACT

>Mmu_Psg23N1 (Mus musculus; house mouse) WGS CAKLHV010000014.1

CCTCCCTCTTGACCTGCTGGCTTCTGTCCACCACTGCTAGTGTCACCATCCAGTCACCACAACACGTAGTTGAAGGAGAGAATATTCTTCTACAAGTTGACAATCTGCCAGAGAATCTTCTAGCTTTTGCCTGGTACAGAGGACTGACAAATTGGAGGCTCACAATTGCTGTGTATTTACTGGACTATAGCACAAGTATGACAGGGCCTGAGCACAGTGATAGAGAGATATTGTACAGCAACGGGTCCCTATGGATCCAAAATGTCACACAGGAGGACACAGGATATTACACTCTTCAAACCATAAGTAACCATGGAGAACTGGAATCAAATACATCCACATTTCTTCAGGTCTACT

>Mmu_Psg24N1 (Mus musculus; house mouse) WGS CAKLHV010000014.1

CCTCCCTCTTAACCTGCTGGCTCCTGCCCACCACTACCCAAGTCGACATCGAATCCTTACCACCCCAAGTGGTTGAAGGAGAAAATGTTCTTCTACGGGTTGACAATCTGCCAGAGAATCTTCTAGGCTTTATCTGGTACAAAGGGGTGACAGACATGAGCCTCGGAATTGCACTGTATTCACTGACCTATAGCAGAGGTGTGACGGGACCTGTGCACAGTGGTAGAGAGACATTGTACCGAAATGGGTCCCTGTGGATTCAAAATGTCACCCAGGAGGACACAGGATTCTACACCCTACGAACCATAAGTAAACGTGGAGAAATTATATCAAATACATCCATGCACCTTCATGTGTACT

>Mmu_Psg25N1 (Mus musculus; house mouse) WGS CAKLHV010000014.1

CCTCCATCTTAACCTACTGGCTCCTGCCCACCACTGCCAGAGTCATCATCCATTCTTTACCACTCCAAGTGGTTGAAGGAGAAAATGTTCTTCTACATGTTTACAATCTGCCAGAGAATCTTCTAGGCCTTGCCTGGTACAGAGGGTTGCTAAATTTGAAACTTGGAATTGCACTGTATTCACTACAATATAATGTAAGTGTGACTGGACCTGAGCACAGCGGTAGAGAGACATTGCACAGAAATGGGTCTCTGTGGATCCAAAATGTCACCCAGGAGGACACAGGATATTACACTCTTCGTACCATAAGTAAAAATGGAAAACTGGAATCAAATACATCCATGTTCCTTCAGGTGTACT

>Mmu_Psg26N1 (Mus musculus; house mouse) WGS CAKLHV010000014.1

CCTCCCTCTTAACCTGCTGGTTCCTGCCCACCACTGCCAGAGTCACCATTGAATCCTTACCACCCCAAGTGGTTGAAGGAGAAAATGTTCTTCTACGTGTTGACAATATGCCAGAGAATCTTCTAGTGTTTGGCTGGTACAGAGGAATGACAAATTTGAGGCAAGCAATTGCACTGCATTCGCTGTACTATAGTGTAACGGTGAAGGGGCTGAAGCACAGCGGCAGAGAGACATTATACATCAACGGGACCCTGTGGATCCAAAATGTCACACAGGAGGACACAGGATATTACACTTTTCAAACCATAAGTAAACAAGGAGAAATGGTATCAAATACATCCCTGTACTTGCACGTGTACT

>Mmu_Psg27N1 (Mus musculus; house mouse) WGS CAKLHV010000014.1

CCTCCCTCTTTACCTGCTGGCTCCTCTCCACTACTGCCCGAGTCACCATCCATTCACCGCTCCAAGTGGTTGAAGGAGAAAATGTTCTTCTACGAGTTGACAATCTGCCCGAGAATCTTCTAGCCTTTTCCTGGTATAGAGGCCTGAAAAATTGGCAGCTTGCAATTGCACTACATTTACTGGACTATAACACAAGTATGACAGGGCCTGATCACAGTGATAGAGAGATATTGTACAGCAATGGATCCCTATGGATCCAAAATGTCACCAAGGAGGACACAGGATATTATACTCTTCGAACCATAAGCAAACATGGAGAACTGGTATCAAATACATCCACATTTCTTCAGGTGTACT

>Mmu_Psg28N1 (Mus musculus; house mouse) WGS CAKLHV010000014.1

CCTTCCTCTTAACCTGCTGGCACCTGCCCACCACTGCCAGAGTCACCATTGAATCCTTTCCACCCCAAGTGGTTGAAGGAGAAAATGTTCTTCTACGTGTTGACAATATGCCAGAGAATCTTCTAGTGTTTGGCTGGTACAGAGGAATGACAAATTTGAGACATGCAATTGCACTGTACTATAGTTTAACAGCGAAGGGGCTGAAGCACAGCGGCAGAGAGACATTATACATCAACGGGTCCCTGTGGATCCAAAATGTCACACAGGAGGACACAGGATATTACACCTTTCAAACCATAAGTAAACAAGGAGAAATGGTATCAAATACATCCCTGTACTTGCACGTGTACT

>Mmu_Psg29N1 (Mus musculus; house mouse) WGS CAKLHV010000014.1

CCTCCTTCTTAACCTGCTGGTATCTGTCTACCACTTCCAAAGTCACCATTGAATTATTGCCATCCCAAGTGGTTGAAGGAGAAGATGTTCTTTTCCTTGTCAATAATCTGCCAGGGAATCTTACAGCCTTTGCCTGGTTTAAAGGGAGGACAAATAGGAAACATGGAATTGCACTGTATGCAGTGGCGTCTGACTTATATGTACACAGCGATAGAGAGACATTGTACAACAACGGATCCCTGATGATCCACAATGTTACCCAGAAGGACAGAGGTTATTACACCCTACGAACCTTCAATAAACATGCAGAAACTGTATCAACAACATTCACATTCCTCCACGTGAACC

>Mmu_Psg30N1 (Mus musculus; house mouse) WGS CAKLHV010000014.1

CCTCCTTTTTAACCTGCTGTCACCTGCCTACCACTGCACAAATAACCATTGAATTAGAGCCACCCCAAGTGATTGAAGGAGAAAATGTTCTCATACGTGTCAACAATTTGACAGAGAATCTTATAACCTTAGCCTGGTTCAGAGGAATGAGGATTAAGAGCCCTCAAATTGGACAATATACACCGGCCACTAAAGTTACTGTGCTGGGTCCTGGTCACAGTGGTAGAGAAACTTTGTACAGCAATGGATCCCTGCAGATCTACAATGTCACCCAGGAGGACATAGGATTCTACAGCCTACGAATCATAAATAAACATGCAGAAATTGTGTCAATAACATCCATATACCTCAACGTGTACT

>Mmu_Psg31N1 (Mus musculus; house mouse) WGS CAKLHV010000014.1

CCTCATTTTTAACCTGTTGTCACCTGCCTGCCACTGCCCAAATAACCATTGAATTAGTGCCACCCCATGTGATTGAAGGAGAAAATGTTCTCATACGTGTCAACAATCTGCCAGAGAATCTTACAACCTTAGTCTGGTTCAGAGGAATGAGGATTAAGAGCCCTCAAATTGGACAATATACACTGGCCACTAATGTTACTGTGCTGGGGCCTGGTCACAGTGGTAGAGAAACTTTGTACAGCAATGGATCCCTGCAGATCTACAATGTCACCCAGGAGGACATAGGATTCTACAGCCTACGAGTCATGAATAGACATGGAAAAATTGTGTCAATAACATCCATATACCTCAACGTGTACT

>Mmu_Psg32N1 (Mus musculus; house mouse) WGS CAKLHV010000014.1

CTTTTCTTTTAACCTCTTGGTTCCTGCCCACCACTGTCCAAGTCACCATTGAATTAGTGCCACCACAAGTGGCTGAAGGAGAAAATGTCCTTATTATTGTTTACAGTCTGCCAGAGGATCTTACAGCCATAGCCTGGTTCAAAGGAGTGACAAATATGAACCTCGGAATTGCATTGTATGCACTGGCCTCTAACATCAGTGTGAAAGGGCCCGAACACAGTGGTAGAGAGACAGTGTTCAGCAATGGATCCCTGTTGCTTCACAATGTCACCCAGAAGGACACAGGATTCTATACTATACGGACCTTAAATAGACATGGAAAAATTGTATCCACAACATCCATATACCTCCACGTGTACA

>Mmu_cas_Ceacam9N (Mus musculus castaneus; southeastern Asian house mouse) WGS LVXN01029175.1

CCTTCCTCTTAACCTGCTGGAATGCAACCGCCGCTGCCGAGCTCACTATTGAATTAGTGCCACCCATGGTTGCCGAAGGCGGAAACTCCGTTCTGTTTGTGCATGAAATGCCACTGAACGTCCAGGCGTTTTACTGGTACAAACAGAGAGATTCGACGAAGAGCTACGAAGTCGCACGGTACTTAACACCCACGAACCAAAGTTCGAAGATGCCTCAGCACAGTGATAGGAAAACCGTATTCTACAGTGGATCCCTGCTGATCAGAAACGTCACCAAGGCTGACAGTGGAGTCTATACCTTACTAACATTTAACACGGAAATGGAAAGCGAATTAACACATGTGCATCTGGAAGTGCATG

>Mmu_cas_Ceacam11N1 (Mus musculus castaneus; southeastern Asian house mouse) WGS LVXN01029218.1

TCTCCCTTTTAACCTGCTGGCTGATTCCAACTACCACCCAGATCACCATTGAATCAGTGCCTCCCATTGCTGTTGAAGGGGAAAATGTTCTTCTGTTTGTGCATAACTTGCCAGAGAATGTTAAAGCCCTTTCCTGGTACACAGGAGTTAAACCACTCAAGAATTGTGAAATTGCAAGTCATGTGATAGCTACCAATTCTACTGTGGTGGGACCTGCACACAGTGGTAGAGAGATTGTGCTCAAAAATGGATCTCTGCTGATCAAGAGTACCACCAGAAAAGACTCAGGTTACTACACTCTACAAATACTTGATACAACCTCAAGACCTGAATTAATACGTGCAGAATTCTTTGTTCACA

>Mmu_cas_Ceacam12N1 (Mus musculus castaneus; southeastern Asian house mouse) WGS LVXN01029218.1

TCTCCATTTTAACATGCTGGCTGCTTCCCACTACTGCCCAGATAACTATTGAATCAGTGCCTCCCATTGCTGTTGAAGGGGATAATGTTCTTCTGTTTGTGCAAAACTTGCCAGAGAATGTTCAAACCCTTTCCTGGTACACAGGAGGTAAACCGCTCAAGATGTTTGAAATTGCAAGACATGTGATAGCTACCAATTCTAGTGTGATGGGACCTGCACACAGTGGTAGAGAGACGATGCTCAATAATGGATCTCTGATGATCAAGAATGTCACCAGAAAAGACTCGGGATACTACACTCTACAAATACTTGATACAACCTCAAGACGTGAAATAATGCGTGCAGAATTCTTTGTACAGA

>Mmu_cas_Ceacam13N1 (Mus musculus castaneus; southeastern Asian house mouse) WGS LVXN01029218.1

TCTCTCTTTTAACATGCTGGCTGCATCCCACTACTTCTCATCTCACCATTAAAGCAGTGCCTCCCATTGCTGTTGAAGGGGAAAACGTTCTTCTGTTTGTGCATAACCTGCCAAAGAATGTTAAAGCCTTTTCCTGGTACTCGGGAGTTGCACCGTTCAAGTGTTGTGAAATTGCAAGTCATGTGATAGCTACCAATTTTACTGCAGTGGGACTTGCACACAGTGGTAGAGAGACAGTACTCAACAATGGATCTCTGTTGATCAAGAGTGTTACCAGAAAAGACTCAGGATACTACACTCTACGAACACTTGATTCAACCTCAAGACCTGAAATAATACGTGCAGAATTCTTTGTACACC

>Mmu_cas_Ceacam14N1 (Mus musculus castaneus; southeastern Asian house mouse) WGS LVXN01029214.1

TCTCCCTTTTAACCTGCTGGCTGATTCCCACTACTTCCCAGCTCACCATCAAATCAGTGCCTCCCATTGCTGTTGAAGGGGAAAATGTTCTTCTGTTTGTGCATAACCTGCCGAAGAATGTTAAAGTCTTTTCCTGGTTCACAGGAGCTAGAGTGCTCAAGAGTTGTGAAATTGCAACTCATGTGATAGCTATCAATGCTACTGTGATCGGACTTTCACATAGTGGTAGAGAGACAGTGTTCAAAAATGGATCTCTGCTGATCAAGAGTGTCACCAGTAAAGACTCAGGATACTACACTCTACGGATACTTGATGCAAACCCAAGACCTGAAATAATACGTACAGAATTCTTTGTACACA

>Mmu_cas_Ceacam15N (Mus musculus castaneus; southeastern Asian house mouse) WGS LVXN01029175.1

CCTCACTTTTAATCTGCTGGAACTGGTCCACTGCAGCACTGCTGACCTCTAAAGAAATGCGCTTCTCAGCTGCTGAAGGGGCAAAGGTTCTTTTCTCTGTTCCTGACCAGGAGGAGAACCTCCTCTCCTTTTCCTGGTACAAAGGGAAGGATGTAAATGAAAATTTTACAATTGCACATTATAAAAAGTCCAGCGATTCACTTCAACTTGGAAAGAAAGTCAGCGGCAGGGAAGAAATCTATAAGGATGGCTCCATGATGCTCCGGGCCATCACCCTGGAAGACACGGGATTCTACACGTTACAAACCTTTAAAGCACATGGCCAACAGGAAGTAACACATGTCCATCTCCAAGTATACA

>Mmu_cas_Psg16N1 (Mus musculus castaneus; southeastern Asian house mouse) WGS LVXN01029177.1

CCTCCCTTTTAGCCTGCTGGCTCCTGTCCACCACTGCCCAGGTCACCATTGAATCAGTGCCATTCAATGTGGTTGAAGGAGAAAATGTCCTTCTTCGTGTTGACAATCTGCCAGAGAATCTTATAACCTTAGCCTGGTACAGAGGGCTGAGGAAAATTGTTGTATACACACTGAACACTAAAGTAAGTGTGATGGGGCAAATGTACAGTGGTAGAGAGATAGTGTCCAGCAACGGGTCCCTGTGGATCCACAATGTCACCCGGAAGGACACAGGACTCTACACCCTACGGACCGTAAATAGACGTGGAGAAATTGTATCAACATCATTCACGTACCTCTACGTGTACA

>Mmu_cas_Psg17N1 (Mus musculus castaneus; southeastern Asian house mouse) WGS LVXN01029273.1

CCTCCCTCTTATCCTGCTGCCTCCTGCCCACCACTGCCAGAGTCACTGTGGAATTCTTACCTCCCCAAGTGGTTGAAGGAGAAAATGTTCTTCTACGCGTTGATAATCTGCCAGAGAATCTTCTAGGTTTTGTGTGGTACAAAGGGGTGGCAAGTATGAAGCTTGGAATTGCACTGTATTCACTGCAATATAATGTAAGTGTGACAGGGCTTAAGCACAGCGGTAGAGAGACACTGCACAGAAATGGGTCCCTGTGGATCCAAAATGTCACCTCGGAGGACACAGGATATTACACCCTTCGAACCGTAAGTCAACGTGGAGAACTGGTATCAGATACATCCATATTCCTTCAGGTGTACT

>Mmu_cas_Psg18N1 (Mus musculus castaneus; southeastern Asian house mouse) WGS LVXN01050623.1

CCTCCCTCTTAATCTGCTGGCTCCTGCCCACCACTGCCAGAGTCACCATTGAATCCTTACCACCCCAAGTGTATGAAGGAGAAAATGTTCTACTACGTGTTGACAATATGCCAGAGAATCTTCTAGTGTTTGGCTGGTACAGAGGAATGACAAATTTGAGGCAAGCAATTGCACAGCATTGGCTGTACTACTATAGTGTAATGGTGAAGGGGCTGAATCACAGCGGCAGAGAGATATTATACATCAACGGGTCCCTGTGGATCCAAAATGTCACACAGGAGGACACAGGATATTACACTTTTCAAACCATAAGTAAACGAGGAGAAATAGTATCAAATACATCCCTGTACTTGCACGTGTACT

>Mmu_cas_Psg19N1 (Mus musculus castaneus; southeastern Asian house mouse) WGS LVXN01029268.1

CCTCCCTCTTAACCTGCTGGTTCCTGCCCATCACTGCCCGAGTCACCATCGAATCCGTACCACCCAAATTGGTCGAAGGAGAAAATGTTCTTCTACGAGTGGACAATCTGCCAGAGAATCTTCGAGTCTTTGCCTGGTACAGAGGGGTAATAAAATTTAAGCTTGGAATTGCACTGTATTCACTGGACTATAACACAAGTGTGACAGGACCTGAGCACAGTGGTAGAGAGACATTGCACAGCAACGGGTCCCTGTGGATCCAAAGTGCCACCCGGGAAGACACAGGATATTACACGTTTCAAACCATAAGTAAAAATGGAAAAGTGGTATCAAATACATCCATGTTCCTTCAGGTGTACT

>Mmu_cas_Psg20N1 (Mus musculus castaneus; southeastern Asian house mouse) WGS LVXN01029266.1

CCTCCCTCTTTACCTGCTGGCTTCTGTCCACCACTGCCAAGGTCACTATCCATTCACCGCTTCAAGTGGTTGAAGGACAAAACGTTTTTCTACGAGTTGACAATCTGCCAGAGGATCTTCTAGCTTTTGCCTGGTACAGAGGACTGAGAAATTGGAGGGTCGCAATTGCACTGCGTTTAGTGGAGTATAATGCAAGTATGACAGGGCCTGAGCACAGTGATAGAGAGATATTGCACAGCAACGGGTCCCTGTGGATCCAAAATGTCACCCAGGAGGACACAGGATATTATACTCTTCAAACCATAAGTAAACATGGAAAACTGGTATCAAATACATCCACATTTCTTCAGGTGTACT

>Mmu_cas_Psg21N1 (Mus musculus castaneus; southeastern Asian house mouse) WGS CAKLHS010000017.1

CCTCCCTCTTGACCTGCTGGCTTCTGTCCACCACTGCTAGTGTCACCATCCAGTCACCAAAACACGTAGTTGAAGGAGAGAATATTCTTCTACAAGTTGACAATCTGCCAGAGAATCTTCTAGCTTTTGCCTGGTACAGAGGACTGATAAATTGGAGGCTCACAATTGCTCTGCATTTcCTGGACTATAGCACAAGTATGACAGGGCCTGAGCACAGTGATAGAGAGATATTGTACAGCAACGGGTCCCTATGGATCCAAAATGTCACCCAGGAGGACACAGGATATTACATTTTTCAAACCATAAGTAACCATGGAGAACTGGAATCAAATACaTCCACATTTCTTCAGGTCTACT

>Mmu_cas_Psg22N1 (Mus musculus castaneus; southeastern Asian house mouse) WGS LVXN01029266.1

CCTCCCTCTTAACCTGCTGGCTCTTGCCCATCACTGCCGGAGTCACCATCGAATCCGTACCACCCAAATTGGTTGAAGGAGAAAATGTTCTTCTACGAGTGGACAATCTGCCAGAGAATCTTCGAGTCTTTGTCTGGTATAGAGGGGTGACAGACATGAGCCTCAGAATTGCATTGTATTCACTTGACTATAGCACAAGTGTGACAGGACCTAAGCACAGCGGTAGAGAGACATTGTACAGAAACGGGTCCCTGTGGATCCAAAATGTCACCCGGGAAGACACAGGATATTACACTCTTCAAACCATAAGTAAAAATGGAAAAGTGGTATCAAATACATCCATATTCCTTCAGGTGAACT

>Mmu_cas_Psg23N1 (Mus musculus castaneus; southeastern Asian house mouse) WGS LVXN01051839.1

CCTCCCTCTTGACCTGCTGGCTTCTGTCCACCACTGCTAGTGTCACCATCCAGTCACCACAACACGTAGTTGAAGGAGAGAATATTCTTCTACAAGTTGACAATCTGCCAGAGAATCTTCTAGCTTTTGCCTGGTACAGAGGACTGACAAATTGGAGGCTCACAATTGCTGTGTATTTACTGGACTATAGCACAAGTATGACAGGGCCTGAGCACAGTGATAGAGAGATATTGTACAGCAACGGGTCCCTATGGATCCAAAATGTCACCCAGGAGGACACAGGATATTACACTCTTCAAACCATAAGTAACCATGGAGAACTGGAATCAAATACATCCACATTTCTTCAGGTCTACT

>Mmu_cas_Psg24N1 (Mus musculus castaneus; southeastern Asian house mouse) WGS LVXN01029182.1

GCCTCCCTCTTAACCTGCTGGCTCCTGCCCACCACTACCCAAGTCGACATCGAATCCTTACCGCCCCAAGTGGTTGAAGGAGAAAATGTTCTTCTACGGGTTGACAATCTGCCAGAGAATCTTCTAGGCTTTATCTGGTACAAAGGGGTGACAGACATGAGCCTTGGAATTGCACTGTATTCACTGACCTATAGCAGAGGTGTGACGGGACCTGTGCACAGTGGTAGAGAGACATTGTACCGAAATGGGTCCTTGTGGATTCAAAATGTCACCCAGGAGGACACAGGATTCTACACCCTACGAACCATAAGTAAACATGGAGAAATTATATCAAATACATCCATGCACCTTCATGTGTACT

>Mmu_cas_Psg25N1 (Mus musculus castaneus; southeastern Asian house mouse) WGS LVXN01029251.1

CCTCCATCTTAACCTACTGGCTCCTGCCCACCACTGCCAGAGTCATCATCCATTCTTTACCACTCCAAGTGGTTGAAGGAGAAAATGTTCTTCTACATGTTTACAATCTGCCAGAGAATCTTCTAGGCCTTGCCTGGTACAGAGGGTTGCTAAATTTGAAACTTGGAATTGCACTGTATTCACTACAATATAATGTAAGTGTGACTGGACCTGAGCACAGCGGTAGAGAGACATTGCACAGAAATGGGTCTCTGTGGATCCAAAATGTCACCCAGGAGGACACAGGATATTACACTCTTCGTACCATAAGTAAAAATGGAAAACTGGAATCAAATACATCCATGTTCCTTCAGGTGTACT

>Mmu_cas_Psg26N1 (Mus musculus castaneus; southeastern Asian house mouse) WGS CAKLHS010000017.1

CCTCCCTCTTAACCTGCTGGTTCCTGCCCACCACTGCCAGAGTCACCATTGAATCCTTTCCACCCCAAGTGGTTGAAGGAGAAAATGTTCTTCTACGTGTTGACAATATGCCAGAGAATCTTCTAGTGTTTGGCTGGTACAGAGGAATGACAAATTGGAGGCAAGCAATTGCACTGCATTCGCTGTACTATAGTGTAACAGTGAAGGGGCTGAAGCACAGCGGCAGAGAGACATTATACATCAACGGGACCCTGTGGATCCAAAATGTCACACAGGAGGACACAGGATATTACACTTTTCAAACCATAAGCAAACAAGGAGAAATGGTATCAAATACATCCCTGTACTTGCACGTGTACT

>Mmu_cas_Psg27N1 (Mus musculus castaneus; southeastern Asian house mouse) WGS LVXN01029251.1

CCTCCCTCTTTACCTGCTGGCTCCTCTCCACTACTGCCCGAGTCACCATCCATTCACCGCTCCAAGTGGTTGAAGGAGAAAATGTTCTTCTACGAGTTGACAATATGCCCGAGAATCTTCTAGCCTTTTCCTGGTATAGAGGACTGAAAAATTGGCAGCTTGCAATTGCACTACATTTACTGGACTATAACACAAGTATGACAGGGCCTGATCACAGTGATAGAGAGATATTGTACAGCAATGGATCCCTATGGATCCAAAATGTCACCAAGGAGGACACAGGATATTATACTCTTCGAACCATAAGCAAACATGAAGAACTGGTATCAAATACATCCACATTTCTTCAGGTGTACT

>Mmu_cas_Psg28N1 (Mus musculus castaneus; southeastern Asian house mouse) WGS CAKLHS010000017.1

CCTCCCTCTTAACCTGCTGGCTCCTGCCCACCACTGCCAGAGTCACCATTGAATCCTTTCCACCCCAAGTGGTTGAAGGAGAAAATGTTCTTCTACGTGTTGACAATATGCCAGAGAATCTTCTAGTGTTTGGCTGGTACAGAGGAATGACAAATTTGAGGCATGCAATTGCACTGCACTATAGTTTAACAGCGAAGGGGCTGAAGCACAGCGGCAGAGAGACATTATACATCAACGGGTCCCTGTGGATCCAAAATGTCACACAGGAGGACACAGGATATTACACCTTTCAAACCATCAGTAAACAAGGAGAAATGGTATCAAATACATCCCTGTACTTGCACGTGTACT

>Mmu_cas_Psg29N1 (Mus musculus castaneus; southeastern Asian house mouse) WGS

CCTCCTTCTTAACCTGCTGGTATCTGTCTACCACTTCCAAAGTCACCATTGAATTATTGCCATCCCAAGTGGTTGAAGGAGAAGATGTTCTTTTCCTTGTCAATAATCTGCCAGGGAATCTTACAGCCTTTGCCTGGTTTAAAGGGAGGACAAATAGGAAACATGGAATTGCACTGTATGCAGTGGCGTCTGACTTATATGTACACAGCGATAGAGAGACATTGTACAACAACGGATCCCTGATGATCCACAATGTTACCCAGAAGGATAGAGGTTATTATACCCTACGAACCTTCAATAAACATGCAGAAACTGTATCAACAACATTCACATTCCTCCACGTGAACC

>Mmu_cas_Psg30N1 (Mus musculus castaneus; southeastern Asian house mouse) WGS LVXN01029207.1

CCTCCTTTTTAACCTGCTGTCACCTGCCTACCACTGCACAAATAACTATTGAATTAGAGCCACCCCAAGTGATTGAAGGAGAAAATGTTCTCATACGTGTCAACAATTTGACAGAGAATCTTATAACCTTAGCCTGGTTCAGAGGAATGAGGATTAAGAGCCCTCAAATTGGACAATATACACCGGCCACTAAAGTTACTGTGCTGGGTCCTGGTCACAGTGGTAGAGAAACTTTGTACAGCAATGGATCCCTGCAGATCTACAATGTCACCCAGGAGGACATAGGATTCTACAGCCTACGAATCATAAATAAACATGCAGAAATTGTGTCAATAATATCCATATACCTCAATGTGTACT

>Mmu_cas_Psg31N1 (Mus musculus castaneus; southeastern Asian house mouse) WGS CAKLHS010000017.1

CCTCATTTTTAACCTGTTGTCACCTGCCTGCCACTGCCCAAATAACCATTGAATTAGTGCCACCCCATGTGATTGAAGGAGAAAATGTTCTCATACGTGTCAACAATCTGCCAGAGAATCTTACAACCTTAGCCTGGTTCAGAGGAATGAGGATTAAGAGCCCTCAAATTGGACAATATACACTGGCCACTAATGTTACTGTGCTGGGACCTGGTCACAGTGGTAGAGAAACTTTGTACAGCAATGGATCCCTGCAGATCTACAATGTCACCCAGGAGGACATAGGATTCTACAGCCTACGAGTCATGAATAGACATGGAGAAATTGTGTCAATAACATCCATATACCTCAACGTGTACT

>Mmu_cas_Psg32N1 (Mus musculus castaneus; southeastern Asian house mouse) WGS LVXN01029207.1

CTTTTTTTTTAACCTCTTGGTTCCTGCCCACCACTGTCCAAGTCACCATTGAATTAGTGCCACCACAAGTGGCTGAAGGAGAAAATGTCCTTATTATTGTTTACAGTCTGCCAGAGGATCTTACAGCCATAGCCTGGTTCAAAGGAGTGACAAATATGAACCTCGGAATTGCATTGTATGCACTAGCCTCTAACATCAGCGTGAAAGGGCCCGAACACAGTGGTAGAGAGACAGTGTTCAGCAATGGATCCCTGTTGCTTCACAATGTCACCCAGAAGGACACAGGATTCTATACTATACGGACCTTAAATAGACATGGAAAAATTGTATCCACAACATCCATATACCTCCACGTGTACA

>Mmu_dom_Ceacam9N (Mus musculus domesticus; western European house mouse) WGS LVXW01029911.1

CCTTCCTCTTAACCTGCTGGAATGCACCCGCCGCTGCCGAGCTCACTATTGAATTAGTGCCACCCATGGTTGCCGAAGGCGGAAACTCCGTTCTGTTTGTGCATGAAATGCCACTGAACGTCCAGGCGTTTTACTGGTACAAACAGAGAGATTCGACGAAGAGCTACGAAGTCGCACGGTACTTAACACCCACGAACCAAAGTTCGAAGATGCCTCAGCACAGTGATAGGAAAACCGTATTCTACAGTGGATCCCTGCTGATCAGAAACGTCACCAAGGCTGACAGTGGAGTCTATACCTTACTAACATTTAACACGGAAATGGAAAGCGAATTAACACATGTGCATCTGGAAGTGCAGG

>Mmu_dom_Ceacam11N1 (Mus musculus domesticus; western European house mouse) WGS LVXW01029994.1

TCTCCCTTTTAACCTGCTGGCTGATTCCAACTACCACCCAGATCACCATTGAATCAGTGCCTCCCATTGCTGTTGAAGGGGAAAATGTTCTTCTGTTTGTGCATAACTTGCCAGAGAATGTTCAAACCCTTTCCTGGTACACAGGAGTTAAACCACTCAAGAATTGTGAAATTGCAAGTCATGTGACAGCTACCAATTCTACTGTGGTGGGACCTGCACACAGTGGTAGAGAGATTGTACTCAAAAATGGATCTCTGCTGATCAAGAGTACCACCAGAAAAGACTCAGGTTATTACACTCTACAAATACTTGATACAACCTCAAGACCTGAATTAATACGTGCAGAATTCTTTGTTCACA

>Mmu_dom_Ceacam12N1 (Mus musculus domesticus; western European house mouse) WGS LVXW01029994.1

TCTCCATTTTAACATGCTGGCTGCTTCCCACTACTGCCCAGATAACTATTGAATCAGTGCCTCCCATTGCTGTTGAAGGGGATAATGTTCTTCTGTTTGTGCAAAACTTGCCAGAGAATGTTCAAACCCTTTCCTGGTACAAAGGAGGTAAACTGCTCAAGATGTTTGAAATTGCAAGACACGTGATAGCTACCAATTCTAGTGTGATGGGACCTGCACACAGTGGTAGAGAGACGATGCTCAATAATGGATCTCTGATGATCAAGAATGTCACCAGAAAAGACTCGGGATACTACACTCTACAAATACTTGATACAACCTCAAGACGTGAAATAATGCGTGCAGAATTCTTTGTACAGA

>Mmu_dom_Ceacam13N1 (Mus musculus domesticus; western European house mouse) WGS LVXW01029994.1

TCTCTCTTTTAACATGCTGGCTGCATCCCACTACTTCTCATCTCACCATTAAAGCAGTGCCTCCCATTGCTGTTGAAGGGGAAAACGTTCTTCTGTTTGTGCATAACCTGCCAAAGAATGTTAAAGCCTTTTCCTGGTACTCGGGAGCTGCACCGTTCAAGTGTTGTGAAATTGCAAGTCATGTGATAGCTACCAATTTTACTGCAGTGGGACTTGCACACAGTGGTAGAGAGACAGTACTCAACAATGGATCTCTGTTGATCAAGAGTGTTACCAGAAAAGACTCAGGATACTACACTCTACGAACACTTGATTCAACCTCAAGACCTGAAATAATACGTGCAGAATTCTTTGTACACC

>Mmu_dom_Ceacam14N1 (Mus musculus domesticus; western European house mouse) WGS LVXW01029991.1

TCTCCCTTTTAACCTGCTGGCTGATTCCCACTACTTCCCAGCTCACCATCAAATCAGTGCCTCCCATTGCTGTTGAAGGGGAAAACGTTCTTCTGTTTGTGCATAACCTGCCGAAGAATGTTAAAGTCTTTTCCTGGTTCACAGGAGCTAGAGTGCTCAAGAGTTGTGAAATTGCAACTCATGTGATAGCTATCAATGCTACTGTGATCGGACTTTCACATAGTGGTAGAGAGACAGTGTTCAAAAATGGATCTCTGCTGATCAAGAGTGTCACCAGTAAAGACTCAGGATACTACACTCTACGGATACTTGATGCAAACTCAAGACCTAAAATAATACGTACAGAATTCTTTGTACACA

>Mmu_dom_Ceacam15N (Mus musculus domesticus; western European house mouse) WGS LVXW01029906.1

CCTCACTTTTAATCTGCTGGAACTGGTCCACTGCAGCACTGCTGACCTCTAAAGAAATGCGCTTCTCAGCTGCTGAAGGGGCAAAGGTTCTTCTCTCTGTTCCTGACCAGGAGGAGAACCTCCTCTCCTTTTCCTGGTACAAAGGGAAGGATGTAAATGAAAATTTTACAATTGCACATTATAAAAAGTCCAGCGATTCGCTTCAACTTGGAAAGAAAGTCAGCGGCAGGGAAGAAATCTATAAGGATGGCTCCATGATGCTCTGGGCCATCACCCTGGAAGACACGGGATTCTACACGTTACAAACCTTTAAAGCACACGGCCAACAGGAAGTAACACATGTCCATCTCCAAGTATACA

>Mmu_dom_Psg16N1 (Mus musculus domesticus; western European house mouse) WGS LVXW01029919.1

CCTCCCTTTTAGCCTGCTGGCTCCTGTCCACCACTGCCCAGGTCACCATTGAATCAGTGCCTTTCAATGTGGTTGAAGGAGAAAATGTCCTTCTTCGTGTTGACAATCTGCCAGAGAATCTTATAACCTTAGCCTGGTACAGAGGGCTGAGGAAAATTGTTGTATACACACTGAACACTAAAGTAAGTGTGATGGGGCAAATGTACAGTGGTAGAGAGATAGTGTCCAGCAACGGGTCCCTGTGGATCCACAATGTCACCCGGAAGGACACAGGACTCTACACCCTACGGACCGTAAATAGACGTGGAGAAATTGTATCAACATCATTCACGTACCTCTACGTGTACA

>Mmu_dom_Psg17N1 (Mus musculus domesticus; western European house mouse) WGS LVXW01030059.1 CCTCCCTCTTATCCTGCTGCCTCCTGCCCACCACTGCCAGAGTCACTGTGGAATTCTTACCTCCCCAAGTGGTTGAAGGAGAAAATGTTCTTCTACGCGTTGACAATCTGCCAGAGAATCTTCTAGGTTTTGTGTGGTACAAAGGGGTGGCAAGTATGAAGCTTGGAATTGCACTGTATTCACTGCAATATAATGTAAGTGTGACAGGGCTTAAGCACAGCGGTAGAGAGACACTGCACAGAAATGGGTCCCTGTGGATCCAAAATGTCACCTCGGAGGACACAGGATATTACACCCTTCGAACCGTAAGTCAACGTGGAGAACTGGTATCAGATACATCCATATTCCTTCAGGTGTACT

>Mmu_dom_Psg19N1 (Mus musculus domesticus; western European house mouse) WGS LVXW01030059.1

CCTCCCTCTTAACCTGCTGGTTCCTGCCCATCACTGCCCGAGTCACCATCGAATCCGTACCACCCAAATTGGTCGAAGGAGAAAATGTTCTTCTACGAGTAGACAATCTGCCAGAGAATCTTCGAGTCTTTGCCTGGTACAGAGGGGTAATAAAATTTAAGCTTGGAATTGCACTGTATTCACTGGACTATAACACAAGTGTGACAGGACCTGAGCACAGTGGTAGAGAGACATTGCACAGCAACGGGTCCCTGTGGATCCAAAGTGCCACCCGGGAAGACACAGGATATTACACGTTTCAAACCATAAGTAAAAATGGAAAAGTGGTATCAAATACATCCATGTTCCTTCAGGTGTACT

>Mmu_dom_Psg20N1 (Mus musculus domesticus; western European house mouse) WGS CAKLHL010000014.1

CCTCCCTCTTTACCTGCTGGCTTCTGTCCACCACTGCCAAGGTCACTATCCATTCACCGCTCCAAGTGGTTGAAGGACAAAACGTTTTTCTACGAGTTGACAATCTGCCAGAGGATCTTCTAGCTTTTGCCTGGTACAGAGGACTGAGAAATTGGAGGGTCGCAATTGCACTGCATTTAGTGGAGTATAATGCAAGTATGACAGGGCCTGAGCACAGTGATAGAGAGATATTGCACAGCAACGGGTCCCTGTGGATCCAAAATGTCACCCAGGAGGACACAGGATATTATACTCTTCAAACCATAAGTAAACATGGAAAACTGGTATCAAATACATCCACATTTCTTCAGGTGTACT

>Mmu_dom_Psg21N1 (Mus musculus domesticus; western European house mouse) WGS CAKLHL010000014.1

CCTCCCTCTTGACCTGCTGGCTTCTGTCCACCACTGCTAGTGTCACCATCCAGTCACCAAAACACGTAGTTGAAGGAGAGAATATTCTTCTACAAGTTGACAATCTGCCAGAGAATCTTCTAGCTTTTGCCTGGTACAGAGGACTGATAAATTGGAGGCTCACAATTGCTCTGCATTTTCTGGACTATAGCACAAGTATGACAGGGCCTGAGCACAGTGATAGAGAGATATTGTACAGCAACGGGTCCCTATGGATCCAAAATGTCACCCAGGAGGACACAGGATATTACATTTTTCAAACCATAAGTAACCATGGAAAACTGGAATCAAATACGTCCACATTTCTTCAGGTCTACT

>Mmu_dom_Psg22N1 (Mus musculus domesticus; western European house mouse) WGS LVXW01030053.1

CCTCCCTCTTAACCTGCTGGCTCTTGCCCATCACTGCCGGAGTCACCATCGAATCCGTACCACCCAAATTGGTTGAAGGAGAAAATGTTCTTCTACGAGTGGACAATCTGCCAGAGAATCTTCGAGTCTTTGTCTGGTATAGAGGGGTGACAGACATGAGCCTCGGAATTGCATTGTATTCACTTGACTATAGCACAAGTGTGACAGGACCTAAGCACAGCGGTAGAGAGACATTGTACAGAAACGGGTCCCTGTGGATCCAAAATGTCACCCGGGAAGACACAGGATATTACACTCTTCAAACCATAAGTAAAAATGGAAAAGTGGTATCAAATACATCCATATTCCTTCAGGTGAACT

>Mmu_dom_Psg23N1 (Mus musculus domesticus; western European house mouse) WGS CAKLHL010000014.1

CCTCCCTCTTGACCTGCTGGCTTCTGTCCACCACTGCTAGTGTCACCATCCAGTCACCACAACACGTAGTTGAAGGAGAGAATATTCTTCTACAAGTTGACAATCTGCCAGAGAATCTTCTAGCTTTTGCCTGGTACAGAGGACTGACAAATTGGAGGCTCACAATTGCTGTGTATTTACTGGACTATAGCACAAGTATGACAGGGCCTGAGCACAGTGATAGAGAGATATTGTACAGCAACGGGTCCCTATGGATCCAAAATGTCACCCAGGAGGACACAGGATATTACACTCTTCAAACCATAAGTAACCATGGAGAACTGGAATCAAATACATCCACATTTCTTCAGGTCTACT

>Mmu_dom_Psg24N1 (Mus musculus domesticus; western European house mouse) WGS LVXW01029921.1

CCTCCCTCTTAACCTGCTGGCTCCTGCCCACCACTACCCAAGTCGACATCGAATCCTTACCACCCCAAGTGGTTGAAGGAGAAAATGTTCTTCTACGGGTTGACAATCTGCCAGAGAATCTTCTAGGCTTTATCTGGTACAAAGGGGTGACAGACATGAGCCTCGGAATTGCACTGTATTCACTGACCTATAGCAGAGGTGTGACGGGACCTGTGCACAGTGGTAGAGAGACATTGTACCGAAATGGGTCCCTGTGGATTCAAAATGTCACCCAGGAGGACACAGGATTCTACACCCTACGAACCATAAGTAAACGTGGAGAAATTATATCAAATACATCCATGCACCTTCATGTGTACT

>Mmu_dom_Psg25N1 (Mus musculus domesticus; western European house mouse) WGS LVXW01030029.1

CCTCCATCTTAACCTACTGGCTCCTGCCCACCACTGCCAGAGTCATCATCCATTCTTTACCACTCCAAGTGGTTGAAGGAGAAAATGTTCTTCTACATGTTTACAATCTGCCAGAGAATCTTCTAGGCCTTGCCTGGTACAGAGGGTTGCTAAATTTGAAACTTGGAATTGCACTGTATTCACTACAATATAATGTAAGTGTGACTGGACCTGAGCACAGCGGTAGAGAGACATTGCACAGAAATGGGTCTCTGTGGATCCAAAATGTCACCCAGGAGGACACAGGATATTACACTCTTCGTACCATAAGTAAAAATGGAAAACTGGAATCAAATACATCCATGTTCCTTCAGGTGTACT

>Mmu_dom_Psg26N1 (Mus musculus domesticus; western European house mouse) WGS CAKLHL010000014.1

CCTCCCTCTTAACCTGCTGGTTCCTGCCCACCACTGCCAGAGTCACCATTGAATCCTTACCACCCCAAGTGGTTGAAGGAGAAAATGTTCTTCTACGTGTTGACAATATGCCAGAGAATCTTCTAGTGTTTGGCTGGTACAGAGGAATGACAAATTTGAGGCAAGCAATTGCACTGCATTCGCTGTACTATAGTGTAACGGTGAAGGGGCTGAAGCACAGCGGCAGAGAGACATTATACATCAACGGGACCCTGTGGATCCAAAATGTCACACAGGAGGACACAGGATATTACACTTTTCAAACCATAAGTAAACAAGGAGAAATGGTATCAAATACATCCCTGTACTTGCACGTGTACT

>Mmu_dom_Psg27N1 (Mus musculus domesticus; western European house mouse) WGS LVXW01030029.1

CCTCCCTCTTTACCTGCTGGCTCCTCTCCACTACTGCCCGAGTCACCATCCATTCACCGCTCCAAGTGGTTGAAGGAGAAAATGTTCTTCTACGAGTTGACAATCTGCCCGAGAATCTTCTAGCCTTTTCCTGGTATAGAGGCCTGAAAAATTGGCAGCTTGCAATTGCACTACATTTACTGGACTATAACACAAGTATGACAGGGCCTGATCACAGTGATAGAGAGATATTGTACAGCAATGGATCCCTATGGATCCAAAATGTCACCAAGGAGGACACAGGATATTATACTCTTCGAACCATAAGCAAACATGGAGAACTGGTATCAAATACATCCACATTTCTTCAGGTGTACT

>Mmu_dom_Psg28N1 (Mus musculus domesticus; western European house mouse) WGS CAKLHL010000014.1

CCTTCCTCTTAACCTGCTGGCACCTGCCCACCACTGCCAGAGTCACCATTGAATCCTTTCCACCCCAAGTGGTTGAAGGAGAAAATGTTCTTCTACGTGTTGACAATATGCCAGAGAATCTTCTAGTGTTTGGCTGGTACAGAGGAATGACAAATTTGAGACATGCAATTGCACTGTACTATAGTTTAACAGCGAAGGGGCTGAAGCACAGCGGCAGAGAGACATTATACATCAACGGGTCCCTGTGGATCCAAAATGTCACACAGGAGGACACAGGATATTACACCTTTCAAACCATAAGTAAACAAGGAGAAATGGTATCAAATACATCCCTGTACTTGCACGTGTACT

>Mmu_dom_Psg29N1 (Mus musculus domesticus; western European house mouse) WGS LVXW01029924.1

CCTCCTTCTTAACCTGCTGGTATCTGTCTACCACTTCCAAAGTCACCATTGAATTATTGCCATCCCAAGTGGTTGAAGGAGAAGATGTTCTTTTCCTTGTCAATAATCTGCCAGGGAATCTTACAGCCTTTGCCTGGTTTAAAGGGAGGACAAATAGGAAACATGGAATTGCACTGTATGCAGTGGCGTCTGACTTATATGTACACAGCGATAGAGAGACATTGTACAACAACGGATCCCTGATGATCCACAATGTTACCCAGAAGGACAGAGGTTATTACACCCTACGAACCTTCAATAAACATGCAGAAACTGTATCAACAACATTCACATTCCTCCACGTGAACC

>Mmu_dom_Psg30N1 (Mus musculus domesticus; western European house mouse) WGS LVXW01029994.1

CCTCATTTTTAACCTGTTGTCACCTGCCTGCCACTGCCCAAATAACCATTGAATTAGTGCCACCCCATGTGATTGAAGGAGAAAATGTTCTCATACGTGTCAACAATCTGCCAGAGAATCTTACAACCTTAGTCTGGTTCAGAGGAATGAGGATTAAGAGCCCTCAAATTGGACAATATACACTGGCCACTAATGTTACTGTGCTGGGGCCTGGTCACAGTGGTAGAGAAACTTTGTACAGCAATGGATCCCTGCAGATCTACAATGTCACCCAGGAGGACATAGGATTCTACAGCCTACGAGTCATGAATAGACATGGAAAAATTGTGTCAATAACATCCATATACCTCAACGTGTACT

>Mmu_dom_Psg31N1 (Mus musculus domesticus; western European house mouse) WGS LVXW01029994.1

CCTCCTTTTTAACCTGCTGTCACCTGCCTACCACTGCCCAAATAACCATTGAATTAGTGCCACCCCATGTGATTGAAGGAGAAAATGTTCTCATACGTATCAACAATCTGCCAGAGAATCTTACAACCTTAGCCTGGTTCAGAGGAATGAGGATTAAGAGCCCTCAAATTGGACAATATACACTGGCCACTAATGTTACTGTGCTGGGGCCTGGTCACAGTGGTAGAGAAACTTTGTACAGCAATGGATCCGTGCAAATCTACAATGTCACCAAAGAGGATATAGGATTCTACAGCCTACGAGTCATGAATAGACATGGAGAAATTGTGTCAATAACATCCATATACCTCAACGTGTACT

>Mmu_dom_Psg32N1 (Mus musculus domesticus; western European house mouse) WGS LVXW01029985.1

CTTTTCTTTTAACCTCTTGGTTCCTGCCCACCACTGTCCAAGTCACCATTGAATTAGTGCCACCACAAGTGGCTGAAGGAGAAAATGTCCTTATTATTGTTTACAGTCTGCCAGAGGATCTTACAGCCATAGCCTGGTTCAAAGGAGTGACAAATATGAACCTCGGAATTGCATTGTATGCACTGGCCTCTAACATCAGTGTGAAAGGGCCCGAACACAGTGGTAGAGAGACAGTGTTCAGCAATGGATCCCTGTTGCTTCACAATGTCACCCAGAAGGACACAGGATTCTATACTATACGGACCTTAAATAGACATGGAAAAATTGTATCCACAACATCCATATACCTCCACGTGTACA

>Mmu_mus_Ceacam9N (Mus musculus musculus; eastern European house mouse) WGS LVXU01014974.1

CCTTCCTCTTAACCTGCTGGAATGCACCCGCCGCTGCCGAGCTCACTATTGAATTAGTGCCACCCATGGTTGCCGAAGGCGGAAACTCCGTTCTGTTTGTGCATGAAATGCCACTGAACGTCCAGGCGTTTTACTGGTACAAACAGAGAGATTCGACGAAAAGCTACGAAGTCGCACGGTACTTAACACCCACGAACCAAAGTTCGAAGATGCCTCAGCACAGTGATAGGAAAACCGTATTCTACAGTGGATCCCTGCTGATCAGAAACGTCACCAAGGCTGACAGTGGAGTCTATACCTTACTAACATTTAACACGGAAATGGAAAGCGAATTAACACATGTGCATCTGGAAGTGCAGG

>Mmu_mus_Ceacam11N1 (Mus musculus musculus; eastern European house mouse) WGS LVXU01014997.1

TCTCCCTTTTAACCTGCTGGCTGATTCCAACTACCACCCAGATCACCATTGAATCAGTGCCTCCCATTGCTGTTGAAGGGGAAAATGTTCTTCTGTTTGTGCATAACTTGCCAGAGAATGTTAAAGCCCTTTCCTGGTACACAGGAGTTAAACCACTCAAGAATTGTGAAATTGCAAGTCATGTGATAGCTACCAATTCTACTGTGGTGGGACCTGCACACAGTGGTAGAGAGATTGTACTCAAAAATGGATCTCTGCTGATCAAGAGTACCACCAGAAAAGACTCAGGTTACTACACTCTACAAATACTTGATACAACCTCAAGACCTGAATTAATACGTGCAGAATTCTTTGTTCACA

>Mmu_mus_Ceacam12N1 (Mus musculus musculus; eastern European house mouse) WGS LVXU01014997.1

TCTCCATTTTAACATGCTGGCTGCTTCCCACTACTGCCCAGATAACTATTGAATCAGTGCCTCCCATTGCTGTTGAAGGGGATAATGTTCTTCTGTTTGTGCAAAACTTGCCAGAGAATGTTCAAACCCTTTCCTGGTACACAGGAGGTAAACCGCTCAAGATGTTTGAAATTGCAAGACACGTGATAGCTACCAATTCTAGTGTGATGGGACCTGCACACAGTGGTAGAGAGACAATGCTCAATAATGGATCTCTGATGATCAAGAATGTCACCAGAAAAGACTCAGGATACTACACTCTACAAATACTTGATACAACCTCAAGACGTGAAATAATGCGTGCAGAATTCTTTGTACAGA

>Mmu_mus_Ceacam13N1 (Mus musculus musculus; eastern European house mouse) WGS LVXU01014997.1

TCTCTCTTTTAACATGCTGGCTGCATCCCACTACTTCTCATCTCACCATTAAAGCAGTGCCTCCCATTGCTGTTGAAGGGGAAAACGTTCTTCTGTTTGTGCATAACCTGCCAAAGAATGTTAAAGCCTTTTCCTGGTACTCGGGAGCTGCACCGTTCAAGTGTTGTGAAATTGCAAGTCATGTGATAGCTACCAATTTTACTGCAGTGGGACTTGCACACAGTGGTAGAGAGACAGTACTCAACAATGGATCTCTGTTGATCAAGAGTGTTACCAGAAAAGACTCAGGATACTACACTCTACGAACACTTGATTCAACCTCAAGACCTGAAATAATACGTGCAGAATTCTTTGTACACC

>Mmu_mus_Ceacam14N1 (Mus musculus musculus; eastern European house mouse) WGS LVXU01014993.1

TCTCCCTTTTAACCTGCTGGCTGATTCCCACTACTTCCCAGCTCACCATCAAATCAGTGCCTCCCATTGCTGTTGAAGGGGAAAATGTTCTTCTGTTTGTGCATAACCTGCCGAAGAATGTTAAAGTCTTTTCCTGGTTCACAGGAGCTAGAGTGCTCAAGAGTTGTGAAATTGCAACTCATGTGATAGCTATCAATGCTACTGTGATCGGACTTTCACATAGTGGTAGAGAGACAGTGTTCAAAAATGGATCTCTGCTGATCAAGAGTGTCACCAGTAAAGACTCAGGATACTACACTCTACGGATACTTGATGCAAACCCAAGACCTGAAATAATACGTACAGAATTCTTTGTACACA

>Mmu_mus_Ceacam15N (Mus musculus musculus; eastern European house mouse) WGS LVXU01014972.1

CCTCACTTTTAATCTGCTGGAACTGGTCCACTGCAGCACTGCTGACCTCTAAAGAAATGCGCTTCTCAGCTGCTGAAGGGGCAAAGGTTCTTCTCTCTGTTCCTGACCAGGAGGAGAACCTCCTCTCCTTTTCCTGGTACAAAGGGAAGGATGTAAATGAAAATTTTACAATTGCACATTATAAAAAGTCCAGCGATTCGCTTCAACTTGGAAAGAAAGTCAGCGGCAGGGAAGAAATCTATAAGGATGGCTCCATGATGCTCCGGGCCATCACCCTGGAAGACACGGGATTCTACACGTTACAAACCTTTAAAGCACACGGCCAACAGGAAGTAACACATGTCCATCTCCAAGTATACA

>Mmu_mus_Psg16N1 (Mus musculus musculus; eastern European house mouse) WGS LVXU01014974.1

CCTCCCTTTTAGCCTGCTGGCTCCTGTCCACCACTGCCCAGGTCACCATTGAATCAGTGCCATTCAATGTGGTTGAAGGAGAAAATGTCCTTCTTCGTGTTGACAATCTGCCAGAGAATCTTATAACCTTAGCCTGGTACAGAGGGCTGAGGAAAATTGTTGTATACACACCGAACACTAAAGTAAGTGTGATGGGGCAAATGTACAGTGGTAGAGAGATAGTGTCCAGCAACGGGTCCCTGTGGATCCACAATGTCACCCGGAAGGACACAGGACTCTACACCCTACGGACCGTAAATAGACATGGAGAAATTGTATCAACATCATTCACGTACCTCTACGTGTACA

>Mmu_mus_Psg17N1 (Mus musculus musculus; eastern European house mouse) WGS LVXU01015001.1

CCTCCCTCTTATCCTGCTGCCTCCTGCCCACCACTGCCAGAGTCACTGTGGAATTCTTACCTCCCCAAGTGGTTGAAGGAGAAAATGTTCTTCTACGCGTTGATAATCTGCCAGAGAATCTTCTAGGTTTTGTGTGGTACAAAGGGGTGGCAAGTATGAAGCTTGGAATTGCACTGTATTCACTGCAATATAATGTAAGTGTGACAGGGCTTAAGCACAGTGGTAGAGAGACACTGCACAGAAATGGGTCCCTGTGGATCCAAAATGTCACCTCGGAGGACACAGGATATTACACCCTTCGAACCGTAAGTCAACGTGGAGAACTGGTATCAGATACATCCATATTCCTTCAGGTGTACT

>Mmu_mus_Psg18N1 (Mus musculus musculus; eastern European house mouse) WGS CAKLHR010000018.1

CCTCCCTCTTAACCTGCTGGCTCCTGCCCACCACTGCCAGAGTCACCATTGAATCCTTACCACCCCAAGTGTACGAAGGAGAAAATGTTCTTCTACGTGTTGACAATATGCCAGAGAATCTTCTAGTGTTTGGCTGGTACAGAGGAATGACAAATTTGAGGCAAGCAATTGCACAGCATTGGCTGTACTACTATAGTGTAATGGTGAAGGGGCTGAATCACAGCGGCAGAGAGATATTATACATCAACGGGTCCCTGTGGATCCAAAATGTCACACAAGAGGACACAGGATATTACACTTTTCAAACCATAAGTAAACGAGGAGAAATAGTATCAAATACATCCCTGTACTTGCACGTGTACT

>Mmu_mus_Psg19N1 (Mus musculus musculus; eastern European house mouse) WGS LVXU01015001.1

CCTCCCTCTTAACCTGCTGGTTCCTGCCCATCACTGCCCGAGTCACCATCGAATCCGTACCACCCAAATTGGTCGAAGGAGAAAATGTTCTTCTACGAGTAGACAATCTGCCAGAGAATCTTCGAGTCTTTGCCTGGTACAGAGGGGTAATAAAATTTAAGCTTGGAATTGCACTGTATTCACTGGACTATAACACAAGTGTGACAGGACCTGAGCACAGTGGTAGAGAGACATTGCACAGCAACGGGTCCCTGTGGATCCAAAGTGCCACCCGGGAAGACACAGGATATTACACGTTTCAAACCATAAGTAAAAATGGAAAAGTGGTATCAAATACATCCATGTTCCTTCAGGTGTACT

>Mmu_mus_Psg20N1 (Mus musculus musculus; eastern European house mouse) WGS LVXU01015001.1

CCTCCCTCTTTACCTGCTGGCTTCTGTCCACCACTGCCAAGGTCACTATCCATTCACCGCTCCAAGTGGTTGAAGGACAAAACGTTTTTCTACGAGTTGACAATCTGCCAGAGGATCTTCTAGCTTTTGCCTGGTACAGAGGACTGAGAAATTGGAGGGTCGCAATTGCACTGCATTTAGTGGAGTATAATGCAAGTATGACAGGGCCTGAGCACAGTGATAGAGAGATATTGCACAGCAACGGGTCCCTGTGGATCCAAAATGTCACCCAGGAGGACACAGGATATTATACTCTTCAAACCATAAGTAAACATGGAAAACTGGTATCAAATACATCCACATTTCTTCAGGTGTACT

>Mmu_mus_Psg21N1 (Mus musculus musculus; eastern European house mouse) WGS LVXU01015001.1

CCTCCCTCTTGACCTGCTGGCTTCTGTCCACCACTGCTAGTGTCACTATCCAGTCACCACAACACGTAGTTGAAGGAGAGAATATTCTTCTACAAGTTGACAATCTGCCAGAGAATCTTCTAGCTTTTGCCTGGTACAGAGGACTGATAAATTGGAGGCTCACAATTGCTCTGCATTTCCTGGACTATAGCACAAGTATGACAGGGCCTGAGCACAGTGATAGAGAGATATTGTACAGCAACGGGTCCCTATGGATCCAAAATGTCACCCAGGAGGACACAGGATATTACATTTTTCAAACCATAAGTAACCATGGAGAACTGGAATCAAATACGTCCACATTTCTTCAGGTCTACT

>Mmu_mus_Psg22N1 (Mus musculus musculus; eastern European house mouse) WGS LVXU01015001.1

CCTCCCTCTTAACCTGCTGGCTCTTGCCCATCACTGCCGGAGTCACCATCGAATCCGTACCACCCAAATTGGTTGAAGGAGAAAATGTTCTTCTACGAGTGGACAATCTGCCAGAGAATCTTCGAGTCTTTGTCTGGTATAGAGGGGTGACAGACATGAGCCTCGGAATTGCATTGTATTCACTTGACTATAGCACAAGTGTGACAGGACCTAAGCACAGCGGTAGAGAGACATTGTACAGAAACGGGTCCCTGTGGATCCAAAATGTCACCCGGGAAGACACAGGATATTACACTCTTCAAACCATAAGTAAAAATGGAAAAGTGGTATCAAATACATCCATATTCCTTCAGGTGAACT

>Mmu_mus_Psg24N1 (Mus musculus musculus; eastern European house mouse) WGS LVXU01014976.1

CCTCCCTCTTAACCTGCTGGCTCCTGCCCACCACTACCCAAGTCGACATTGAATCCTTACCGCCCCAAGTGGTTGAAGGAGAAAATGTTCTTCTACGGGTTGACAATCTGCCAGAGAATCTTCTAGGCTTTATCTGGTACAAAGGGCTGACAGACATGAGCCTCGGAATTGCACTGTATTCACTGACCTATAGCAGAGGTGTGACGGGACCTGTGCACAGTGGTAGAGAGACATTGTACCGAAATGGGTCCCTGTGGATTCAAAATGTCACCCAGGAGGACACAGGATTCTACACCCTACGAACCATAAGTAAACGTGGAGAAATTATATCAAATACATCCATGCACCTTCATGTGTACT

>Mmu_mus_Psg25N1 (Mus musculus musculus; eastern European house mouse) WGS LVXU01015001.

CCTCCATCTTAACCTACTGGCTCCTGCCCACCACTGCCAGAGTCATCATCCATTCTTTACCACTCCAAGTGGTTGAAGGAGAAAATGTTCTTCTACATGTTTACAATCTGCCAGAGAATCTTCTAGGCCTTGCCTGGTACAGAGGGTTGCTAAATTTGAAACTTGGAATTGCACTGTATTCACTACAATATAATGTAAGTGTGACTGGACCTGAGCACAGCGGTAGAGAGACATTGCACAGAAATGGGTCTCTGTGGATCCAAAATGTCACCCAGGAGGACACAGGATATTACACTCTTCGTACCATAAGTAAAAATGGAAAACTGGAATCAAATACATCCATGTTCCTTCAGGTGTACT

>Mmu_mus_Psg27N1 (Mus musculus musculus; eastern European house mouse) WGS CAKLHR010000018.1

CCTCCCTCTTTACCTGCTGGCTCCTCTCCACTACTGCCCGAGTCACCATCCATTCACCGCTCCAAGTGGTTGAAGGAGAAAATGTTCTTCTACGAGTTGACAATCTGCCCGAGAATCTTCTAGCCTTTTCCTGGTATAGAGGCCTGAAAAATTGGCAGCTTGCAATTGCACTACATTTACTGGACTATAACACAAGTATGACAGGGCCTGATCACAGTGATAGAGAGATATTGTACAGCAATGGATCCCTATGGATCCAAAATGTCACCCAGGAGGACACAGGATATTATACTCTTCGAACCATAAGTAAACATGGAGAACTGGTATCAAATACATCCACATTTCTTCAGGTGTACT

>Mmu_mus_Psg28N1 (Mus musculus musculus; eastern European house mouse) WGS LVXU01015001.1

CCTTCCTCTTAACCTGCTGGCACCTGCCCACCACTGCCAGAGTCACCATTGAATCCTTACCACCACAAGTGGTTGAAGGAGAAAATGTTCTTCTACTTGTTGACAATATGCCAGAGAATCTTCTAGTGTTTGGCTGGTACAGAGGAATGACAAATTTGAGGCAAGCAATTGCACTGCATTCGCTGTACTATAGTGTAACGGCGAAGGGGCTGAATAACAGCGGCAGAGAGACATTATACATCAACGGGTCCCTGTGGATCCAAAACGTCACACAGGAGGACACAGGATATTACACTTTTCAAACCATAAGTAAACAAGGAGAAATGGTATCAAATACATCCCTGTACTTGCACGTGTACT

>Mmu_mus_Psg29N1 (Mus musculus musculus; eastern European house mouse) WGS LVXU01014977.

CCTCCTTCTTAACCTGCTGGTATTTGTCTACCACTTCCAAAGTCACCATTGAATTATTGCCATCCCAAGTGGTTGAAGGAGAAGATGTTCTTTTCCTTGTCAATAATCTGCCAGGGAATCTTACAGCCTTTGCCTGGTTTAAAGGGAGGACAAATAGGAAACATGGAATTGCACTGTATGCAGTGGTGTCTGACTTATATGTACACAGCGATAGAGAGACATTGTACAACAACGGATCCCTGATGATCCACAATGTTACCCAGAAGGACAGAGGTTATTACACCCTACGAACCTTCAATAAACATGCAGAAACTGTATCAACAACATTCACATTCCTCCACGTGAACC

>Mmu_mus_Psg30N1 (Mus musculus musculus; eastern European house mouse) WGS CAKLHR010000018.1

CCTCCTTTTTAACCTGCTGTCACCTGCCTACCACTGCACAAATAACTATTGAATTAGAGCCACCCCAAGTGATTGAAGGAGAAAATGTTCTCATACGTGTCAACAATTTGACAGAGAATCTTATAACCTTAGCCTGGTTCAGAGGAATGAGGATTAAGAGCCCTCAAATTGGACAATATACACCGGCCACTAAAGTTACTGTGCTGGGTCCTGGTCACAGTGGTAGAGAAACTTTGTACAGCAATGGATCCCTGCAGATCTACAATGTCACCCAGGAGGACATAGGATTCTACAGCCTACGAATCATAAATAAACATGCAGAAATTGTGTCAATAATATCCATATACCTCAATGTGTACT

>Mmu_mus_Psg31N1 (Mus musculus musculus; eastern European house mouse) WGS LVXU01014995.1

CCTCATTTTTAACCTGTTGTCACCTGCCTGCCACTGCCCAAATAACCATTGAATTAGTGCCACCCCATGTGATTGAAGGAGAAAATGTTCTCATACGTGTCAACAATCTGCCAGAGAATCTTACAACCTTAGCCTGGTTCAGAGGAATGAGGATTAAGAGCCCTCAAATTGGACAATATACACTGGCCACTAATGTTACTGTGCTGGGGCCTGGTCACAGTGGTAGAGAAACTTTGTACAGCAATGGATCCGTGCAAATCTACAATGTCACCAAAGAGGATATAGGATTCTACAGCCTACGAATCATGAATAGACATGGAGAAATTGTGTCAATAACATCCATATACCTCAACGTGTACT

>Mmu_mus_Psg32N1 (Mus musculus musculus; eastern European house mouse) WGS LVXU01014993.1

CTTTTCTTTTAACCTCTTGGTTCCTGCCCACCACTGTCCAAGTCACCATTGAATTAGTGCCACCACAAGTGGCTGAAGGAGAAAATGTCCTTATTATTGTTTACAGTCTGCCAGAGGATCTTACAGCCATAGCCTGGTTCAAAGGAGTGACAAATATGAACCTCGGAATTGCATTGTATGCACTGGCCTCTAACATCAGTGTGAAAGGGCCCGAACACAGTGGTAGAGAGACAGTGTTCAGCAATGGATCCCTGTTGCTTCACAATGTCACCCAGAAGGACACAGGATTCTATACTATACGGACCTTAAATAGACATGGAAAAATTGTATCCACAACATCCATATACCTCCACGTGTACA

>Mna_Ceacam9N (Mastomys natalensis; African soft-furred rat) WGS JADRCE010221256.1

CCTCCCTCTTAACCTGCTGGAATGCACCTGCCGCTGCCGAGCTCACTATTGAATTAGTGCCACCCATGGTTGCGGAAGGTGGAAATTCCGTTCTGTTTGTACATGAAATGCCACTGAATGTCCAGGCGTTTTACTGGTACAAACAGAGAGATTCGACGAAGAGCTATGAAGTTGCACGGTACTTAACACCCACTAACGAAAGTTCGAAGATGCCTCAGCACAGTGATAGGAAAACCGTATTCTACAGTGGATCCCTGCTGATCAGAAATGTCACCCAGGCCGACAGTGGAGTCTACACCTTACTAACATTCAACACAGAAATGGAAAGTGAATTAACACACGTGCATCTGGAAGTACGCA

>Mna_Ceacam11aN1 (Mastomys natalensis; African soft-furred rat) WGS JJAJTUV010001927.1

TTTCCCTTTTAACCTGCTGGCTGCTTCCCACTACTGCCCAGCTCACCATTGAATCAGTGCCTCCCATTGCTATTGAAGGGGAAAATGTTCTTGTGCTTGTGCAAAACCTGCCAAAGAATGTTAAAGCCCTTTCCTGGTACAGAGGAGATAAACCACTCAATAATTTTGAAATTGCAAGACATGATATAGCTACCAATTCTAGTGTGGTGGGACCTGCAAACAGTCATAGAGAAACAGTACTCAACAGTGGATCTCTGCTGATCAAGAGTGTAACCAGAAAAGATTCTGGATACTACACCCTACAAATACTTCATAGAACCTCAAGACCTGAAATAATGCGTGCAGAATTCTTTGTACAGA

>Mna_Ceacam11bN1 (Mastomys natalensis; African soft-furred rat) WGS JJAJTUV010001927.1

TTTCCCTTTTAACCTGCTGGCTGCTTCCCATTACTACTCAGCTCACCATTGAATCAGTGCCTCCCATTGCTGTTGAAGGGGAAAATGTTCTTTTGTTAGTGCATAATTTGCCAAAGAAGGTTAAAGTCCTTTCATGGTACACAGGAGATAAACTGCTGAAGAACTGTGAAATTGCAAGACATGTGATAGATACCAATTCTAGTGTGGTGGGACTTGCACACAGTGGTAGAGAGACAGTACTCAACAATGGATCTCTGCTGATTAAGAGTGTCACCAGAAAAGACTCAGGATACTACACCCTACAAATACTTGATTCAACCTCAAGATCTGAAATAATACATGCAGAATTCTTTGTACACA

>Mna_Ceacam11cN1 (Mastomys natalensis; African soft-furred rat) WGS JJAJTUV010001927.1

TTTCCCTTTTAACCTGCTGGCTGCTTCCCACTACTGCCAAGCTCACCATTGAATCAGTGCCTCCCATTGCTGTTCAAGGGGAAAATGTCCTTCTGTTAGTGCATAATTTGCCAAAGAATGTTAAAGCCCTTTCGTGGTACACAGGAGTTGCAGCACTCAAGAGTTGTGAAATTGCAAGACATGATATAGCTACCAATTCTAGTGTGGTGGGACTTGCACACAGTGGTAGAGAGACAGTGCTCAACAATGGATCTCTGGTGATCAAGAGTGTCACCAGAAAAGACTCAGGATACTACACCCTACAAATACTTGATTCAGCCTCAAGACCTGAAATAATACATGCAGAATTCTTTGTACACA

>Mna_Ceacam12N1 (Mastomys natalensis; African soft-furred rat) WGS JADRCE010270839.1

TCTCCCTTTTAATCTGCTGGCTGCTTTCCACTACTGACCAGCTCACTATTGAATCAGTGCCTCCCATTGCTGTTGAAGGGGAAAATGTTTTGCTATTTGTGCATGACCTGCCAGAGAATGTTCAAGCCCTTTCCTGGTACACAGGAGGTAAACCACTCAAGAGGTTTGAAATTACAAGACATGTGATAGCTACCAATTCTAGTGTGATGGGACCTGCACACAGTGGTAGAGAGACAATACTCAACAATGGATCTTTGCTGATCAAGAGTGTCACCAGAAAAGACTCAGGATACTACACTCTAAAAATACGTGATACAACCTCAAGACGTAAAATAACACGTGCAGAATTCTTTGTACAGG

>Mna_Ceacam13aN1 (Mastomys natalensis; African soft-furred rat) WGS JADRCE010249454.1

TCTCCCTTTTAACCTGCTGGCTGCTTCCCACCACTTCCAAGCTCACCATAAGATCAATGCCTCCCATTGCTGTTGAAGGGGAAAATGTTCTTCTGTTTGTGCATAACCTGCCGAAGAATGTTAAAGCCTTTTCCTGGTACACAGGACCTGCACCATTCAAGTGTTGTGAAATTGCAAGTCATGTGATAGCAACCAATTTTACTGTGGCAGGACTTGCACACAGTGGTAGAGAGACAGTACTCAACAATGGATCTCTGCTGATCAAAAGTGTCACCAGAAAAGACTCAGGATACTACACTCTACGAACACTTGACTCAACCTCAAGACCTGAAATAATACATACTGAATTCTTTGTACACA

>Mna_Ceacam13bN1_P (Mastomys natalensis; African soft-furred rat) WGS JADRCE010248668.1

TCTCCCTTTTAATCTGTTGGCTGCTTTCCACTACTTCCCAGTTCACCATTAAATCAGTGTCTCCCATTGCTGTTGAAATGGAAAATTTTCCTCTGTTTGTGTATAACCTGAAGAATGTTAAAGCCTTTTCCTGGTACACAGGAGTTACCATGCTCAAGGGCTATGAAATTGCAAGTCATGTGATAGCTACCAATTTTACTGTAGTGGGGCTTGCACACAGTGGTAGAGAGACAGTACTCAACAATGGCTCTCTGCTGATCAAGAGTGTCACCAGAAAAGATTCAGGATACTACACTCTATGATCACTTGATGCAACCTTCAAGACCGGAAGTAATAAGTGCAGAATTCTTTGTACACA

>Mna_Ceacam14N1 (Mastomys natalensis; African soft-furred rat) WGS JADRCE010265771.1

TCTCACTTTTAATTTGCTGGCTGCTTCCCAGTACTTCCCAGCTCACCATTAAATCAGTGCCTCCAATTGCTGTTGAAGGGGAAAATGTTCTTCTGTTTGTGCATAACCTGCCGAAGAATGTTAAAGCCTTTTCCTGGTACACAGGAGTTAAAGCTATCAAGAGTTGTGAAATTGCAAGTCATGTGATTGCTACCAAATTTACTGTGGTGGGACCTGCACACAGTGGTAGAGAGACACTATTCAACAATGGATCTTTGCTGATCAAGAGTGTCACCAGAAAAGACTCAGGATACTACACTCTACAAATACTTGGTGCAACCTCAAGACGTAAAATAATACGTGCAGAATTCTTTGTGCACA

>Mna_Ceacam15N (Mastomys natalensis; African soft-furred rat) WGS JADRCE010098446.1

CCTCACTTTTATACTGCTGGAGCTCACCCACGGTGGCACTGCAAACATCTAAAGAAATGCGCTTCTCGGCTGCTGAAGGGGCAAAGGTTCTTCTCTCTGTTCCTGACCAGGAAGAGGATCTCCTCTCCTTTTCCTGGTACAAAGGGAAGGATGTAAATAAAAATTTTACAATTGCACATTACAAAAAGTCCAGCGATTCACTTCAGCTTGGAAAGAATGTCAGCGGCAGGGAAGAAATCTATAAGGATGGCTCCATGATGCTCCAGGCCGTCACCCAGGAAGACACGGGATTCTACACTTTAGAAACCTTTAAAGCACACGATCAACAGGAGATAACATATGTCCATCTCCAAGTATACA

>Mna_Psg36N1 (Mastomys natalensis; African soft-furred rat) WGS JAJTUV010001803.1

CCTCCCTCTTAACTTGCTGGCTCCTGCCCACCACTGCCCAAGTTGCCATCGAATCCTTACCACCCCAAGTGGTTGAAGGAGAAAATGTTCTTCTACGTGTTGACAATTTGCCAGAGAATCTTCTAGCCTTTGTCTGGTACAGAGGGGTGACAAATATGAGTCTTGGAATTGCACTGTATTCACTGACCTATAGAGTAACTGTGACGGGACCCGTGCACAGTGGTAGAGAGACATTGTACAGCAACGGGTCCCTGTGGATCCAAAATGTCACCCAGAAGGACACAGGATTCTACACCCTACTAACCATAAGTAAAAATGGAGAAATTGTATCAAATACAACAATGCACCTTCAAGTGTACT

>Mna_Psg36LN1 (Mastomys natalensis; African soft-furred rat) WGS JADRCE010276561.1

CCTCCCTCTTAACCTGCTGGCTCCTGTCCACTACTGCCCAAATTGACATCAAATCTTTACCACCCCAAGTGGTTGAGGGAGAAAATGTTCTTCTATGCGTTGAAAATCTGCCAGAGGATCTTATAGCCTTTGTCTGGTACAAAGGGGTGACAGACATGAGCCTCGGAATTGCACTGTATTCACTGACCTACAGCGTAAGTGTGACGGGGCCTGTGCACAGTGGTAGAGAGACACTGTACCGCAACGGGTCCCTGTGGATCCAAAATGTCACCCAGGAGGACACAGGATTCTACACCCTACGAACCATAAGTAAACGTGGAGAAATTGTATCAAACACGTCGATGCACCTTCTCGTGTACT

>Mna_Psg37N1 (Mastomys natalensis; African soft-furred rat) WGS JADRCE010169831.1

CCTCCCTCTTAACCTGCTGGCTCCTGTCCACCACTACCAGTGTCACCATCGAATCCTTACCTCCCAAAGTGGTTGAAGGAGAAAATGCTCTTCTACACGTTGACAATCTGCCAGAGAATCTTCTAGTCTTTGCCTGGTACAGAGAAGTGACAAGTATGAAGCTTGTAATTGGACTGTATTTACCAGATTACAGAACAAGTGTAAAGGGGCCTGAGCACAGTGGTAGAGAGATATTGTACAGCAACGGGTCCCTGTGGATCCAAAATGTCACCAGAGAGGACACAGGATATTACACTCTTCGAACCAAAAGCAAAAATGGAGAAATTGTATCAAATACATCTGTGTACCTTCAGGTGTACT

>Mna_Psg38N1 (Mastomys natalensis; African soft-furred rat) WGS JADRCE010091458.1

CCTCCCTTTTAACCTGCTGCCTCCTGACCACTGCCCAGGTCACCATTGAATCGGTGCCATTCAATGTGGTTGAAGGAGAAAACGTTCTTCTTCTTGTCGACAATCTGCCAGAGAATCTTCTAGCCTTAGCATGGTACAGGGGGCTGAGGAAAATCATTGTATACACACTGAACACTAAAGAGAGTGTGATGGGGCCTATTAACAGTGGCAGAGAGACAGTGTCCAGCAACGGGTCCCTGTGGATCCACAATGTCACCCAGAAGGACACAGGATTCTACACCTTACGAACCGTAAATAGACGTGGAGAAATTGTGTCAACCACATCCACGTACCTCTACGTGTACT

>Mna_Psg39N1 (Mastomys natalensis; African soft-furred rat) WGS JADRCE010207999.1

CCTCCATCTTAACATGCTGGCTCCTGCCCACCACGGCCCGAGTCACCATGGAATCCTTACCACCCAAAGTGGTTGAAGGAGAAAATGTTCTTCTAAGTGTTGACGGTCTGCCAGAGAATCTTGTAGCATTTGTCTGGTACAAAGGGGTGACAGACATGAGCCTCGGAATTGCACTGTATTCACTGACATATAGAAGAAGTGTGACGGGGCCTGTGCATAGTGGTAGAGAGATATTGTACAGCAACGGATCCCTGTGGATCAAAAATGTCACCCAGGAGGACACAGGATTCTACACTCTTCGAACTATAAGTAAACATGGAGAAATGGTGTTAAATGTATCCACGTACCTTGAGGTGTACT

>Mna_Psg40N1_P (Mastomys natalensis; African soft-furred rat) WGS JADRCE010004438.1

ACTCCCTTTTAACCTACTGGTACTTTTCTACAACTTCCCAAGTCACCATTGAATTAGTGCTACCCCAAGTGGTTGAAGGAGAAGATGTCCTATTCCTTCTCCACAAACTGCCAGAAATTCTTATGTCCTTAGGCTGGTTCAAAGGGATGACAGTTATAAAAACGTGGAATTGCACTGTATGCAACAAACACTAAAATATGACAGGGCCCATGTACAGTGCTAGAGAGACCTTGTACAGAAACGGGTCCCTGTTGATCCACAATGTCACCCAAGGACACAGGATTCTATACCTTATGAACCTTAAATAGACTTGGAGATATTGTGCCAACATCCACGTTCCTCTATA

>Mna_Psg41N1 (Mastomys natalensis; African soft-furred rat) WGS JADRCE010133267.1

CTTCCTTTTTCACCTGCTTGCTCCTGCCCACCACTGCCCAGGTCACCATTGAATCAGTGCCACTCCACGTGGTTGAAGGAGAAAATGTTCTTTTTCTCGTACACAATTTGCCAGAGAATCTTATAGCCTTAGTCTGGTTAAGAAGACTGGGGAAAATGAACCATGCAATTGGCCTATATGCAATGAACACTAAAATAAGTGTGATGGGGCCCATGAACAGCGGTAGAGAGACAGTGTCCAGCAACGGGTCCCTGTGGATCCGCAATGTCACCCAGAAGGACACAGGATTCTACATCTTACAAACCATAAATAGACGTGGAGAAATTGTCTCAAGAACACCCATGTACCTCTACGTGTACA

>Mna_Psg42N1 (Mastomys natalensis; African soft-furred rat) WGS JADRCE010248073.1

CCTCCCTTTTAACATGCTGGCACCTGTCTACCACTTCCAAAGTCACCATTGAATTATTGCCACCCCACGTGGTTGAAGGGGAAGATGTTCTTTTCCTTGTCCAAAATCTGCCAGAGGATCTTTCAGCCTTTGCCTGGTTTAAAGGGAGGACAAATAAGAAACATGGAATTGCACTATATGCAGTGGCCTCTGACATACATGTACATAGCGATAAAGAGACATTGTATAGCAATGGATCCCTGATGATCCACAACATCACCCAGAAGGACAGAGGTTACTATACCCTACGAACCTTCAATAAACATTCAGAAACTGTATCAACAACATCCACATTCCTCCATGTGAACC

>Mna_Psg42LN1 (Mastomys natalensis; African soft-furred rat) WGS JAJTUV010001803.1

CCTCCCTTTTAACCTGTTGGCACCTGTCTACCACTTCTGAAGTCACCATTGAATTAGTGCCACCACAAGTGGTTGAAGGGGAAGATGTTCTTTTCCTTGTCCATAATCTGCCAGAGAATCTTACAGCCTTTGCTTGGTTTAAAGGCAGGACAAATATGAAACGAGGAATTGCACTGTATGCATTTGCCTCTGACTTACATGTACACAGTGATAGAGAGACACTGTACAGCAATGGATCCATGATTATCCACAATATCACCCAGAAGGACAGAGCTTATTATACCCTACGAACCTTCAATAGACATGCAAAAACTGTATCAACAACAACCACATTCCTCCATGTGAACC

>Mna_Psg43N1 (Mastomys natalensis; African soft-furred rat) WGS JADRCE010107709.1

CTTTTCTTTTAATCTCCTGGTTCCTTCCCAACACTGTCCAAGTCACCATTGAATTAGTGCCACCCCAAGTGGCTGAAGGAGAAAATGTCCTTCTTCTTGTTTACAATCTGCCAGAGAATCTTATAGCCATAGCCTGGTTCAAAGGAGTGACAAATACGAACCTCGGAATTGTGTTGTATGCACTGGCCTCTAACATTAGTGTGACAGGGCCTGAACACAGTGGTAGAGAGACAATGCACAGGAATGGATCCCTGCTGCTTCACAATGTCACCCAGAAGGACACAGGATTCTATACTCTACGGACCTTTAATAGACATGGAAAAATTGTATCAACAACATCCATATACCTCCATGTGTACA

>Mna_Psg44N1 (Mastomys natalensis; African soft-furred rat) WGS JADRCE010292829.1

CCTCCTTTTTATCCTGCTGGCATCTGCCTACCACTGCCCAAATAACCATTGAATTAGTGCCTCCCCATGTGATTGAAGGAGAAAATGTTCTCATACGTATCAACAATCTGCCAGACAATCTTATAACCTTAGCCTGGTTCAGAGGGACGAGGATTAAGAGCCCTCAAATTGGACAATATACACTGGCCACTAATGTGACTGTTGTGGGGCCTGGTCACAGTGGTAGAGAGACTTTGTACAGCGATGGATCCCTGCAGATCTACAATGTCACCCAGGAGGATATAGGATTCTACAGCCTACGACTCATAAATAGACATGCAGAAATTGTGTCAATAATGTCTACATACCTCAATGTGTACT

>Mna_Psg46N1_partial (Mastomys natalensis; African soft-furred rat) WGS JADRCE010171488.1

CCTCCCTTTTAACATGCTGGCACCTGTCTACCACAGCCAAGACCACCATTGAATTATTGCCACCCCACGTGGTTGAAGGAGAAGATGTCCTTTTTCTTGTCCACAATCTGCCAGAGAATCTTACAGCCTTTGCCTGGTTTAAAGGGAGGACAAATGAGAAACATGGAATTGCACTATATGCAGTGGTCTCTGATATACATATACACAGCGATAGAGAGACATTATACAGCAATGGATCCCTGATGATCCACAATATCACCCAGAAGGACAGAGATTATTACACCCTACGAACCTTCAATAAACATGCAGAAACTGTATCAACAACATCCACATTCnnnnnnnnnnnn

>Moc_Ceacam9N (Microtus ochrogaster; prairie vole) WGS AHZW01167869.1

CCTTCCTCTTAACCTGCTGGAATGCACCCACCACTGCCGAACTCACTATTGAATTAGTGCCCCCCATGGTTGCTGAAGGTGGAAACTCCGTCCTATTTGTGCATAAAATGCCGCTGAACGTCCAGGCATTTTACTGGTACAAACAGAAAGATGCGACCAAGAGCTACGAAGTTGCACGCTACTTAACACCCAATAACACAACGTCGAAGATGCCTCAACATAGCGGTAGGAAAACGGTATTCTACAGTGGATCCCTGCTGATCAGAAACGTCACCCAGGCTGACAGCGGATTCTACACCTTACTGACGTTCAACACAGAAATGCAAAGTGAACTCACACACATACATCTGGAAGTATACA

>Moc_Psg1N1 (Microtus ochrogaster; prairie vole) WGS AHZW01168534.1

CCTTCCTTTTAACCTGTTGGCACCTGCCTACCACTGCCCAAGTCACCATCGAATTAGTGCCGCCCCAAGTGGTTGAAGGAGAAAATGTTCTTCTGCGTGTTCATAATCTACCAGAGAATCTTCTAGCCTTTGTCTGGCACAAGGGGGTGAGGAATATGAGCCTTGGAATTGCACTATATTCATTGGCCAAGGGTTTAAGTGTGACAGGGCCCATACACAGTGGTAGAGAGACAGTATACAGCAATGGATCCCTGCAGATCTACAATGTCACCCAGAAGGACACAGGATTCTACACCTTTCGAACCATAAATGGACAAGTAGGAGTTGCATCAATAACAACCACGTACCTTCACGTGTACA

>Moc_Psg2N1 (Microtus ochrogaster; prairie vole) WGS AHZW01168473.1

CCTACTTTTTAACCTGTTGGCACCTGCCCACCACTGTCCAAGTCATTGTTGATTTAGTGCCATCCCATGTTGTTGAAGGAGAAAATGTCCTTCTTCGTGTTCGCAATCTGCCAGAAGATCTTGTTGCCTTTGTCTGGCACAAAGGGGTGACAAAGATGGACCTCGGAATTGTACTTTATTCACTGACCACTAATTTAAAAATCACAGGGCCTGGACACAGTGGTAGAGAGATAGTGTACAGAAATGGATCTCTGTGCCTCCAAAATGTCACCCAGAAGGACACAGGATTCTACACGCTACGATCCTTAAATAGGCATAAAGGAATTGTATCAACAACATCTATATACCTGCATGTATACT

>Moc_Psg3N1 (Microtus ochrogaster; prairie vole) WGS JAATJU010025930.1

CCTCCCTTTTCTCCTTCTGGCATCTCCCCACTACTGCTCAGGTGTCCACTGAATCAGTGCCACCCCTAGTGGCTGAAGGTGATAATGTTCTTATCCTTGTCAACAATCTGCCAGAGAATCTTTTAGCCTTAGCCTGGTTCAAAGGGTTAACAAATATGAAGCAAGGAATCGCATTATATGCACTGCACAAAAATATAAGTGTTACAGGGCCTGTGCACAGTGGCAGAGAGACAATATATCACAATGGATCCTTGTTGATTGAAAAACTCACCCAGAAGGACACAGGATTCTACACCTTTCGAGCCTATAATAGACGTGGAAGAATTGTATCAAGCACATCCACCTACCTCAATGTGCAAG

>Moc_Psg4N1 (Microtus ochrogaster; prairie vole) WGS JAATJU010023499.1

CCTCACTTTTAGGCTGCTGTCTATCCACCACTGACTATATCACCATTAAATCTGCCCAACCCCATGTGGCCAGTGGAGAAGACGTCCTTCTTCATGTCCACAATCTGCCAGAGGATATTCTAGCCTTCGCCTGGTTCAAAGGGGCAACAAGCATGAAACATGGAATTGCAGTATATGCACTGAACAAAAATTTAAGTGCGACAGGGCCTGCACATAGTGGTAGAGAGACAGTGTACCATAATGGATCCCTGCTGCTCCAAAGTGTCACTGAGAAGGACACAGGATTCTATACCCTACGAACCTTAGATAGACACGGAGAGATTGTATCAACAACAACCATGCGCCTCTATGTGTACC

>Moc_Psg5N1 (Microtus ochrogaster; prairie vole) WGS JAATJU010025930.1

TCTCCCTTTTAAGCTCCTGGCATCTGTCCACAAATGCCCATATGACTATTGAAAAAGTGCCAGCCCTAGCTGCTGAAGGAGATGACGTCTTTTTCCATGTCAATGATCTGCCAGAGAATACTACAACCATAGCCTGGTTCAAAGGTCTAAGAAATACGACACAAGGAATTGGAGCATATGCACCGTTCTTAAATTTGAGTAGGCCAGGTCCTATGTACAGTGGTAGAGAGACAATATACCGCAATGGATCCCTGCTGATAAAAAATGTCAACCCGATGGACATTGGATTCTATACCCTACGAACTTATAATAATCATGGAACTAGGACATCAATAACATCCGAGTACCTCCAAGTGCACG

>Moc_Psg6N1 (Microtus ochrogaster; prairie vole) WGS JAATJU010025930.1

TCTCCCTTTTAACCTCCTGGCACCTGTCCACTGCTGTCCATATAACTACTGAGTCAAGTCGAGTGGTTGAAGGAGAAAACATCCTTTTCCTTGTGCATGATCTACCAGATAATACTAAATCCTTAGTCTGGTTCAAAGCTCTAAAAAATGTGACAGAAGAAATTGCAGCATATGCACTGCCCTACAATTTAAGTAGGCCAGGTCCTCTGTACAGTGGTAGAGAGACAATATATCGCAATGGATCCCTGATGATAGAAAATGTCAACCTCAAGGACACAGGATTCTATATTCTACAAACCTATAACAGACGTAAAAAAGTCATATCAACAACAACCATGTACCTCCAAGTGAATG

>Moc_Psg7N1 (Microtus ochrogaster; prairie vole) WGS AHZW01168540.1

CCTCCCTTTTAACCCTCTGGCACCTGTCCATCACGGCCTCTGTGACCATTGAATCAGTGCCACCCCTGATGGCCGAAGGAGATGACATTCTTTTTCTTGTCGACAATCTACCGGAGAAGACTGTAACCTTAGTCTGGTTCAAAGGGCTAACAAATATGAAAGCTGTGATTGCAATATATGGACGGCACATCAATTTAAGTGCATCTGGGCCTTTGCACAGTGGTAGAGAGACAATATATTACAATGGATCCCTGCTGATTAAAAAGATTACCCAGAAAGACACAGGATTCTATACCCTACGAAgctatgataagtatttaaacatcatatcaacaacattcacatacgtccatgttcacg

>Moc_Psg8N1 (Microtus ochrogaster; prairie vole) WGS WGSAHZW01167815.1

CCTTCCTTTTATCCTGTTGGCACCTGCCTACCACTGCCCAAGTCACCATCGAATTAGTGCCGCCCCAAGTGGTTGAAGGAGAAAACGTTCTTCTACGTGTTCATAATCTACCAGAGAATCTTCTAGCCTTTGTCTGGCACAAAGGGATGAGGAATATGAGCCTTGGAATTGCACTATATTCACTGGCCAAGGGTTTAAGTGTGACAGGGCCCATACACAGTGGTAGAGAGACAGTGTACAGCAACGGATCCCTGCAGATCCACAATGTCACCCAGAAGGACACAGGATTCTACACCTTTCGAACCATAAATGGACAAGTAGGAGTCTCATCAATAACAACCACGTACCTTCATGTGTACA

>Moc_Psg9N1 (Microtus ochrogaster; prairie vole) WGS AHZW01167820.1

CCTTCCTTTTAATCTGTTGTCATTCACCCGCCACTGCTGAAGTAACCATTGAATCAGTGCCGCCCAATGTGTTCGAAGGAGACAATGTTCTTCTATATGTCCACAGTCTGCCAGAGAATCTGCTAGGCTTTGCTTGGTTCAAAGGGCTAACAAATATGAAACGCAGAATTGTACTCTATGAACTGAACAGCAATTTAAGTTTGCCGGGGCCTGAATACAGCGGTAGAGAGACAGTCTACAGCAATGGATCCCTGTGGATTTCCAATGTCACCCACATGGACACAGGATTCTATACCCTACGAACCATCAGTAGACATTCAAGAGTTGTGTCACTAACAACCATCCACCTCCCTGTGTACA

>Moc_Psg10N1 (Microtus ochrogaster; prairie vole) WGS AHZW01168457.1

CCTCCATTTTAGGCTTCTGGCACATGTCCACTACTGCCCATGAGATCACTGAGTCACTGCCACGCCAAGTGGTTGAAGGAGACAACGTTCTTTTGCTTGTCCACAATCTGCGAGAGAATCTTATAGCCTTTGCCTGGTTCAAAGGGCTAAAAAATATGACTCAAGGAATTGCAGTATATACAGTGCACAACAATTTAAGTGCACCAGGGCCTGTGCACAGTAGCAGAGAGACAGTTTATAGCAATGGATCCCTGCTGATAGAAAATGTCACTCAGAAAGACACAGGAATCTATACCCTACGAACCTATAATAGAAGAGGAAAAATTGCATCAACAACATCTATGTACCTCCACGTGCACG

>Moc_Psg11N1 (Microtus ochrogaster; prairie vole) WGS AHZW01167817.1

CTTCCCTTTTAACTTACTGGTATCTACCCATCACTGCCCAAGTCACCATTGAATTAGTGCCACCCAAAGTGTTCCAAGGAGAAAATGTTCTTCTAGAGGTCCACAATCTGCCAGAGGACTTTCTAGCCTTTGCTTGGTACAGAGGGGTGACAAACATGAAACGCGGAATTGCAGTCTACGCCAAAAGAAACAGTTTAAATGCAACGGGGCCTGGGTACAGTGGTAGACAGACAATGTACAGTGACGGATCACTGCTGCTCCAGCATGTCATCCTCAAGGACACAGGATTCTACACCCTACGAGTCATAAATAGACAAGGAGAATTTATATCAACAACATCTGTGTTCCTCCATGTGCAGA

>Moc_Psg12N1 (Microtus ochrogaster; prairie vole) WGS JAATJU010025359.1

CCTCCCTTTTAATCTTTGGGCACCTGCCCACCACTGCCCGTGTGATCACTGAATTAGTACCACCCGAAGTGGCTGAAGGAGAAAACGTTCTTTTTATTGTCCACAATCTGACAGAGAATGTTAAATCCTTTGCCTGGTTCAAAGGGCTAAAAATGGAGAAACAAGGAATTGCAACGTATAGACGGCGCAAGAGTTTAGTTACAAATGGGCCTATGCACAGTGGCAGAGAGACCATATATCGCAATGGATCCCTGCTGCTCCAAAAGGTCTCCCATAATGACACAGGATTCTTTACCCTACAAACCTATGATAGGCATGAAAAAATCCTATCAACCACTTCTGTGTATCTCCATGTGCATG

>Moc_Psg13N1 (Microtus ochrogaster; prairie vole) WGS JAATJU010025359.1

CCTCCCTTTTAACCTTCTGGCTCCTCTCCACCACTGCCCATGAGATCACTGTATCAGTGCCACCCATAGTGTCTGAAGGAGATGACGTCCTGTTCCTTGTCCACAATCTGCCAGGGGAAATTGAATCCTTAGCCTGGTTCAAAGGGCTAGGAGATGAGGCAGAAGAAATTGCAACATATGCACTGCACAGAGGTTTAAGCAGGCCAGGTCCTGCGCACAGCAGTAGAGAGACAGTATATCACAATGGATCCATGATGTTTGAGAAGGTCAACCTGAAGGACACAGAATTCTATACCCTACGAACCTATAATAGAAGTGGAAAAATCATATCAACAGCAAACGTGTACCTCAATGTGTATG

>Moc_Psg14N1 (Microtus ochrogaster; prairie vole) WGS JAATJU010025359.1

CCTCCCTTTTAACCTTCTGGCACCTGTCCACCATCGCCCGTGAGACCACTGTGTCAGTGCCACCCCTAGTGCCTGAAGGAGATGACGTCCTGTTCCTTGTCCACAATCTGCCAAAGGACATTAAATCCTTAGCCTGGTTCAAAGGGCAAGGAAACACAAGCAAAAAAATTGCAACATATACACTGCACAACGATTTAAGGAGGCGAGGTCTTGCATACAGCAATAGAGAGACAATATATCACAACGGATCTATGCTGTTTGAGAAGGTCACCCTGAAGGACTCAGGATTCTATACCCTACAAACCTATAATAGACATGGAAAAAATGTATCAACAACATCTGTGATCCTCAATGTGAAAG

>Moc_Psg15N1_partial (Microtus ochrogaster; prairie vole) WGS JAATJU010025359.1

CCTTCCTTTTAACCTTCTGGCACCTGTCCACCACTGCCTTTGTGACCACTGTGTCAGTGCCATCCCGAGTGGCCGAAGGAAATGACGTCCTGTTCCTTGTCCACAATCTGCCAGAGAAATTTAAAACCGTTGCCTGGTTCAGAGGGCCCTCAAATATGACTGCAATATATGGACTGCCCGACAATTTAAGTAGGCCAGGTCTTGCACACAGCGGCAGAGAGACAATATTTCACAATGGATCCATGCTCCTTGAAAAGGTCAACCTGAAGGACACAGGCTTCTATACCGTACGAACCTACAATATACATGGAAATGCCATTTCAACAACATTCACATACCTCAACGTGTATG

>Moc_Psg16N1 (Microtus ochrogaster; prairie vole) WGS AHZW01168474.1

CCTCCCTTTTAACCTGCTGGCACCTGTCCACCACTGTCAAAATCACAATTGACTCAGTGCCACTCCAAGTGGTTGAAGGAGAAAGCGTCCTTCTACGTGTCAACAATCTGCCACAGAATCTTCGAAATTTTGCCTGGTTCAAAGGGGTGACAAATATGAACTTCAGAATTGCATTATATTCACTGACCAGTAATCTATGTGTGATGGGGCCTGAAAATAGTGGTAGAGAAGCTGTGTACAGCAATGGATCCCTGTTTCTCAAAAATGTCTCCCAGAAGGACACAGGATTTTATATACTACGAACAGTAATTGGAGGTGGAAAAATTGTATATACAACCACATACCTCCACGTGTATG

>Moc_Psg19N1 (Microtus ochrogaster; prairie vole) WGS JAATJU010000725.1

CCTCCCTTTTAACCTCCTGGCACCTGTCTTCCACTGCCAATGTGACCATTGAATTACTGCCAACTCCAGTGGCTGAAGGAGATAACGTCCTTTTCCTCGTCCAAAATCTGCCAGAGGAAATAAAAGCTGTAGCCTGGTTCAAAGGGCTGGGAAATAAGAAACAACAAATTGCAGTGTATGTACTGGACAAAAAAATTAGTAAGCCAGGTCCTATGCACAGCGGGAGAGAGACAATATATCACAATGGATCCCTGCTTCTTGAAAAGGTCACACAGAAGGATGCAGGATTCTATACCCTACGAACCTATGAAAGAGGTGGAAAATTTGTATCAACAATAACCATGTACCTCTACGTGCAAG

>Moc_Psg20N1 (Microtus ochrogaster; prairie vole) WGS JAATJU010025930.1

CCTACCTTTTAACCTGTTGGTACCTGACCACCACTGTCCAAGTCATTGTTGATATAGTGCCACCCCATGTTGTTGAAGGAGAAAATGTCCTTCTTCATGTCCACAATCTGCCAGAAGATCTTGTAGCCTTTGTCTGGCACAAAGGGGTGACAAAGATGAACCTCGGAATTGTACCTTATTCACTGACCACTAATTTAAAAATCACAAGGCCTGGACACAGTGGTAGAGATATAGTGTACAGAAATGGATCTCTGTGCCTCCAAAATGTCACCCAGAAGGACACAGGATTCTACACGCTACGATCCTTAAATAGGCATAAAGGAATTGTATCAACAACATCTATATACCTGCATGTATACT

>Moe_Ceacam9N (Microtus oeconomus; root vole) WGS VIIU01016382.1

CCTTCCTCTTAACCTGCTGGAATGCACCCACCACTGCCGAACTCACTATTGAATTAGTGCCCCCCATGGTTGCTGAAGGTGGAAACTCCGTCCTATTTGTGCATAAAATGCCGCTGAACGTCCAGGCATTTTACTGGTACAAACAGAAAGATGCGACCAAGAGCTACGAAGTTGCACGCTACTTAACACCCGATAACACAACGTCGAAGATGCCTCAACACAGTGGTAGGAAAACGGTATTCTACAGTGGATCCCTGCTGATCAGAAACGTCACCCAGGCTGACAGTGGATTCTACACCTTACTGACGTTCAACACAGAAATGCAAAGTGAACTCACACACGTACATCTGGAAGTATACA

>Moe_Psg1N1 (Microtus oeconomus; root vole) WGS VIIU01009452.1

CCTTCCTTTTAACCTGTTGGCACCTGCCTACCACTGCCCAAGTCACCATCGAATTAGTGCCGCCCCAAGTGGTTGAAGGAGAAAACGTTCTTCTACGTGTTCATAATCTACCAGAGAATCTTCTAGCCTTTGTCTGGCACAAGGGGGTGAGGAATATGAGCCTTGGAATTGCACTATATTCAGTGGCCAAGGGTTTAAGTGTGACAGGGCCCATACACAGTGGTAGAGAGACAGTGTACAGCAATGGATCCCTGCATATCCACAATGTCACCCAGAAGGACACAGGATTCTACACCTTTCGAACCATAAATGGACAAGTAGGAGTCTCATCAATAACAACCACGTACCTTCACGTGTACA

>Moe_Psg2N1 (Microtus oeconomus; root vole) WGS VIIU01017577.1

CCTACCTTTTAACTTGTTGGCACCTGCCCACCACTGTCCAAGTCATTGTTGATTTAGTGCCTCCCCATGTTGTTGAAGGAGAAGATGTCCTTCTTCGTGTCCGCAATCTCCCGGAAGATCTTGTAGCCTTTGTCTGGCACAAAGGGGCGACAAAGATGGACCTCGGAATTGTACTTTATTCACTGACCACTAATTTAAAAATCACAGGGCCTGGACATAGTGGTAGAGAGATAGTGTACAGAAATGGATCTCTGCGCCTCCAAAATGTCACCCAGAAGGACACAGGATTCTACACGCTACGATCCTTAAATAGGCATAAAGGAATTGTATCAACAACATCTATACACCTGCATGTATACT

>Moe_Psg3N1 (Microtus oeconomus; root vole) WGS VIIU01017577.1

CCTCCCTTTTCTCCTTCTGGCATCTTCCCACTACTGCTCATGTGTCCACTGAATCAGTGCCACCCCTAGTGGCTGAAGGTGATAATGTCCTTATCCTTGTCAACAATCTGCCAGAGAATCTTTTAGCCTTAGCCTGGTTCAAAGGGCTAACAAATATGAAGCAAGGAATTGCATTATATGCACTGCACAAAAATGTAAGTGTTACAGGGCCTGTGCACAGTGGCAGAGAGACAATATATCACAATGGATCCTTGTTGATTGAAAAACTCACCCAGAAGGACACAGGATTCTACACCTTTCGAGCCTTTAATAGACGTGGAAGAATTGTATCAAGCACATCCACCTACCTCCATGTGCAAG

>Moe_Psg4N1 (Microtus oeconomus; root vole) WGS VIIU01027258.1

CCTCACTTTTAGGCTGCTGCCTATCCACCACTGACTATATCACCATTAAATCTGTCCAACCCCATGTGGCCAGTGGAGAAGACGTCCTTCTTCATGTCCACAATCTGCCAGAGGATATTCTAGCCTTCGCCTGGTTCAAAGGGGCAACAAGCATGAAACATGGAATTGCGGTATATGCACTGAACAAAAATTTAAGTGCGACAGGGCCTGCACATAGTGGTAGAGAGACAGTGTACCACAATGGATCCCTGCTGCTCCGAAGTGTCACCGAGAAGGACACAGGATCCTATACCCTAAGAACCTTAGATAGACACGGAGAGATTGTATCAACAACAACCATGCGCCTCTATGTGTACC

>Moe_Psg5N1 (Microtus oeconomus; root vole) WGS VIIU01006426.1

TCTCCCTTTTAAGCTCCTGGCATCTGTCCACAAATGCCCATATGACTATTGAAAAAGTGCCAGCCCTAGCTGCTGAAGGAGATGACATCTTTTTCCATGTCAATGATCTGCCAGAGAATACTACAACCATAGCCTGGTTCAAAGGTCTAAGAAATACGACAAAAGGAATTGGAGCATATGCACCGCTCTTAAATTTGAGTAGGCCAGGTCCTATGTACAGTGGTAGAGAGACAATGTATCGCAATGGATCCCTGCTGATAAAAAATGTCAACCCAACGGACACTGGATTCTATACCCTACGAACTTATAATAATCATGGAACTAGGACATCAATAACATCCGCGTACCTCCAAGTGCACG

>Moe_Psg6N1 (Microtus oeconomus; root vole) WGS VIIU01006426.1

TCTCCCTTTTAACCTCCTGGTACCTGTCCACCGCTGTCCATATAACTACTGAGTCAAGCCGAGTGGTTGAAGGAGAAAACATCCTTTTCCTTGTGCATGATCTGCCAGATAATACTAAATCCTTAGTCTGGTTCAAAGCTCTAAGAAACGCCACAGAAGAAATTGCAGCATATGCACTGTCCTACAATTTAAGCAGGCCAGGTCCTCTGTACAGTGGTAGAGAGACAATATATCGCAATGGATCCCTGATGATAGAAAATATCAACCTCAAGGACACAGGATTCTATATTCTACAAACCTATAACAGACGTAAAAAAGTCATATCAACAACAACCGTGCACCTCCAAGTGAATG

>Moe_Psg7N1 (Microtus oeconomus; root vole) WGS VIIU01017803.1

CCTCCTTTTTAGCCCTCTGGCACCTGTCCATCACTGCCTCAGTGACCATTGAATCAGTGCCACCCCTGATGGCCGAAGGAGATAACATTCTTTTTCTTGTCGACAATCTGCCGGAGAAGACTGTAACCTTAGTCTGGTTCAAAGGGCTAACAAATATGAAAGCTGTGATTGCAATATATGGACGGCACATCAATTTAAGTGCATCTGGGCCTTTGCACAGCGGTAGAGAGACAATATATTACAACGGATCCCTGCTGACTAAAAATGTTACCCAGAAAGACACAGGATTCTATACCCTACGAAGCTATGATAAGTATTTAAACATCATATCAACAACATACACATACGTCCATGTTCACG

>Moe_Psg8N1 (Microtus oeconomus; root vole) WGS VIIU01001864.1

CCTTCCTTTTAACCTGTTGGCACCTGCCCACCACTGCCCAAGTCACCATCGAATTAGTGCCGCCCCAAGTGGTTGAAGGAGAAAACATTCTTCTACGTGTTCATGATCTACCAGAGAATCTTCTAGCCTTTGTCTGGCACAAAGGGGTGAGGAATATGAGCCTTGGAATCACACTATATTCAGTGGCCAAGGGTTTAAGTGTGACAGGGCCCATACACAGTGGTAGAGAGACAGTGTACAGCAATGGATCCCTGCAGATCCACAATGTCACCCAGAAGGACACAGGATTCTACACCTTTCGAACCATAAATGGACAAGTAGGAGTCTCATCAATAACAACCACGTACCTTCACGTGTACA

>Moe_Psg9N1_P (Microtus oeconomus; root vole) WGS VIIU01001864.1

CCTTCCTTTTAATCTGGTGTCATTCCCACCACTGCTGAAGTCACCATTGAACCAGTGCCGCCCAATGTGTTAGAAGGAAACAATGTCCTTCTATATGTCCACAGTCTTCCAGAGAATCTGCTAGCCTTTGCTTGGTTCAAAGGGCTAACAAATATGAAACGCAGAATTGTACTCTATGAACTGAACAACAATTTAAGTTTGCCGGGGCCTGAATACAGCGGTAGAGAGACAGTCTACCGCAATGGATCCCTGTGGATTTCCAATGTCACCCACGTGGACACAGGATTCTGTACCCTATGAACCATCAATAGACATTCAAGAGTTGTGTCACTAACAACCATCCACCTCCCTGTGTACA

>Moe_Psg10N1 (Microtus oeconomus; root vole) WGS VIIU01028298.1

CCTCCATTTTAGGCTTCTGGCACATGTCCACTACTGCCCATGAGACCACTGAGTCACTGCCACGCCAAGTCGTTGTAGGAGACAACGTTCTTTTGCTTGTCCACAATTTGCCAAAGAATCTTATAGCCTTTGCCTGGTTCAAAGGGCTAACAAATATGACGCAAGGAATTGCAGTATATACACTGCACAACAATTTAAGTGCACCAGGGCCTGTGCACAGTAGTAGAGAGACAGTTTATAGCAATGGATCCCTGCTGATAGAAAATGTCACCCAGAAAGACACAGGAATCTACACCCTACGAACCTATAATAGAAGAGGAAAAATTGCATCAACAACATCTATGTACCTCCACGTGCACG

>Moe_Psg12N1 (Microtus oeconomus; root vole) WGS VIIU01009452.1

CCTCCCTTTTAATCTTTGGGCACCTGCCCACCACTGCCCGTGTGATCACTGAATTAGTACCACCCGAAGTGGCTGAAGGAGAAAACGTTCTTTTTATTGTCCACGATCTGCCAGAGAATGTTAAATCCTTTGCCTGGTTCAAAGGGCTAAAAATCGAGAAACAAGGAATTGCAACGTATAGACGGAGCAAGAATTTAGTTACAAATGGGCCTATGCACAGTGGCAGAGAAACCATATACCACAATGGATCCCTGCTTCTCCAAAATGTCTCCCATAATGACACAGGATTCTTTACCCTACAAACCTATGATACACATGAAAAAATCCTATCAACAACTTCTGTATATCTCCATGTGCATG

>Moe_Psg13N1 (Microtus oeconomus; root vole) WGS VIIU01010366.1

CCTTCCTTTTAACCTTCTGGCTCCTCTCCACCACTGCCCATGAGACCACTGTATCAGTGCCACCCATAGTGTCTGAAGGAGATGACGTCCTGTTCCTTGTCCACAATCTGCCAGGGGAAATCGAATCCTTAGCCTGGTTCAAAGGGCTAGGAGATGAGGCAGAAGAAATTGCAACATACGCACTGCACAGAGGTTTAAGCAGGCCAGGTCCTGCGCACAGCAGTAGAGAGACAATATATCACAACGGATCCATGCTGTTTGAGAAGGTCACCCTGAAGGACACAGAATTCTATACCCTACGAACCTATAATAGAAGTGGAAAAATCATATCAACAGCAAACGTGTACCTCAATGTGTATG

>Moe_Psg14N1 (Microtus oeconomus; root vole) WGS VIIU01038223.1

CCTCCCTTTTAACCTTCTGGCACCTCTCCACCACCGCCCGTAAGACCACTGTGTCAGTGCCATCCCAAGTGGCTGAAGGAGATGACGTCCTGTTCCTTGTGCACAATCTGCCAAAGGACATTAAATCCTTAGCCTGGTTCAAAGGGCAAGGAAACACAACCAAAAAAATTGCGACATATACACTGCACAGCGATTCAAGTAGGCGAGGTCTTGCGTACAGCAATAGAGAGACAATATATCACAACGGATCTATGCTGTTTGAGAAGGTCACCCTGAAGGACTCGGGATTCTATACACTACAAACCTATAACAGACATGGAAAAAATGTATCAACAACATCCGTGATCCTCGATGTGAAAG

>Moe_Psg15N1 (Microtus oeconomus; root vole) WGS VIIU01047973.1

CTTTCCTTTTAACCTTCTGGTACCTGTCCACCACTGCCTTTGTGACCACTGTATCAGTGCCATCCCGAGTGGACGAAGGAAAAGACGTTCTATTCCGTGTCCACAATCTGCCAGAGAAATTTAAAACCATTGCCTGGTTCAGAGGGCCCTCAAATATGACTGCAATATATGGACTGCCAGACAATTTAAGTAGGCCAGGTCCTGCACACAGCGGCAGAGAGACAATATTTCACAATGGATCCATGCTCCTTGAAAAGGTCAACCTGAAGGACACAGGCTTTTATACTGTACGAACCTATAATATACCTGGAAATGTCATATCAACAGCATACACATACCTCAACGTGTATG

>Moe_Psg16N1 (Microtus oeconomus; root vole) WGS VIIU01023036.1

CCTCCCTTTTAACCTGCTGGCACCTGTCCACCACTGTCAAAATCACAATTGACTCAGTGCCACTCCAAGTGGTTGAAGGAGAAAGCGTCCTTCTACATGTCAACAATCTGCCACAGAATCTTCGAAATTTTGCCTGGTTCAAAGGGGTGACAAATATGAACTTCAGAATTGCATTATATTCACTGACCAGTAATCTATGTGTGATGGGGCCTGAAAATAGTGGTAGAGAAGCTGTGTACAGCAATGGATCCCTGTTTCTCAAAAATGTCTCCCAGAAGGACACAGGATTTTATATACTACGAACAGTAATTAGAGGTGGAAAAATTGTATCTACAACCAAATACCTCCACGTGTATG

>Moe_Psg19N1 (Microtus oeconomus; root vole) WGS VIIU01025084.1

CCTCCATTTTAACCTCCTGGCACCTGTCTTCCACTGCCAATGTGACCATTGAATTACTGCCAACTCCAGTGGCCGAAGGAGATAACGTCCTTTTCCTTGTCCACAATCTGCCAGATGAAATAAAAGCTGTAGCCTGGTTCAAAGGGCTGGGAAATAAGAAACAACAAATTGCAATGTATGTACTGGACAAAAATTTAAGTAAGCCAGGTCCTATGCACAGCGGGAGAGAGACAATATATCACAATGGATCCCTGCTTCTTGAAAAGGTCACCCAGAAGGATGCAGGATTCTATACCCTACGAACCTATGATAGAGGTGGAAAATTTGTATCAACAATAACCATGTACCTCTACGTGCAAG

>Mor_Ceacam9N (Microtus oregoni; creeping vole) WGS JAGKIF010000085.1

CCTTCCTCTTAACCTGCTGGAATGCACCTACCACTGCCAAACTCACTATTGAATTAGTGCCCCCCATGGTTGCTGAAGGTGGAAACTCCGTCCTATTTGTGCATAAAATGCCGCTGAACGTCCAGGCATTTTACTGGTACAAACAGAAAGATGCGACCAAGAGCTACGAAGTTGCACGCTACTTAACACCCAATAACACAACGTCGAAGATGCCTCAACACAGTGGTAGGAAAACAGTATTCTACAGTGGATCCCTGCTGATCAGAAACGTCACCCAGGCTGACAGCGGATTCTACACCTTACTGACGTTCAACACAGAAATGCAAAGTGAACTCACACACGTACATCTGGAAGTATACA

>Mor_Psg1N1 (Microtus oregoni; creeping vole) WGS JAGKIF010000085.1

CCTTCCTTTTAACCTGTTGGCACCTGTCCACCACTGCCCAAGTCACCATCGAATTAGTGCCACCCCAAGTGGTTGAAGGAGAAAATGTTCTTCTACGTGTTCATAATCTACCAGAGAATCTTCTAGCCTTTGTCTGGCACAAAGGGGTGAGGAATATGAGCCTTGGAATTGCACTATATTCACTGGCCAAGGGTTTAAGTGTGACAGGGCCCATACACAGTGGTAGAGAGACAGTGTACAGCAACGGATCCCTGCAGATCCACAATGTCACCCAGAAGGACACGGGATTCTACACCTTTCGAACCATAAATGGACAAGTAGGAGTCTCATCAATAACAACCACGTACCTTCATGTGTACA

>Mor_Psg2N1 (Microtus oregoni; creeping vole) WGS JAGKIF010000085.1

CCTACCTTTTAACCTGTTGGTACCTGCCCACCACTGTCCAAGTCATTGTTGATTTAGTGCCACCCCATGTTGTTGAAGGAGAAAATGTCCTTCTTCGTGTTCGCAATCTGCCAGAAGATCTTGATGCCTTTGTCTGGCACAAAGGGGTGACAAAGATGGACCTCGGAATTGTACTTTATTCACTGACCACTAATTTAAAAATCACAGGGCCTGGACAGAGTGGTAGAGAGATAGTGTACAGAAATGGATCTCTGTGCCTCCAAAATGTCACCCAGAAGGACACAGGATTCTACACGCTACGATCCTTAAATAGGCATAAAGGAATTGTATCAACAACATCTATATACCTGCATGTATACT

>Mor_Psg2LN1 (Microtus oregoni; creeping vole) WGS JAGKIF010000085.1

CTTCCCTTTTAACTTACTGGTATCTACCCATCACTGCCCAAGTCACCATTGATTTAGTGCCACCCCATGTTGTTGAAGGAGAAAATGTCCTTCTTCGTGTTCGCAATCTGCCAGAAGATCTTGATGCCTTTGTCTGGCACAAAGGGGTGACAAAGATGGACCTCGGAATTGTACTTTATTCACTGACCACTAATTTAAAAATCACAGGGCCTGGACAGAGTGGTAGAGAGATAGTGTACAGAAATGGATCTCTGTGCCTCCAAAATGTCACCCAGAAGGACACAGGATTCTACACGCTACGATCCTTAAATAGGCATAAAGGAATTGTATCAACAACATCTATATACCTGCATGT

>Mor_Psg3N1 (Microtus oregoni; creeping vole) WGS JAGKIF010000085.1

CCTCCCTTTTCTCCTTCTGGCATCTCCCCACTACTGCTCATGTGTCCACTGAATCAGTGCCACCCCTAGTGAGTGAAGGTGATAATGTCCTTATCCTTGTCAACAATCTGCCAGAGAATCTTTTAGCCTTAGCCTGGTTCAAAGGGCTAACAAATATGAAGCAAGGAATCGCATTATATGCACTGCACAAAAATGTAAGTGTTACAGGGCCTGTGCACAGTGGCAGAGAGACAATATATCACAATGGATCCTTGTTGATTGAAAAACTCACCCAGAAGGACACAGGATTCTACACCTTTCGAGCCTATAATAGACGTGGAAGAATTGTATCAAGCACATCCACCTACCTCCATGTGCAAG

>Mor_Psg4N1 (Microtus oregoni; creeping vole) WGS JAGKIF010000085.1

CCTCACTTTTAGGCTGCTGCCTATCCACCACTGACTATATCACCATTAAATCTGTCCAACCCCATGTGGCCAGTGGAGAAGACGTCCTTCTTCATGTCCACAATCTGCCAGAGGATATTCTAGCCTTCGCCTGGTTCAAAGGGGCGACAAGCATGAAACATGGAATTGCAGTATATGCACTGAACAAAAATTTAAGTGCGACAGGGCCTGCACATAGTGGTAGAGAGACAGTGTACCATAATGGATCCCTGCTACTCCAAAGTGTCACCGAGAAGGACACAGGATTCTATACCCTACGAACCTTAGATAGACACGGAGAGATTGTATCAACAACAACCATGCGCCTCTATGTGTACC

>Mor_Psg5N1 (Microtus oregoni; creeping vole) WGS JAGKIF010000085.1

TCTCCCTTTTAAGCTCCTGGCATCTGTCCACAAATGCCCATATGACTATTGAAAAAGTGCCAGCCCTAGCTGCTGAAGGAGATGACATCTTTTTCCATGTCAATGATCTGCCAGAGAATACTACAACCATAGCCTGGTTCAAAGGTCTAAGAAATACAACACAAGGAATTGGAGCAATTGCACCGCTCTTAAATTTGAGTAGGCCAGGTCCTATGTACAGTGGTAGAGAGACAATATATCGCAATGGATCCCTGCTGATAAAAAATGTCAACCCGACGGACACCGGATTCTATACTCTACGAACTTATAATAGTCATGGAACTAGGACATCAATAACATCCGAGTACCTCAAAGTGCACG

>Mor_Psg6N1 (Microtus oregoni; creeping vole) WGS JAGKIF010000085.1

TCTCCCTTTTAACCTCCTGGCACCTGTCCACTGCTGTCCATATAACTACTGAGTCAAGTCGAGTGGTTGAAGGAGAAAACATCCTTTTGCTTGTGCATGATCTGCCAGATAATACTAAATCCTTAGTCTGGTTCAAAGCTCTAAGAAATGCGACAGAAGAAATTGCAGCATATGCACTGCCCTACAATTTAAGTAGGCCAGGTCCTCTGTACAGTGGTAGAGAGACAATATATCGCAATGGATCCCTGATGATAGAAAATGTCAACCTCAAGGACGCAGGATTCTATATTCTACAAACCTATAACAGACGTAAAAAAGTCATATCAACAACAACCATGTACCTCCAAGTGAATG

>Mor_Psg7N1 (Microtus oregoni; creeping vole) WGS JAGKIF010000085.1

CCTCCCTTTTAACCCTCTGGCACCTGTCCATCACTGCCTCTGTGACCATTGAATCAGTGCCACCCCTGATGGCCAAAGGAGATAACATTCTTTTTCTTGTCGACAATCTACCGGAGAAGACTGTAACCTTAGTCTGGTTCAAAGGGCTAACAAATATGAAAGCTGTGATTGCAATATATGGACGGCACATCAATTTAAGCGCATCTGGGCCTTTGCACAGCGGTAGAGAGACAATATATTACAACGGATCCCTGCTGATTAAAAAGGTTACCCAGAAAGACACAGGATTCTATACCCTACGAAGCTATGATAAGTATTTAAACATCATATCAACAACATTCACATACGTCCATGTTCACG

>Mor_Psg8N1 (Microtus oregoni; creeping vole) WGS JAGKIF010000085.1

CCTTCCTTTTAACCTGTTGGCACCTGACTACCACTGCCCAAGTCACCATCGAATTAGTGCCACCCCAAGTGGTTGAAGGAGAAAACGTTCTTCTACGTGTTCATAATCTACCAGAGAATCTTCTAGCCTTTGTCTGGCACAAGGGGGTGAGGAATATGAGCCTTGGAATTGCACTATATTCACTGGCCAAGGGTTTAAGTGTGACAGGGCCCATACACAGTGGTAGAGAGACAGTGTACAGCAATGGATCCCTGCAGATCTACAATGTCACCCAGAAGGACACAGGATTCTACACCTTTCGAACCATAAATGGACAAGTAGGAGTTGCATCAATAACAACCACGTACCTTCACGTGTACA

>Mor_Psg9N1 (Microtus oregoni; creeping vole) WGS JAGKIF010000085.1

CCTTCCTTTTAATCTGTTGTCATTCACCCGCCACTGCTGAAGTCACCATTGAATCAGTGCCGCCCAGTGTGTTCGAAGGAGACAGTGTCCTTCTATATGTCCACAGTCTGCCAGAGAATCTGCTAGGCTTTGCTTGGTTCAAAGGGCTAACAAATATGAAACGCAGAATTGTACTCTATGAACTGAACAACAATTTAAGTTTGCCGGGGCCTGAATACAGCGGTAGAGAGACAGTCTACCGCAATGGATCCCTGTGGATTTCCAATGTCACCCACGTGGACACAGGATTCTATACCCTACGAACCATCAGTCGACATTCAAGAGTTGTATCACTGACAACCATCCACCTCCCTGTGTACA

>Mor_Psg10N1 (Microtus oregoni; creeping vole) WGS JAGKIF010000085.1

CCTCCATTTTAGGCTTCTGGCACATGTCCACTACTGCCCATGAGACCACTGAGTCACTGCCACGCCAAGTGGTTGAAGGAGACAACGTTCTTTTGCTTGTCCACAATCTGCCAGAGAATCTTATAGCCTTTGCCTGGTTCAAAGGGCTAACAAATATGACGCAAGGAATTGCAGTATATACAGTGCACAACAATTTAAGTGCACCAGGGCCTGTGCACAGTAGTAGAGAGACAGTTTATAGCAATGGATCCCTGCTGATAGAAAATGTCACTCAGAAAGACACAGGAATCTATACCCTACGAACCTATAATAGAAGAGGAAAAATTGCATCAACAACATCTATGTACCTCCACGTGCATG

>Mor_Psg10LN1 (Microtus oregoni; creeping vole) WGS JAGKIF010000085.1

CCTCCATTTTAGGCTTCTGGCACATGTCCACTACTGCCCATGAGACCACTGTGTCACTGCCACGCCAAGTGGTTGAAGGAGAAAATGTTCTTTTGCTTGTCCACAATCTGCCAGAGAATCTTATAGCCTTTGCCTGGTTCAAAGGGCTAACAAATATGACGCAAAGAATTGCAGTATATACACTGCACAACAATTTAAGTGCACGAGGGCCAATGCACAGTAGTAGAGAGACAGTTTATAGCAATGGATCCCTGCTGATAGAAAATGTCACCCAGAAAGACACAGGAATCTATACCCTACGAACCTACAATAGAAGAGGAAAAATTGCATCAACAACATCTATGTACCTCCACGTGCA

>Mor_Psg11N1 (Microtus oregoni; creeping vole) WGS JAGKIF010000085.1

CTTCCCTTTTAACTTACTGGTATCTACCCATCACTGCCCAAGTCACCATTGAATTAGTGCCACCCAACGTGTTCCAAGGAGAAAATGTCCGTCTAGAGGTCCACAATCTGCCAGAGGACTTTCTAGCCTTTGCTTGGTACAGAGGGGTGACACACATGAAACGCGGAATTGCAGTCTATGCTGTAAGAATGGGTTTAAATGCAACGGGGCCTGCGTACAGTGGTAGACAGATAATGTACAGTGACGGATCACTGCTGCTCCAGCGTGTCATCCTCAAGGACACAGGATTCTACACCCTACGAGTCTTAAATAGACAAAAAGAAATTGTATCAACAACATCCGTGTTCCTCCATGTGCAGA

>Mor_Psg12N1 (Microtus oregoni; creeping vole) WGS JAGKIF010000085.1

CCTCCCTTTTAATCTTTGGGCACCTGCCCACCACTGCCCGTGTGATCACTGAATTAGTACCACCCGAAGTGGCTGAAGGAGAAAACGTTCTTTTTATTGTCCACAATCTGCCAGAGAATGTTAAATCCTTTGCCTGGTTCAAAGGGCTAAAAATGGAGAAACAAGGAATTGCAACGTATAGACGGCGCAAGAGTTTAGTTACAAATGGGCCTATGCACAGTGGCAGAGAGACCATATATCGCAATGGATCCCTGCTGCTCCAAAAGGTCTCCCATAATGACACAGGATTCTTTACCCTACAAACCTATGATAGACATGAAAAAATCCTATCAACCACTTCTGTGTATCTCCATGTGCATG

>Mor_Psg13N1 (Microtus oregoni; creeping vole) WGS JAGKIF010000085.1

CCTTCCTTTTAACCTTCTGGCTCCTCTCCGCCACTGCCCATGAGACCACTGTATCAGTGCCACCCATAGTGTCTGAAGGAGATGACGTCCTGTTCCTTGTCCACAATCTGCCAGGGGAAATTGAATCCTTAGCCTGGTTCAAAGGGCTAGGAGACGAGGCAGAAGAAATTGCAACATATGCACTGCACAAAGGTTTAAGCAGGCCAGGTCCTGCACACAGCAGTAGAGAGACAATATATCACAACGGATCCATGCTGTTTGAGAAGGTCAACCTGAAGGACACAGAATTCTATACCCTACGAACCTATAATAGAAGTGGAAAAATCATATCAACAGCAAACGTGTACCTCAATGTGTATG

>Mor_Psg14N1 (Microtus oregoni; creeping vole) WGS JAGKIF010000085.1

CCTCCCTTTTAACCTTCTGGCAACTGTCCACCACCGCCCGTAGGACCACTGTGTCAGTGCCACCCCTAGTGTCTGAAGGAGATGACGTCCTGTTCCTTGTCCGCAATCTGCCAAAGGACATTAAATCCTTAGCCTGGTTCAAAGGGCAAGGAAACACAACCAAAAAAATTGCAACATATACACGGCACAACGATTTAAGTAGGCGAGGTCTTGCGTACAGCAATAGAGAGACAATATATCACAACGGATCTATGCTGTTTGAGAAGGTCACCCTGAAGGACTCAGGATCCTATACCCTACAAACCTATAATAGACATGGAAAAAATGTATCAACAACATCTGTGATCCTCAATGTTAAAG

>Mor_Psg15N1 (Microtus oregoni; creeping vole) WGS JAGKIF010000085.1

CCTTCCTTTTAACCTTCTGGCACCTGTCCACCACTGCCTTTGTGACCACTGTATCAGTGCCATCCCGAGTGGCCGAAGGAAATGACGTCCTGTTCCTTGTCCACAATCTGCCAGAGAAATTTAAAACCGTTGCCTGGTTCAGAGGGCCCTCAAATATGACTGCAATATATGGACTGCCCGACAATTTAAGTAGGCCAGGTCCTGCACACAGTGGCAGAGAGACAATATTTCACAATGGATCCATGCTCCTTGAAAAGGTCAACCTGAAGGACACAGGCTTTTATACCGTACGAACCTATAATATACATGGAAATGCCATATCAACAACATACACATACCTCAACGTGTATG

>Mor_Psg16N1 (Microtus oregoni; creeping vole) WGS JAGKIF010000085.1

CCTCCCTTTTAACCTGCTGGCACCTGTCCACCACTGTCAAAATCACAATTGACTCAGTGCCACTCCAAGTGGTTGAAGGAGAAAGCGTCCTTCTACGTGTCAACAATCTGCCACAGAATCTTCGAAATTTTGCCTGGTTCAAAGGGGTGACAAATATGAACTTCAGAATTGCATTATATTCACTGACCAGTAATCTATGTGTGATGGGGCCTGAAAATAGTGGTAGAGAAGCTGTGTACAGCAACGGATCCCTGTTTCTCAAAAATGTCTCCCAGAAGGACACAGGATTTTATATACTACAAACAGTAATTAGAGGTGGAAAAATTGTATCTACAACCACATACCTCCACGTGTATG

>Mor_Psg19N1 (Microtus oregoni; creeping vole) WGS JAGKIF010000085.1

CCTCCATTTTAACCTCCTGGCACCTGTCTTCCACTGCCTGTGTGACCATTGAATTACTGCCAACTCCAGTGGCTGAAGGAGATGACGTCCTTTTCCTTGTCCACAATCTGCCAAAGGATATTATAGATGTTACCTGGTTCAAAGGGCGGGGAAAAGAGAAACAACAAATTGCAGTGTATGTACTGCACAAAAATTTAAGTATGCCAGGTCCTATACACAGCGGGAGAGAGATAATATATCACAATGGATCCCTGCTTCTTGAAAAGGTCACCCAGAAGGATGCAGGATTCTATTCCCTACGAACCTATAGTAGAGGAAGAAAATTTATATCAACAATGCCCATATACCTCCACGTACA

>Mpa_Ceacam9N (Mus pahari; shrew mouse) WGSFMBV02008332.1

CCTTCCTCTTAACCCGCTGGAATGCGTCCGCCGCTGCCGAGCTCACTATTGAACTAGTGCCGCCCAAGGTGGCCGAAGGCGGAAACTCCATTCTGTTTGTGCATGAAATGCCACTGAATGTCCAAGCGTTTTACTGGTACAAACAGAGAGATTCCACGAAGAGCTACGAAGTCGCACGGTACTTAACACCCACGAACCAAAGTTCCAAGATGCCTCAGCACAGTGATAGGAAAACCGTATTCTACAGTGGATCCCTGCTGATCAGAAACGTCACCAAGGCTGACAGTGGAGTCTACACCTTACTAACATTTAACACGGAAATGGAAAGCGAATTAACACATGTGCATCTGGAAGTGCACG

>Mpa_Ceacam11N1 (Mus pahari; shrew mouse) WGS FMBV02017296.1

TCTCCCTTTTAACCTGCTGGCTGATTCCAACTACCGCCCAGATCACCATTGAATCAGTGCCTCCCATTGCTGTTGAAGGGGAAAATGTTCTTTTGTTTGTGCACAACTTGCCAGAGAATGTTAAAGCCCTTTCCTGGTACACAGGAGTTAAACCACTCAAGAGTTGTGAAATTGCAAGTCATGTGATAGCTACAAATTCTACTGTGGTGGGACTTGCACACAGTAGTAGAGAGGCTGTACTCAAAAATGGATCTCTGCTGATCAAGAGTGTCACCACAAAAGACTCAGGTTACTACACTCTACAAATACTTGATACAACCTCAAGACCTGAATTAATACGTGCAGAATTCTTTGTTCACA

>Mpa_Ceacam12N1 (Mus pahari; shrew mouse) WGS FMBV02016657.1

TCTCCCTTTTAACATGCTGGCTGCTTCCCACTACTGCCCAGATCAGTATTGAATCAGTGCCTCCCATCGCTGTTGAAGGGGATAATGTTCTGCTCTTAGTGCAAAACTTGCCGGAGGATGTTCAATCCCTTTCCTGGTACACAGGAGGTAAACCACTCAAGAGGTTTGAAATTGCAAGACATGTGATAGCTACCAATTCTAGTGTGCCGGGACCTGCACACAGTGGTAGACAGACAGTACTCAACAGTGGATCTCTGCTGATCAAGAGTGTCACCAGAAAAGACTTGGGATACTACACTCTACAAATACTTGATACAACCTCAAGACGTGAAATAACACGTGCAGAATTCTTTGTACAGA

>Mpa_Ceacam13N1 (Mus pahari; shrew mouse) WGS FMBV02017296.1

TCTCCCTTTTAACATGCTGGCTGCATCCCACTACTTCTCAGCTCACCATTAAATCAGTGCCTCCCATTGCTGTTGAAGGGGAAAATGTTCTTCTGTTTGTGCATAACCTGCCAAAGAATGTTAAAGCCTTTTCCTGGTACTCAGGAGTTGCACCATTCAAGTGTTGTGAAATTGCAAGTCATATGATAGCTACCAATTTTACTGTAATGGGACTTGCACACAGTGGTAGAGAGACAGTACTCAACAATGGATCTCTGTTAATCAAGAGTGTTACCAGAAAAGACTCAGGATACTACACTCTTCGAACACTTGATTCAACATCAAGACCTGAAATAATACGTGCAGAATTCTTTGTACACC

>Mpa_Ceacam14N1 (Mus pahari; shrew mouse) WGS FMBV02015760.1

TCTCCCTTTTAACCTGCTGGCTGATTCCCACTACTTCCCAACTCACCATTAAATCAGTGCCTCCCATTGCTGTTGAAGGGGAAAATGTTCTTCTGTCTGTGCATAACCTGCCGAAGAATGTTAAAGTCTTTTCCTGGTTCACAGGAGTTAGAGTGCTCAAGAGTTGTGAAATTGCAACTCATGTGATAGCTATCAATGTTACTTTGGTGGGACATTCACATAGTGGCAGAGAGATAGTATTCAAAAATGGATCTCTGCTGATCAAGAGTGTCACCAGGAAAGACTCAGGATACTACACTCTACAAACACTTGATGCAACCTCAAGACCTGAAATAATACTTGCAGAATTCTTTGTACACA

>Mpa_Ceacam15N (Mus pahari; shrew mouse) WGS FMBV02008332.

CATCACTTTTAATCTGCTGGAGCTGGCCCACTGCAGCACTGCTGACCTCTAAGGAAATGCGCTTTTCGGCTGCTGAAGGGGCAAAGGTGCTTTTCTCTGTTCCTGACCAGGAAGAGAACCTCCTCTCCTTTTCCTGGTACAAAGGGAAGGATGTAAATGAAAATTTTACAATTGCACTTTATAAAAAATCCAGCGATTCACTTCAACTTGGAAAGACTGTCAGCGGCAGGGAAGAAATCTATAAGGATGGCTCCATGATGCTCCAGGCTGTCACCCCGGAAGACACGGGATTCTACACGTTACAAACCTTTAAAGCACATGGTCAACAGGAAGTAACATATGTCCATCTCCAAGTATACA

>Mpa_Psg16N1 (Mus pahari; shrew mouse) WGS FMBV02008336.1

CCTCCCTTTTAGCCTGCTGGCTCCTGTCCACCACTGCCCAGGTCACCATTGAATCGGTGCCATTCAATGTGGTTGAAGGAGAAAATGTCCTTCTTCTCATCGACAATCTGCCAGAGAACCTTATAGCCTTAGCCTGGTACAGAGGACTGAGGAAAATTGTTGTATACACACTGAACACTAAAGTAAGTGTAATGGGGCAAATGCACAGTGGTAGAGAGATAGTGTCCAGCAACGGGTCCCTGTGGATCCACAATGTCACCCGGAAGGACACAGGATTCTACACCCTACGGACCATAAATAGACGTGGAGAAATTGTATCAACATCATCCATGTACCTCTATGTGTACA

>Mpa_Psg17N1 (Mus pahari; shrew mouse) WGS FMBV02008351.1

CCTCCCTCTTAACCTGCTGCCTCCTGCCCACCACTGCCAGAGTCACCGTGGAATCTTTACCTCCCCAAGTGGTTGAAGGAGAACATGTTCTTCTACGTGTTGACAATCTGCCAGAGAATCTTCAAGGTTTTGTCTGGTACAAAGGGGTGACAAGTATGAGGCTTGGAATTGCACAGTATTCCCTGCGCTATAATGTAAGTGTGACTGGGCCTAAGCACAGCGGTAGAGAGACATTGTACAGCAACGGGTCCCTGTGGATACAAAATGTCACCCGGGAGGACACAGGATATTACACTCTTCGAACCATAAGTCAACGTGGAGAACTGGTATCAAATACATCCATCTTCCTTCAGGTGTACT

>Mpa_Psg18N1 (Mus pahari; shrew mouse) WGS FMBV02008349.1

CCTCCCTCTTAACCTGTTGGCTCCTACCCGCCACTGCCAGAGTCACCATCAAATCCTTACCACCCCAAGTGGTTGAAGGAGAAAATGTTCTTCTATACGTTGACAATCTACCAGAGAATGTTCTAGTCTTTGGCTGGTACAGAGGAATGACAAATTTTCGGCATGCAATTGTACTGCATTCTCTGTACTATAGTGCAAGTGCGAAGGGGCTGAAGCACAGTGGCAGAGAGACATTGTACATCAACGGGTCCCTGCGGATCCAAAATGTCACTCAGGAAGACACAGGATATTACACTTTTCAAACCATAAGTAAACATGGAGAAATGATATCAAATACATCACTGTACTTTCATGTGTACT

>Mpa_Psg19N1 (Mus pahari; shrew mouse) WGS FMBV02008350.1

CCTCCCTCTTAACCTGCTGGCTCCTGCCCACCACTGCCCATGTCACCATCGAATCCTTACCACCCGATGTGGTTGAAGGAGAAAATGTTCTTCTACAAGTTGACAATCTGCCAGAGAATCTTCGAGTCTTTGTCTGGTACAGAGGGCTGACAGACAAGAGCCTTGGAATTGCATTGTATTCACTGGACTATAGCACAAGTGTGACAGGACCTGAGCACAGCGGTAGAGAGACATTGTACAGCAACGGGTCCCTGTGGATCCAAAATGTCACCCGGGAGGACACAGGATATTATACTCTTCAAACTATAAGTAAAAATGGAAAACTGGTATCAAATACATCCNTGTTCCTTCAGGTGTACT

>Mpa_Psg20N1 (Mus pahari; shrew mouse) WGS FMBV02017628.1

CCTCCCTCTTGACCTGCTGGCTCCTGTCCACAACTGCCCAAATTACCATCCAGTCACCACTCCAAGTAGTTGAAGGAGAAAATATTCTTCTACGAGTTGACAATCTGCCAGAGAATCTTCTAGCCTTTTCCTGGTACAGAGGGATGACAAATTGGAGGTTCACAATTGCTTTGCATTTACTGGATTATAACACAAGTATGACAGGGCTTGAGCACAGTGGCAGAGAGATATTGTACAGCAACGGGTCCCTGTGGATCCAAAATGTCACCCAGGAGGACACAGGATATTACACTCTTCAAACCATAAGTAGACATGTAGAACTGGTGTCAAATACATCCACATTCCTTCAGGTGTACT

>Mpa_Psg24N1 (Mus pahari; shrew mouse) WGS FMBV02008338.1

CCTCCCTCTTAACTTGCTGGCTCCTGCTCACCACTGCCCAAGTTGACATCGAATCCTTACCACCCCAAGTGGTTGAAGGAGAAAATGTTCTCCTATGTGTTGACAATCTGCCAGAGAATCTTCTAGGCTTTATCTGGTACAAAGGGGTGACAGACATGAGCCTCGGAATAGCACTGTATTCACTGGCCTATAGCGTAAGTGTGACGGGACCTGTGCACAGTGGTAGAGAGACATTGTACCGCAATGGATCCCTGTGGATTCAAAATGTCACTCAGGAGGACACTGGATTCTACACCCTACGAACCATAAGTAAACGTGGAGAAATTATATCAAATACATCCATACATCTTCACGTGTACT

>Mpa_Psg25N1 (Mus pahari; shrew mouse) WGS FMBV02017628.1

CCTCCATCTTAACCTATTGGCTCCTGCCTACCACTGCCAGAGTCATCATCCATTCCTTACCACTCCAAGTGGTTGAAGGAGAAAATGTTCTTCTACGAGTTGACAATCTGCCAGAGAATCTTCTAGCCTTTGCCTGGTACAGAGGGTTGATGAATTTGAAGCTTGGAATTGCACTGTATTCACTGTGCTATAACGTAGATGTGACAGGACCTGAGCACAGCGGTAGAGAGACATTGTACAGCAACGGGTCCCTGTGGATCCAAAATGTCACCCGGGAGGACACAGGATATTACACTTTTCGAACCATAAGTCAACGTGGAGAACTTGTATCAAATACATCCATCTTCCTTCAGGTGTACT

>Mpa_Psg27N1 (Mus pahari; shrew mouse) WGS FMBV02017628.1

CCTCTCTCTTGACCTGCTGGCTCCTGTCCACCACCGCCAGAGTCACCATCCATTCACCACTCCAAGTGGTTGAAGGAGAAAGTGTTCTTCTACAAGTTGACAATCTGCCAGAGAATCTTCTAGCCTTTTCCTGGTACAGAGGCCTGACAAATTGGAAGCTTGCAATCGCACTGCATTTACTGGACTATAACACAAGTATGACAGGGCCTGACTACAGCGATAGAGAAATATTGTACAGCGACGGGTCCCTATGGATCCAAAATGTCACCCAGGAGGACACAGGATATTATACTCTTCGAACCAAAAGTAAGCATGGAGAACTGGTATCAAATACATCCACATTCCTTCACGTGTACT

>Mpa_Psg29N1 (Mus pahari; shrew mouse) WGS FMBV02008340.1

CCTCCCTTTTAACCTGCTGTTACCTGTCTACCACTTCCAAAGTCACCATTGAATTATTGCCACCCCAAGTGGTTGAAGGAGAAGATGTTCTTTTCCTTGTCAATAATCTGCCAGGGAATCTTACAGCCTTTGCCTGGTTTAAAGGGAGGACAAATAGGAAACGTGGAATTGCACTGTATGCAGTGACCTCCGACATACATATACACAGTGACAAAGAGACATTGTACAGCAATGGATCTCTGATGATCCACAATACCACCCAGAAGGACAGAGGTTATTACACCCTACGTACCTTCAATAAACATGCAGAAACTGTATCAACAACATCCACATTCCTCCATGTGAACC

>Mpa_Psg30N1 (Mus pahari; shrew mouse) WGS FMBV02008342.1

CTTCCTTTTTAACTCGCTGGCACCTGCCTACCACTGCACAAATAACCATTGAATTAGTGCCACCTCAAGTGATTGAAGGAGAAAATGTTCTCATACGTATCGACAATCTGACAGAGAATCTTATAACCTTAGCCTGGTTCAGAGGAACGAGGATTAAGAGCCCTCAAATTGGACAATATACACTTTCCACTAATGTTACTGTGCTGGGGCCTGGTCACAGTGGTAGAGAGACTTTGTACAGTAATGGATCCCTGCAGATCTACAATGTCACCCAGGAGGACATAGGCTTCTACAGCCTACGGATCATGAATAGACATGCAGAAATTGTGTCAATAACATCCATATACCTCAACGTGTACT

>Mpa_Psg31N1a (Mus pahari; shrew mouse) WGS FMBV02008345.1

CCTCCTTTTTAACCTGCTGTCACCTGCCTACCACTGCCCAAATAACCATTGAATTAGTGCCACCCCAAGTGATTGAAGGAGAAAATGTTCTCATACATATCAACAATCTGCCAGAGAATCTTACAACCTTAGCCTGGTTCAGAGGAATGAGGATTAAGAGTCCTCAAATTGGACAATATACACTTTCCACTAATGTTACTGTGCTGGGGCCTGGTCACAGTGGTAGAGAAACTTTGTACAGCAATGGATCCTTGCAGATCTACAATGTCACCAAGGAGGATATAGGATTCTACAGCCTACGAATCATAAATCAACATGCAGAAATTGTGTCAATAACATCCATATACCTCAACGTGTACT

>Mpa_Psg32N1 (Mus pahari; shrew mouse) WGS FMBV02008342.1

CTTTTCTTTTAACCTCTTGTTTCCTGCCCACCACTGTCCAAATCACCATTGAATTAGTGCCACCACAAGTGGCTGAAGGAGAAAATGTCCTTATTATTGTTTACAGTCTGCCAGAGAATCTTACAGCCATAGCCTGGTTCAAAGGAGTGACAAATATGAACCTCGGAATTGCATTGTATGCACTGGCCTCTAACATCAGTGTGAAAGGACCTGAACACAGTGGTAGAGAGACAGTGTTCAGCAATGGATCCCTGCTGCTTCACAATGTCACCCAGAAGGACACAGGATTCTATACTATACGAACCTTAAATAGAGATGGAAAAATTGTATCAACAACATCCATATACCTCCATGTGTACA

>Mri_Ceacam9N (Microtus richardsoni; water vole) WGS JAEPQW010001180.1

CCTTCCTCTTAACCTGCTGGAACGCACCCACCACTGCCGAACTCAGTATTGAATTAGTGCCCCCCATGGTTGCTGAAGGTGGAAACTCCGTCCTATTTGTGCATAAAATGCCGCTGAACGTCCAGGCATTTTACTGGTACAAACAGAAAGATGCGACCAAGAGCTACGAAGTTGCACGCTACTTAACACCCGATAACACAACGTCGAAGATGCCTCAACACAGCGGTAGGAAAACGGTATTCTACAGTGGATCCCTGCTGATCAGAAACGTCACCCAGGCTGACAGTGGATTCTACACCTTACTGACGTTCAACACAGAAATGCAAAGTGAACTCACACACGTACATCTGGAAGTATACA

>Mri_Psg1N1 (Microtus richardsoni; water vole) WGS JAEPQW010014208.1

CCTTCCTTTTAACCTGTTGGCACCTGCCCACCACTGCCCAAGTCACCATCGAATTAGTGCTGCCCCAAGTGGTTGAAGGAGAAAACGTTCTTCTACGTGTTCATAATCTACCAGAGAATCTTCTAGCCTTTGTCTGGCACAAAGGGGTGAGGAATATGAGCCTTGGAATTGCACTATATTCACTGGCCAAGGGTTTAAGCGTGACAGGGCCCATACACAGTGGTAGAGAGACAGTGTACAGCAACGGATCCCTGCAGATCCACAATGTCACCCAGAAGGACACAGGATTCTACACCTTTCGAACCATAAATGGACAAGTAGGAGTCTCATCAATAACAACCACGTACCTTCACGTGTACA

>Mri_Psg2N1 (Microtus richardsoni; water vole) WGS JAEPQW010001530.1

CCTACTTTTTAACCTGTTGGCACCTGCCCACCACTGTCCAAGTCATTGTTGATTTAGTGCCACCCCATGTTGTTGAAGGAGAAAATGTCCTTCTTCGTGTTCGCAATCTGCCAGAAGATCTTGTTGCCTTTGTCTGGCACAAAGGGGTGACAAAGATGGACCTCGGAATTGTACTTTATTCACTGACCACTAATTTAAAAATCACAGGGCCTGGACACAGTGGTAGAGAGATAGTGTACAGAAATGGATCTCTGTGCCTCCAAAATGTCACCCAGAAGGACACAGGATTCTACACACTACGATCCTTAAATAGGCATAAAGGAATTGTATCAACAACATCTATATACCTGCATGTATACT

>Mri_Psg3N1 (Microtus richardsoni; water vole) WGS JAEPQW010001530.1

CCTCCCTTTTCTCCTTCTGGCATCTCCCCACTACTGCTCAAGTGTCCACTGAATCAGTGCCACCCCTAGTGGCTGAAGGTGATAATGTCCTTATCCTTGTCAACAATCTGCCAGAGAATCTTTTAGCCTTAGCCTGGTTCAAAGGGCTAACAAATATGAAGCAAGGAATCGCATTATATGCACTGCACAAAAATGTAAGTGTTACAGGGCCTGTGCACAGTGGCAGAGAGACAATATATCACAATGGATCCTTGTTGATTGAAAAACTCACCCAGAAGGACACAGGATTCTACACCTTTCGAGCCTATAATAGACGTGGAAGAATTGTATCAAGCACATCCACCTACCTCCATGTGCAAG

>Mri_Psg4N1 (Microtus richardsoni; water vole) WGS JAEPQW010012451.1

CCTCACTTTTAGGCTGCTGCCTATCCACCACGGACTATATCACCATTAAATCTGTCCAACCCCATGTGGCCAGTGGAGAAGACGTCCTTCTTCATGTCCACAATCTGCCAGAGGATATTCTAGCCTTCGCCTGGTTCAAAGGGGCGACAAGCATGAAACATGGAATTGCAGTATATGCACTGAACAAAAATTTAAGTGCGACAGGGCCTGCACATAGTGGTAGAGAGACAGTGTACCATAATGGATCCCTGCTGCTCCAAAGTGTCACCGAGAAGGACACAGGATTCTATACCCTACGAACCTTAGATAGACACGGAGAGATTGTATCAACAACAACCATGCGCCTCTATGTGTACC

>Mri_Psg5N1 (Microtus richardsoni; water vole) WGS JAEPQW010012692.1

TCTCCCTTTTAAGCTCCTGGCATCTGTCCACAGATGCCCATATGACTATTGAAAAAGTGCCAGCCCTAGCTGCTGAAGGAGATAACGTCTTTTTCCATGTCAATGATCTGCCAGAGAATACTACAACCATAGCCTGGTTCAAAGGTCTAAGAAATACGACACAAGGAATTGGAGCATATGCACCGGTCTTAAATTTGAGTAGGCCAGGTCCTATGTACAGTGGTAGAGAGACAATATATCGCAATGGATCCCTGCTGATAAAAAATGTCAACCCGACGGACACTGGATTCTATACCCTACGAACTTATAATAGTCATGGAACTAGGACATCAATAACATCCGAGTACCTCCAAGTGCACG

>Mri_Psg6N1 (Microtus richardsoni; water vole) WGS JAEPQW010012692.1

TCTCCCTTTTAACCTCCTGGCACCTGTCCACTGCTGTCCATATAACTACTGAGTCAAGTAGAGTGGTTGAAGGAGAAAACATCCTTTTCCTTGTGCATGATCTGCCAGATAATACTAAATCCTTAGTCTGGTTCAAAGCTCTAAAAAATGTGACAGAAGAAATTGCAGCATATGCACTGCCCTACAACTTAAGTAGGCCAGGTCCTCTGTACAGTGGTAGAGAGACAATATATCACAATGGATCCCTGATGATAGAAAATGTCAACCTCAAGGACACAGGATTCTATATTCTACAAACCTATAACAGACGTAAAAAAGTCATATCAACAACAACCATGTACCTCCAAGTGAATG

>Mri_Psg7N1 (Microtus richardsoni; water vole) WGS JAEPQW010001530.1

CCTCCCTTTTAACCCTCTGGCACCTGTCCATCACTGCCTCTGTGACCATTGAATCAGTGCCACCCCTGATGGCTGAAGGAGATAACATTCTTTTTCTTGTCGACAATCTACCAGAGAAGACTGTAACCTTAGTCTGGTTCAAAGGGCTAACAAATATGAAAGCTGTGATTGCAATATATGGACGGCACATCAATTTAAGTGCATCTGGGCCTTTGCACAGCGGTAGAGAGACAATATATTTCAACGGATCCCTGCTGATTAAAAAGGTTACCCAGAAAGACACAGGATTCTATACCCTACGAAGCTATGATAAGTATTTAAACATCATATCAACAACATTCACATACGTCCATGTTCACG

>Mri_Psg8N1 (Microtus richardsoni; water vole) WGS JAEPQW010001530.1

CCTTCCTTTTAACCTGTTGGTACCTGCCTACCACTGCCCAAGTCACCATCGAATTAGTGCCGCGCCAAGTGGTTGAAGGAGAAAACGTTCTTCTACGTGTTCATAATCTACCAGAGAATCTTCTAGCCTTTGTCTGGCACAAGGGGGTGAGAAATATGAGCCTTGGAATTGCACTACATTCATTGGACAAGGGTTTAAGTGTGACAGGGCCCATACACAGTGGTAGAGAGACAGTGTACAGCAATGGATCCTTGCAGATCTACAATGTCACCCAGAAGGACACAGGATTCTACACCTTTCGAACCATAAATGGACAAGTAGGAGTTGCATCAATAACAACCACGTACCTTCACGTGTACA

>Mri_Psg9N1 (Microtus richardsoni; water vole) WGS JAEPQW010014208.1

CCTTCCTTTTAATCTGTTGTCATTCACCCGCCACTGCTGAAGTCACCATTGAATCAGTGCCGCCCAATGTGTTTGAAGGAGACAGTGTCCTTCTATATGTCCACAGTCTGCCAGAGAATCTGCTAGCCTTTGCTTGGTTCAAAGGGCTAACAAATATGAAACGCAGAATTGTACTCTATGAACCGAACAACAATTTAAGTTTGCCGGGGCCTGAATACAGTGGTAGAGAGACAGTCTACCGCAATGGATCCCTGTGGATTTCCAATGTCACCCACGTGGACACAGGATTCTATACCCTACGAACCATCAGTAGACATTCAAGAGTTGTGTCACTAACAACCATCCACCTCCCTGTGTACA

>Mri_Psg10N1 (Microtus richardsoni; water vole) GS JAGDQN010238546.1 CCTCCATTTTAGGCTTCTGGCACATGTCCACTACTGCCCTTGAGACCACTGAGTCACTGCCACACCAAGTGGTTGAAGGAGACAACGTTCTTTTGCTTGTCCACAATCTTCCAGAGAATCTTATAGCCTTTGCCTGGTTCAAAGGGCTAACAAATATGACGCAAGGAATTGCAGTATATACACTGCACAACAATTTAAGTGCACCAGGGCCTGTGCACAGTAGTAGAGAGACAGTTTATAGCAATGGATCCCTGCTGATAGAAAGTGTCACCCAGAAAGACACAGGAATCTATACCCTACAAACCTATAATAGAAGAGGAAAAATTGCATCAACAACATCTATGTACCTCCACGTGCATG

>Mri_Psg11N1 (Microtus richardsoni; water vole) WGS JAEPQW010014208.1

CTTCCCTTTTAACTTACTGGTATCTACCCATCACTGCCCAAGTCACCATTGAATTAGTGCCACCCAACGTGTTCCAAGGAGAAAATGTCCGTCTAGAGGTCCACAATCTGCCAGAGGACTTTCTAGCCTTTGCTTGGTACAGAGGGGTGACAAACATGAAACGCGGAATTGCAGTCTATGCCAAAAGAAAGGGTTTAAATGCAACGGGGCCTGCGTACAGTGGTAGACAGACAATGTACAGTGACGGATCACTACTGCTCCAGCGTGTCATCCTCAAGGACACAGGATTCTACACCCTACGAGTCATAAGTAGACAAGGAGAAATTGTATCGACAACATCCGTGTTCCTCCATGTGCAGA

>Mri_Psg12N1 (Microtus richardsoni; water vole) WGS JAEPQW010001530.1

CCTCCTTTTTAATCTTTGGGCACTTGCCCACCACTGCCCGTGTGATCACTGAATTAGTACCACCAGAAGTGGCTGAAGGAGAAAACGTTCTTTTTATTGTCCACAATCTGCCAGAGAATGTTAAATCCTTTGCCTGGTTCAAAGGGCTAAAAATGGAGAAACAAGGAATTGCAACGTATAGACGGCGCAAGAATTTAGTTACAAACGGGCCTATGCACAGTGGCAGAGAGACCATATATCGCAATGGATCCCTGCTGCTCCAAAAGGTCTCCCATAATGACACAGGATTCTTTACCCTACAAACCTATGATAGAAATGAAAAAATCCTATCAACCACTTCTGTATATCTCCATGTGCATG

>Mri_Psg13N1 (Microtus richardsoni; water vole) WGS JAEPQW010001530.1

CCTTCCTTTTAACCTTCTGGCTCCTCTCCACCACTGCCCATGAGACCACTGTATCAGTGCCACCCATAGTGTCTGAAGGAGATGACGTCCTGTTCCTTGTTCACAATCTGCCAGGGGAAATTGAATCCTTAGCCTGGTTCAAAGGGCTAGGAGATGAGGCAGAAGAAATTGCAACATATGCACTGCACAGAGGTTTAAGCAGGCCAGGTCCTGCGCACAGCAGTAGAGAGACAATATATCACAACGGATCCATGCTGTTTGAGAAGGTCAACCTGAAGGACACAGAATTCTATACCCTACGAACCTATAATAGAAGTGGAAAAATCATATCAACAGCAAACGTGTACCTCAATGTATATG

>Mri_Psg14N1 (Microtus richardsoni; water vole) WGS JAEPQW010001530.1 CCTCCCTTTTAACCTTCTGGCATCTCTCCACCACCGCCCGTAAGACCACTGTGTCAGTGCCACCCCTAGTGGCTGAAGGAGATGACGTCCTGTTCCTTGTCCACAATCTGCCAAAGGACATTAAATCCTTAGCCTGGTTCAAAGGGCAAGGAAACACAACCAAAAAAATTGCAACATATACACTGCACAACGATTTAAGTAGGCGAGGTCTTGCGTACAGCAATAGAGAGACAATATATAACAACGGATCTATGCTGTTTGAGAAGGTCACCCTGAAGGACTCAGGATTCTATACACTACAAACCTATAACAGACATGGAAAAAATGTATCAACAACATCCGTGATCCTCAATGTGAAAG

>Mri_Psg15N1 (Microtus richardsoni; water vole) WGS JAGDQN010040125.1

CCTTCCTTTTAACCTTCTGGCACCTGTCCACCACTGCTTTTGTGACCACTGTATCAGTGCCATCCCGAGTGGCCGAAGGAAATGACGTCCTATTCCTTGTCCACAATCTGCCAGAGAAATTTAAAACCGTTGCCTGGTTCAGAGGGCCCTCAAATATGACTGCAATATATGGACTGCCTGACAATTTAAGTAGGCCAGGTCCTGCACACAGTGGCAGAGAGACAATATTTCACAATGGATCCATGCTCCTTGAAAAGGTCAACCTGAAGGACACAGGCTTCTATACTGTACGAACCTATAATATACATGGAACTGCCATATCAACAACATACACATACCTCAACGTGTATG

>Mri_Psg16N1 (Microtus richardsoni; water vole) WGS JAEPQW010001530.1

CCTCCCTTTTAACCTGCTGGCACCTGTCCACCACTGTCAAAATCACAATTGACTCAGTGCCACTCCAAGTGGTTGAAGGAGAAAGCGTCCTTCTACGTGTCAACAATCTGCCACAGAATCTTCGAAATTTTGCCTGGTTCAAAGGGGTGGCAAATATGAACTTCAGAATTGCATTATATTCACTGACCAGTAATCTATGTGTGATGGGGCCTGAAAATAGTGGTAGAGAAGCTGTGTACAGCAATGGATCCCTGTTTCTCAAAAATGTCTCCCGGAAGGACACAGGATTTTATATACTACGAACAGTAATTAGAGGTGGAAAAATTGTATCTACAACCACATACCTCCACGTGTATG

>Mri_Psg19N1 (Microtus richardsoni; water vole) WGS JAEPQW010014208.1

CCTCCATTTTAACCTCCTGGCACCTGTCTTCCACTGCCTGTGTGACCATTGAATTACTGCCAACTCCAGTGGCCGAAGGAGATAACGTCCTTTTCCTTGTCCACAATCTGCCAAAGGATATTATAGACGTTACCTGGTTCAAAGGGTGGGGAAAAGAGAAACAACAAATTGCACTGTATGTACTGCACAAAAATTTAAGTATGCCAGGTCCTATATACAGCGGGAGAGAGATAATATATCACAATGGATCCCTGCTTCTTGAAAAGGTCACCCAGAAGGATTCAGGATTCTATTCCCTACGAACCTATAGTAGAGGAAGAAAATTTATATCAACAATGCCCATGTACCTCCACGTGCAAG

>Msi_Ceacam9N (Mus spicilegus; steppe mouse) WGS QGOO01036868.1

CCTTCCTCTTAACCTGCTGGAATGCACCCGCCGCTGCCGAGCTCACTATTGAATTAGTGCCACCCATGGTTGCCGAAGGCGGAAACTCCGTTCTGTTTGTGCATGAAATGCCACTGAACGTCCAGGCGTTTTACTGGTACAAACAGAGAGATTCGACGAAGAGCTACGAAGTCGCACGGTACTTAACACCCACGAACCAAAGTTCGAAGATGCCTCAGCACAGTGATAGGAAAACCGTATTCTACAGTGGATCCCTGCTGATCAGAAACGTCACCAAGGCTGACAGTGGAGTCTATACCTTACTAACATTTAACACGGAAATGGAAAGCGAATTAACACATGTGCATCTGGAAGTGCAAG

>Msi_Ceacam11N1 (Mus spicilegus; steppe mouse) WGS QGOO01036951.1

TCTCCCTTTTAACCTGCTGGCTGATTCCAACTACCACCCAGATCACCATTGAATCAGTGCCTCCCATTGCTGTTGAAGGGGAAAATGTTCTTCTGTTTGTGCATAACTTGCCAGAGAATGTTAAAGCCCTTTCCTGGTACACAGGAGTTAAACCACTCAAGAATTGTGAAATTGCAAGTCATGTGATAGCTACCAATTCTACTGTGGTGGGACCTGCACACAGTGGTAGAGAGACTGTACTCAAAAATGGATCTCTGCTGATCAAGAGTACCACCAGAAAAGACTCAGGTTACTACACTCTACAAATACTTGATACAACCTCAAGACCTGAATTAATACGTGCAGAATTCTTTGTTCACA

>Msi_Ceacam12N1 (Mus spicilegus; steppe mouse) WGS QGOO01036951.1

TCTCCATTTTAACATGCTGGCTGCTTCCCACTACTGCCCAGATAACTATTGAATCAGTGCCTCCCATTGCTGTTGAAGGGGATAATGTTCTTCTGTTTGTGCAAAACTTGCCAGAGAATGTTCAAACCCTTTCCTGGTACACAGGAGGCAAACCGCTCAAGATGTTTGAAATTGCAAGACACGTGATAGCTACCAATTCTAGTGTGATGGGACCTGCACACAGTGGTAGAGAGATGGTGCTCAATAATGGATCTCTGATGATCAAGAATGTCACCAGAAAAGACTCGGGATACTACACTCTACAAATATTTGATACAACCTCAAGACGTGAAATAATGCGTGCAGAATTCTTTGTACAGA

>Msi_Ceacam13N1 (Mus spicilegus; steppe mouse) WGS QGOO01036951.1

TCTCTCTTTTAACATGCTGGCTGCATCCCACTACTTCTCATCTCACCATTAAAGCAGTGCCTCCCATTGCTGTTGAAGGGGAAAATGTTCTTCTGTTTGTGCATAACCTGCCAAAGAATGTTAAAGCCTTTTCCTGGTACTCGGGTGCACCATTCAAGTGTTGTGAAATTGCAAGTCATGTGATAGCTACCAATTTTACTGTAGTGGGACTTGCACACAGTGGTAGAGAGACAGTACTCAACAATGGATCTCTGTTGATCAAGAGTGTTACCAGAAAAGACTCAGGATACTACACTCTACGAACACTTGATTCAACCTCAAGACCTGAAATAATACGTGCAGAATTCTTTGTACACC

>Msi_Ceacam14N1 (Mus spicilegus; steppe mouse) WGS QGOO01036951.1

TCTCCCTTTTAACCTGCTGGCTGATTCCCACTACTTCCCAGCTCACCATCAAATCAGTGCCTCCCATTGCTGTTGAAGGGGAAAACGTTCTTCTGTTTGTGCATAACCTGCCGAAGAATGTTAAAGTCTTTTCCTGGTTCACAGGAGCTAGAGTGCTCAAGAGTTGTGAAATTGCAACTCATGTGATAGCTATCAATGCTACTGTGATCGGACTTTCACATAGTGGTAGAGAGACAGTGTTCAAAAATGGATCTCTGCTGATCAAGAGTGTCACCAGTAAAGACTCAGGATACTACACTCTACGGATACTTGATGCAAACTCAAGACCTAAAATAATACGTACAGAATTCTTTGTACACA

>Msi_Ceacam15N (Mus spicilegus; steppe mouse) WGS QGOO01036868.1

CCTCACTTTTAATCTGCTGGAACTGGTCCACTGCAGCACTGCTGACCTCTAAAGAAATGCGCTTCTCGGCTGCTGAAGGGGCAAAGGTTCTTCTCTCTGTTCCTGACCAGGAGGAGAACCTCCTCTCCTTTTCCTGGTACAAAGGGAAGGATGTAAATGAAAATTTTACAATTGCACATTATAAAAAGTCCAGCGATTCACTTCAACTTGGAAAGAAAGTCAGCGGCAGGGAAGAAATCTATAAGGATGGCTCCATGATGCTCCGGGCCATCACCCTGGAAGACACGGGATTCTACACGTTACAAACCTTTAAAGCACACGGCCAACAGGAAGTAACACATGTCCATCTCCAAGTATACA

>Msi_Psg16N1 (Mus spicilegus; steppe mouse) WGS QGOO01036951.1

CCTCCCTTTTAGCCTGCTGGCTCCTGTCCACCACTGCCCAGGTCACCATTGAATCAGTGCCATTCAATGTGGTTGAAGGAGAAAATGTCCTTCTTCTTGTTGACAATCTGCCAGAGAATCTTATAACCTTAGCCTGGTACAGAGGGCTGAGGAAAATTGTTGTATACACACTGAACACTAAAGTAAGTGTGATGGGGCAAATGTACAGTGGTAGAGAGATAGTGTCCAGCAACGGGTCCCTGTGGATCCACAATGTCACCCGGAAGGACACAGGACTCTACACCCTACGGACCGTAAATAGACGTGGAGAAATTGTATCAACATCATTCACGTACCTCTACGTGTACA

>Msi_Psg17N1 (Mus spicilegus; steppe mouse) WGS QGOO01036322.1

CCTCCCTCTTATCCTGCTGCTTCCTGCCCACCACTGCCAGAGTCACTGTGGAATTCTTACCTCCCCAAGTGGTTGAAGGAGAAAATGTTCTTCTACGCGTTGATAATCTGCCAGAGAATCTTCTAGGTTTTGTGTGGTACAAAGGGGTGACAAGTATGAAGCTTGGAATTGCACTGTATTCACTGCAATATAATGTAAGTGTGACAGGGCTTAAGCACAGCGGTAGAGAGACACTGCACAGAAATGGGTCCCTGTGGATCCAAAATGTCACCTCGGAGGACACAGGATATTACACCCTTCGAACCGTAAGTCAACGTGGAGAACTGGTATCAGATACATCCATATTCCTTCAGGTGTACT

>Msi_Psg18N1 (Mus spicilegus; steppe mouse) WGS QGOO01013723.1

CCTCCCTCTTAACCTGCTGGCTCCTGCCCACCACTGCCAGAGTCACCATTGAATCCTTACCACCCCAAGTGTATGAAGGAGAAAATGTTCTTCTACGTGTTGACAATATGCCAGAGAATCTTCTAGTGTTTGGCTGGTACAGAGGAATGACAAATTTGAGGCAAGCAATTGCACAGCATTGGCTGTACTACTATAGTGTAATGGTGAAGGGGCTGAATCACAGCGGCAGAGAGACATTATACATCAACGGGTCCCTGTGGATCCAAAATGTCACACAGGAGGACACAGGATATTACACTTTTCAAACCATAAGTAAACGAGGAGAAATAGTATCAAATACATCCCTGTACTTGCACGTGTACT

>Msi_Psg19N1 (Mus spicilegus; steppe mouse) WGS QGOO01036322.1

CCTCCCTCTTAACCTGCTGGCTCCTGCCCATCACTGCCCGAGTCACCATCGAATCCGTACCACCCAAATTGGTCGAAGGAGAAAATGTTCTTCTACGAGTGGACAATCTGCCAGAGAATCTTCGAGTCTTTGCCTGGTACAGAGGGGTAATAAAATTTAAGCTTGGAATTGCACTGTATTCGCTGGACTATAACACAAGTGTGACAGGACCTGAGCACAGTGGTAGAGAGACATTGCACAGCAACGGGTCCCTGTGGATCCAAAGTGCCACCCGGGAAGACACAGGATATTACACGTTTCAAACCATAAGTAAAAATGGAAAAGTGGTATCAAATACATCCATGTTCCTTCAGGTGTACT

>Msi_Psg20N1 (Mus spicilegus; steppe mouse) WGS QGOO01036322.1

CCTCCCTCTTTACCTGCTGGCTTCTGTCCACCACTGCCAAGGTCACTATCCATTCACCGCTCCAAGTGGTTGAAGGACAAAACGTTTTTCTACGAGTTGACAATCTGCCAGAGGATCTTCTAGCTTTTGCCTGGTACAGAGGACTGAGAAATTGGAGGGTCGCAATTGCACTGCATTTAGTGGAGTATAATGCAAGTATGACAGGGCCTGAGCACAGTGATAGAGAGATATTGCACAGCAACGGGTCCCTGTGGATCCAAAATGTCACTCAGGAGGACACAGGATATTATACTCTTCAAACCATAAGTAAACATGGAAAACTGGTATCAAATACATCCACATTTCTTCAGGTGTACT

>Msi_Psg21N1 (Mus spicilegus; steppe mouse) WGS QGOO01036322.1

CCTCCCTCTTGACCTGCCGGCTTCTGTCCACCACTGCTAGTGTCACCATCCAGTCACCACAACACGTAGTTGAAGGAGAGAATATTCTTCTACAAGTTGACAATCTGCCAGAGAATCTTCTAGCTTTTGCCTGGTACAGAGGACTGATAAATTGGAGGCTCACAATTGCTCTGCATTTCCTGGACTATAGCACAAGTATGACAGGGCCTGAGCACAGTGATAGAGAGATATTGTACAGCAACGGGTCCCTATGGATCCAAAATGTCACCCAGGAGGACACAGGAAATTACATTTTTCAAACCATAAGTAACCATGGAGAACTGGAATCAAATACGTCCACATTTCTTCAGGTCTACT

>Msi_Psg22N1 (Mus spicilegus; steppe mouse) WGS QGOO01036322.1

CCTCCCTCTTAACCTGCTGGCTCTTGCCCATCACTGCCGGAGTCACCATCGAATCCGTACCACCCAAATTGGTTGAAGGAGAAAATGTTCTTCTACAAGTGGACAATCTGCCAGAGAATCTTCGAGTCTTTGTCTGGTATAGAGGGGTGACAGACATGAGCCTCGGAATTGCATTGTATTCACTTGACTATAGCACAAGTGTGACAGGACCTAAGCACAGCGGTAGAGAGACATTGTACAGAAACGGGTCCCTGTGGATCCAAAATGTCACCCGGGAAGACACAGGATATTACACTCTTCAAACCATAAGTAAAAATGGAAAAGTGGTATCAAATACATCCATATTCCTTCAGGTGAACT

>Msi_Psg_23N1 (Mus spicilegus; steppe mouse) WGS QGOO01036322.1

CCTCCCTCTTGACCTGCCGGCTTCTGTCCACCACTGCTAGTGTCACCATCCAGTCACCACAACACGTAGTTGAAGGAGAGAATATTCTTCTACAAGTTGACAATCTGCCAGAGAATCTTCTAGCTTTTGCCTGGTACAGAGGACTGATAAATTGGAGGCTCACAATTGCTCTGCATTTCCTGGACTATAGCACAAGTATGACAGGGCCTGAGCACAGTGATAGAGAGATATTGTACAGCAACGGGTCCCTATGGATCCAAAATGTCACCCAGGAGGACACAGGAAATTACATTTTTCAAACCATAAGTAACCATGGAGAACTGGAATCAAATACGTCCACATTTCTTCAGGTCTACT

>Msi_Psg24N1 (Mus spicilegus; steppe mouse) WGS QGOO01036951.1

CCTCCCTCTTAACCTGCTGGCTCCTGCCCATTACTACCCAAGTCGACATCGAATCCTTACCGCCCCAAGTGGTTGAAGGAGAAAATGTTCTTCTACGGGTTGACAATCTGCCAGAGAATCTTCTAGGCTTTGTCTGGTACAAAGGGGTGACAGACATGAGCCTCGGAATTGCACTGTATTCACTGGCCTATAGCAGAGGTGTGACGGGACCTGTGCACAGTGGTAGAGAGACATTGTACCAAAATGGGTCCCTGTGGATTCAAAATGTCACCCAGGAGGACACAGGATTCTACACCCTACGAACCATAAGTAAACGTGGAGAAATTATATCAAATACATCCATGCACCTTCATGTGTACT

>Msi_Psg25N1 (Mus spicilegus; steppe mouse) WGS QGOO01036322.1

CCTCCATCTTAACCTACTGGCTCCTGCCCACCACTGCCAGAGTCATCATCCATTCTTTACCACTCCAAGTGGTTGAAGGAGAAAATGTTCTTCTACATGTTTACAATCTGCCAGAGAATCTTCTAGGCCTTGCCTGGTACAGAGGGTTGCTAAATTTGAAACTTGGAATTGCACTGTATTCACTACAATATAATGTAAGTGTGACTGGACCTGAGCACAGCGGTAGAGAGACATTGCACAGAAATGGGTCTCTGTGGATCCAAAATGTCACCCAGGAGGACACAGGATATTACACTCTTCGTACCATAAGTAAAAATGGAAAACTGGAATCAAATACATCCATGTTCCTTCAGGTGTACT

>Msi_Psg26N1_partial (Mus spicilegus; steppe mouse) WGS QGOO01011682.1

CCTTCCTCTTAACCTGCTGGCTCCTGCCCACCACTGCCAGAGTCACCATTGAATCCTTACCACCACAAGTGGTTGAAGGAGAAAATGTTCTTCTACGTGTTGACAATATGCCAGAGAATCTTCTAGTGTTTGGCTGGTATAGAGGAATGACAAATTTGAGGCAAGCAATTGCACTGCATTCGCTGTACTATAGTGTAACGGTGAAGGGGCTGAAGCACAGCGGCAGAGAGACATTATACATCAACGGGACCCTGTGGATCCAAAATGTCACACAGGAGGACACAGGATATTACACTTTTCAAACCATAAGTAAACAAGGAGAAATGGTATCAAATACATCCCTnnnnnnnnnnnnnnnnn

>Msi_Psg27N1 (Mus spicilegus; steppe mouse) WGS QGOO01036322.1

CCTCCCTCTTTACCTGCTGGCTCCTCTCCACTACTGCCCAAGTCACCATCCATTCACCGCTCCAAGTGGTTGAAGGAGAAAATGTTCTTCTACGAGTTGACAATCTGCCCGAGAATCTTCTAGCCTTTTCCTGGTACAGAGGACTGACAAATTGGCAGCTTGCAATTGCACTACATTTACTGGACTATAACACAAGTATGACAGGGCCTGATCACAGTGATAGAGAGATATTGTACAGCAATGGATCCCTATGGATCCAAAATGTCACCAAGGAGGACACAGGATATTATACTCTTCGAACCATAAGCAAACATGGAGAACTGGTATCAAATACATCCACATTTCTTCAGGTGTACT

>Msi_Psg29N1 (Mus spicilegus; steppe mouse) WGS QGOO01036951.1

CCTCCTTCTTAACCTGCTGGTATCTGTCTACCACTTCCAAAGTCACCATTGAATTATTGCCACCCCAAGTGGTTGAAGGAGAAGATGTTCTTTTCCTTGTCAATAATCTGCCAGGGAATCTTACAGCCTTTGCCTGGTTTAAAGGGAGGACAAATAGGAAACATGGAATTGCACTGTATGCAGTGGCGTCTGACATATATGTACACAGCGATAGAGAGACATTGTACAACAACGGATCCCTGATGATCCACAATGTTACCCAGAAGGACAGAGGTTATTACACCCTACGAACCTTCAATAAACATGCAGAAACCGTATCAACAACATTCACATTCCTCCATGTGAACC

>Msi_Psg30N1 (Mus spicilegus; steppe mouse) WGS QGOO01036951.1

CCTCCTTTTTAACCTGCTGTCACCTGCCTACCACTGCACAAATAACCATTGAATTAGTGCCACCCCAAGTGATTGAAGGAGAAAATGTTCTCATACGTGTCAACAATTTGACAGAGAATCTTATAACCTTAGCCTGGTTCAGAGGAACGAGGATTAAGAGCCCTCAAATTGGACAATATACACCGGCCACTAAAGTTACTGTGCTGGGTCCTGGTCACAGTGGTAGAGAAACTTTGTACAGCAATGGATCCCTGCAGATCTACAATGTCACCCAGGAGGACATAGGATTCTACAGCCTACGAATCATAAATAAACATGCAGAAATTGTGTCAATAACATCCATATACCTCAACGTGTACT

>Msi_Psg31N1 (Mus spicilegus; steppe mouse) WGS QGOO01036951.1

CCTCATTTTTAACCTGTTGTCACCTGCCTGCCACTGCCCAAATAACCACTGAATTAGTGCCACCCCATGTGATTGAAGGAGAAAATGTTCTCATACGTGTCAACAATCTGCCAGAGAATCTTACAACCTTAGCCTGGTTCAGAGGAATGAGGATTAAGAGCCCTCAAATTGGACAATATACACTGGCCACTAATGTTACTGTGCTGGGGCCTGGTCACAGTGGTAGAGAAACTTTGTACAGCAATGGATCCCTGCAGATCTACAATGTCACCCAGGAGGACATAGGATTCTACAGCCTACGAGTCATGAATAGACATGCAGAAATTGTGTCAATAACATCCATATACCTCAACGTGTACT

>Msi_Psg32N1 (Mus spicilegus; steppe mouse) WGS QGOO01036951.1

CTTTTCTTTTAACCTCTTGGTTCCTGCCCACCACTGTCCAAGTCACCATTGAATTAGTGCCACCACAAGTGGCTGAAGGAGAAAATGTCCTTATTATTGTTTACAATCTGCCAGAGGATCTTACAGCCATAGCCTGGTTCAAAGGAGTAACAAATATGAACCTCGGAATTGCATTGTATGCACTGGCCTCTAACATCAGCGTGAAAGGGCCCGAACACAGTGGTAGAGAGACAGTGTTCAGCAATGGATCCCTGTTGCTTCACAATGTCACCCAGAAGGACACAGGATTCTATACTATACGGACCTTAAATAGACATGGAAAAATTGTATCCACAACATCCATATACCTCCACGTGTACA

>Msp_Ceacam9N (Mus spretus; Western wild mouse) WGS LVXV01009957.1

CCTTCCTCTTAACCTGCTGGAATGCACCCGCCGCTGCCGAGCTCACTATTGAATTAGTGCCACCCATGGTTGCCGAAGGCGGAAACTCCGTTCTGTTTGTGCATGAAATGCCACTGAACGTCCAGGCGTTTTACTGGTACAAACAGAGAGATTCGACGAAGAGCTACGAAGTCGCACGGTACTTAACACCCACGAACCAAAGTTCGAAGATGCCTCAGCACAGTGATAGGAAAACCGTATTCTACAGTGGATCCCTGCTGATCAGAAACGTCACCAAGGCTGACAGTGGAGTCTATACCTTACTAACATTTAACACGGAAATGGAAAGCGAATTAACACATGTGCATCTGGAAGTGCACG

>Msp_Ceacam11N1 (Mus spretus; Algerian mouse) WGS LVXV01010008.1

TCTCCCTTTTAACCAGCTGGCTGATTCCAACTACCACCCAGATCACCATTGAATCAGTGCCTCCCATTGCTGTTGAAGGGGAAAATGTTCTTCTGTTTGTGCATAACTTGCCAGAAAATGTTAAAGCCCTTTCCTGGTACACAGGAGTTAAACCACTCAAGAATTGTGAAATTGCAAGTCATGTGATAGCTACCAATTCTACTGTGGTGGGACCTGCACACAGTGGTAGAGAGACTGTACTCAAAAATGGATCTCTGCTGATCAAGAGTACCACCAGAAAAGACTCAGGTTACTACACTCTACAAATACTTGATACAACCTCAAGACCTGAATTAATACGTGCAGAATTCTTTGTTCACA

>Msp_Ceacam12N1 (Mus spretus; Algerian mouse) WGS LVXV01010008.1

TCTCCATTTTAACATGCTGGCTGCTTCCCACTACTGCCCAGATCACTATTGAATCAGTGCCTCCCATTGCTGTTGAAGGGGATAATGTTCTTCTGTTTGTACAAAACTTGCCAGAGAATGTTCAAACCCTTTCCTGGTACACAGGAGGTAAACCGCTCAAGATGTTTGAAATTGCAAGACATGTGATAGCTACCAATTCTAGTGTGATGGGGCCTGCACACAGTGGTAGAGAGACAGTGCTCAATAATGGATCTCTGATGATAAACAATGTCACCAGAAAAGACTCAGGATACTACACTCTACAAATACTTGATACAACCTCAAGACGTGAAATAACACGTGCAGAATTCTTTGTACAGA

>Msp_Ceacam13N1 (Mus spretus; Algerian mouse) WGS LVXV01010008.1

TCTCTCTTTTAACATGCTGGCTGCATCCCACTACTTCTCATCTCACCATTAAAGTAGTGCCTCCCATTGCTGTTGAAGGGGAAAATGTTCTTCTGTTTGTGCATAACCTGCCAAAGAATGTTAAAGCCTTTTCCTGGTACTCGGGAGTTGCACCATTCAAGTGTTGTGAAATTGCAAGTCATGTGATAGCTACCAATTTTACTGTAGTGGGACTTGCACACAGTGGTAGAGAGACAGTACTCAACAATGGATCTCTGTTGATCAAGAGTGTTACCAGAAAAGACTCAGGATACTACACTCTACGAACACTTGATTCAACCTCAAGACCTGAAATAATACGTGCAGAATTCTTTGTACACC

>Msp_Ceacam14N1 (Mus spretus; Algerian mouse) WGS LVXV01010008.1

TCTCGCTTTTAACCTGCTGGCTGATTCCCACTACTTCCCAGCTCACCATCAAATCAGTGCCTCCCATTGCTGTTGAAGGGGAAAACGTTCTTCTGTTTGTGCATAACCTGCCGAAGAATGTTAAAGTCTTTTCCTGGTTCACAGGAGCTAGAGTGCTCAAGAGTTGTGAAATTGCAACTCATGTGATAGCTATCAATGCTACTGTGATCGGACTTTCACATAGTGGTAGAGAGACAGTGTTCAAAAATGGATCTCTGCTGATCAAGAGTGTCACCAGTAAAGACTCAGGATACTACACTCTACGGATACTTGGTGCAAACTCAAGACCTGAAATAATACGTACAGAATTCTTTGTACACA

>Msp_Ceacam15N (Mus spretus; Algerian mouse) WGS LVXV01009957.1

CCTCACTTTTAATCTGCTGGAACTGGTCCACTGCAGCACTGCTGACCTCTAAAGAAATGCGCTTCTCAGCTGCTGAAGGGGCAAAGGTTCTTCTCTCTGTTCCTGACCAGGAGGAGAACCTCCTCTCCTTTTCCTGGTACAAAGGGAAGGATGTAAATGAAAATTTTACAATTGCACATTATAAAAAGTCCAGCGATTCACTTCAACTTGGAAAGAAAGTCAGCGGCAGGGAAGAAATCTATAAGGATGGCTCCATGATGCTCTGGGCCATCACCCCGGAAGACACAGGATTCTACACATTACAAACCTTTAAAGCACACGGCCAACAGGAAGTAACACATGTCCATCTCCAAGTATACA

>Msp_Psg16N1 (Mus spretus; Algerian mouse) WGS LVXV01009959.1

CCTCCCTTTTAGCCTGCTGGCTCCTGTCCACCACTGCCCAGGTCACCATTGAATCAGTGCCATTCAATGTGGTTGAAGGAGAAAATGTCCTTCTTCTTGTTGACAATCTGCCAGAGAATCTTATAACCTTAGCCTGGTACAGAGGGCTGAGGAAAATTGTTGTATACACACTGAACACTAAAGTAAGTGTGATGGGGCAAATGTACAGTGGTAGAGAGATAGTGTCCAGCAACGGGTCCTTGTGGATCCACAATGTCACCCGGAAGGACACAGGACTCTACATCCTACGGACCGTAAATAGACGTGGAGAAATTGTATCAACATCATTCATGTACCTCTACGTGTACA

>Msp_Psg17N1 (Mus spretus; Algerian mouse) WGS WGS CAKLHJ010000017.1

CCTCCCTCTTATCCTGCTGCCTCCTGCCCACCACTGCCAGAGTCACTGTGGAATTCTTACCTCCCCAAGTGGTTGAAGGAGAAAATGTTCTTCTACGAGTTGACAATCTGCCAGAGAATCTTCTAGGTTTTGTGTGGTACAAAGGGGTGACAAGTATGAAGCTTGGAATTGCACTGTATTCACTGCAATATAATGTAAGTGTGACTGGGCCTAAGCACAGTGGTAGAGAGACATTGCACAGAAATGGGTCCCTGTGGATCCAAAATGTCACCTGGGAGGACACAGGATATTACACCCTTCGAACCGTAAGTCAACGTGGAGAACTGGTATCAAATACATCCGTATTCCTTCAGGTGTACT

>Msp_Psg18N1 (Mus spretus; Algerian mouse) WGS CAKLHJ010000017.1

CCTCCCTCTTAACCTGCTGGCTCCTGCCCACCACTGCCAGAGTCACCATTGAATCCTTACCACCCCAAGTGTATGAAGGAGAAAATGTACTTCTACGTGTTGACAATATGCCAGAGAATCTTCTATTGTTTGGCTGGTACAGAGGAATGACAAATTTGAGGCAAGCAATTGCACAGCATTGGCTGTACTACTATAGTGTAATGGCGAAGGGGCTGAATCACAGCGGCAGAGAGACATTATACATCAATGGGTCCCTGTGGATCCAAAATGTCACACAGGAGGACACAGGATATTACACTTTTCAAACCATAAGTAAACGAGGAGAAATAGTATCAAATACATCCCTGTACTTGCACGTGTACT

>Msp_Psg19N1 (Mus spretus; Algerian mouse) WGS CAKLHJ010000017.1

CCTCCCTCTTAACCTGTTGGCTCCTGCCCACCACTGCCCGAGTCACCATCAAATCCGTACCACCCAAATTGGTCGAAGGAGAAAATGTTCTTCTACGAGTGGACAATCTGCCAGAGAATCTTCGAGTCTTTGCCTGGTACAGAGGGGTAATAAAATTTAAGCTTGGAATTGCACTGTATTCACTGGACTATAACACAAGTGTGACAGGACCTGAGCACAGTGGTAGAGAGACATTGCACAGCAACGGGTCCCTGTGGATCCAAAATGCCACCCGGGAAGACACAGGATATTACACGTTTCAAACCATAAGTAAAAATGGAAAACTGGTATCAAATACATCCATGTTCCTTCAGGTGTACT

>Msp_Psg20N1 (Mus spretus; Algerian mouse) WGS LVXV01010010.1

CCTCCCTCTTTACCTGCTGGCTTCTGTCCACCACTGCCAAGGTCACTATCCATTCACCGCTCCAAGTGGTTGAAGGACAAAACGTTTTTCTACGAGTTGAGAATCTGCCAGAGGATCTTCTAGTTTTTGCCTGGTACAGAGGACTGACAAATTGGAGGGTTGCAATTGCACTGCATTTAGTGGAGTATAATGCAAGTATGACAGGGCCTGAGCACAGTGATAGAGAGATATTGCACAGCAACGGGTCCCTGTGGATCCAAAATGTCACCCAGGAGGACACAGGATATTACACTCTTCAAACCATAAGTAAACATGGAAAACTGGTATCAAATACATCCACATTTCTTCAGGTGTACT

>Msp_Psg21N1 (Mus spretus; Algerian mouse) WGS CAKLHJ010000017.1

CCTCCCTCTTGACCTGCTGGCTTCTGTCCACCACTGCTAGTGTCACTATCCAGTCACCACAACACGTAGTTGAAGGAGAGAATATTCTTCTACAAGTTGACAATCTGCCAGAGAATCTTCTAGCTTTTGCCTGGTACAGAGGACTGATAAATTGGAGGCTCACAATTGCTCTGCATTTCCTGGACTATAGCACAAGTATGACAGGGCCTGAGCACAGTGATAGAGAGATATTGTACAGCAACGGGTCCCTATGGATCCAAAATGTCACCCAGGAGGACACAGGATATTACATTTTTCAAACCATAAGTAACCATGGAGAACTGGAATCAAATACATCCACATTTCTTCAGGTCTACT

>Msp_Psg22N1 (Mus spretus; Algerian mouse) WGS CAKLHJ010000017.1

CCTCCCTCTTAACCTACTGGCTCTTGCCCATCACTGCCGGAGTCACCATCGAATCCATACCACCCAAATTGGTTGAAGGAGAAAATGTTCTTCTACGAGTGGACAATCTGCCAGAAAATCTTCGAGTCTTTGTCTGGTATAGAGGGGTGACAGACATGAGCCTTGGAATTGCACTGTATTCACTTGACTATAGCACAAGTGTGACAGGACCTGAGCACAGTGGTAGAGAGACATTGTACAGCAACGGGTCCCTGTGGATCCAAAATGTCACCAGGGAAGACACAGGATATTACACTCTTCAAACCATAAGTAAAAATGGAAAAGTGGTATCAAATACATCCGTATTCCTTCAGGTGAACT

>Msp_Psg24N1 (Mus spretus; Algerian mouse) WGS CAKLHJ010000017.1

TCTCCCTCTTAACCTGCTGGCTCCTGCCCACCACTACCCAAGTTGACATCGAATCCTTACCGCCCCAAGTGGTTGAAGGAGAAAATGTTCTTCTACGGGTTGACAATCTGCCAGAGAATCTTCTAGGCTTTGTCTGGTACAAAGGGGTGACAGACATGAGCCTCGGAATTGCACTGTATTCACTGGCCTATAGCAGAGGTGTGACGGGACCTGTGCACAGTGGTAGAGAGACATTGTACCAAAATGGGTCCCTGTGGATTCAAAATGTCACCCAAGAGGACACAGGATTCTACACTCTACGAACCATAAGTAAACATGGAGAAATTATATCAAATACATCCATGCACCTTCATGTGTACT

>Msp_Psg25N1 (Mus spretus; Algerian mouse) WGS CAKLHJ010000017.1

CCTCCATCTTAACCTACTGGCTCCTGCCCACCACTGCCAGAGTCATCATCCATTCTTTACCACTCCAAGTGGTTGAAGGAGAAAATGTTCTTCTACATGTTTACAATCTGCCAGAGAATCTTCTAGGCCTTGCCTGGTACAGAGGGTTGCTAAATTTGAAACTTGGAATTGCACTGTATTCACTGCAATATAATGTAAGTGTGACTGGACCTGAGCACAGTGGTAGAGAGACATTGCACAGAAATGGGTCCCTGTGGATCCAAAATGTCACCCAGGAGGACACAGGATATTACACTCTTCGTACCATAAGCAAACATGGAAAACTGGAATCAAATACATCCATGTTCCTTCAGGTGTACT

>Msp_Psg27N1 (Mus spretus; Algerian mouse) WGS CAKLHJ010000017.1

CCTCCCTTTTTACCTGCTGGCTCCTCTCCACTACTGCCCGAGTCACCATCCATTCACCGCTCCAAGTGGTTGAAGGAGAAAATGTTCTTCTACGAGTTGACAATCTGCCCGAGAATCTTCTAGCCTTTTCCTGGTACAGAGGACTGACAAATTGGCAGCTTGCAATTGCACTACATTTACTGGACTATAACACAAGTATGACAGGGCCTGATCACAGTGATAGAGAGATATTGTACAGCAATGGATCCCTATGGATCCAAAATGTCACCAAGGAGGACACAGGATATTATACTCTTAGAACCATAAGCAAACATGGAGAACTGGTATCAAATACATCCACATTTCTTCAGGTGTACT

>Msp_Psg28N1 (Mus spretus; Algerian mouse) WGS CAKLHJ010000017.1

CCTCCCTCTTAACCTGCTGGCTCCTGCCCACCACTGCCAGAGTCACCATTGAATCCTTTCCACCCCAAGTGGTTGAAGGAGAAAATGTTCTTCTACGTGTTGACAATATGCCAGAGAATCTTCTAGTGTTTGGCTGGTACAGAGGAATGACAAATTTGAGGCATGCAATTGCACTGCACTATAGTTTAACAGCGAAGGGGCTGAATCACAGCGGCAGAGAGACATTATACATCAACGGGTCCCTGTGGATCCAAAATGTCACACAGGAGGACACAGGATATTACACCTTTCAAACCATAAGTAAACAAGGAGAAATGGTATCAAATACATCCCTGTACTTGCACGTGTACT

>Msp_Psg29N1 (Mus spretus; Algerian mouse) WGS CAKLHJ010000017.1

CCTCCTTCTTAACCTGCTGGTATCTGTCTACCACTTCCAAAGTCACCATTGAATTATTGCCACCCCAAGTGGTTGAAGGAGAAGATGTTCTTTTCCTTGTCAATAATCTGCCAGGGAATCTTACAGCCTTTGCCTGGTTTAAAGGGAGGACAAATAGGAAACATGGAATTGCACTGTATGCAGTGGCGTCTGACATATATGTACACAGCGATAGAGAGACATTGTACAACAACGGATCCCTGATGATCCACAATGTTACCCAGAAGGACAGAGGTTATTACACCCTACGAACCTTCAATAAACATGCAGAAACTGTATCAACAACATTCACATTCCTCCATGTGAACC

>Msp_Psg30N1 (Mus spretus; Algerian mouse) WGS CAKLHJ010000017.1

CCTCCTTTTTAACCTGCTGTCACCTGCCTACCACTGCACAAATAACCATTGAATTAGAGCCACCCCAAGTGATTGAAGGAGAAAATGTTCTCATACGTGTCAACAATTTGACAGAGAATCTTATAACCTTAGCCTGGTTCAGAGGAATGAGGATTAAGAGCCCTCAAATTGGACAATATACACTGGCCACTAATGTTACTGTGCTGGGGCCTGGTCACAGTGGTAGAGAAATTTTGTACAGCAATGGATCCCTGCAGATTTACAATGTCACCCAGGAGGACATAGGATTCTACAGCCTACGAATCATAAATAAACATGCAGAAATTGTGTCAATAACATCTATATACCTCAATGTGTACT

>Msp_Psg31N1 (Mus spretus; Algerian mouse) WGS CAKLHJ010000017.1

CCTCATTTTTAACCTGCTGTCACCTGCCTGCCACTGCCCAAATAACCACTGAATTAGTGCCACCCCATGTGATTGAAGGAGAAAATGTTCTCATACGTATCAACAATCTGCCAGAGAATCTTACAACCTTAGCCTGGTTCAGAGGAAGGAGGATTAAGAGCCCTCAAATTGGACAATATACACTGGCCACTAATGTTACTGTGCTGGGGCCTGGTCACAGTGGTAGAGAAACTTTGTACAGCAATGGATCCCTGCAGATCTACAATGTCACCCAGGAGGACATAGGATTCTACAGCCTACGAGTCATGAATAGACATGCAGAAATTGTGTCAATAACATCCATATACCTCAACGTGTACT

>Msp_Psg32N1 (Mus spretus; Algerian mouse) WGS CAKLHJ010000017.1

CTTTTCTTTTAACCTCTTGGTTCCTGCCCACCACTGTCCAAGTCACCATTGAATTAGTGCCACCACAAGTGGCTGAAGGAGAAAATGTCCTTATTATTGTTTACAGTCTGCCAGAGGATCTTACAGCCATAGCCTGGTTCAAAGGAGTGACAAATATGAACCTCGGAATTGCATTGTATGCACTGGCCTCTAACATCAGCGTGAAAGGGCCCGAACACAGTGGTAGAGAGACAGTGTTCAGCAATGGATCCCTGTTGCTTCACAATGTCACCCAGAAGGACACCGGATTCTATACTATACGGACCTTAAATAGACATGGAAAAATTGTATCCACAACATCCATATACCTCCACGTGTACA

>Mun_Ceacam9N_P (Meriones unguiculatus; Mongolian gerbil strain 243) WGS PVJK01000749.1

CCTCCCTCTTAACCTACTGGACCACACCAGCCACTGCCGAGCTCACTGGTGCCACCCGTGGTTGCTGAAGATGGAAACTCCATTCTGTTTGTGCATAAAATGCCTTTGAACGCCCAGGCATTTTACTAGTACAAACAGGAAGATCCCCCAAAGAGCTACACGGTACTTAACGCCCACTAATACAGCTTTCCCAGATGCCCCAACACAGCTCAGGAAACTGTGTTCTGCAGTGGATCCCTGCTGACCAGAGGCGTCACCCAGGCTGACGGTGGAGTCTACACCTTACGAACATTTAACCCAGAAATGGAAAGCGAATTAACACAAGTGCATTGGGAAGTACACG

>Mun_Ceacam15N (Meriones unguiculatus; Mongolian gerbil strain 243) WSG VFHZ01014783.1

CCTCACTTTTAACCTGCTGGAACTCACCTGCCGCTGCAGCCCGACTAACTAAAGAAATGCGGTTCTCTGCTGCCGAAGGGGCAAAGGTTCTTCTCCATGTTCCTAACCAGGAAGAGAATCTTCTCTCCTTTTCCTGGTACAAAGGGAAAAATGAACATGAAAATTTTACAATTGCACGTTATGATAAGGCCACAGATGTACTTAAACTTGGAGATAAAACCAGCGGCAGAGAAGACATATATAAGGATGGATCCATGATGCTCCGGTCCGTCACCCAGGAAGACACAGGATTCTACACTTTAGAAACCTTTGAAGCACACAATCAGCGTGAAATAACATATGTCCACCTCCAAGTGTACAG

>Mun_Psg1N1 (Meriones unguiculatus; Mongolian gerbil strain 243) WGS VFHZ01017532.1

CCTCCCTTTTAGCCTGCTGGCACCTGCTCACCACTGCCGAAGTCACCATTGAATTAGTGCCGCCCCACGCGGTTGAAGGAGAAAATGTCATGTTCCTTGTTCACAATCTGCCGAAGAAGGCTATAGGCCTCGCCTGGTTCAAAGGAAGAACAAACAGGAGCCTTGGAATTGCAGTGTACTCTCTGACAGCTAAAGTGGGTGTGGAGGGGCCTCTGTACAGTGGGAGAGAGAGGGTGTACAGCAACGGATCTCTGCGAATTGACAACATCACCCGGGGGGACACGGGATACTACACCCTCCAAACCTTCAATAGACAATCCGAAACCGAGTCAATGGTGTCTACATACCTCCACGTGAACA

>Mun_Psg2N1 (Meriones unguiculatus; Mongolian gerbil strain 243) WGS VFHZ01017532.1

CCTCCCTTTTAACCTGCTGGCACCTGTCCACCACTGCCGACGTCATCATTGAATTAGTTCCACCTCAAGTGGTGGAAGGAGAAAATGTCCTTCTCCTCGTCCACAATCTTCCAAGGAATCTTATAGCCCTAGCCTGGTACAAAGGGACGACTAATGTGAACACGAAAATTGTACTGTATGCACTGAACACCGATGTAAGTGTGCTGGGGCCTGTACACAACAGCAAAGGCAGCATGTACAGGAATGGATCTCTGCGGATTGACAATGTCACCCTGGGGGACACAGGATACTACACCCTCCGAACCTTTAATAGACAGGTAGAAACTGTATCAGTGACATCTACGTACCTCCACGTGAACA

>Mun_Psg3N1 (Meriones unguiculatus; Mongolian gerbil strain 243) WGS NHTI01000426.1 CTTGCATTCACCCTTCTGCCACTGACCAGCTCACTATCGAACCAATGCCACCCAATGTTGATGAAGGGAAAAATGCTCTCCTACTTGTTCATAACATCCCAGAGAACCTTCGATCCTTTTCCTGGTACAAAGGGGTAGCCACTGTCAAGAGACATGAGATTGCACGGAATGTCATAAAAACTAACAAGAGTGTTCTGGGACCTGCACACACCGGCAGAGAGACAGTGTACACTAATGGATCCCTGCTACTGCACAATACCACCCGGGAAGACGCTGGATTCTACACCCTACGAACCCTAAATACACGGCGTGAAAGTCAAGAAACACACGTGTACCTCCATATATACA

>Nle_Ceacam15N (Neotoma lepida; (desert woodrat) WGS LZPO01097217.1

CATCACTTCTTGCCTGCTGGGACTCATCCACCGTGGCGCTAAGAACTACAGAAATGCGGTTTTCTGCAGCTGAAGGAGGGAAGGTTCTTTTCTCTGTTCCTATTCAGGCAGAGAACCTGCTCTCCTTTCACTGGTACAAAGGGAAAGATCTAAACAAAGATTTTACAATTGCCCATTATGAAAAGAACACAGATTTACTTGAACTTGGGAATAAAACCAGCGGCAAGGAAGAGATATATAAGGACGGATCCATGATGCTCCAGGACGTCACCCAGGAAGACACGGGGATCTACACCCTAGAAACCTTAGGAACACATAATCAAGTTGAAATAACACATGTCCACCTCCAAGTGTACA

>Nle_Psg1N1 (Neotoma lepida; desert woodrat) WGS LZPO01097126.1

CCTCCCTTTTAACCTGCCGGCATCTTTCCATCAGTGATTATGTCACCATGAAATCTGTCCCACCACAAGTGGCCAATGGAGACAACGTCCTTCTTATTGTCCACAACCTGCCAGAGGATCTTGTAACCTTTGCCTGGTTTAAAGGGGAGACAGGCATGAACCTTGGAATTGCAATATATTCACTGGACAAAGATTCAAGCATGCCAGGGCCTGGATATAGCGGTAGAGAGACATTGTACTGCAATGGATCCCTGCTGCTTCAAAACGTCAATGAGAAGGACACAGGATTATACACCCTGCAAACCTTAGGCAGACATGGAGATGTTCTGTCAACAACAACCATGCACCTTCACGTGTACC

>Nle_Psg3N1_P (Neotoma lepida; desert woodrat) WGS LZPO01097126.1

CCTCCCTTTTAACCTCCTGGCACTTTTCTACCACTGCCCATGTGACAACTGAATCAGTGCCACCCCTAGTGGCCGAAGAAGAAAACGTCCTTTTCCTTGTCCATGATCTGCCAGAAAATCTCATAGCCTTCGCCCGGTTCAAAGGACTAAGAAATATGAAACAAGGAACTGCAATATATACACTGCACAACAATTTAAGTGTGACAGGGCCTGCACACAGTGGTAGAGAGACAATATATCACAATGGGTCTCTGTTGCTTGAAAATGTCACCCAGAAGGACACAGGATTCTATACCCTATGAACCTATAATAGACGTGCAAAAATCGTATCAACAACATCCACGTACCTCCAAGTGCACG

>Nle_Psg4N1 (Neotoma lepida; desert woodrat) WGS LZPO01109967.1

CCTTCCTCTTAACCTTCTGGCCCCTGACCATCACTGCCCATGTGACCATTGAATCACTGCCACCCCAAGTGGCTGAAGGAGAAAACATCCTTTTCCTTGTCCATGATCTACCAGNGAATCTTACATCCTTTGCCTGGTTCAAAGGGCTAACAAATATGACACAAGGAATTGCATGGTATACANTGGACAACAATTTATATGGGCCAGGGCCTGTGCACAGCGGTAGAGAGACAGTNTATTGCAATGGATCCCTGCTGCTCCAAAATGTCACCCAGAAGGACTCAGGAACCTATACCCTACAAATGTATAATAGACATGGAAAAATCATATCAACATCATCCATATACCTCCACTTGCCCG

>Nle_Psg5N1 (Neotoma lepida; desert woodrat) WGS LZPO01034778.1

CCTCCCTTTTAACCTTCTGGCACCTGTCCATCACTGCCCATGTGACCATTGAGTCACTGCCACTCCAAGTGGCTGAAGGAGAAAACGTCCTTTTCCTTGTCCATGGTCTGCCAGAGAATCTTGCAACCTTTGCCTGGTTCAAAGGGCTAACAAATATGACACAAAGAATTGCAATGTATACAAAGGACAACAATTTAAGTACGCCAGGACATGTGAACATCAGTAGAGAGACAATATATCATAACGGATCCCTGTTACTCAAAAATGTCACCCAGAAGGACACAGGAATCTATACTCTACGAACCTATAATAGACATGGAAAAATAGTATCAACAACATCCATGTACCTACACGTGTCCG

>Nle_Psg6N1 (Neotoma lepida; desert woodrat) WGS LZPO01027714.1

CCTCCCTTTTAAGCTTCTGGCACCTGTCCAGCACTGCCCATGTGACCATTGAATTAGTTCCACCCCAAGTAGCTGAAAGAGAAAACGTCCTTTTTCTTGTCCGTGATCTGCCAAAGAATCTTATAGCCTTTGCCTGGTTCAAAGGGCTAACAAATATGACACATGGAATTGCATGGTATACACTGGACAACAATTTACGTGGGCCAGGGCCTGTGCACAGTGGTAGAGAGACAGTGTATCGAAATGGATCCCTGCTGCTCCAAAATGTCACCCTGGAGGACACAGGAACCTATACCCTGCGAATCTTTAATAGACGTGGAAAAATCATATCAACAACATCCATATACCTCCACGTGCCTG

>Nle_Psg7N1 (Neotoma lepida; desert woodrat) WGS LZPO01018103.1

TCTCCCTTTTAACCTCCTGGCTCCTGTCCACCACTGTCACTGTAACCACTGAATCAATGCTACCCCTTGCGGCTGAAGGAGAAAGCATCCTTGTCCTTGTCCAAGGTCTTCCAGAGAATATTACAGCCTTAGCTTGGTTCAAAGGACTAACAAATATGACACAAGGAATTGCAGCGTATGCACTNAACAACAATNTAAGTTGTCCAGGGCCGGTACACAGTGGTAGAGAGACAATATATCGCAATGGATCCCTTCTGCTCAAAAACGTNAATCAGAACGACACAGGATTCTATACCCTACGAACCTATAATAGACTTGGAAAAATCNTATCANCATCATCCATGTACCTCCATGTGCGTG

>Nle_Psg8N1 (Neotoma lepida; desert woodrat) WGS LZPO01018704.1

CCTCCCTTTTAACCTGCTGGCACCTGCTCACCACTGCCAAAGTCACCATTGAATCAGTGCCACCCCAAGTGGTNGAAGGAGAAAACGTCCTTTTACGTGTGCATAATCTACCAGAGAACCTTCTAACCTTCGTCTGGCACAAAGGGGCGAGGGATATGAGCCTTAGAATTGCACTATACTTACCGGCTAAGAACTTAAGTCTGACAGGTCCTGTACACAGTGGTAGAGAGACAGTGTACAGCAATGGATCCCTGCAGATCCACAATGTCACCCAGAAGGACACAGGATTCTATACCTTACGAATCATGAATAGACACATAGGAGTCGTATCAATAACCACCATGTACCTTCACGTGTACA

>Nle_Psg11N1 (Neotoma lepida; desert woodrat) WGS LZPO01048405.1

CCTCCCTTTTAACCTCCTGGCATCTGTCCACTTCTGCCCATGTGGCCACTAAAACAGTGCCACCCCAAGTAGCTGAAGGAGAAAACGTCCTTTTCTATGTGTATAGTCTTCCAGAGGATATTGTAGCCTTTGTCTGGTTCAAAGGAGAAAGAATTGTGAACAATATGAAACAAGCAATTGCAATATATGGACTGCACCTCAATATAAGTGGTCCAGCACCTGCGAACAGTGGTAGAGAGACAATATATCGCAATGGATCCCTGCTGTTTGAAAAGGTCACCCAGAAGGACACAGGATTTTACACCTTCCGGACCTATAATAGACATGCAGAAATCGTATCAACAACATCCATGTACCTCCATGTGGACA

>Nle_Psg13N1_P (Neotoma lepida; desert woodrat) WGS LZPO01018032.1

CCTCCCTTTTAACCTGTTGGCACCTGCCCACCACTGCCCAAGTCACCATTGAGTTAGTGCCACCCCAAGTGGTTGAAGGAGAAAACATCATTTTCCATGTCCGCAATCTGCCAGAGAATCTTTTAGGCTTATCCTGGTTTAAAGAGACAACGAATATGAAGCATAGAGTTGCAAGCTATGAAATGGACTACAATCAAGCTTTTCTAGGGGAGGCACACAGTGGTAGAGAGACAGTGTACCGCAATGGATCCCTGTGGATTCAAAATGTCACCTAGAATGACTCAGGAGTCTATATCCTAAGAAGCAAAAATAGACAAGTAAGAATTGTGTCATCAACATACATATATCTTCATGTGTACA

>Nle_Psg14N1 (Neotoma lepida; desert woodrat) WGS LZPO01011879.1

CCTCCCTTTTAACCTGTTGGCACCTGGCCCCCACTGCCCAAGTCACCATTGAATCAGTGCCACCCTATGTGGTTGAAGGAGAAAGCAATCTTTTCCTTGTCCACAATCTGCCAGAGAATCTTTTAGCCATATCCTGGTTTAAATGGGCAAGGAATATGAACCATGGAATTGCAACCTATGCACTGAAGTACAATATAACTGTGCCGGGGGCTGCACACAGTGGCAGAGAGACAGTGTACCCAAACGGATCCCTGTGGATTCAAAATGTCACCTATAAGGACACAGGATTCTATATCCTACTAACCATAAGTAAAGAAGTAAAAATTGTATCAACAACATACATACACCTTCATGTGTACA

>Nle_Psg17N1 (Neotoma lepida; desert woodrat) WGS LZPO01008140.1

CCTCCCTTTTAACCTGCTGGTATCCACCCACCACTGCCCAAGTCACCATTGAATCAGTGCCACTCCAAGTGGTTGAAGGAGAAAACGTCCTTCTACGTGCCNACAANATGCCAGAGAATCTTCTAGCTTTTTCCTGGTACAAAGGTGTGAGGAGCATGAACTTCAGAATCGCACTATATGCACTGAACACTAATCTAAGTATCATGGGGCCTGAACATAGTGGCAGAGAAACAGTGTACAGTAATGGATCCCTGTGGCTCAAAAATGTCACCCAGAAGGACACAGGATTCTATACCCTACAGACAGTAAATAGACGTGGAAAAATTGTATCTACAACAACCATGTACCTCCATGTGTACA

>Nle_Psg19N1 (Neotoma lepida; desert woodrat) WGS LZPO01117471.1

CCTCCCTTTTANTCTGCTGTCATTCACCCACCACGGCCAAAATCACCATTGAATCAGTGCCACTCAGTGTGTTCGAAGGAGACAATGTCCTTCTACATGTCCACAATCTGCCAACAGATCTTCTAGCCTTTGCTTGGCTCAAAAGGCTGCGAAATAGGCCACACAGAATTGCACTCTATATACTGAACATCAATGTAATTGTGCCGAGGCCTNTACCCAGTGGTAGAATGAGAGTGTATCGCAATGGATCCCTGTGGATTCAGAATGTCACCTATAAAGACTCNGGATACTACACCCTAGAGACCATAAATAGACGTACAAGTATTTTATCAACAGCAACCATGTACCTCCATGTGCACC

>Nle_Psg23N1 (Neotoma lepida; desert woodrat) WGS LZPO01061754.1

CCTCCCTTTTAACCTGTTGGCACCTACCCACCACTGTCCAAGTCATGATTGAATTAGTGCCACCTGATGTGGTTGAAGGAGAAAATGTCCTTCTCCTTGTTCGCGATCTGCCAGAGGATCATGAAGCCTTTGCCTGGTACAAAGGGGCAAGAAATATGAACTTTGGAATTGTATTCTATTCACTGGCCAGTAATTTAACAGTGACCGGGCCTGAATACAGTGGTAGAGAGACAGTGTACAGAAATGGATCCCTGCGTCTCCAAGATGTCACCCAAAAGGACACAGAATTCTATACCCTACGATCCATAAATGGACAGAAAAAAATCATATCAACAACATCCATCTACCTGCACGTGTACT

>Oto_Ceacam9N (Onychomys torridus; southern grasshopper mouse) OnyTor_scaffold_134326

CCTCCCTCTTGACCTGCTGGAATGCACCTACTGCTGCTGAGCTCACTATTGAATTAGTGCCTCCCATGGTGGCTGAAGGTGGAAACTCCGTTCTGTTTGTGCACAAAATGCCGTTGAACGTCCAGGCATTTTACTGGTACAAACAGAAAGATCCGACCAAGAGCTATGAAGTTGCCCGGTACTTAACACCCGATAACACAACTTCGAAGATGCCTCAACACAGCGGCAGGAAAACGGTGTTCTACAGTGGATCCCTGCTGATCAGAAACGTCACCCAGGCTGACAGCGGATTCTACACCTTACTAACATTTAACACAGAAATGGAAAGTGAACTAACACACGTGTATCTGGAAGTCCACA

>Oto_Ceacam11N1 (Onychomys torridus; southern grasshopper mouse) WGS PVIT010003714.1

CCTCCCTTTTAACCTTCTGGCTGCCTCCTACTGTTGCCCAGCTCACCATTGAATCAGTGCCACCCATTTCTGCTGAAGGGGATAATGTTCTTCTGCTTGTGCACAACCTTCCTGAGAATGTTCAAGCCTTTTCCTGGTACTCAGGAGTTATGGTGCTCAAGAGCCGTGAAATTGCAAGGTGTGCAATAGCTACTAATTCATGTGTGCTGGGGCCTGCACACAGTAGTAGAGAGACAGCATTCAAGAATGGATCTCTGCTGATGAAGAATGTCACCAGGAAGGACTCAGGATACTACATCCTACAAACACATAATAAAAATTCGAGATCTGAAATAACACGTGCGGAATTTTTTGTGCACA

>Oto_Ceacam15N (Onychomys torridus; southern grasshopper mouse) WGS PVIT010129585.1

CCTCACTTTTAACCTGCTGGAACTCACCCACTGCAGCGCTAAGAACTACCAAGGAAATTCGGTTTTCTGCGGCTGAAGGGGGCAAGGTTCTTCTCTCTGTTCCTATTCAGGCAGAGAATCTTATCTCCTTTCACTGGTACAAAGGGAAAGATGAAGACCAAGATTTTACAATTGCCCATTATGAAAAGGACACAGATTTACTTAAACTTGGGAATGCAACCAGCGGCAGGGAAGAGATATATAAGGACGGATCCATGATGCTCCAGGACGTCACCCAGGAAGACACGGGGATCTACACCCTAGAAACCTTTGGAACACATGATCATCTTGAAATAACACATTTCTACCTCCAAGTGTACA

>Oto_Psg1N1 (Onychomys torridus; southern grasshopper mouse) WGS PVIT010022511.1

CCTCCATTTTAACCTGCTGGCACCTTTCCACCACTGACCATATCACCATTAAATCTGTCCCATCCCAAGTGGCCAATGGAGACAACGTCCTTCTTCTTGTCAACAATCTGCCAGAAGGTCTTCTAACCTTTGCCTGGTTTAAAGGGGAGACAGGCATGAACCTTGGAATTGCAATATATGCACTGGACAGAGATTTAAGCATACAAGGGCCTGGATATAGTGGTAGAGAGACAGTGTACCGCAATGGATCCCTACTGATCCAAAATGTCAATGAGAAGGACACAGGACTCTACACCCTACAAACCTTAAATGAACATGGAGATGTTCTGTCAATAACAACTATGCGCCTGCACGTGTACC

>Oto_Psg3N1 (Onychomys torridus; southern grasshopper mouse) WGS PVIT010089964.1

CCTCCCTGTTAACCTTCTGGCACCTGTCCACCACTGCGGATGTGACTATTGAATCACTGCCACCCCAAGTGACTGAAGGAGAAAATGTCCTTTTCCTTGTCCGTGATCTGCCAGAAAATATTACGGCCTTAGCCTGGTTCAAAGGACTAAGAAGTATGAAACATGGAATTGCAATATATACACTGCACAAGAATTTAAGTGTGACAGGGCCTGTGCACAGTGGTAGAGAGGCAATATATCACAATGGATCTCTGTTGCTTGAAAATGTCACCCAGAAGGACACAGGATACTATACTCTACGAACCTTTAATAGACGTGCAAAACTCATATCAACAACATCCATTTACCTCCAAGTGCATG

>Oto_Psg4N1 (Onychomys torridus; southern grasshopper mouse) WGS CAJFAA010002655.1

CCTCCCTATTAACCTTCTGGCACCTGTCTACCACTGCGGATGTGGCCATTGAATCACTGCCACCCCAAGTTGCTGAAGGAGAAAATGTCCTTTTCCTTGTCCGTGGTCTGCCAAAGAATCATATAGCCTTTGCCTGGTTCAAAGGGCTAACAAATACGACACATGGAATTGCATGGTATACACTGGACAACAATTTACATGGGCCAGGGCCTGTGAACAGTGGTAGAGAGACAGTGTATCGCAATGGATCCCTGTTGCTTCAAAATATCACCCAGAAGGACACAGGGACCTATACCCTAAAAATCTATAATAGACGTGGAAAAATCATATCAACAACATCCATTTACCTCCATGTGCCTG

>Oto_Psg5N1 (Onychomys torridus; southern grasshopper mouse) WGS CAJFAA010002655.1

CCTCCCTCTTTACTTCCTGGCACCTGCCCACCACTGCCCATGTGACCATTGAGTCACTTCCACTCCAAGTGGTTGAAGGAGAAAACGTCCTTTTCCTTGTCCATGATCTGCCAAAGAATCTTATAACCTTTGCCTGGTTTAAAGGGCTAACAAATATGACACAAAGAATTGCAATGTATACAATGGATAACAATGTAAGTGTGCCAGGACATGTGCACAGTGATAGAGAGACAATATATCATAATGGATCCCTGTTGATTGAAAATGTCACCCAGAAGGACACAGGATTCTATACCCTACGAACCTATAATAGACATGGAAAAACTGTATCAACAACATCCATGTACTTTCATGTATCTG

>Oto_Psg6N1 (Onychomys torridus; southern grasshopper mouse) WGS CAJFAA010002655.1

CCTTCCTGTTAACCTTCTGGCACATTTCCACTACTGCCCATGTGTCCATTGAATCACTGCCACCCCAAGTGACTGAAGGAGAAAATGTCCTTTTCTTTGTCCATGGTCTGCCAAAGAATCTTATCGGCTTTGCCTGGCTCAAAAGGCAAAAAAATATGACACAAGGAATTGCATGGTATACATTGGACAACAATTTATGTGGACCAGGGCCTGGGCACAGTGGGAGAGAGATAGTATATCACAATGGATCCCTGCTTCTCCAAAATGTTAGGCAGAAGGACACAGGAAGCTATATCCTACAAACCTATAATAGACGTAGAAAAATCATATCAACAGCATCCATTTACCTCCATGTGCATG

>Oto_Psg7N1 (Onychomys torridus; southern grasshopper mouse) WGS CAJFAA010002655.1

CCTCCCTTTTAACCTTCTGGCTCCCATCCACCACTGCCACTGTAAACACTGAATCAATGCCACTCCTTGTGGCTGAAGGAGAAAACGTCCTTTTCCCTGTCCAAGATCTTCCAGAGAATATTATAGCCTTAGCCTGGTTCAAAGGACTAACCAAGATGACACAAGGAATTGCCTTGTATGCTCTGCACAGCAATTTAAGTTGTCCAGGTTCTGCGCACAGCGGTAGAGAGACAATATATCGCAATGGATCCCTGCTGCTGGAAAAGGTCACCCAGAAGGACACAGGATTTTATACCCTACGAACCTTTAATAGACACAGAAAAATCATGTCCACAGCATCCATTTACCTCCATGTGCATG

>Oto_Psg8N1 (Onychomys torridus; southern grasshopper mouse) WGS PVIT010047406.1

TCTCCCTTTTAACCTGCTGTCACATGCCCACCACTGCCCAAGTCACCATTGAATCAGTGCCACCCCACGTGGTTGAAGGAGAAAATGTCCTCCTACATGTTCATAATCTACCAGAGAATCTTCTAGCCTTTGTCTGGCACAAAGGGGCAAGGAATATGAGCCTTAGAATTGCACTGTATTTACTGGCCAAGGACGTAAGTGTGAAAGGCCCCGTACACAGTGGTAGAGAGACCGTGTACAGCAATGGATCCTTACAGATCAACAATGTCACCCAGAAGGACACAGGATTCTATACCTTACGAACCATTAATAGACGCATAGGAATTGTATCAATAACAACCAAGTACCTTCACGTGCACA

>Oto_Psg9N1 (Onychomys torridus; southern grasshopper mouse) WGS PVIT010001958.1

CCTCCCTGTTAACCTTATGGCACCTGTCCACCACTGCCCACGTGACCATTGAATCACTTCCACCTGAAGTGACTGAAGGCAAAAATGTCCTTTTCCTTGTACGTGATCTTCCAAAGAATCTTATCGCCTTTGCTTGGTTCAAAGGGCAAACAAATATGACACAACGAATTGCATGGTATACATTGGAAAACAATTTGCATGGGCCAGGGCCTGTGCACAGTGGTAGAGAGACAGTGTATCGCAATGGATCCCTGCTGCTCCAAAACGTCACCCAGAAGGACACAGGACCCTATACCCTACAAACTTTTAATAGACACAGAAAAATCCTTTCAACAACATCTATTTACCTCCATGTGCATG

>Oto_Psg10N1 (Onychomys torridus; southern grasshopper mouse) WGS CAJFAA010002655.1

CCTTCCTCTTAACCTTCTGGCATCTGTCTACCATGGCCCACGTGACTATTGAGTCACTTCCACTCCAAGTGACTGAAGGAGAAAATGTCCTTTTCCTTGTCCATGATCTCCCAGAGAATCTTACAACCTTTGCCTGGTTCAAAGGTCTAACAAATATGACACAAAGAATTGCAATTTGTACAATGGACAACAATTTAAGTCTTCCAGGACCTGTGCACACTGATAGAGAGACAATATATTGCAATGGATCCCTGTTGCTCAAAAATGTCAACCAAAAGGACACAGAAAATTATACCCTACAAACCTATAATAGACATGGAAAAATTGTCTCAACAACATCCATGTACCTCCATGTGTCTG

>Oto_Psg11N1 (Onychomys torridus; southern grasshopper mouse) WGS CAJFAA010002655.1

CCTGCCTTTTAACCTCTTGGCGTCTGTCCACCACTGCCCATGTGACCACTAAAATAGTGCCACCCCAAGTGGCTGAAGGAGAAAACGTCCTTTTCCTGGTGCATGAGCTTCCAGAGAATATTATAGGCTTTGCCTGGTTCAAAGACCTAACAAATATGAAAACAGCAATTGCAGTATATGGACTGCACATCAATTTAAGCGCACCGGGGCCTGTGCACAGTGGTAGAGAAACACTATATCGCAATGGATCCCTGCTGCTTGAAAAAGTCACCAAGAAGGACATAGGATTTTATACCCTGCGGACCTATGATAAATATGTAAAAATCGTATCAACAACATCCACGTACCTCCATGTGGACA

>Oto_Psg12N1 (Onychomys torridus; southern grasshopper mouse) WGS PVIT010031371.1

CCTCCCTTTTAACCTTCTGGTATCCATACTCCACTGCCCAAGTCACCATTGAACTACTGCCGCCCAATGTGTTTGAAGGAGACAATGTCCTTCTTCATGTCCACAATCTGCCAGAGAATCTTCTAGCCTTTTCTTGGTTCAAAGGGGTGACAAATTTGAAACGTGGAATTGCAATCTATTCACTGAAATACAATTTAAGTGTCACAGGGCCTGTACACAGTGGAAGAGAGACAGTGTACAGCAATGGATCCCTGCTGCTCCAGCATGTCACCCACAAGGACACAGGATTCTATACCCTACGAACCATAAGTAGACAAGGAGAAATTGTATCAATAACATCCATGTACCTCCACGTGCACA

>Oto_Psg14N1 Onychomys torridus; southern grasshopper mouse) WGS PVIT010020713.1

CCTCCCTTTTAACCTGTTGGTACCGGACCACCACTGCCCAAGTCACCATTGAATCAGTGCCTCCCCATGTAGTCGAAGGAGAAAGCAACCTTTTCCTTGTCTACAATCTGCCAGAGAATCTTTTAACCATATCCTGGTTTAAAGAAGGAGCTAATATGGACCATAAAATTGCCATCTACTCACTGAAATATAATATAGCTGTGCCGGGGCCAGCACACAGTGGTAGAGAGACAGTGTACCCAAATGGATCCCTGTGGATTCAAAATGTCACCCATGATGACACGGGATTCTATATCCTACAAACCAGAAGTAGACAAGTAAAAACTGTATCAACAACATACATACACCTTCATGTGTACA

>Oto_Psg16N1 (Onychomys torridus; southern grasshopper mouse) WGSPVIT010002880.1

CCTCCCTTTTAACCTGTTGGTACCGGACCACCACTGCCCAAGTCACCATTGAATCAGTGCCTCCCCATGTAGTTGAAGGAGAAAGCAACCTTTTCCTTGTCCACAATCTGCCAGAGAAACTTTTAGCCATATCCTGGTTTAAAGAAGGAGCAAATATGGACCATAGAATTGTGACCTACACACTGAAATACAATATATCTGTGCCAGGGGAAGCACACAGTGGTAGAGAGACAGTGTATCCAAATGGATCCCTGTGGATTCAAAATGTCACCTATAAGGACACAGGATTCTATATCCTACGAACCATAAGTAGACAAGTACAAACTGTATCAAGAACATACATACACCTTCATGTGTACA

>Oto_Psg17N1 (Onychomys torridus; southern grasshopper mouse) WGS CAJFAA010002655.1

CCTCCCTTTTAACCTGCTGGCACCTATCCACCACTGCACAAGTTACCATTGAATCAGTGCCTCCCCAAGTGTTTGAAGGAGAAAACGTCCTTCTACGTGCCAACAACCTCCCAGAGAATCTTCTAGCTTTTTCCTGGTACAAGGAAGTGAGGAATATGAATGTCAGAATTGCACTATTTGCACTGAACACTAATCTAAGTGTGATGGGGCCTGAACAAAGTGACAGAGAAACAGTGTACAGCAATGGATCCCTTTGGCTCAAAAATGTCACCAAGAAGGACACAGGATTCTATACTCTCCAAACAGTAAATAGAGGTGGAAAAATTGTATCTACAACAACCATGTACTTCCATGTATACA

>Oto_Psg18N1 (Onychomys torridus; southern grasshopper mouse) WGS PVIT010030024.1

CCTCCATTTTAACCTGCTGGCACCTTTCAACCACTGATGATTTTACCGTGAAACCTGTCCCACCTTATGTGGCCAATGGAGACAATGTCCTTCTACATGTCCACAGTCTGCCAGAGAATCTTCTAGCCTTTGCCTGGATCAAAGGGGCGATAAGCATGAATCATACAATTGCAATATATATACCAAACAAAAAGTTAAGTGTGCCAGGGCATTTATATAGTGGCAGAGAGACAGTGTATGGCAATGGATCCCTGCTGCTCCAAAATGTCAATGAGAAGGACACAGGAATTTATACCCTACAAACCTTTAATAGACGCACAGATACTGTGTTGCAAACATCCATGAACCTCTATGTTCACA

>Oto_Psg19N1_P (Onychomys torridus; southern grasshopper mouse) WGS PVIT010275663.1

CCTCCTTTTAATCTGCTTTCATTTGGCCACCACTGCCAAAGACACCACTGAATCAGAGCCACTCAATGTGTTCAAAGGAGACAATGTCCTTCTACATGTCCACAGTCTGCCAGAGAATCTTCTAGCCTTTGCTTGGTTCAAAAGGCTGACAAAATGAAACACAGAATTGCACTCTATGCACTGAACATTAATTTAATTGTTCCAGGGCCTGTACATAGTGGTAGAGAGACAGTGTACTACAATAGATCCCTGTGGACTCAGAATGTCACCTGTAACGACACAGGATTCTACACCCTACAGACCATAAATAGACATGGAAGAACTGTATTAACAACAGCAATGTACTTCCATGTGTACA

>Oto_Psg20N1_P_partial (Onychomys torridus; southern grasshopper mouse) WGS PVIT010264932.1

nnnnnnnnnnnnnnnnnnnnnnnnnnnnCCACCACTGCCAAAGTCACCACTGAATCAGAGCCACTCAATGTGTTCAAAGGAGACAATGTCCTTCTACATGTCCACAGTCTGCCAGAGAATCTTCTAGCCTTTGCTTGGTTCAAAAGGCTGACAAAATGAAACACAGAATTGCACTCTATGCACTGAACATTGATTTAATTGTTCCAGGGCCTGTACACAGTGGTAGAGAGACAGTGTACTGCAACGGATCCCTGTGGACTCAGAATGTCACCTGTAACGACACAGGATTCTACACCCTACAGACCATAAATAGACATGGAAGAACTGTATTAACAACAGCAATGTACCTATATGTGTACA

>Oto_Psg22N1_P (Onychomys torridus; southern grasshopper mouse) WGS PVIT010150115.1

TCTTCCTTTTAACCTGCTGTCACATGCCCACCACTGCCCAAGTCACCATTGAATCAGTGCCACCCCACGTGGTTGAAGGAGAAAACATCCTCCTACGTGTTCATCATCTACCAGAGAATCTTCTAGCCTTTGTCTGGCACAAAGGGGCGAGGAATATGAGCCTTGGAATTGCACTATATTCACTGACCAAGGATGCAAGTGTGACAGGTCCTGTACACAGTGGTAGAGAGACAGTGTACAGCAACGGATCCCTGCAGATTCACAATGTCTCTCAGAAGGACACAGGATTCTATACCTTTTGAACTATAAATGCACAAGTAGGAGTTGTATTAGTAACAACATACCTTCATGTGCACA

>Oto_Psg23N1 (Onychomys torridus; southern grasshopper mouse) WGS PVIT010008418.1

CCTCTCTTTTAACCTGTTGGCACCTACCCACCACTGTCCAAGTCATTATTGAATTAGTGCCACCTGATGTGGTTGAAGGAGAAAATGTCCTTCTCCTTGTCCGCAATCTGCCAGAGAATCTTGAAGCCTTTGCCTGGCACAAAGGAGTGACAAATATGAACCTTGGAATTGTATTGTATTCTCTGACCACTAATTTAACAGTGGCAGGGCCTGAATACAGTGGTAGAGAGACAGTGTACAGAAATGGATCCCTGCATCTCCAAGATGTCACCCAGAAGGACACAGGATTCTATACATTACGATCCATAAACAGACATAAAGAAATCATATCAACAACATCCATATACCTCCACGTGTACT

>Oto_Psg24N1 (Onychomys torridus; southern grasshopper mouse) WGS CAJFAA010002655.1

CCTCCATTTTAACCTGCTGGCACCTTTCCACCACTGATCATTTTACCATGGAATCTGTCCCACCTCATGTGGCCAATGGAGACAATATCCTTTTCCTTGTTCACAATCTGCCAAAGAATCTTCTAGCCTTCGCCTGGATCAAAGGAGCAATGAGCATGAATGATGCAATCATAGTATATATACCAAACAAAAATTTAAGTGTGCCAGGGCGTTTTTACAGTGGTAGAGAGACAGTGTATGGCAATGGATCCCTGCTGATCCAAAATATCAACCAGAAGGACACAGGAATTTATTCTCTACAAGCCTTTCATAGACGCACAGATACCATGTCACAAATATCCACATACCTCTTTGTGAACA

>Ozi_Ceacam9N (Ondatra zibethicus; muskrat) 6.6.22 WGS PVIU01005340.1

CCTTCCTCTTAACCTGCTGGAATGCACCCACCACTGCCGAACTCACTATTGAATTAGTGCCCCCCATGGTTGCTGAAGGTGGAAACTCCGTCCTATTTGTCCATAAAATGCCATTGAACGTCCAGTCATTTTACTGGTACAAACAGAAAGATCAGACCAAGAGCTATGAAGTTGCACGCTACTTAACACCCGATAACACAACGTCAAAGATGCCTCAACACAGTGGTAGGAAAACGGTATTCTACAGTGGATCCCTACTGATCAGAAACGTCACCCAGGCTGACAGTGGATTCTACACCTTACTAACGTTCAACACAGAAATGCAAACTGAACTCACACACGTACATCTGGAAGTATATA

>Ozi_Psg1N1 (Ondatra zibethicus; muskrat) WGS PVIU01004945.1

TCTTCCTTTTAACCTGTTGGCACCTGCCCACCACTGCCCAAGTCACTATTGAATTAGTGCCGCCCCAAGTAGTTGAAGGAGAAAATGTTCTTCTACGTGTTCATAATCTACCAGAGAATCTTCTAGCCTTTGTCTGGCACAAAGGGGTGAGGAATATGAGCCTTGGAATCGAACTATATTCACTGGCCAAGGGTTTAAGCGTGACAGGGCCCATACACAGCGGGAGAGAGACAGTGTACAGCAACGGATCCCTGCAGATCTACAATGTCACCCAGAAGGACACAGGATTCTACACCTTTCGAACCATAAATGGACATGTAGGAGTTGTATCAGTAACAACCACGTACCTTCACGTGTACA

>Ozi_Psg2N1 (Ondatra zibethicus; muskrat) WGS PVIU01028915.1

CCTACCTTTTAACCTGTTGGCACCTGCCCACCACTGTCCAAGTCATTGTTGATTTAGTGCCACCCCATGTTGTTGAAGGAGAAAACGTCCTTCTTCGTGTCCGCAATCTGCCAGAGGATCTTGTAGCCTTTGTCTGGCACAAAGGGGTGACAAAGATGAACCTCGGAATTGTACTTTATTCACTGACCACTAATTTAAACATCACGGGGCCTGGACACAGTGGTAGAGAGATAGTGTACAGAAATGGATCTCTGCACCTCCAAAATGTCACCCAGAAGGACACGGGATTCTACACGCTACGATCTTTAAATAAGCATAAAGGAATTGTATCAACAACATCTATATACCTGCATGTATACT

>Ozi_Psg3N1 (Ondatra zibethicus; muskrat) WGS PVIU01007063.1

CCTCCCTTTTCTCCTTCTGGCATCTCCCCACTACTGCCCATGTGTCCACTGAATCAGTGCCACCCCTAGTGGCTGAAGGAGATAACGTCCTTATCCTTGTCAACAATCTGCCAGAGAATCTTTTAGCCTTAGCCTGGTTCAAAGGGCTAACAAATATGAAACAAGGAATTGCATTATATGCACTGCACAAAAATGTAAGTGCTACAGGGCCTGTGCACAGTGGCAGAGAGACAATATATCACAATGGATCCTTGTTGATTGAAAAACTCACCCAGAAGGACACAGGATTCTACACCTTTCGAGCCTATAATAGACGCGGAAGAATTGTATCAACCACATCCACCTACCTCCATGTGCAAG

>Ozi_Psg4N1 (Ondatra zibethicus; muskrat) WGS PVIU01007514.1

CTTCACTTTTAGGCTGCTGCCTATCCACCACTGACTATATCACCATTAAGTCTGTCCCACCCCATGTGGCCAGTGGAGATGACGTCTTTCTCCATGTCCACAATCTGCCAGAGGATCTTCTAGCCTTCGCCTGGTTCAAAGGGGTGACAAGCATGAAGCACGGAATTGCAGTATATGCACTGAACAAAAATTTAAGTGCGACAGGGCCTGCACATAGTGGTAGAGAGACAGTGTACCACAATGGATCCCTGCTGATCCAAAGTGTCACCGAGAAGGACACAGGATTCTATACCCTACGAACCTTAGATAGACACGGAGAGATTGTGTCAACAACAACCACGCACCTCTATGTGTACC

>Ozi_Psg5N1 (Ondatra zibethicus; muskrat) WGS PVIU01012565.1

TGTCGCTTTTAAGCTCCTGCCATCTGCCCACAAATGCCCGTGTGATTATTGAAAAAGTGCCAACCCTAGTTGCTGAAGGAGATAACATCATTTTCCATGTCCATGATCTGCCAGAGAATATTACAACCTTAGCCTGGTTCAAAGGTGTAAGAAATGCGACACAAGGAATTGCAGCATATGCACCGCTCTTCAAATTGAGTAGGTCAGGTCCTATGTACAGTGGTAGAGAGACAATATATCGCAATGGATCCCTGCTGATAGAAAACGTCAACCCGACAGACACTGGATTCTATACCCTACGAACTTATAATAGACATGGAACTAGGATATCAATATCATCTACGTACCTCCAAGTGCATG

>Ozi_Psg6N1 (Ondatra zibethicus; muskrat) WGS PVIU01018536.1

TTTCCCTTTTAACCTCCTGGCACCTGTCCACCGCTGCCCATATAACTACTGAGTCAGTGCCAACCCGAGTGGTTGAAGGAGGAAACGTCCTTTTCCTTGTGCATGATCTGCCAGATAATACTAAAACTTTAGCTTGGTTCAAAGCTCTAAGAAATATGACAGAAGGAATTGCAGCATATTCACTATCCTACAATTTAAGTAGGTCAGGTCCTCTGTACAGTGGTAGAGAGACAATATATCACAATGGATCCCTGCTGATAGAAAATGTCAACCACAAGGACACAGGAGTCTATATCCTAGAAACATATAACAGACGAGGAAAAGTCATATCAACAACAACCATGTACCTCCAAGTTAATG

>Ozi_Psg8N1 (Ondatra zibethicus; muskrat) WGS PVIU01028669.1

TCTTCCTTTTAACCTGTTGGCACCTGCCCACCACTGCCCAAATCACCATTGAATCAGTGCCACCCCAAGTGGTTGAAGGAGAAAACGTTCTTCTGCGTGTTCATAATCTACCAGAGAATCTTCTAGCCTTTGTCTGGCACAAAGGGGTGAGGAATATGAGCCTTGGAATTGCACTATATTCAGTGGCCAAGGGTTTAAGTGTGACAGGGCCCATACACAGCGGGAGAGAGACAGTGTACAGCAATGGATCCCTGCAGATCTACAATGTCACCCAGAAGGACACAGGATTCTACACCTTTCGAACCATAAATGGACAAGTAGGAGTCTCATCAAAAACAACCACGTACCTTCACGTGTATA

>Ozi_Psg9N1 (Ondatra zibethicus; muskrat) WGS PVIU01009591.1

CCTTCCTTTTAATCTGTTGTCATTCACCCACCACTGCCAAAGTCATCATTGAATCAGTGCCGCCCAATGTGTTCGAAGGAGACAATGTCCTTCTATATGCCCACAATCTGCCAGAGAATCTTCTAGCCTTTGCTTGGTTCAAAGGACTAACAAATATGAAACGCAGAATTGTACTCTATGAACTGAACAACAATTTAAGTTTGACGGGGCCTGAATACAGCGGTAGAGAGACAGTGTACTGCAATGGATCCCTGTGGATTTCCAATGTCACCCACGTGAACACAGGATTCTATACCCTACGAACCATAAGTAGACATTCAAGAATTGTATCAATAACAACCATCCACCTCCCTGTGTATA

>Ozi_Psg10N1 (Ondatra zibethicus; muskrat) WGS PVIU01007063.1

TCTCCATTTTAAGTTTCTGGCACATGTCCACCACTGCCCATGAGACCACTGAGTCACTGCCACATCAAGTGGTTGAAGGAGAAAACGTCCTTTTGCTTGTCCACGATCTGCCAGAGAATCTTATAGCCTTTGCCTGGTTCAAAGGGCTAACAGATATGACACAAGGAATTGCAGTATATACATTGCACAACAATTTAAGTGCACCAGGGCCTGTGCACAGTAGTAGAGAGACATTGTATAGCAATGGATCCCTGCTGATAGAAAATGTCACCCAGAAAGACACAGGAATCTATACCCTACGAACCTATAATAGAAGTGGAAAAATTGCATCAACAACATCTATGTACCTCCACGTGCATG

>Ozi_Psg11N1 (Ondatra zibethicus; muskrat) 10.5.21 WGS PVIU01009591.1

CCTACCTTTTAACTTACTGGTATCTACCCACCACTGCCCAAGTCACCATCGAATTAGTGCCGCCCAATGTGTTCCAAGGAGAAAATGTCCTTCTAGAGGTCCACAATCTGCCAGAGGATTTTCTAGCCTTTGCTTGGTACAGAGGGGCGACAAACATGAAACGCGGAATTGCAGTCTATGCCAAAAGAAACAATTTAAGAGCATCGGGGCCTGCGTACAGTGGTAGACAGACAGTGTACAGTGATGGATCACTGCTGCTCCAGCGTGTCACCCTCAAGGACACAGGATTCTACACCCTACGAGTCATAAATAGACAAGCAGAAATTGTATCAACAACATCCGTGTTCCTCCATGTGCACA

>Ozi_Psg12N1 (Ondatra zibethicus; muskrat) WGS PVIU01044888.1

CCTCCCTTTTAACCTTTGGGCACCTGTCCATCACTGCCCATGTGATCACTGAATCAGTGCCTCCCCAAGTGGCTGAAGGAGAAAACGTTCTTTTCATTGTCCACAATCTGCCAGTGAATGTTAAATCTTTTGCCTGGTTCAAAGGGCTAAAAATTGAGAAACAAGGAATTGCAATGTATAGACTGCGCAGGAATTTAGTTACAAATGGGCCTATGCACAGTGGTAGAGAGACCATATATCTCAATGGATCCCTGCTGCTTGAAAAGGTCTCCCATATGGACACAGGATTCTATACCCTACAAACCTATAATAGACATGCAAAAATCCTATCAACAACTGCTGTGTATCTCCATGTGCATG

>Ozi_Psg13N1 (Ondatra zibethicus; muskrat) WGS PVIU01009644.1

CTTCCCTTTTAACCTTGTTGCACCTGTCTACCACTGCCCATGTGACCACTGTATCAGTGCCACCCATAGTGTCTGAAGGAGATGACGTCCTGTTCCTTGTCTACAATCTGCCAGAGGAAATTGAATCCTTAGCCTGGTTCAAAGGGCTAGGAAACACAACAGAAGAAATTGCATCATATGCACTGAACAGCAGTTTAAGTAGGCCAGGTCCTGCGCACAGCAGTAGAGAGACAATATATCACAACGGATCCATGCTGTTTGAGAAGGTCAACCTGAAGGACACAGAATTCTATACCCTACAAACCTATAATAGAAGTGGAAAAATCTTATCAACAGCAAGCGTGTACCTCAATGTGTATG

>Ozi_Psg14N1 (Ondatra zibethicus; muskrat) WGS PVIU01012604.1

CCTCCCTTTTAACCTTCTGGCACCTGTTCACCACTGCCCATGAGACCACTGTATCAGTGCCACCCCAAGTGGCCGAAGGAGATGACGTTCTGTTCCTTGTCCACAATCTGCCAGAGAAAATTAAATCCTTAGCCTGGTTCAAAGGGCTAGGGAACACAACAGAAGAAATTGCATCATATGCACTGCACAGCAATTTAAGTAGGCCAGGTGCTGCGCACAGCAATAGAGAGACAATATATCACAACGGATCCATGCTGTTTGAAAAGGTCATCCTGAAGGACTCAGGATTCTATACGCTACAAACCTATAACAGACGTGGAAAAGCTGTATCAACAACATACGTGATTCTCAATGTGCATG

>Ozi_Psg15N1 (Ondatra zibethicus; muskrat) WGS PPVIU01021207.1

CCTTCCTTTTAACCTTTGGGCACCTGTCCACCACTGCCCTTGTGACCACTGTATCTGTGCCATCCCGAGTGGCTGAAGGAGATGATGTCCTATTCCTTGCCCACAATCTGCCAGAGAAAATTAAAACCATAGCCTGGTTCAGAGGGCCCTCAGATATGACTGCAATATATCGACTGCCCTACAATTTAAGTAGGCCAGGTCCTGCACACAGCGGTAGAGAGACAATATTTCACAATGGATCCATGCTGCTTCAAAAGGCCAACCTGAAGGACACAGGATTCTATACTGTACGAACCTATAATAGACATGGAAATGTCATATCAACAACATACACATACCTCAACGTGTATG

>Ozi_Psg16N1 (Ondatra zibethicus; muskrat) WGS PVIU01013410.1

CCTCCCTTTTAACCTGCTGGCACCTGTCCACCACTGTCAAAATCACAATTGACTCAGTGCCACCCCAAGTGGTTGAAGGAGAAAACGTCCTTCTACATGCCAACAATCTGCCACAGAATCTTCTAACTTTTTCCTGGTTCAAAGGGGTGACAAATATGAACTCCAGGATTGCACTATATACACTGACCACTAATCTAATTGTAACGGGGCCTGAAAATAGTGGTAGAGAAGCTGTGTACAGCAATGGATCCCTGTGGCTCAAAAATGTCACCCAGAAGGACACAGGATTTTATATACTACAGACAGTTAATAGAGGTGGAAAAATTTTATCTACAACAACCACATACCTCCATGTGTATG

>Ozi_Psg19N1 (Ondatra zibethicus; muskrat) WGS PVIU01032737.1

CCTCTCTTTTAACCTCCTGGCACCTGTCTACCACTGCCAATGTGACCATTGAATTAATGCCAACTCCGGTGGCTGAAGGAGATAACATCCTTTTCCTTGTCCACAATCTGCCAAAGGAAATTAAAGCCGTAGCCTGGTTCAAAGGGGTAAGAAATAAGAAAAAACAAATTGCAGTGTATGTACTGCACAAAAATTTAAGTAGGCCAGGTCCTATGCACAGTGGTAGAGAGATAATATATCACAACGGATCCCTGCTGCTTGAAAAGGTCACCCAGAAGGATGAAGGATTCTATACCCTACGAACCTATGACAGAGGTGGAAAATTTGTATCAACCATACCCATTTACCTCCACGTGCACG

>Ozi_Psg20N1_P (Ondatra zibethicus; muskrat) WGS PVIU01036502.1 CTTCCATTTTAATATGTTGGCACCTGCCCACAGCTGTCCAAGTCAGAATTGAATCTGTCCCACCCCAAATAGTCGAAGGGAAAATGTCTTTCTTCTTGTTTACAATCTGCCAGAGAATCTTCTAGCCTTAGCCTGGTCCAAATTGGTGAAAAGTATGAACCATGGAATTGGAACATATTTACTGAACAAAGATTTAAATGTACCAGGGCCTTTACACAGTGGTAGAGAGACAGTGTACAGCAATGGATCCCTGCTGCTGAGAAATATCACCAAGAAGGATACAGGATTCTATACCCTACGAATCTTAAATAGACATGTAGCTATTGTGTCAACAACAACCATGTACCTTCACGTGCACA

>Ozi_Psg21N1 (Ondatra zibethicus; muskrat) WGS PVIU01042331.1 -

CAACCCTTTTAATATGTTGGCACCTGCCCACAGCCGTCCCAGTCAGAATTGAATCTATCCCACCCCAAGTGGTTGAAGGAGAAAACGTCCTTCTTCTTGTCCACAATCTGCCAGAGAATCTTCTAGCTTTAGTCTGGTCCAAAGGCATGGAAAGTATGCACCATGGAATTGGAACATATTTACTGAACGAAGATTTAAGTGTACCAGGGCCTTTACACAGTGGTAGAGAGACAGTGTACAGCAATGGATCCCTGCTGCTGAGAAATATCACCAAGAAGGATACAGGATTCTATACCCTACGAATCTTAAATAGACATGTAGCTATTGTGTCAACAACAAGCATGTACCTTCATGTGCACA

>Ozi_Psg22N1_P (Ondatra zibethicus; muskrat) WGS PVIU01022727.1

CCTTCCTTTTAATCTGCTGTCAGTCACCCACCACTGCTGAAGTCATCATTGAATCAGTGCTGCCCAATGTGTTCGAAGGAGACAATGTCTTCTATATGCCCACAATCTGCCAGACAATCTTCTAGCCTTTGCTTGGTTCAAAGGGCTAACAAATATGATACACAGAATTGTACTCCATGAACTGAATTTAAGTTTGACAGGGCCTGAATACAGCGGTAGAGAGACAATGTACTGCAATGGATCCCTGTGGATTTCCAATGTCACCCACGTGGACACAGGATTGTATACCCTATGAACCATAAGTAGACATTCAAGAATTGTATCAATAACAACCATCCACCTCCCTGTGTACA

>Pat_Ceacam9N (Peromyscus attwateri; Texas deermouse) WGS CABHPP010123625.1

CCTCCCTCTTGACCTGCTGGAATGCACCTTCTACTGCCGAGCTCACTATTGAATTAGTGCCTCCCATGGTGGCTGAAGGTGGAAACTCCGTTCTATTTGTGCACAAAATGCCGTTGAACGTCCAGGCATTTTACTGGTACAAACAGAAAGATCCGACGAAGAGCTATGAAGTTGCCCGGTACTTAACACCCGATAACACAACTTCGAAGATGCCTCAACACAGCGGCAGGAAAACGGTGTTCTACAGTGGATCCCTGCTGATCAGAAACGTCACCCAGGCTGACAGCGGATTCTACACCTTACTAACGTTTAACACAGAAATGGAAAGTGAACTAACACACGTGTATCTGGAAGTCCACA

>Pat_Ceacam11N1 (Peromyscus attwateri; Texas deermouse) WGS CABHPP010129382.1

CCTCCCTTTTAACCTGCTGGCTGCCTCCTACTGTTGTCCAGCTCACCATTGAATCAGTGCCACCCATTTCTGCTGAAGGGGATAATGTTCTTCTGCTTGTGCACAACCTTCCTGAGAATGTTCAAGCCTTTTCCTGGTACACAGGAGTTATGGTGCTCAAGAGCCGTGAAATTGCAAGATGTGCAATAGCTACCAATTCATGTGTGCTGGGGTCTGCACACAGTGGTAGAGAGACAGCATTCAATAATGGATCTCTGCTGATCGAGAATGTCACCAGGAAGGACTCAGGATACTACATCCTACAAACACTTAATACAAATTCAAGATCTGAAATAACACGTGCAGAATTTTTTGTACACA

>Pat_Ceacam15N (Peromyscus attwateri; Texas deermouse) CABHPP010001086.1

CCTCACTTTTAACCTGCTGGAACTCACCCACTGCGGCGCTAAGAACTACCAAAGAAATGAGGTTCTCTGCGGCTGAAGGGGGGAAGGTTCTTCTCTCTGTTCCTATTCAGGCAGAGAATCTCGTCTCCTTTCGCTGGTACAAAGGGAAAGAAGAAGACCAAGATTTTACAATTGCCCATTATGAAAAGGACACAGATTTACTTAAACTTGGGAATGTAACCAGCGGCAGGGAAGAGATATATAAGGACGGATCCATGATGCTCCAGGACGTCACCCAGGAAGACACAGGGATCTACACCCTAGAAACCTTTGGAACACATGATCATATTGAAATAACACATGTCTACCTCCAAGTGTACA

>Pat_Psg1N1 (Peromyscus attwateri; Texas deermouse) WGS CABHPP010089428.1

CCTCCATTTTAACCTGCTGGCACCTTTCCACCACTGACCATATCACCATTAAATCTGTCCCATCCCAAGTGGCCAATGGAGACAACGTCCTTCTTCTTGTCAACAATCTGCCAGAAGATCTTCTAACCTTTGCCTGGTTTAAAGGAGAGACAGGCATGAACGTTGGAATTGCAATATATGCACCAGACAGAGATTTAATCATGCAAGGGCCTGGATATAGCGGTAGAGAGATAGTGTACCGCAATGGATCCCTGCTGATCCAAAATATCAATGAGAAGGACACAGGACTCTACACCCTGCAAACCTTAAATGAACATGGAGATGTTCTGTCAATAACAACTATGCGCCTGCATGTGTACC

>Pat_Psg2N1 (Peromyscus attwateri; Texas deermouse) WGS CABHPP010110544.1

CCTCCCTCTTATCACGCTGGCGCCTTTCCACCACTGCCCAGTTTACCCTTGACTCTGTCCCACCTGAAGTCATCGAAGGAGAAAACGTCCTTTTCCTTGTCCACAATCTGCCAGAGAATCTTGCAGCCATAGTCTGGTCCAAAAGTGTGAAAAGTATGAAGCATGGAATTGTAACATATGCACTGAACAAAGATTCAAGTGTGCCAGGGCCTCTACACAGTGGTAGAGAGACACTGTACCGCAATGGATCCCTGCTGTTCAGAAATGTCACCAGGAAGGATACAGGACTTTATACCATAGAGCTCTTAGACAGACTTGGAGATAGTGTGTCAACAATAACTGCTTATCTTCGCGTGCACA

>Pat_Psg3N1 (Peromyscus attwateri; Texas deermouse) WGS CABHPP010131249.1

CCTCCCTTTTAACTTTCTGGCACCTGTCCACCACTGCCGATGTGACCATTGAATCACTGCCGCCCCTAGTGGCCGAAGGAGAAAATGTCCTTTTCCTTGTCCACCATCTGCCGGAAAATCTTACAGCCTTAGCCTGGTTCAAAGGACTAACAAATATGAAACAAGGAATTGCAATATATACACTGCACAGAAATTTAAGTGTGACAGGGCCTGCGCACAGTGGCAGAGAGACGATATATCACAATGGAGCTCTGTTGCTGGAAAATGTCACCCAGAAGGACACAGGATACTATACTCTACGAACCTATAACAGACATGCAAAAATCGTATCAACAACATCCATGTACCTCCAAGTGCACG

>Pat_Psg4N1 (Peromyscus attwateri; Texas deermouse) WGS CABHPP010139240.1

CCTCCCTGTTAACCTTCTGGCACCTGTCCACCACTGCCCACGTGACCATTGAATCACTGCCACCCCAAGTGGCTGAAGGAGAAAATGTCCTTTTCCTTGTCCATGGTCTGCCAAAGAATCTTATAGCCTTTGCCTGGTTGAAAGGGCTAGCAAATATGACACATGGAATTGCATGGTATACACTGGACAACAATTCACATGGGCCAGGGCCTGTAAACAGTGGTAGAGAGATAGTGTTTCGCAATGGATCCCTGCTGCTTCAAAATATCACTCAGAAGGACACAGGAACCTATACCCTACGAATCTATAATAGACGTGGAAAAATCATATCAACAACATCCATTTACCTCAATGTGCCTG

>Pat_Psg5N1 (Peromyscus attwateri; Texas deermouse) WGS CABHPP010123203.1

CCTCCCTCTTAACCTCCTGGCACCTGTCCACCACTGCCCATGTGACCATTGAGTCACTTCCACTCCAAGTGGCTGAAGGAGAAAACGTCATTTTCCTTGTCCATGATCTGCCAAAGAATCTTATAACCTTTGCCTGGTTTAAAGGGCTAACAAATATGACACAAAGAATTGCAATGTATACAATGGACAGCAATGTAAGTGGGTCAGGACATGTGCACAGTGATAGAGAGAGAATATATTGCAATGGATCCCTGTTGATCGAAAATGTCACCCAGAAGGACACAGGAATCTATACCCTACGAACTTATAACAGACATGGAAAAACTGTATCAACAACATCCATGTACTTTCACGTGTCCG

>Pat_Psg6N1 (Peromyscus attwateri; Texas deermouse) WGS CABHPP010138241.1

CCTCCCTGTTAACCTTCTGGCACCTGTCCACCGCTGCCCATGTGACCATTGAATCACTGCCACCCCAAGTGACTGAAGGAGAAAACGTCCTTTTCTTTGTCCGTAGGCTGCCAAAGAATCATATTGCCTTTGCCTGGTTCAAAAGGCTAACAAATATGACACGAGGAATTGCATGGTATACACTGGACAATAATTTACGTGGGCCAGGGCCTGGGCACAGTGGTAGAGAGATAGTGTATCACAATGGATCCCTGCTGCTCCAAAAGGTCACCCAGAAGGACACAGGAAGCTATATCTTACAAACCTATAATAGACGTAGAAAAATCATATCAACAACATCCATTTACATCCATGTGCATG

>Pat_Psg7N1 (Peromyscus attwateri; Texas deermouse) WGS CABHPP010118345.1

CCTCCCTTTTAACCTTCTGGCTCCTGTCCACCACTGCCACTGTAAACACTGAATCAATGCCACTCCTTGCGGCTGAAGGAGAAAACATCCTTTTCCCTGCTCAAGGTCTTCCAGAGAATATTATAGCCTTAGCCTGGTTCAAAGGACTAACAAAGATGACACAAGGAATTGCATTGTATGCGCTGCAGAGCAATTTAAGTTGCCCAGGTTCTGTGCACAGTGGTAGAGAGACAATATATCGCAATGGATCCCTGCTGCTGGAAAAGGTCACCCAGAATGACACAGGATTTTATACCCTACGAACCTTTAATAGATACCGAAAAATCATGTCAACAACATCCATTTACCTCCATGTGCATG

>Pat_Psg8N1 (Peromyscus attwateri; Texas deermouse) WGS CABHPP010133251.1

CCTCCCTTTTAACCTGCTGTCACCTGTCCGCCACTGCCCAAGTCACCATTGAATCAGTGCCACCCCAAGTGGTTGAAGGAGAAAACGTCCTCCTCCGTGTTCATAATCTACCAGAGAATCTTCTAGCCTTTATCTGGCACAAAGGGGCAAGGAATATGAGCCTTAGAATTGCACTGTATTCACTGGCCAAGGATGTAAGTGTGAAAGGTCCCGTACACAGCGGTAGAGAGACAGTGTACAGCAACGGATCCCTGCAGATCCACAATGTCACCCAGAAGGACACAGGATTCTATACCTTACGAACCATTAATAGAGGCATAGGAATTGTATCAATAACAACCAAGTACCTTCACGTGTACA

>Pat_Psg9N1 (Peromyscus attwateri; Texas deermouse) WGS CABHPP010000641.1

CCTCCCTGTTAGCCTTCTGGCACCTGTCCACCACTGCCCATGTGACCATTGAATCACTGCCACCCGAAGTGGCTGAAGGAGAAAACGTCCTTTTCCTTGTGCGTGATCTTCCAAAGAATGTTATCGCCTTTGCTTGGTTCAAAGGGCAAACAAATATGACACAAGGAATTGCATGGTATACATTGGACAACAATTCACGTGGGCCAGGGCCTGTGCACAGTGGTAGAGAGACAATATATCGCAATGGATCCCTGCTGCTCCAAAAGGTCACCCAGAAGGACACAGGACCCTATACCCTACAAACTTTTAATAGACAAAGAAAAATCATTTCAACAACATCCATTTACCTCCATGTGCATG

>Pat_Psg10N1 (Peromyscus attwateri; Texas deermouse) WGS CABHPP010142557.1

CCTTCCTCTTAACCTTCTGGCATCTGTCCACCATGGCCCATGTGACCATTGAGTCACTTCCACTCCAAGTGACTGAAGGAGAAAACGTCCTTTTCATTGTCCATGATCTCCCAGAGAATCTTACAACCTTTGCCTGGTTCAAAGGTCTAACAAATATGACACAAAGAATTGCAATATGTACAATGGACAACAATTTAAGTGGGCCAGGACATGTGCATAATGGTAGAGAGACAATATATTGCAATGGATCCCTGTTGCTCGAAAATGTCAACCAGAAGGACACAGAAAATTATACCCTACAAACCTATAATAGACATGGAAAAATCGTATCAACAACATCCATGTACCTCCATGTGTCTG

>Pat_Psg11N1 (Peromyscus attwateri; Texas deermouse) WGS CABHPP010000641.1

CTTGCCTTTTAACCTCCTGGCACCTGTCCACCACTGCCTATGTGACCACTAAAACAGTGCCACCCCAAGTGGCTGAAGGAGAAAACGTCCTTTTCCTGGTGCATGATCTTCCAGAGAATATTATAGGCTTTGCCTGGTTCAAAGAGCTAAGAAATATGAAAAAAGCAATTGCAGTATATGGACTGCACATCAATTTAAGTGCACCAGGGCCTGTGCACAGTGGTAGAGAAACAGTATATCGCAATGGATCCCTGCTGCTTGAAAAAGTCACCCGGAAGGACATAGGATTTTATACCCTGCGGACCTATGATAGAAATGTAAAAATCGTATCAACAACATCCACGTACCTCCATGTGGACA

>Pat_Psg12N1 (Peromyscus attwateri; Texas deermouse) WGS CABHPP010107409.1

CCTCCCTTTTAAGCTGCTGGTATCCACCCACCACTGCCCAAATCACCATTGAACTACTGCCGCCCAATGTGTTCGAAGGAGACAATGTCCTTCTACATGTCCGCAATCTGCCGGAGAATCTTTTAGCCTTTGCTTGGTTCAAAGGGGTGACACATTTGAAACGTGGAATTGCCATCTATTCACTGAAATACAATTTAAGTGTCACGGGGCCTGTTCACAGCGGAAGAGAGACAGTGTACAGCAATGGATCCCTGCTGCTCCAGCGTGTCACCCACAAGGACACAGGATTCTACACCCTACGAACCATAAACAGACAAGCAGAAATTGTATCAATGACATCCATGTACCTCCACGTGCACA

>Pat_Psg13N1 (Peromyscus attwateri; Texas deermouse) WGS CABHPP010107409.1 +TCTCCTTTTTAACCTGTTGGTACCTGACCACCACTGCCCAAGTCACCATTGAATTAGTGCCACCCCAAGTGGTTGAAGGAGAAAATGTCATTTTCCTTGTCCGTAATCTTCCAGAGAAACTTTTTGGCTTATCTTGGTTTAAAGAGGAGACAAATACGAAGTATAGAATTGCAAGCTATGAAATGGCCTACAGTCAGGATTTTCTGGGGGCAGCACACACTGGTAGAGAGACAGTGTACCCCAACGGATCCCTGCGGATTCAAAACGTCACCCAGAGTGACTCAGGAGTCTATATCCTACGAAGCAATAATAGAGTAAGAATTGTATCGTCAACATACATATATCTTCACGTGTACA

>Pat_Psg16N1 (Peromyscus attwateri; Texas deermouse) WGS CABHPP010124000.1

CCTCCCTTTTAACCTGTTGGTACCGGCCCACCACTACCCAAGTCACCATTGAATCAGTGCCTCCCCATGTGGTCGAAGGAGAAAGCAACCTTTTTCTTGTCCACAATCTTCCAGAGAATCTTTTAGCCATATCCTGGTTTAAAGAAGGAGCAAATATGGACCATAGAATTGTGACCTACACACTGAAATACAATATATCTCTGCCAGGAACAGCACACAGTGGTAGAGAGACAGTGTACCCAAATGGATCCCTGTGGATTCAAAATGTCACCCATAAGGACACAGGATTCTATATACTACAAACCATAAGTAGACAAGTAAAAACTGTATCAAGAACATACATACACCTTCATGTGTACA

>Pat_Psg17N1 (Peromyscus attwateri; Texas deermouse) WGS CABHPP010146717.1

CCTCCCTTTTAACCTGCTGGCACCTGTCCACCACTGCCCAAGTTACCATTGAATCAGTGCCACCCCAAGTGGTTGAAGGAGAAAATGTCCTTCTACGTGCCAACAATCTGCCAGAGAATCTTCTAGCTTTTTCCTGGTACAAGGAAGTGAGAAATAGGAACCTCAGAATTGCACTATTTGCACTGAACACTAATCAAAGTGTGATGGGGCCTGAACAAAGTGACAGAGAAACAGTGTACAGCAATGGATCCCTGTGGCTGAAAAATGTGACCAAGAAGGACACAGGATTCTATACCCTACAAACAGTAAATAGAGGTGGAAAAATTGTATCTACAACAACCATGTACTTCCATGTGTACA

>Pat_Psg18N1 (Peromyscus attwateri; Texas deermouse) WGS CABHPP010142557.1

CCTCCATTTTAATCTACTGGCACCTTTCAACCACTGATGATTTTACCATGGAACCTGTCCCACCCCATGTGGCCAATGGAGACAATGTACTTCTTCTTGTCCACAATCTGCCAGAGAATCTTATAGTCTTTGCCTGGATCAAAGGGGAGATAAGCATGAATCATACAATTGCAATATATATACCAAACAAGAAGTTAAGTGTGCCAGGGCGTTTATATAGTGGTAGAGAGACAGTGTATGGCAATGGATCCCTGCTCCTCCAAAATGTCAATGAGAAGGACACAGGAATTTATACCCTACAAACCTTTAATAGACGCACAGATACTGTGTCGCAAACATCCATGTACCTCTATGTTCACA

>Pat_Psg19N1 (Peromyscus attwateri; Texas deermouse) WGS CABHPP010048122.1

CCTCCCTTTTAATCTGCTGTCATTTAGCCACCACTGCCAAAGTCACCATTGAATCAGTGCCACTCAATGTGTTCGAAGGAGACAATGTCCTTCTACATGTCCACAATCTGCCAGAGAGTCTTCTAGCCTTTGCTTGGTTCAAAAGGCTGACAAAAACAAAGCACAGAATTGCACTCTATGCACTGAACACCAATTTAATTGTGCCGGGGCCTGTACACAGTGGTAGAGAGACAGTGTACCGCAATGGATCCCTTTGGATTCAGAATGTCACCCATAAGGACACAGGATTCTACACCCTACAAACCATAAATAGACATGGAAGAACTGTATCAATAACAGCCATGTACCTCCATGTGTACA

>Pat_Psg21N1 (Peromyscus attwateri; Texas deermouse) WGS CABHPP010146580.1

CCTCCTTTTTAACCTTTTGGCACCTGTCTACAACTGACCATGTGACCATTGAATCACTGCCACCCCAAGTGGCTGAAGGAGAAAACATCCTTTTCCTTGTCCAGAATCTGCCAAAGAATCTTATAGCCTTTGCTTGGTTCAAAGGGCTAAGAAGTATGAAACGAGTAATTGCAATGTATACACTGCACAACAATTTAAGTGGTCCAGGGCCTGTGCACAGCGGTAGAGAGACAATATATCACAATGGATCCCTGCTGCTCGAAAAGGTCACCCAGAAGGACACAGGATACTATACGCTAAGAACATATAACAGACGGGGAAAAATCGTATCAACAACATCCATGTACCTCCACGTGCATG

>Pat_Psg23N1 (Peromyscus attwateri; Texas deermouse) WGS CABHPP010134784.1

CCTCTCTTTTAACCTGTTGGCACCTACCCACCACTGTCCAAGTCATTATTGAATTAGTGCCACCTGATGTGGTTGAAGGAGAAAATGTCCTTCTCCTTGTCCGCAATCTGCCAGAGAATCTTGAAGCCTTTGTCTGGTACAAAGGGGTGACAAATATGAACCTCGGAATTGTACTTTATTCGCTGACCACTAATTTAAGCGTGGCAGGGCCTGAATACAGTGGTAGAGAGACAGTTTACAGAAATGGATCCCTGCGTCTCCAAGATGTCACCCAGAAGGACACAGGATTCTATACCCTACGATCCATAAGCAGACATAAGGAAATCATATCAACAACATCCATATACCTCCACGTGTACT

>Pat_Psg24N1 (Peromyscus attwateri; Texas deermouse) WGS CABHPP010098702.1

CCTCCATTTTAACCTGCTGGCACCTTTCCACCACTGATGGTTTTGCTGTGGAAACTGTCCCACCCCATGTGGCCAACGGAGACAACATCCTTTTCCTTGTCCGCAATCTGCCAAAGAATCTTCTATCTTTCGCCTGGATCAAAGGGGGAACGAGCATGAATGATGCAATCATAGTATATATACCAAACAAAAATTTAAGTGTGCCAGGGCGTTTTCACAGTGGTAGAGAGACAGTGTATGACAATGGATCCCTGCTGATCCAAAATGTCAACCAGAAGGACACAGGAATCTATTCTCTACGAGCTTTTCATAGACGCACAGATTCTGTGTCACAAATAGGCACATACCTCTACGTGCACA

>Pat_Psg25N1 (Peromyscus attwateri; Texas deermouse) WGS CABHPP010134784.1

CCTCCCTTTTAACCTGCTGGCACCTGTCCACCACTGCCCAAGTTACTATTGAATCAGTGCCGCCCCAAGTGGTTGAAGGAGAAAATGTCCTTCTACGTGCCAACAATCTGCCAGAGAATCTTCTAGCTTTCTCCTGGTACAAGGAGGTGAGGAATATGAACCTCAGAATTGCACTATTTGCACTGAACACTAATGTAAGTGTGATGGGGCCTGAACAAAGGGACAGAGAAACAGTGTACAGCAATGGATCCCTGTGGCTGAAAAATGTCACCAAGAAGGACACAGGATTCTATACCCTACGAACAGTAAATAGACACGGAAAAACTGTATCTACAACAACCATGTACTTCCATGTCTACA

>Paz_Ceacam9N (Peromyscus aztecus; Aztec mouse) WGS CABHPQ010143705.1

CCTCCCTCTTGACCTGCTGGAATGCACCTACTACTGCCGAGCTCACTATTGAATTAGTGCCTCCCATGGTGGCTGAAGGTGGAAACTCCGTTCTATTTGTCCACAAAATGCCGTTGAACGTCCAAGCATTTTACTGGTACAAACAGAAAGATCCGACGAAGAGCTATGAAGTTGCCCGGTACTTAACACCCGATAACACAACTTCGAAGATGCCTCAACAGAGCGGCAGGAAAACGGTGTTCTACAGTGGATCCCTGCTGATCAGAAACGTCACCCAGGCTGACAGCGGATTCTACACCTTACTAACATTTAACACAGAAATGGAAAGTGAACTAACACACGTGTATCTGGAAGTCCACA

>Paz_Ceacam11N1 (Peromyscus aztecus; Aztec mouse) WGS CABHPQ010001443.1

CCTCCCTTTTAACCTGCTGGCTGCCTCCTGCTGTTGCCCAGCTCACCATTGAATCAGTGCCACCCATTTCTGCTGAAGGGGATAATGTTCTTCTGCTTGTGCACAACCTTCCTGAGAATGTTCAAGCCTTTTCCTGGTACACAGGAGTTATGGTGCTCAAGAGCCGTGAAATTGCAAGATGTGCAATAGCTACCAATTCATGTGTGCTGGGGTCTGCACACAGTGGTAGAGAGACAACATTCAATAATGGATCTCTGCTGATCGAGAATGTCACCAGGAAGGACTCAGGATACTACATCCTACAAACACTTAATACAAATTCGAGATCTGAAATAACACGTGCAGAATTTTTTGTACACA

>Paz_Ceacam15N (Peromyscus aztecus; Aztec mouse) WGS CABHPQ010118331.1

CCTCACTTTTAACCTGCTGGAACTCACCCACTGCGGCGCTAAGAACTACCAAAGAAATGAGGTTCTCTGCGGCTGAAGGGGGGAAGGTTCTTCTCTCTGTTCCTATTCAGGCAGAGAATCTTCTCTCCTTTCGCTGGTACAAAGGGAAAGAAGAAGACCAAGATTTTACAATTGCCCATTATGAAAAGGGCACAGATTTACTTAAACTTGGGAATGCAACCAGCGGCAGGGAAGAGATATATAAGGACGGATCCATGATGCTCCAGGACGTCACCCAGGAAGACACAGGGATCTACACCCTAGAAATCTTTGGAACACATGATCATATTGAAATAACACATGTCTACCTCCAAGTGTACA

>Paz_Psg1N1 (Peromyscus aztecus; Aztec mouse) WGS CABHPQ010162957.1

CCTCCATTTTAACCTGCTGGCACCTTTCCACCACCGACCATATCACCATTAAATCTGTCCCATCCCAAGTGGCCAATGGAGACAACGTTCTTCTTCTTGTCAACAATCTGCCAGAAGATCTTCTAACCTTTGCCTGGTTTAAAGGAGAGACAGGCATGAATGTTGGAATTGCGATATATGCACCAGACAGAGATTTAATTATGCAAGGGCCTGGATATAGCGGTAGAGAGATAGTGTACCGCAATGGATCCCTGCTGATCCAAAATATCAATGAGAAGGACACAGGACTCTACACCCTGCAAACCTTAAATGAACATGGAGATGTTCTGTCAATAACAACTATGCGCCTGCATGTGTACC

>Paz_Psg3N1 (Peromyscus aztecus; Aztec mouse) WGS CABHPQ010163527.1

CCTCCCTTTTAACTTTCTGGCACCTGTCCACCACTGCCAATGTGACCATTGAATCACTGCCGCCCCTAGTGGCCGAAGGAGAAAATGTCCTTTTCCTTGTCCACCATCAGCTGGAAAATCTTACGGCTTTAGCCTGGTTCAAAGGACTAACAAATATGAAACAAGGAATTGCAATATATACACTGCACAGAAATTTAAGTGTGACAGGGCCTGCGCACAGTGGCAGAGAGACGATATATCACAATGGATCTCTGTTGCTGGAAAATGTCACCCAGAAGGACACGGGATACTATACTTTACGAACCTATAACAGACGTGCAAAAATCGTATCAACAACATCCATGTACCTCCAAGTGCACG

>Paz_Psg4N1 (Peromyscus aztecus; Aztec mouse) WGS CABHPQ010163084.1

GCTCCCTGTTAACCTTCTGGCACCTGTCCACCGCTGCCCATGTGACCATTGAATCACTGCCACCCCAAGTGGCTGAAGGAGAAAATGTCCTTTTCCTTGTCCATGGTCTGCCAAAGAATTTTATAGCCTTTGCCTGGTTGAAAGGGCTAACAAATATGACACATGGAATTGCATGGTATACACTGGACAACAATTCACATGGGCCAGGGCCTGTAAACAGTGGTAGAGAGATAGTGTTTCGCAATGGATCCCTGCTGCTTCAAAATATCACCCAGAAGGACACAGGAACCTATACCCTACGAATCTATAATAGACGTGGAAAAATCATATCAACAACATCCATTTACCTCCATGTGCCTG

>Paz_Psg5N1 (Peromyscus aztecus; Aztec mouse) WGS CABHPQ010163073.1

CCTCCCTCTTAACCTCCTGGCACCTGTCCACCACTGCCCATGTGACCATTGAGTCACTTCCACTCCAAGTGGCTGAAGGAGAAAACGTCATTTTCCTTGTCCATGATCTGCCAAAGAATCTTATAACCTTTGCCTGGTTTAAAGGACTAACAAATATGACACAAAGAATTGCAATGTATACAATGGAGAGCAATGTAAGTGGGTCAGGACATGTGCACAGTGATAGAGAAAGAATATATTGCAATGGATCCCTGTTGATCGAAAATGTCACCCAGAAGGACACAGGAATCTATACCCTACGAACCTATAATAGACATGGAAAAACTGTATCAACAACATCCATGTACTTTCACGTGTCCG

>Paz_Psg6N1 (Peromyscus aztecus; Aztec mouse) WGS CABHPQ010164757.1

CCTCCCTGTTAACCTTCTGGCACCTGTCCACCGCTGCCCATGTGACCATTGAATCACTGCCACCCCAAGTGACTGAAGGAGAAAACGTCCTTTTCTTTGTCCGTCAGCTGCCAAAGAATCATATCGCCTTTGCCTGGTTCAAAAGGCAAACAAATATGACACGAGGAATTGCATGGTATACACTGGACAATAATTTACGTGGGCCAGGGCCTGGGCACAGTGGTAGAGAGACAGTGTATCACAATGGATCCCTGCTGCTCCAAAAGGTCACCCAGAAGGACACAGGAAGCTATATCCTACAAACCTATAATAGACGTAGAAAAATCATTTCAACAACGTCCATTTACCTCCATGTGCATG

>Paz_Psg7N1 (Peromyscus aztecus; Aztec mouse) WGS CABHPQ010131622.1

CCTCCCTTTTAACCTTCTGGCTCCTGTCCACCACTGCCACTGTAAACAATGAATCAATGCCACTCCTTGTGGCTGAAGGAGAAAACATCCTTTTCCCTGCCCAAGATCTTCCAGAGAATATTATAGCCTTAGCCTGGTTCAAAGGACTAACAAAGATGACACAAGGAATTGCACTGTATGCCCTGCACAGCAATTTAAGTTGCCCAGGTTCTGTGCACAGTGGTAGAGAGACAATATATCGCAATGGATCCCTGCTGCTGGAAAAGGTCACCCAGAATGACACAGGGTTTTATACCCTACGAACCTTTAATAGATACCGAAAAATCATGTCAACAACATCCATTTACCTCCATGTGCATG

>Paz_Psg8N1 (Peromyscus aztecus; Aztec mouse) WGS CABHPQ010157143.1

CCTCCCTTTTAACCTGTTGTCACCTGTCCACCACTGCCCAAGTCACCATTGAAGTAGTGCCACCCCAAGTGGTTGAAGGAGAAAACGTCCTCCTCCGTGTTCATAATCTACCAGAGAATCTTCTAGCCTTTATCTGGCACAAAGGGGCAAGGAATATGAGCCTTAGAATTGCACTGTATTCACTGGCCAAGGATGTAAGTGTGAAAGGTCCTGTACACAGTGGTAGAGAGACAGTGTACAGCAACGGATCCCTGCAGATCCACAATGTCACCCAGAAGGACACAGGATTCTATACCTTACGAACCATTAATAGAGGCATAGGAATTGTATCAATAACAACCAAGTACCTTCACGTGCACA

>Paz_Psg9N1 (Peromyscus aztecus; Aztec mouse) WGS CABHPQ010164757.1

CCTCCCTGTTAGCCTTCTGGCACCTGTCCACCACTGCCCATGTGACCATTGAATCACTGCCACCTGAAGTGGCTGAAGGAGAAAACGTCCTTTTCCTTGTGCATGAACTTCCAAAGAATGTTATCGCCTTTGCTTGGTTCAAAGGGCAAACAAATATGACACAAGGAATTGCATGGTATACACTGGACAACAATTCACGTGGCCCAGGGCCTGTGCACAGTGGTAGAGAGACAGTGTATCGCAATGGATCCCTGCTGCTCCAAAAGGTCACCCAGAAGGACACAGGACCCTATACCCTACAAACTTTTAATAGACACAGAAAAATCATTTCAACAGCATCCATTTACCTCCATGTGCACG

>Paz_Psg10N1 (Peromyscus aztecus; Aztec mouse) WGS CABHPQ010154498.1

CCTTCCTCTTAACCTTCTGGCATCTGTCTACCGTGGCCCATGTGACCATTGAGTCACTTCCACTCCAAGTGACTGAAGGAGAAAACGTCCTTTTCCTTGTCCATGATCTCCCAGAGAATCTTACAACCTTTGCCTGGTTCAAAGGTCTAACAAATATGACACAAAGAATTGCAATGTGTACAATGGACAACAATTTAAGTGGGCCAGGACATGTGCACAGCGGTAGAGAGACAATATATTGCAATGGATCCCTGTTGCTCGAAAATGTCAACCAGAAGGACACAGAAAATTATACCCTACAAACCTATAATAGACATGGAAAAATCGTATCAACAACATCCATGTACCTCCATGTGTCTG

>Paz_Psg11N1 (Peromyscus aztecus; Aztec mouse) WGS CABHPQ010180814.1

CCTGCCTTTTAACCTCCTGGCACCTGTCCACCACTGCCTATGTGACCACTAAAACAGTGCCACCCCAAGTGACTGAAGGAGAAAACGTCCTTTTTCTGGTGCATGAACTTCCAGAGAATATTATAGGCTTTGCCTGGTTCAAAGAGCTAAGAAATATGAAAAAAGCAATTGCAGTATATGGACTGCACATCAATTTAAGTGCACCAGGGCCTGTGCACAGTGGTAGAGAAACAGTATATCGCAATGGATCCCTGCTGCTTGAAAAAGTCACCCGGAAGGACATAGGATTTTATACCCTGCGGACCTATGATAGAAATGTAAAAATCGTATCAACAACATCCACGTACCTCCATGTGGACA

>Paz_Psg12N1 (Peromyscus aztecus; Aztec mouse) WGS CABHPQ010128958.1

CCTCCCTTTTAAGCTGCTGGTATCCACCCACCACTGCCCAAATCACCATTGAACTACTGCCGCCCAATGTGTTCGAAGGAGACAATGTCCTTCTACATGTCCACAATCTGCCGGAGAATCTTCTAGCCTTTGCTTGGTTCAAAGGGGTGACAAATTTGAAACGTGGAATTGCCATCTATTCACTGAAATACAATTTAAGTGTCACGGGGCCTGTACACAGTGAAAGAGAGACAGTATACAGCAATGGATCCCTGCTGCTCCAGCGTGTCACCCACAAGGACACAGGATTCTACACCCTACGAACCATAAATAGACAAGCAGAAATTGTATCAATGACATCCGTGTACCTCCACGTGCACA

>Paz_Psg13N1 (Peromyscus aztecus; Aztec mouse) WGS CABHPQ010128958.1

TCTCCTTTTTAACCTGCTGGCACCTGACCACCACTGCCCAAGTCACCATTGAATTAGTGCCACCCCAAGTGGTTGAAGGAGAAAATGTCATTTTCCTTGTCCGTAATCTTCCGGAGAAGATTTTTGGCTTATCCTGGTTTAAAGAGGAGACAAATACGAAGTATAGAATTGCAAGCTATGAAATGCCCTACAATCAGGATTTTCTGGGGGCAGCACACACTGGTAGAGAGACAGTGTACCCCAACGGATCCCTGTGGATACAAAATGTCACCCAGAGTGACTCAGGAGTCTATATCCTACGAAGCAATAATAGAGTAAGAATTGTATCGTCAACATACATATACCTTCACGTGTACA

>Paz_Psg14N1 (Peromyscus aztecus; Aztec mouse) WGS CABHPQ010071435.1

TCTCCCTTTTAACCTGTTGGTACTGGCCCACTATTGCCCAAGTCACCATTGAATCAGTGCCTCTCCATGTGGTCGAAGGAGAAAGCAACCTTTTTCTTGTCCACAATCTGCCAGAGAATCTTTTAACCATATCATGGTTTAAAGAAGAAGAAAATGTGGACCATAAAATTGCGACCTACACACTGAAATACAATATTGCTGTGCCGGGGGCAGCACACAGTGGTAGAGAGACAGTGTACCCAAATGGATCCCTATGGATTCAAAATGTCACCCATAAGGACACAGGATTCTATATACTACGAACCAGAAGTAGACAAGTAAAAATTATATCAAAAATATATACATACCTTCATGTATACA

>Paz_Psg15N1 (Peromyscus aztecus; Aztec mouse) WGS CABHPQ010071435.1

TCTCCCTTTTAACCTGTTGGTACTGGCCCACTATTGCCCAAGTCACCATTGAATCAGTGCCTCTCCATGTGGTCGAAGGAGAAAGCAACCTTTTTCTTGTCCACAATCTGCCAGAGAATCTTTTAACCATATCATGGTTTAAAGAAGAAGAAAATGTGGACCATAAAATTGCGACCTACACACTGAAATACAATATTGCTGTGCCGGGGGCAGCACACAGTGGTAGAGAGACAGTGTACCCAAATGGATCCCTATGGATTCAAAATGTCACCCATAAGGACACAGGATTCTATATACTACGAACCAGAAGTAGACAAGTAAAAATTATATCAAAAATATATACATACCTTCATGTATACA

>Paz_Psg16N1 (Peromyscus aztecus; Aztec mouse) WGS CABHPQ010150973.1

CCTCCCTTTTAATCTGTTGGTACCGGGCCACCACTGCCCAAGTCACCATTGAATCAGTGCCTCCCCATGTGGTCGAAGGAGAAAGCAACCTTTTTCTTGTCCACAATCTTCCAGAGAATCTTTTAGCCATATCCTGGTTTAAAGAGGGAGCAAATATGGACCACAGAATTGCGACCTACACACTGAAATACAATGTATCTCTGCCAGGAACAGCTCACAGCGGTAGAGAGACAGTGTACCCAAATGGATCCCTGTGGATTCAAAATGTCACCCATAAGGACACAGGATTCTATATACTACAAACCATAAGTAGACAAGTAAAAATTGTATCAAGAACATACATACACCTTCATGTGTACA

>Paz_Psg17N1 (Peromyscus aztecus; Aztec mouse) WGS CABHPQ010180811.1

CCTCCCTTTTAACCTGCTGGCATCTGTCCACCACTGCCCAAGTTACCGTTGAATCAGTGCCACCCCAAGTGGTTGAAGGAGAAAATGTCCTTCTACGTGCCAACAATCTGCCAGAGAACCTTCTAGCTTTTTCCTGGTACAAGGAGGTGAGAAATAGGAACGTCAGAATTGCACTATTTGCACTGAACACTAATCAAAGTGTGATGGGGCCTGAACAAAGTGGCAGAGAAACAGTGTACAGCAATGGATCCCTGTGGCTGAAAAATGTGACCAAGAAGGACACAGGATTCTATATCCTACAAACAGTAAATAGAGGTGGAAAAATTGTATCTACAACAACCATGTACTTCCATGTGTACA

>Paz_Psg18N1 (Peromyscus aztecus; Aztec mouse) WGS CABHPQ010029613.1

CCTCCATTTTAATCTGCTGGCACCTTTCAACCACTGATGATTTTACCATGGAACCTGTCCCACCCCATGTGGCCAATGGAGACAATGTACTTCTTCTTGTCCACAATCTGCCAGAGAATCTTATAGCCTTTGCCTGGATCAAAGGGGAGATAAGCATGAATCATACAATTGCAATATATATACCAAACAAAAAGTTAAGTGTGCCAGGGCGTTTACAGACTGGTAGAGAGATAGTGTATGGCAATGGATCCCTGCTCCTCCAGAATGTCAATGAGAAGGACACAGGAGTTTATACCCTACAAACCTTTAATAGACGCACAGATACTGTGTCGCAAACATCCATGTACCTCTATGTTCACA

>Paz_Psg19N1 (Peromyscus aztecus; Aztec mouse) WGS CABHPQ010142170.1

CTTCCCTTTTAATCTGCTGTCATTTAGCCACCACTGCCAAAGTCACCATTGAATCAGTGCCACTCAATGTGTTCGAAGGAGACAATGTCCTTCTACATGTCCACAATCTGCCAGAGAGTCTTCTAGCCTTTGCTTGGTTCAAAAGGCTGACAAAAACGAAGCACAGAATTGCACTCTATGCACTGAACACCAATTTAATTGTGCCGGGGCCTGTACACAGTGGTAGAGAGACAGTGTACCGCAATGGATCACTGTGGATTCAGAATGTCACCCATAAGGACACAGGATTCTACACCATACAGACCATAAATAGACATGGAAGAACTGTATCAACAACAGCCATGTACCTCCATGTATACA

>Paz_Psg21N1 (Peromyscus aztecus; Aztec mouse) WGS CABHPQ010157143.1

CCTCCTTTTTAACCTTTTGGCACCTGTCTACAACTGACCATGTGACCATTGAATCACTGCCACCCCAAGTGGCTGAAGGAGAAAACATCCTTTTCCTTGTCCAGAATCTGCCAAAGAATCTTATAGCCTTTGCTTGGTTCAAAGGGCTAAGAAGTATGAAACAAGGAATTGCAATGTATACACTGCACAACAATTTAAGTGGTCCAGGGCCTGTGCACAGCGGTAGAGAGACAATATATCACAATGGATCCCTGCTGCTCGAAAAGGTCACCCAGAAGGACACAGGATACTATATGCTAAGAACATATAACAGACGGGGAAAAATCATATCAACAACATCCATGTACCTCCACGTGCATG

>Paz_Psg22N1 (Peromyscus aztecus; Aztec mouse) WGS CABHPQ010157143.1

CCTCCCTTTTAACCTGTTGTCACCTGTCCACCACTGCCCAAGTCACCATTGAAGTAGTGCCACCCCAAGTGGTTGAAGGAGAAAACGTCCTCCTCCGTGTTCATAATCTACCAGAGAATCTTCTAGCCTTTATCTGGCACAAAGGGGCAAGGAATATGAGCCTTAGAATTGCACTGTATTCACTGGCCAAGGATGTAAGTGTGAAAGGTCCTGTACACAGTGGTAGAGAGACAGTGTACAGCAACGGATCCCTGCAGATCCACAATGTCACCCAGAAGGACACAGGATTCTATACCTTACGAACCATTAATAGAGGCATAGGAATTGTATCAATAACAACCAAGTACCTTCACGTGCACA

>Paz_Psg23N1 (Peromyscus aztecus; Aztec mouse) WGS CABHPQ010155442.1

CCTCTCTTTTAACCTGTTGGCACCTACCCACCACTGTCCAAGTCATTACTGAATTAGTGCCACCTGATGTGGTTGAAGGAGAAAATGTCCTTCTCCTTGTCCGCAATCTGCCAGAGAATCTTGAAGCCTTTGTCTGGTACAAAGGGGTGACAAATATGAACCTGGGAATTGTACTTTATTCGCTGACCACTAATTTAAGCGTGGCAGGGCCTGAATACAGTGGTAGAGAGACAGTTTACAGAAATGGATCCCTGCATCTCCAAGATGTCACCCAGAAGGACACAGGATTCTATACCCTACTATCCATAAGCAGACATAAAGAAATCATATCAACAACATCCATATACCTCCACGTGTACT

>Paz_Psg24N1 (Peromyscus aztecus; Aztec mouse) WGS CABHPQ010150973.1

CCTCCATTTTAACCTGCTGGCACCTTTCCACCACTGATGGTTTTTCTGTGGAAACTGTCCCACCCCATGTGGCCAACGGAGACAACATCCTTTTCCTTGTCCACAATCTGCCAAAGAATCTTCTATCTTTCGCCTGGATCAAAGGAGGAACAAGCATGAATGATGCAATCATAGTATATATACCAAACAAAAATTTAAGTGTGCCAGGGCGTTTTCACAGTGGTAGAGAGACAGTGTATGGCAATGGATCCCTGCTGATCCAAAATGTCAACCAGAAGGACACAGGAGTCTATTCTCTACGAGCTTTTCATAGACGCACAGATACTGTGTCACAAATAGGCACATACCTCTACGTGAACA

>Pca_Ceacam9N (Peromyscus californicus insignis; California mouse) WGS VALE03000004.1

CCTCCCTCTTGACCTGCTGGAATGCACCTACTACTGCCCAGCTCACTATTGAATTAGTGCCTCCCATGGTGGCTGAAGGCGGAAACTCCGTTCTGTTTGTGCACAAAATGCCGCTGAACGTCCAGGCATTTTACTGGTACAAACAGAAAGATCCGACGAAGAGCTATGAAGTTGCCCGGTACTTAACACCCGATAACACAACTTCGAAGATGCCTCAACACAGCGGCAGGAAAACGGTGTTCTACAGTGGATCCCTGCTGATCAGAAACGTCACCCAGGCTGACAGCGGATTCTACACCTTACTAACATTTAACACAGAAATGGAAAGCGAACTAACACACGTGTATCTGGAAGTCCATA

>Pca_Ceacam11N1 (Peromyscus californicus insignis; California mouse) WGS VALE02034109.1

CCTCCCTTTTAACCTGCTGGCTGCCTCCTACTGTTGCCCAGCTCACCATTGAATCAGTGCCACCCATTTCTGTTGAAGGGGATAATGTTCTTCTGCTTGTGCACAACCTTCCTGAGAATGTTCAGGCCTTTTCCTGGTACACAGGAGTTATGGTGCTCAAGAGCCATGAAATTGCAAGATGTGCAATAGCTACCAATTCATGTGTGCTGGGGCTTGCACACAGTGGTAGAGAGACAGCATTCAATAATGGATCTCTGCTGATTAAGAATGTCACCGGGAAGGACTCAGGATACTACATCCTACAAACACTTAATACAAATTCGAGATCTGAAATAACATGTGCGGAATTTTTTGTACACA

>Pca_Ceacam15N (Peromyscus californicus insignis; California mouse) WGS VALE02034109.1

CCTCACTTTTAACCTGCTGGAACTCACCCGCTGCGGCGCTAAGATCTACCAAAGAAATGCGGTTTTCTGCGGCTGAGGGGGGGAGGGTTCTTCTCTCTGTTCCTGTTCAGGCAGAGAACCTTCTCTCCTTTCACTGGTACAAAGGAAAAGAAGAAGACCAGGGTTTTACAATTGCCCGTTATGAGAAGGACACAGATTTACTTAAACTTGGGAATGCAACCAGCGGCAGGGAAGAGGTATATAAGGACGGATCCATGATGCTCCAGGACGTCACCCAGGAAGACACGGGGATCTACACCCTAGAAACCTTTGGAGCACATGATCATATTGAAATAACACATTTCTACCTCCAAGTGTACA

>Pca_Psg1N1 (Peromyscus californicus insignis; California mouse) WGS VALE02034109.1

CCTCCATTTTAACCTGCTGGCACCTTTCCGCCACTGACCATATCACCATTAAATCTGTCCCATCCCAAGTGGCCAATGGAGACAACGTCCTTCTTCTTGTCAACAAACTGCCAGAAGATCTTCTAAACTTTGCCTGGTTTAAAGGCGAGTCAGGCATGAACCTTGGAATTGCAATATATGCACCAGACAGAGATTTAATCATGCAAGGGCCTGGATATAGCGGTAGAGAGACAGTGTACCGCAATGGATCCCTGCTGATCCAAAATGTCAATGAGAAGGACACAGGACTCTACACCCTGCAAACCTTAAATGAACATGGAGATGTTCTGTCAGTAACAACTATGCGCCTGCATGTGTACC

>Pca_Psg3N1 (Peromyscus californicus insignis; California mouse) WGS VALE02034109.1

CCTCCCTTTTAACTTTCTGGCACCTGTCCACCACTGCCGATGTGACCATTGAATCACTGCCGCCCCTAGTGGCCGAAGGAGAAAATGTCCTTTTCCTTGTCCACCATCTGCCAGAAAATCTTACGGCCTTAGCCTGGTTCAAAGGACTAACAAATATGAAACAAGGAATTGCAATGTATACACTACACAACAATTTAAGTGTGACAGGGCCTGTGCACAGCGGTAGAGAGACAGTATATCACAATGGATCTCTGTTGCTGGAAAACGTCACCCAGAAGGACACAGGATACTATACTCTACGAACCTATAACAGACGTGCAAAAATCCTATCAACAACATCCATGTACCTCCAAGTGCACG

>Pca_Psg4N1 (Peromyscus californicus insignis; California mouse) WGS VALE02034097.1

CCTCCCTATTAACCTTCTGGCACCTGTCCACCACTGCCCATGTGACCATTGAATCACTGCCACCCCACGTGACTGAAGGAGAAAACGTCCTTTTCCTTGTCCATGGTCTGCCAAAGAATCTTATAGCCTTTGCCTGGTTCAAAGGGCAAATAAATATGACACATGGAATTGCATGGTATACACTGGACAACAATTTACATGGGCCAGGGCCTGTAAACAGTGGTAGAGAGACAGTGTATCACAATGGATCTCTGCTGCTTCAAAATATCACCCAGAAGGACACAGGAACCTATACCCTACGAATCTATAATAGACGTGGAAAAATCATATCAACAACATCCATTTACCTCCATGTGCCTG

>Pca_Psg5N1 (Peromyscus californicus insignis; California mouse) WGS VALE02034109.1

CCTCCCTCTTAACCTCCTGGCACCTGTCCACCACTGCCCATGTGACCATTGAGTCACTTCCACTCCAAGTGGCTGAAGGAGAAAACGTCCTTTTCCTTGTCCATGATCTGCCAGAGAATCTTATCGCCTTTGCCTGGTTTAAAGGGCTGGCAAAGACGACACAAAGAATTGCAATGTATACAATGGACAGCAATGTAAGTGGGCCAGGACATGTGCACAGTGATAGAGAGACAATATATCATAATGGATCCCTGTTGATTGAAAATGTCACCCAGAAGGACTCAGAAATCTATACCCTACGAACCTATAATAGACATGGAAAAACTGTATCAACAACATCCATGTACTTTCACGTGTCTG

>Pca_Psg6N1 (Peromyscus californicus insignis; California mouse) WGS VALE02034109.1

CCTCCCTGTTAACCTTCTGGCACCTGTCCACCACTGCCCATGTGACCATTGAATCACTGCCACCCCACGTGACTGAAGGAGAAAATGTCCTTCTCTTTGTCCATCATCTGCCGAAGAATCTTATTGCCTTTGCCTGGTTCAAAAGGCTAACAAATATGACACGAGGAATTGCATGGTATACATTGGACAATAATTTGCGTGGGCCAGGGCCTGGGCACAGTGGTAGAGAGATAGTGTATCACAATGGATCCCTGCTGCTCCAAAATGTCATCCAGAAGGACACAGGAAGCTATATCCTACAAACCTATAATAAACATAGAAAAATCATATCAACAACATCCATTTACCTCCATGTGCATG

>Pca_Psg7N1 (Peromyscus californicus insignis; California mouse) WGS VALE02034109.1

CCTCCCTTTTAATCTTCTGGCTCCTGTCCACCACTGCCACTGTAAACACTGAATCAATGCCACTCCTAGTGGCTGAAGGAGAAAACATCCTTTTCCCTGTCCAAGATCTTCCAGAGAATATTATAGCCGTAGCCTGGTTCAAAGGACTAACAAAGATGACACAAGGAATTGCTTTGTACGCACTGCACAGCGATATAAGTTGCCCAGGTTCTGTGCACAGTGGTAGGGAGACAATATATCGCAATGGATCCCTGCTGCTGGAAAAGGTCACCCAGAATGACACAGGATTTTATACCCAACGAACCTTTAATAAACACAGAAAAATCATGTCAACAGCATCCATTTACCTCCATGTGCATG

>Pca_Psg8N1 (Peromyscus californicus insignis; California mouse) WGS VALE02034109.1

CCTCCCTTTTAACCTGCTGTCACCTGTCCACCACTGCCCAAGTCACCATTGAATCAGTGCCACCCCAAGTTGTTGAAGGAGAAAACGTCCTCCTCCGTGTTCATAATCTACCAGAGAATCTTCTAGCCTTTATCTGGCACAAAGGGGCAAGGGATATGAGCCTTAGAATTGCACTGTATTCACTGGCCAAGGATGTAAGTGTGAAAGGTCCCGTACACAGTGGTAGAGAGACAGTGTTCAGCAACGGATCCCTGCAGATCCACAATGTCACCCAGAAGGACACAGGATTCTATACCTTACGAACCGTTAATAGACGCATAGGAATTGTATCAATAACAACCAAGTACCTCCACGTGCACA

>Pca_Psg9N1 (Peromyscus californicus insignis; California mouse) WGS VALE02034109.1

CCTCCCTGTTAACCTTCTGGCACCTGTCTACCACTGCCCATGTGACCATTGAATCACTGCCACCCGAAGTGGCTGAAGGAGAAAACGTCCTTTTCCTTGTGCGTGATCTTCCAAAGAATCTTATCGCCTTTGCTTGGTTCAAAGGGCAAACAAATATGACACAAGGAATTGCATGGTATACATTGGACAGCAATTTACGTGGGCCAGGGCCTGTGCACAGTGGTAGAGAGACAGTGTATCATAATGGATCCCTGCTGCTCCAAAATGTCACCCAGAAGGACACAGGACCTTATACCCTACAAATTTTTAATAGACGCAGAAAACTCATTTCAACAACATCCATTTACCTCCATGTGCATG

>Pca_Psg10N1 (Peromyscus californicus insignis; California mouse) WGS VALE02034109.1

CCTTCCTCTTAACCTTCTGGCATCTGTCTACCATGGCCCATGTGACCATTGAGTCACATCCACTCCAAGTGACTGAAGGAGAAAACGTCCTTTTCGTTGTCCATGATCTCCCAGAGAATCTTACAACCTTTGCTTGGTTCAAAGGTCTAACAAATATGACACAAAGAATTGCAATGTGTACAATGGACAAAAATTTAAGTGGGCCAGGACATGTGCACATTGGTAGAGAGACAATATATTGCAATGGATCCCTGTTGCTGGAAAATGTCAACCAGAAGGACACAGAAAATTATACCCTGCAAACCTATAATAGACATGGAAAAATCGTATCAACAACATCCATGTACCTCCATGTGTCTG

>Pca_Psg11N1 (Peromyscus californicus insignis; California mouse) WGS VALE02034109.1

CCTGCCTTTTAACCTCCTGGCACCTGTCCACCACTGCCCATGTGACTACTAAAACAGTGCCACCCCAAGTGGTTGAAGGAGAAAACGTCCTTTTCCTGGTTCATGGTCTTCCAGAGGATATTGTAGTCTTTGCCTGGTTCAAAGAGCTAAGAAATATGAAACAAGCAATTGCAGTATATGGAATGCACATCAATTTAAGTGCACCAGGGCCTGTGCACAGCGGTAGAGAAACATTATATCGCAATGGATCCATGCTGCTTGAAAAAGTTACCCGGAAGGACATAGGATTTTATACCCTGCGGACCTATGATAGAAATGTAAAAATCGTATCAACAACATCCACGTACCTCCATGTGGACA

>Pca_Psg12N1 (Peromyscus californicus insignis; California mouse) WGS VALE02034109.1

CCTCCCTTTTAAGCTGCTGGTATCCACCCACCACTGCCCAAATCACCATTGAACTCCTGCCACCCAACGTGTTCGAAGGAGACAATGTCCTTCTACATGTCCACAATCTGCCAGAGAATCTTCTAGCCTTTGCTTGGTTCAAAGGGCTGACAAATATGAAACGTGGAATCACCATCTATTCACTGAAATACAATTTAAGTGTCACGGGGCCTGTACACAGTGGAAGAGAGACAGTGTACAGCAATGGATCCCTGCTGCTCCAGCGTGTCACCCACAAGGACACAGGATTCTACACCCTACGAACCATAAGTAGACAAGCAGAAATTGTATCAGTGACATCCATGTACCTCCACGTGCACA

>Pca_Psg13N1 (Peromyscus californicus insignis; California mouse) WGS VALE02034109.1

TCTCCTTTTTAACCTGTTGGCACCTGACCACCACTGCCCAAGTCACCATTGAAATAATGCCACCCCAAGTGGTTGAAGGAGAAAATGTCATTTTCCTTGTCCGTAATCTTCCAGAGAAGCTTTTTGGCTTATCCTGGTTTAAAGAGGAGACAAATACGAAGTATAGAATTGCAAGCTATGAAATGGCCTACAGTCAGGATTTTCTGGGGGCAGCACACACTGGTAGAGAGACAGTGTACCCTAACGGATCCCTGTGGATTCAAAATGTCACCCAGAGTGACTCAGGAGTCTATATACTACGAAGCAGTAATAGAGAAAGAATTGTATCGTCAACATACATATACCTTCACGTGTACA

>Pca_Psg14N1 (Peromyscus californicus insignis; California mouse) WGS VALE02034109.1

TCTCCCTTTTAACCTGTTGGTACCGGCCCACCACTGCCCACGTCACCATTGAATCAGTGCCTGCCCATGTGGTTGAAGGAGAAAGCAACCTTTTTCTTGTCCACAATCTGCCAGACAATCTTTTAACCATATCCTGGTTTAAAGAAGAAGCAGATATAGACCATAAAATTGCGACCTACACACTGAAATACAATATTGCTGTGCCGGGGGCAGCACACAGCGGTAGAGAGACCGTGTACCCAAATGGATCCCTGTGGATTCAAAATGTCACCCACAAGGACACAGGATTCTATATACTAGAAACCAGAAGTAGAAAAGTAAAATTTATATCAACAATATACATACACCTTCATGTGTACA

>Pca_Psg15N1 (Peromyscus californicus insignis; California mouse) WGS VALE02034109.1

CCTCCCTTTTAACCTGTTGGTACCGGCCCAATACTGCCCAAGTCATCATTGAATCAGTGCCTCTCCATGTGGTCGAAGGAGAAAGCAAATTTTTTCTTGTCCACAATCTGCCAGAGAATCTTTTAACCATATCCTGGTTTAAAAAAAGAGCAAATATGGACCATAAAATTGCGACCTACACACTGAAATACAATATTGCTGTGCCGGGGGCAGCACACAGTGGTAGAGAGACCGTGTACCCAAATGGATCGCTGTGGATTCAAAATGTCACCCATAAGGACACAGGAATCTATATACTACAAACCAGAAGTAGAAAAGTAAAAATTGTATCAACAAAATATACATACCTTCATGTGTACA

>Pca_Psg16N1 (Peromyscus californicus insignis; California mouse) WGS VALE02034109.1

CCTCACTTTTAACCTGTTGGTACCGGCCCACCACTGCCCAAGTCATCATTGAATCAGTGCCTCCCCATGTGGTCGAAGGAGAAAGCAACCTTTTCCTTGTCCACAACCTGCCAGAGAATCTTTTAGCCATATCCTGGTTTAAAGAGGGAGCAAATATGGACCATAGAATTGTGACCTATACACTGAAATACAATATAGCTCTGCCGGGAACAGCACACAGCGGTAGAGAGACAGTGTACCCAAATGGGTCCCTGTGGATTCAAAATGTCACCCATAAGGACACAGGATTCTATATACTACAAACCATAAGTAGACAAGTAAAAATTGTATCAAGAACATACATACACCTTCATGTGTACA

>Pca_Psg17N1 (Peromyscus californicus insignis; California mouse) WGS VALE02034109.1

CCTCCCTTTTAACCTGCTGGCACCTGTCCACCGCTGCCCAAGTTACCATTGAATCAGTGCCGCCCCAAGTGGTTGAAGGAGAAAACGTCCTTCTACGTGCCAACAATCTGCCAGAGAATCTTCTAGCTTTTTCCTGGTACAAGGAGGTGAGAAATATGAACCTCAGAATTGCACTATTTGCACTGAACACTAATCAAAGTGTGATGGGGCCTGGACAAAGCGACAGAGAAACAGTGTACAGCAATGGATCCCTGTGGCTGAAAAATGTCACCAAGGAGGACACAGGATTCTATACCCTACAAACAGTAAATAGAGGTGGAAAAACTGTATCTACAACAACCACGTACTTCCATGTGTACA

>Pca_Psg18N1 (Peromyscus californicus insignis; California mouse) WGS VALE02034109.1

CCTCCATTTTAATCTGCTGGCACCTTTCAACCACTGATGATTTTACCATGGAACCTATCCCACCCCATGTGGCCAATGGAGACAATGTACTTCTTCTTGTCCACAATCTGCCCGAGAATCTTATAGCCTTCGCCTGGATCAAAGGGGAGATAAGAATGAATCATATAATTGCAATATATATACCAAACAAAAAGTTAAGTGTGCCAGGGCGTTTATATAGTGGTAGAGAGAAAGTGTATGGCAATGGATCCCTGCTCCTCCAAAATGTCAATGAGAAGGACACAGGAATTTATACCCTACAAACCTTTAATAGACGCACAGATACTGTGTCGCAAACATCCACGTACCTCTATGTTCACA

>Pca_Psg19N1 (Peromyscus californicus insignis; California mouse) WGS VALE02034109.1

CCTCCCTTTTAATCTGCTGTCATTTAGCCACCACTGCTAAAGTCACCATTGAATCAGTGCCACTCAATGTGTTCGAAGGAGACAATGTCCTTCTACATGTCCACCATCTGCCAGAGAATCTTCTAGCCTTTGCTTGGTTCAAAAGGCTAACAAAAACGAAACACAGAATTGCACTGTATGCACTGAACATCAATTTAGTTGTGCCGGGGCCTGTACACAGTGGTAGAGAGACAGTGTACCGCAATGGATCCCTGTGGATTCAGAATATCACCCATAAGGACACAGGATTCTACACCCTAGAGACCATAAATAGACATGGACGAACTGTATCAAAAACAACCATGTACCTCCATGTGTACA

>Pca_Psg21N1 (Peromyscus californicus insignis; California mouse) WGS VALE02034109.1

CCTCCTTTTTAACCTTTTGGCACCTGTCTACAACCGACCATGTGGCCATTGAATCACTGCCACCCCAAGTGGCTGAAGGAGAAAATGTCCTTTTCCTTGTCCAGAATCTGCCAGAGAATCTTACAGCCTTTGCTTGGTTCAGAGGGCTCAGAAGTATGAAACGAGGAATTGCAATGTATACACTGCACAACAATTTAAGTGATCTGGGGCCTGTGCACAGCGGTAGAGAGACAATACATCACAATGGATCCCTGCTGCTCGTAAAGGTCACCCAGAAGGACACAGGATACTATACGCTAAGAACATATAACAGACGGGGAAAAATCATATCAACAACATCCATGTACCTCCACGTGTATG

>Pca_Psg23N1_P (Peromyscus californicus insignis; California mouse) WGS VALE02034109.1

CCTCTCTTTTAACCTGTTGGCACCTACCCACCACTGTCCAAGTCATTATTGAATTAGTGCCACCTGATGTGGTTGAAGGAGAAAATGTCCTCCTCCTTGTCCGCGATCTGCCAGAGAATCTTGAAGCCTTTGCCTGGTACAAAGGGGTGACAAATATGAACAATTGTATTCGCTAACCACAAATTTAAGAGTGGCAGGGCCTGAATACAGTGGTAGAGAGACAGTGTACAGAAATGGATCCCTGCGTCTCCAAGATGTCACCCAGAAGGACACAGGATTCTATACCCTATGATCCATAAGCAGACATAAAGAAATCATATCAACAACATCCATATACCTCCACGTGTACT

>Pca_Psg24N1 (Peromyscus californicus insignis; California mouse) WGS VALE02034109.1

CCTCCATTTTAACCTGCTGGCACCTTTCCACCACTGATCGTTTTTCTGTGGAAACTGTCCCACCCAATGTGGCCAACGGAGACAGCATCCTTTTCCATGTCCGCAATCTGCCAAAGAATCTTCTATCCTTCGCCTGGATCAAAGGGGCAACAAGCATGAATGATGCAATCATAGTATATATACCAAACAAAAATTTAAGTGTGCCAGGGCGTTTTCACAGTGGTAGAGAGACAGTGTATGGCAATGGATCCCTGCTCATCCAAAATGTCAACCAGAAGGACACAGGAATCTATTCTCTACGAGCTTTTCATAGACGCACAGATACTGTGTCACAAATAGGCACATACCTCTACGTGAACA

>Pde_Ceacam9N (Praomys delectorum; delectable soft-furred mouse) WGS JADRCD010216220.1, JADRCD010011232.1

CCTCCCTCTTAACCTGCTGGAATGCACCCACCGCTGCTGAGCTCACTATCGAATTAGTGCCACCCACGGTTGCTGAAGGCGGAAATTCCGTTCTGTTTGTGCATGAAATGCCACTAAATGTCCAGGnGTTTTACTGGTACAAACAGAGAGATTCGACGAAGAGCTATGAAATTGCACGGTACTTAACACCCACTAACGAAAGTTCGAAGATGCCTCAACACCGTGATAGGAAAACCGTATTCTACAGTGGATCCCTGCTGATCAGAAACGTCACCCAGGCCGACAGTGGAGTCTACACCTTGCTAACATTCAACACAGAAATGGAAAGCGAATTAACACACGTGCATCTGGAAGTTCGCG

>Pde_Ceacam11N1_P (Praomys delectorum; delectable soft-furred mouse) WGS JJADRCD010191334.1

TCTCCCTTTTAACCTGCTGGCTGCTTTGGATTACCATTGAATCAGTGCCTCCCATTGCTGTTGAAGGGGAAAATGTTCTTCAGTTTGTGCATAACCTGTCAAAGAATGTTAAAACCCTTTCCTGGTACACAGGAGGTAAACCACTCAAGAGTTGTGAAAGTGCAAGTCATGTGATAGCTACGAATGCTAGTGTGCTGGAATTTGCACACAGTGGTAGAGAGACAGTACTCAACAATGGATCTCTGCTGATTAAGAGTGTCACCAGAAAAGACTCAGGATACTACACCCTAAAAACACTTGATACAACCTCAAGACCTGAAATAATTCATGCAGAATTCCTTGTTTAGA

>Pde_Ceacam12N1 (Praomys delectorum; delectable soft-furred mouse) WGS JADRCD010160340.1

TCTCCCTTTTAATCTGCTGGCTGCTTCCCACTACTGCCCATCTCACTATTGAATCAGTGCCTCCCATTGCTGTTGAAGGGGAAAATGTTTTGCTGTTTGTGCATAACCTGCCAGAGAATGTTCAAGCCCTTTCCTGGTACACAGGAGGTAAACCACTTAAGAGGTTTGAAATTACAAGACATGTGATAGCTACCAATTCTAGTATAATGGGACCTGCACACAGTGGTAGAGAGACAATACTCAACAATGGATCTCTGCTGATCAAGAGTGTCACCAGAAAAGACTCAGGATACTACACTCTACAAATACGTGATACAACCTCAAGACGTAAAATAACACGTGCAGAATTCTTTGTACAGG

>Pde_Ceacam13aN1 (Praomys delectorum; delectable soft-furred mouse) WGS JADRCD010074982.1

TCTCCCTTTTAACCTGCTGGCTGCTTCCCACTACTGCCCAGCTCACCATAAAATCAGTGCCTCCAATTGCTGTTGAAGGGGAAAATGTTCTTCTGTTTGTGCATAACCTGAAGAATGTTAAAGCCTTTACCTGGTATACAGGACCTGCACCATTCAAGTGTTGTGAAATTGCAAGTCATGTGATAGCCACCAATTTTACTGTGGCAGGACTTGCACACAGTGGTAGAGAGACAGTACTCAACAATGGATCTCTGCTGATCAAAAGTGTCACCAGAAAAGACTCAGGATACTACACTCTACGAACCCTTGATTCAACCTCAAGACCTGAAATAGTACATACAGAATTCTTCGTACACA

>Pde_Ceacam13bN1_P (Praomys delectorum; delectable soft-furred mouse) WGS JADRCD010216316.1

TCTCCCTTTTAATCTGCTGGCTGCTTCCCACTACTTCCCAGCTCACCATTAAATCAGTGCCTCCAATTGCTGTTGAAAGGAAAAATGTTCTTCTGTTTGTGCATAACCTACCGAAGAATGTTAAAAACTTTTCCTGGTACACAGGAGTTACCATGCTCAAGAGCTGTGAAATTGCAAGTCATTTGATAGCTACCAATTTTACTGTAATGGGACTTGCACACGATGGTAGAGAGACAGTACTCAACAATGGATCTTTGCTTATCAAGGGTGTCACCAGAAGAGATTCAGGATACTACACTCTATGAACACTTGATGAAACCTCAAGATCGGAAGTAATAAGTGCAGAATTCTTTGTACACT

>Pde_Ceacam14N1 (Praomys delectorum; delectable soft-furred mouse) WGS JADRCD010212421.1

TCTCCCTTTTAATCTGCTGGCTGCTTCCCACTACTTCCCAGCTCACCATTAAATCAGTGCCTCCCATTGCTGTTGAAGGAGAAAATGTTCTGTTTGTGCATAACCTGCCGAAGAATGTTAAAGCCTTTTCCTGGTACACAGGAGTTACAGCTCTCAAGAGTTGTGAAATTGCAAGTCATGTGATTGCTACCAAAATTACTGTGGTGGGACTTGCACACAGTGGTAGAGAGACACTATTCAACAATGGATCTTTGCTGATTAAGAGTGTCACCAGAAAGGACTCAGGATTCTACACTCTACGAATACTTGATGCAACCTCAAGACCTAAAATAATACGTGTAGAATTCTTTGTGCACA

>Pde_Ceacam15N (Praomys delectorum; delectable soft-furred mouse) WGS JADRCD010213924.1

CCTCACTTTTATTCTGCTGGAGCTCGCCCACAGCGGCGGCCCTGCTAACATCTAAAGAAATGCGCTTCTCGGCTGCGGAAGGGGCAAAGGTTCTTCTCTCTGTTCCTGACCAGGAAGAGGACCTCCTCTCCTTCTCCTGGTACAAAGGGAAGAATGTACATGAAAATTTTACAATTGCACATTATAAAAAGTCCAGCGATTCACTTCAACTTGGGAAGAATGTCAGCGGCAGGGAAGAAATCTATAAGGATACGTCCATGATCCTCCAGGCCGTCACCCAGGAAGACACGGGATTCTACACTTTACAAACCTTTAAAGCACACAATCAACAGGAGATAACATATGTCCATCTCCAAGTATACA

>Pde_Psg36N1 (Praomys delectorum; delectable soft-furred mouse) WGS JADRCD010074007.1

CCTCCCTCTTAACATGCTGGCTCCTGTCCACCACTGCTCAAATTGAAATCGAATCCTTACCACCCCAAGTGGTTGAAGGAGAAAATGTTCTTTTACGTGTGGACAATCTGCCAGAGGATATTATAGCCTTTGTCTGGTACAAAGGGGTGACAGACATGAGCCTTGGAATTGCACTGTATTCACTGACCTACAGTGTAAGTGTGACGGGGCCTGTGCACAGCGGTAGAGAGACACTGTACAGCAACGGGTCCCTGTGGATCCAAAATGTCACCCAGGAGGACACAGGATTCTACACCCTACGAACAATAAGTAAACGTGTAGAAATTGTATCAAATACGTCCATGCACCTTCACGTGTACT

>Pde_Psg37N1 (Praomys delectorum; delectable soft-furred mouse) WGS JADRCD010170483.

CCTCTGTCTTAACCTGTTTGCTCCTGCCCACCACTGCCCAAATCACCGTGGAATCTTTACCACCCAAAGTGGTTGAAGGAGAAAGTGTTCTTCTACTTGTTGACGGTCTGCCAGAGAATGTTGTAGCCTTTGCCTGGTACAAAGGGGTGACAGACATGAGCCTCGGAATTGCACTGCATTCACTGACCTATAGAATAAGTATGACAGGGCCTGTGCACAGTGATAGAGAGATATTGTACAGCAACGGGTCCCTGTGGATCCAAAATGTCACCCAGGAGGACACAGGATTCTACACTCTTCGAACCATAAGTAAACATGGAGAAATGGTATTAAATGTATCCACGTACCTTCGGGTGTACT

>Pde_Psg38N1 (Praomys delectorum; delectable soft-furred mouse) WGS JADRCD010175966.1

CCTCCCTTTTGACCTGCTGGCTCCTGACCACGGCCGAGGTCACCATTGAATCGGTGCCATTCAATGCGGTCGAAGGAGAAAATGTCCTTCTTCTTGTTCACAATCTGCCAGAGAATCTTCTAGCCTTAGCATGGTACAGAGGGCTGAGGAAAATCATTGTATACACACTCAACACTAAAGTAAGTGTGATGGGTATTATGCATAGCGGTAGAGAGACAGTGTCCAGCAACGGGTCCCTGTGGATCCACGATGTCACCCAGAAGGACACAGGATTCTATACCCTACGGACCATAAATAGACGTGGAGAAATTGTGTCAACAACATCCACGTACCTCTACGTGTACT

>Pde_Psg39N1 (Praomys delectorum; delectable soft-furred mouse) WGS JADRCD010087701.1

CTTCTGTCTTAACCTGTTGGCTCCTGCCCACCACTGCCCGAGTCACCATGGAATCTTTACCGCCCAAAGTGGTTGAAGGAGAAAATGTTCTTCTACTTGTTGACGGTCTGCCAGAGAATGTTGTAGCCTTTGCCTGGTACAAAGGGGTGACAGACATGAGCCTCGGAATTGCACTGTATTCACTGACCTATAGAAGAAGTGTGACAGGGCCTGTGCACAGTGGTAGAGAGATATTGTACAGCAACGGGTCCCTGTGGATCCAAAATGTCACCCAGGAGGACACGGGATTCTACACTCTTCGAACCATAAGTAAACATGGAGAAATGGTATTAAATGTATCCACGTACCTTGAGGTGCACT

>Pde_Psg40N1_P (Praomys delectorum; delectable soft-furred mouse) WGS JADRCD010181589.1

CCTCCCTTTTAACTTGCTGGTACTTTTCTACCACTTCCCAAGTCACCATTGAATTAGTGCCACCCCAAGTGGTTGAAGGAGATGTCCTATTCCTTCTCCACAAACTACCAGAAATTCTTATGTCCTTAGGCAGGTTCAAAGGGATGACAGTTATAAAACGTGTACTCACACTGTATGCAACAAACACTAAAGTATGACAGGGCCCATGCACAGTGCTAGAGAGACCTTGTACAGAAACAGGTCCCTGTTGATCCACAATGTCACCCAAAAGGACACAGGATTCTATACCCTATGAACCTTAAATAGAATTGGAGATATTGTGTCAACATCCATGTTCCTCTACGTGAACC

>Pde_Psg41N1 (Praomys delectorum; delectable soft-furred mouse) WGS JADRCD010244611.1

CTTCCTTTTTAACCTGCTTGCTCCTGCCCACTACTGCCCAGGTCACCATTGAATCAGTGCCACCTCAAGTGGTTGAAGGAGAAAACGTTCTTTTTCTTGTACACAATTTGCCAGAGAATCTTATAGCTTTAGTCTGGTTAAGAAGACTGAGGAAAATGAACTGCACAATTGGCCTATATGCAATGAACACTAAAATAAGTGTGATGGGGCCCATGCACAGCGGTAGAGAGATAGTGTCCAGCAATGGTTCCCTGTGGATCCACAATGTCACCCAGAAGGACACAGGATTCTACATCCTACAAACCGTAAATAGACGTGGAGAAATTATGTCAAGAACACCCATGTATCTCTACGTGTACA

>Pde_Psg42N1 (Praomys delectorum; delectable soft-furred mouse) WGS JADRCD010177236.1

CCTCCCTTTTAACATGCTGGCACCTGTCTACCACTTCCAAAGTCACCATTGAATTATTGCCACCACAAGTGGTTGAAGGGGAAGATGTTCTTTTCCTTGTCCAAAATCTGCCAGAGGATCTTGCAGCCTTTGCCTGGTTTAAAGGGAGGACAAATAAGAAACGTGGAATTGCACTGTATGCAGTGGCCTCTGACATACATGTACATAGTGATAGAGAGACATTGTACAGCAATGGATCCCTGATGATCCACAATATCACCCAGAAGGACAGAGGTTACTACACTCTACGAACCTTCAATAAACATTCAGAAACGATATCAACAACATCCACATTCCTCCATGTGAACC

>Pde_Psg43N1_P (Praomys delectorum; delectable soft-furred mouse) WGS JADRCD010212016.1

CTTTTCTTTTAACCTCCTGGTTCCTGCCCAACACTGTCTAAGTCACCATTGAATTAGTGCCACCCCAAGTTGCTGAAGGAGAAAATGTCCTCCTTCTTGTTTACAATCTGCCAGAGAATCTTATAGCCATAGCCTGGTTCAAAGAAGTGACAAGTATGAACCTTAGAATTGTGTTGTATGCACTGGCCTCTAACATTAGTGTGACAGGGCCTGAACACAGCGGTAGAGAGACAATGTACAGAAATGGATCCCTGCTGCTTCATAATGTCACCCAGAAGGACACAGGATTCTATACTCTACGGACCTTAAATAGACATGGAAAAATTGTATCAACAACATCCATATACATCCATGTGTACA

>Pde_Psg44N1 (Praomys delectorum; delectable soft-furred mouse) WGS JADRCD010189830.1

CCTCCTTTTTAACCTGCTGGCATCTGCCTACCACTGCCCGAATAACCATTGAATTAGTGCCTCCCCAAGTGATTGAAGGAGAAAATGTTCTCATACGTATCAAAAATCTGCCAGACAATCTTATAACCTTAGCCTGGTTCAGAGGAAAGAGGATTGAGAGCCCTCAAATTGGACAATATACACTGGCCACTAATGTTACTGTGGTGGGGCCTGCTCACAGTGGTAGAGAGACTTTGTACAGCGATGGATCCCTGCAGATCTACAATGTCACCCAGGAGGACATAGGATTCTACAGCCTACAAATTATAAATAGACACGCAGAAATTGTGTCAATAACATCCATATACCTCAACGTGTACT

>Pde_Psg45N1 (Praomys delectorum; delectable soft-furred mouse) WGS JADRCD010235072.1

CCTCCTTTTTAACCTGCTGGCATCTGCCTACCACTGCCCGAATAACCATTGAATTAGTGCCTCCCCAAGTGATTGAAGGAGAAAATGTTCTCATACGTATCAAAAATCTGCCAGACGACCTTATAACCTTAGCCTGGTTCAGAGGAATGAGGATTAAGAGCCCTCAAATTGGACAATATACACTGGCCACTAATGTTACTGTGGTGGGGCCTGGCCACAGTGGTAGAGAGACTTTGTACAGCGATGGATCCCTGCAGATCTACAATGTCACCCAGGAGGACATAGGATTCTACAGCCTACGAATCATGAATAGACATGCAGAAATTGTGTCTATAATGTCCATATACCTCAATGTGTACT

>Pde_Psg46N1 (Praomys delectorum; delectable soft-furred mouse) WGS JADRCD010168212.1

CCTCCCTTTTAACATGCTGGCACCTGTCCACCACAGCCAAGATCACCATTGAATTATTGCCACCCCACGTGGTTGAAGGAGAAGATGTCCTTTTTCTTGTCCACAGTCTGCCAGAGGATCTTGCAGCCTTTGCCTGGTTTAAAGGGAGGACAAATAAGAAACGTGGAATTGCACTGTATGCAGTGGCCTCTGACATACATATACACAGTGATAGAGAGACATTGTACAGCAATGGATCCCTGATGATCCACAATATCACCCAGAAGGACAGAGATTATTACACCCTACGAACCTTCAATAAACATGCAGAAACTGTATCAACAACATCCACATTCCTCCATGTGAACC

>Per_Ceacam9N (Peromyscus eremicus; cactus mouse) WGS CACRXM010000001.1

CCTCCCTCTTGACCTGCTGGAATGCACCTACTACTGCCGAGCTCACTATTGAATTAGTGCCTCCCATGGTGGCTGAAGGCGGAAACTCCGTTCTGTTTGTGCACAAAATGCCGCTGAACGTCCAGGCATTTTACTGGTACAAACAGAAAGATCCGACCAAGAGCTATGAAGTTGCCCGGTACTTAACACCCGATAACACAACTTCGAAGATGCCTCAACACAGCGGCAGGAAAACGGTGTTCTACAGTGGATCCCTTCTGATCAGAAACGTCACCCAGGCTGACAGCGGATTCTACACCTTACTAACATTTAACACAGAAATGGAAAGCGAACTAACACACGTGTATCTGGAAGTCCACA

>Per_Ceacam11N1 (Peromyscus eremicus; cactus mouse) WGS CACRXM010000001.1

CCTCCCTTTTAACCTGCTGGCTGCCTCCTACTGTTGCCCAGCTCACCATTGAATCAGTGCCACCCATTTCTGCTGAAGGGGATAATGTTCTTCTGCTTGTGCACAACCTTCCTGAGAATGTTCAAGCCTTCTCCTGGTACACAGGAGTTATGGTGCTCAAGAGCCGTGAAATTGCAAGATGTGCAATAGCTACCAATTCATGTGTGCTGGGGCCTGCACACAGTGGTAGAGAGACAGCATTCAATAATGGATCTCTGCTGATCAAGAATGTCACCAGGAAGGACTCAGGATACTACATCCTACAAACACTTAATACAAATTCGAGATCTGAAATAACACGTGCAGAATTTTTTGTACACA

>Per_Ceacam15N (Peromyscus eremicus; cactus mouse) WGS CACRXM010000024.1

CCTCACTTTTAACTTGCTGGAACTCCCCCACCCTGGCGCTAAGAACTATTAAAGAAATGCAGTTTTCTGCTGCTGAAGAGGGGAAGGTTCTTCTCTCTGTTTCTATTCAGGCAGAGAACCTTCTCTCTTTTCACTGGTACAAAGGGAAAGATGTAAATAAAGATTTTACAATTGCCCATTATGAAAAGGACACAGACTTACTTAAACTTGGGATTAAAACCAGAGGCAAGGAAGAGATGTATAAGGATGGATCCATGATGCTCCAGGATGTTACCCAGGAAGATATGGGAATCTATACCCTAGAAACCTTTGGAACACATGATCAACATGAAATAACACATATCTACCTCCAAGTGTACA

>Per_Psg1N1 (Peromyscus eremicus; cactus mouse) WGS CACRXM010000001.1

CCTCCATTTTAACCTGCTGGCACCTTTCCACCACTGACCATATCACCATTAAATCTGTCCCATCCCAAGTGGCCAGTGGAGACAACGTCCTTCTTCTTGTCAACAAACTGCCAGAAGATCTTTTAACCTTCGCCTGGTTTAAAGGCGAGTCAGGCATGAACCTTGGAATTGCAATATATGCACCAGACAGAGATTTAATCATGCAAGGGCCTGGATATAGCGGTAGAGAGACAGTGTACCGCAATGGATCCCTGCTGATCCAAAATGTCAATGAGAAGGACACAGGACTCTACACCCTGCAAACCTTAAATGAACATGGAGATGTTCTGTCAGTAACAACTATGCGCCTGTATGTGTACC

>Per_Psg2N1_P (Peromyscus eremicus; cactus mouse) WGS CACRXM010000001.1

CCTCCTTTTTTATCATGCTGGCACTTTTCCACCACTGCCCGATTTGCCCTTGAATCTATCCCACCTGAAGTCATCGAAGGAGAAAATGTCCTTTTCCTTGTCCACAACCTGCCAGAGAATCTTGCAGCCGTAGTCTGGTCTAAAAGGGTGAAAAGTATGAACCATGGAATTGTAACATATGCACTGAACAAAGATTCAAGTGTGCCAAGGCCTCTACACAGTGGTAGAGAGACAGTGTTCCGCAATGGATCCCTGCTGTTCAGAAATGTCACCACGAAGGATACAGGACTTTATACCATAGAGCTCTTAGACAGACTTGGAGATATTGTGTCAACAACAACCGCTTATCTTTGCGTGCACA

>Per_Psg3N1 (Peromyscus eremicus; cactus mouse) WGS CACRXM010000001.1

CCTCCCTTTTAACTTTCTGGCACCTGTCCACCACTGCCGATGTGACCATTGAATCACTGCCGCCCCTAGTGGCCGAAGGAGAAAATGTCCTTTTCCTTGTCCACCATCTGCCAGAAAATCTTACGGCCTTAGCCTGGTTCAAAGGACTAACAAATATGAAACAAGGAATTGCAATGTATACACTACACAACAATTTAAGTGTGACAGGGCCTGTGCACAGTGGTAGAGAGACAGTATATCACAATGGATCTCTGTTGCTGGAAAACGTCACCCAGAAGGACACAGGATACTATACGCTACGAACCTATAACAGACGTGTAAAAATCATATCAACAACATCCATGTACCTCCAAGTGCACG

>Per_Psg4N1 (Peromyscus eremicus; cactus mouse) 21.2.21 WGS CACRXM010000001.1

CCTCCCTATTAACCTTCTGGCACCTGTCCACCACTGCCCATGTGACCATTGAATTACTGCCACCTGAAGTGGCTGAAGGAGAAAACATTCTTTTCCTTGTCCATGGTCTGCCAAAGAATCTTACAGCCTTTGCCTGGTTCAAAGGGCAAACAAATATGACACATGGAATTGCATGGTATACACTGGACAACAATTTACGTGGGCCAGGGCCTGTAAACAGTGGTAGAGAGACAGTGTATCACAATGGATCCCTGCTGCTTCAAAATATCACCCACAAGGACACAGGAACCTATACCCTACGAATCTATAATAGACGTGGAAAAATCATATCAACAACATCCATTTACCTCCATGTGCCTG

>Per_Psg5N1 (Peromyscus eremicus; cactus mouse) WGS CACRXM010000001.1

CCTCCCTCTTAACCTCCTGGCACCTGTCCACCACTGCCCATGTGACCATTGAGTCACTTCCACTCCAAGTGGCTGAAGGAGAAAACGTCCTTTTCCTTGTCCATGATCTGCCAAAGAATCTTGTAACCTTTGCCTGGTTTAAAGGGCTGACAAATACGACACAAAGAATTGCAATGTATACAATGGACAGCAATGTAAGTGGGCCAGGACATGTGCACAGTGATAGAGAGACAATATATCATAATGGATCCCTGTTGATCGAAAATGTCACCCAGAAGGATGCAGGAATCTATACCCTACGAACCTATAATAGACATGGAAAAACTGTATCAACAACATCCATGTACTTTCATGTGTCTG

>Per_Psg7N1 (Peromyscus eremicus; cactus mouse) WGS CACRXM010000001.1

CCTTCCTTTTAACCTTCTGGCTCCTGTCCACCACTGCCACTGTAAACACTGAATCAATGCCACTCCTAGTGGCTGAAGGAGAAAACATCCTTTTCCCTGTCCAAGATCTTCCAGAGAATATTATAGCCGTAGCCTGGTTCAAAGGACTAACAAAGATGACACAAGGAATTGCTTTGTATGCACTGCACAGTGGTTTAAGTTGCCCAGGTTCTGTGCACAGTGGTAGAGAGACAATATATCGCAATGGATCCCTGCTGCTGGAGAAGGTCACCCAGAATGACACAGGATTTTATACCCAACGAACCTTTAATAGACACAGAAAAGTCATGTCAACAGCATCCATTTACCTCCATGTGTCTG

>Per_Psg9N1 (Peromyscus eremicus; cactus mouse) WGS CACRXM010000001.1

TCTCCCTGTTAACCTTCTGGCACCTGTCTACCACTGCCCATGTGACCATTGAATCACTGCCACCCGAAGTGGCTGAAGGAGAAAACGTCCTTTTCCTTGTGCGTGATCTTCCAAAGAATCTTGTCGCCTTTGCTTGGTTCAAAGGACAAACAAATATAACACGAGGAATTGCATGGTATACATTGGACAACAATTTACGTGGGCCAGGGCCTGTGCACAGTGGTAGAGAGACAGTGTATCGCAATGGATCCCTGCTGCTCCAAAATGTCACCCAGAAGGACACAGGACCCTATACCCTACAAATTTTTAATAGACACAGAAAACTCATTTCAACAACATCTATTTACCTCCATGTGCACG

>Per_Psg10N1 (Peromyscus eremicus; cactus mouse) WGS CACRXM010000001.1

CCTTCCTCTTAACCTTCTGGCATCTGTCTACCATGGCCCATGTGACCATTGAGTCACTTCCAGTCCAAGTGACTGAAGGAGAAAACGTCCTTTTCATTGTCCATGATCTCCCAGAGAATCTTACAACCTTTGCCTGGTTCAAAGGTCCAACAAATATGACACAAAGAATTGCAATGTGTACAATGGACAACAATTTAAGTGGGCCAGGACATGTGCACATCGGTAGAGAGACAATATATTGCAATGGATCCCTGTTGCTTGAAAATGTCAGCCAGAAGGACACAGAAAATTATACCCTACAAACCTATAATAGACATGGAAAAATCGTATCAACAACATCCATGTACCTCCATGTGTCTG

>Per_Psg11N1 (Peromyscus eremicus; cactus mouse) WGS CACRXM010000001.1

CTTGCCTTTTAACCTCCTGGCACCTGTCCACCACTGCCCATGTGACCATTAAAACAGTGCCACCCCAAGTGGCTGAAGGAGAAAATGTCCTTTTCCTGGTTCATGGTCTTCCAGAGAATATTATATGCTTTGCCTGGTTCAAAGAGCTAAGAAATATGAAACAAGCAATTGCAGTATATGGAATGCACATCAATTTAAGTGCACCAGGGCCTGTGCACAGTGGTAGAGAAACATTATATCGCAATGGATCCATGCTGCTTGAAAAAGTTACCCGGAAGGACATAGGATTTTATACTCTGCGGACCTATGATAGAAATGTAAAAATCATATCAACAACATCCACATACCTCCATGTGGACA

>Per_Psg12N1 (Peromyscus eremicus; cactus mouse) 21.2.21 WGS CACRXM010000001.1

CCTCCCTTTTAAGCTGCTGGTATCCACCCACCACTGCCCAAATCACCATTGAACTCCTGCCACCCAACGTGTTCGAAGGAGACAATGTCCTTCTACATGTCCACAATCTGCCAGAGAATCTTCTAGCCTTTGCTTGGTTCAAAGGGGTGACAAATATGAAACGTGGAATTGCCATCTATTCACTGAAATACAATTTAAGTGTCACGGGGCCTGTACACAGCGGAAGAGAGACAGTGTACAGCAATGGATCCCTGCTGCTCCAGGGTGTCACCCACAAGGACACAGGATTCTACACCCTACGAACCATAAGTAGACAAGCAGAAATTGTATCAGTGACATCCATGTACCTCCACGTGCACA

>Per_Psg13N1 (Peromyscus eremicus; cactus mouse) WGS CACRXM010000001.1

CCTCCTTTTTAACCTGTTGGCACCTGACCACCACTGCCCAAGTCACCATTGAAATAATGCCACCCCAAGTGGTTGAAGGAGAAAATGTCATTTTCCTTGTCCGTAATCTTCCAGAGAAGCTTTTTGGCTTATCCTGGTTTAAAGAGGAGACAAATACGAAGTATAGAATTGCAAGCTATGAAATGGCCTACAGTCAGGGTTTTCTGGGGGCAGCACACACTGGTAGAGAGACAGTGTACCCTAACGGATCCCTGTGGATTCAAAATGTCACCCAAAGTGACTCAGGAGTCTATATACTACGAAGCAATAATAGAGAAAGAATTGTATCGTCAACATACATATACCTTCACGTGTACA

>Per_Psg14N1 (Peromyscus eremicus; cactus mouse) WGS CACRXM010000001.1

CCTCCCTTTTAACCTGTTGGTACCAGCCCACCACTGCCCAAGTCACCATTGAATCAGTGCCTGCCCATGTGGTCGAAGGAGAAAGCAACCTTTTTCTTGTCCACAATCTGCCAGACAATCTTTTAACCATATCCTGGTTTAAAGAAGAAGCAGATATGGACCATAGAATTGCGACCTACACACTGAAATACAATATTGCTGTGCCGGGGGCAGCACACAGTGGTAGAGAGACCGTGTACCCAAATGGATCCCTGTGGATTCAAAATGTCACCCACAAGGACACAGGATTCTATATACTAGAAACCAGAAGTAGAAAAGTAAAAATTATATCAACAATATACATACACCTTCACGTGTACA

>Per_Psg15N1 (Peromyscus eremicus; cactus mouse) WGS CACRXM010000001.1

CCTCCCTTTTAACCTGTTGGTACCGGCTCAATACTGCCCAAGTCATCATTGAATCAGTGCCTCTCCATGTGGTCGAAGGAGAAAGCAACCTTTTTCTTGTCCACAATCTGCCCGAGAATCTTTTAACCATATCTTGGTTTAAAAAAATAGCAAATATGGACCACAAAATTGCGACCTACTCACTGAAATACAATATTGCTGTGCCGGGGGCAGCACACAGTGGTAGAGAGACCGTGTACCCAAATGGATCCCTGTGGATTCAAAATGTCACCCATAAGGACACAGGAATCTATATACTACAAACCAGAAGTAGAAAAGTAAAAATTGTATCAACAAAATATACATACCTTCATGTGTACA

>Per_Psg16N1 (Peromyscus eremicus; cactus mouse) WGS CACRXM010000001.1

CCTCCCTTTTAACCTGTTGGTACCAGCCCACCACTGCCCAAGTCATCATTGAATCAGTGCCTCCCCATGTGGTCGAAGGAGAAAGCAACCTTTTCCTTGTCCACAATTTGCCAGAGAATCTTTTAGCCATATCCTGGTTTAAAGAGGGAGCAAATATGGACCATAGAATTGCGACCTATACACTGAAATACAATATAGCTGTGCCAGGAACAGCACACAGCGGTAGAGAGACAGTGTACCCAAATGGGTCCCTGTGGATTCAAAATGTCACCCATAAGGACACAGGATTCTATATACTACAAACCATAAGTAGACAAGTAAAAATTGTATCAAGAACATACATACACCTTCACGTGTACA

>Per_Psg17N1 (Peromyscus eremicus; cactus mouse) WGS CACRXM010000001.1

CCTCCCTTTTAACCTGCTGGCACCTGTCCACCACTGCCCAAGTTACCATTGAATCAGTGCCGCCCCAAGTGGTTGAAGGAGAAAACGTCCTTCTACGTGTCAACAATCTGCCAGAGAATCTTCTAGCTTTTTCCTGGTACAAGGAAGCGAGGAATATGAACCTCAGAATTGCACTATTTGCACTGGACACAAATCTAAGTGTGATGGGGCCTGAACAAAGTGACAGAGAAACAGTGTACAGCAATGGATCCCTGTGGCTGAAAAATGTCACCAAGAAGGACACAGGATTCTATACCCTACAAACAGTAAAGAGAGGTGGAAAAATTGTATCTACAACAACCATGTACTTCCATGTGTACA

>Per_Psg18N1 (Peromyscus eremicus; cactus mouse) WGS CACRXM010000001.1

CCTCCATTTTAATCTGCTGGCACCTTTCAACCACTGATGATTTTACCATGGAACCTGTCCCACCCCATGTGGCCAATGGAGACAATGTACTTCTTCTTGTCCACAATCTGCCCGAGAATCTTATAGCCTTCGCCTGGATCAAAGGGGAGATAAGCATGAATCATATAATTGCAATATATATACCAAACAAGAAGTTAAGTGTGCCAGGGCGTTTATATAGTGGTAGAGAGAAAGTGTATGGCAATGGATCCCTGCTCCTCCAAAATGTCAATGAGAAGGACACAGGAATTTATATCCTACAAACCTTTAATAGACGCACAGATACTGTGTCGCAAACATCCATGTACCTCTATGTTCACA

>Per_Psg19N1 (Peromyscus eremicus; cactus mouse) WGS CACRXM010000001.1

CCTCCCTTTTAATCTGCTGTCATTTAGCCACCACTGCTAAAGTCACCATTGAATCAGTGCCACTCAATGTGTTCGAAGGAGACAATGTCCTTCTACATGTCCACAATCTGCCAGAGAATCTTCTAGTCTTTGCTTGGTTCAAAAGGCTGACAAAAACAAAACACAGAATTGCACTCTATGCACTGAACATCAATTTAGTTGTGCCGGGGCCTGTGCACAGTGGTAGAGAGACAGTGTACCGCAATGGATCCCTGTGGATTCAGAATATCACCCGTAAGGACACAGGATTCTACACCCTAGAGACCATAAATAGACATGGACGAACTGTATCAAAAACAACCATGTACCTCCATGTGTACA

>Per_Psg21N1 (Peromyscus eremicus; cactus mouse) WGS CACRXM010000001.1

CCTCCTTTTTAACCTATTGGCACCTGTCTACTACAACTGACCATGTGGCCATTGAATCACTGCCACCCCAAGTGGCCGAAGGAGAAAACGTCCTTTTCCTTGTCCAGAATCTGCCAGAGAATCTTATAGCCTTTGCTTGGTTCAGAGGGCTCAGAAGTATGAAACGAGGAATTGCAATGTATACACTGCACAACAATTTAAGTGATGCAGGGCCTGTGCACAGCGGTAGAGAGACAGTATATCACAATGGATCCCTGCTGCTGGAAAAGGTCACCCAGAAGGACACAGGATACTATACGCTACGAACATATAACAGACAGGGGAAAATCATATCAACAACATCCATGTACCTCCACGTGTATG

>Per_Psg23N1 (Peromyscus eremicus; cactus mouse) WGS CACRXM010000001.1

CCTCTCTTTTAACCTGTTGGCATGTACCCACCACTGTCCAAGTCATTATTGAATTAGTGCCACCTGATGTGGTTGAAGGAGAAAATGTCCTCCTCCTTGTTCGCGATCTGCCAGAGAATCTTGAAGCCTTTGCCTGGTACAAAGGGGTGACAAATATGAACCTTGGAATTGTACTTTATTCGCTAACCACAAGTTTAAGAGTGGCAGGGCCTGAATATAGTGATAGAGAGACAGTGTACAGAAATGGATCCCTGAGTCTCCAAGATGTCACCCAGAAGGACACAGGATTCTATACCCTACGATCCATAAGAAGACATAAAGAAATCATATCAACAACATCCATATACCTCCACGTGTACT

>Per_Psg24N1 (Peromyscus eremicus; cactus mouse) WGS CACRXM010000001.1

CCTCCATTTTAACTTGCTGGCACCTTTCCACCACTGATCGTTTTTCTGTGGAAACTGTCCCACCCAATGTGGCCAACGGAGACAGCATCCTTTTCCTTGTCCGCAATCTGCCAAAGAATCTTCTATCCTTCGCCTGGATCAAAGGGGCAACAAGCATGAATGATGCAATCATAGTATATATACCAAACAAAAATTTAAGTGTGCCAGGGCGTTTTCACAGTGGTAGAGAGACAGTGTATGGCAATGGATCCCTGCTCATCCAAAATGTCAACCAGAAGGACACAGGAATCTATTCTCTACGAGCTTTTCATAGACGCACAGATACTGTGTCACAAATAGGCACATACCTCTACGTGAACA

>Ple_Ceacam9 (Peromyscus leucopus; white-footed mouse) WGS NMRJ02000002.1

CCTCCCTCTTGACCTGCTGGAATGCACCCACTACTGCCGAGCTCACTATTGAATTAGTGCCTCCCATGGTGGCTGAGGGTGGCAACTCCGTTCTCTTTGTGCACAAAATGCCGTTGAACATCCAGGCGTTTTACTGGTACAAACAGAAAGACCCGACGAAGAGCTATGAAGTCGCCCGGTACTTAACACCCGACAACACAACTTCGAAGATGCCTCAACAGAGCGGCAGGAAAACGGTGTTCTACAGTGGATCCCTGCTGATCAGAAACGTCACCCAGGCTGACAGCGGATTCTATACCTTACTAACATTTAACACGGAAATGGAAAGTGAACTAACACACGTGTATCTGGAAGTCCACA

>Ple_Ceacam11N1 (Peromyscus leucopus; white-footed mouse) WGS NMRJ02000002.1

CCTCCCTTTTAACCTGTTGGCTGCCTCCTACTGTTGCCCAGCTCACCATTGAATCAGTGCCACCCATTTCTGCTGAAGGTGATAATGTTCTTCTGCTTGTGCACAACCTTCCTGAGAATGGTCAAGCCTTTTCCTGGTACACAGGAATTATGGTGCTCAAGAGCCGTGAAATTGCGAGATGTGCAATAGCTACCAATTCATGTGTGCTGGGGCCTGCACACAGTGGTAGAGAGACAGCATTCAATAATGGATCTCTGCTGATCAAGAATGTCACCAGGAAGGACTCAGGATACTACATCCTACAGACACTTAATACAAATTCAAGATCTGAAATAACACGTGCGGAATTTTTTGTACACA

>Ple_Ceacam15N (Peromyscus leucopus; white-footed mouse) WGS NMRJ02000002.1

CCTCACTTTTAACCTGCTGGAACTCACCCACTGCGGCGCTAAGATCTACCAAAGAAATGCGGTTTTCTGCAGCTGAAGGGGGGAAGGTTCTTCTCTCTGTTCCTATTCAGGCAGAGAATCTTCTCTCCTTTCGCTGGTACAAAGGGAAAGAAGAAGACCAAGATTTTACAATTGCCCATTATGAAAAGGACACAGATTCACTTAAATTTGGGAATGCAACCAGCGGCAGGGAAGAGGTATATAAGGATGGGTCCATGATGCTCCGGGACGTCACCCAGGAAGATACAGGGATCTACACCCTAGAAGCCTTTGGAACACATGATCACATTGAAATAACACATTTCTACCTCCAGGTGTACA

>Ple_Psg1N1 (Peromyscus leucopus; white-footed mouse) WGS NMRJ02000002.1

CCTCCATTTTCACCTGCTGGCACCTTTCCACCACTGACCATATCACCATTAAATCTGTCCCATCTCAAGTGGCCAATGGAGACAACGTCCTTCTTCTTGTCAACGATTTGCCTGAAGATCTTCTAACCTTTACCTGGTTTAAAGGCGAGACAGGCATGGACCTTGGAATTGCAAGATATGCACCAGACAGAGATTTAATCATGCAAGGGCCTGGATATAGTGGTAGAGAGACAGTGTACCGCAATGGATCCCTGCTGATCCAAAATGTCAATGAGAAGGACACAGGACTCTACACTCTGCAAACCTTAAATGAACATGGAGATGTTCTGTCAATAACAACTATGCGCCTGCATGTGTACC

>Ple_Psg2N1 (Peromyscus leucopus; white-footed mouse) WGS NMRJ02000002.1

CCTCGCTTTTATCGTGCTGGTACCTTTCTACCACTGCCCGATTTGCCCTTGAATCTGTCCCACCTGAAGTCATCGAAGGAGAAAATGCCTTTTTCCTTGTCCACAATCTGCCAGAGAATCTTGCAGCTGTAGTCTGGTCCAAAAGGGTGAAAAGTATGAACCATGGAATTGTAACATACGCACTGAACAAAGATTTAAGTGTGCCGGGGCCTCTACACAGTGGCAGAGAGACAGTGTACCGCAATGGATCCCTGCTGCTCAGAAATGTCACCAGGAAGGATACAGGACTTTATACCATAGAGCTCTTAGACAGACTTGGAGATATTGTGTCAACAATAACCGCTTATCTTCGCGTGCACA

>Ple_Psg3N1 (Peromyscus leucopus; white-footed mouse) WGS NMRJ02000002.1

CCTCCCTTTTAACTTTCTGGCACTTGTCCACCACTGCCGATGTGACCATTGAATCACTGCCACCCCTAGTGGCCGAAGGAGAAAATGTCCTTTTCCTTGTCCATGATCTGCCAGAAAATCTTATGGCCTTAGCCTGGTTCAAAGGACTAACAGATATGAACCAAGGAATTGCGGTATATACACTGCACAACAATTTAAGTGTGACAGGGTCTGTGCACAGTGGCAGAGAGACAATATATCACAATGGATCTCTGTTGCTGGAAAACGTCACCCAGAAGGACACAGGATACTACACTCTACGAACCTATAACAGACGTGCAAAAATCGTATCAACAACATCCATGTACCTCCAAGTGCACG

>Ple_Psg4N1 (Peromyscus leucopus; white-footed mouse) WGS NMRJ02000002.1

CCTCCTTATTAAACTTCTGGCACCTGTCCACCACTGCCCATGTGACCATTGAATCACTGCCACCCCAAGTGACTGAAGGAGAAAACGTCCTTTTCCTTGTTCATGGTCTGCCAAAGAATCTTATAGCCTTTGCCTGGTACAAAGGGCAAACAAATATGACACATGGAATTGCATGGTATACATTGGACAACAATTTACGTGGGCCAGGGCCTTTAAACAGTGGTAGAGAGACAGTGTATCGCAATGGATCCCTGCTGCTTCAAAATATCACCCAGAGGGACACAGGAACCTATACCCTACGAATCTATAATAGACGTGGAAAAATCATATCAACAACATCCATTAACCTCCATGTGACTG

>Ple_Psg5N1 (Peromyscus leucopus; white-footed mouse) WGS NMRJ02000002.1

CCTCCCTCTTAACCTCCTGGCACCTGTCCACCATGGCCCATGTGACTATTGAGTCACTTCCACTCCAAGTGGCTGAAGGAGAAAACGTCCTTTTCCTTGTCCATGATCTGCCAAAGAATCTTACAACCTTTGCCTGGTTTAAAGGGCTAATGAATATGACACAAAGAATTGCAATGTATACAATGGACAATACTGTAAGTGGGCCAGGACATGTGCACAGTGATAGAGAGACAATATATCATAATGGATCCCTGTTAATCGAAAATGTCACCCAGAAGGACACAGGAATCTATACCCTACGAACCTATAATAGACACGGAAAAACTGTATCTACAACATCCATGTACTTTCACGTGTCTG

>Ple_Psg6N1 (Peromyscus leucopus; white-footed mouse) WGS NMRJ02000002.1

CCTCCATGTTAACCTTCTGGCACCTGTCCACCACTGCCCATGTGACCATTGAATCACTGCCACCCCAAGTGACTGAAGGAGAAAGCGTTCTTTTCTTTGTCCATCGTCTGCCAAAGAATCTTAATGCCTTTGCCTGGTTAAAAAGGCTAAAAAATACGACACGAGGAATTGCATGGTATACATTGGACAATAATTTACGTGGGCCAGGGCCTGGGCACAGTGGTAGAGAGATAGTGTATCACAATGGATCCCTGCTGCTCCAAAATGTTACCCAGAAAGACACAGGAAGCTATATCCTACAAACCTATAATAGACGTAGAAAAATCATATCAACAACAACCATTTACCTCCATGTGCACG

>Ple_Psg7N1 (Peromyscus leucopus; white-footed mouse) WGS NMRJ02000002.1

CCTCCCTGTTAACCTTCTGGCTCCTGTCCATCACTGCCACTGTAAACACTGAATCAATGCCACTCCTTGTGGCTGAAGGAGAAAACATCCTTTTCCCTGTCCAAGATCTTCCAGAGAATATTATGGCCGTAGCCTGGTTCAAAGGACAAACAAAGATGACACAAGGAATTGCATTGTATGCACTGCATAGTGATTTAAGTTGCCCAGGTTCTGTGCACAGTGGTAGAGAGACAATATATCGCAACGGATCCCTGCTGCTAGAAAGGGTCACCCAGAATGACACAGGATTTTATACCCTGCGAACCTTTAATAGACACAGAAAAATCATGTCAGCAACATCCATTTACCTCCATGTGCATG

>Ple_Psg8N1 (Peromyscus leucopus; white-footed mouse) WGS NMRJ02000002.1

CCTCCCTTTTCACCTGCTGTCACCTGTCTACCACGGCCCAAGTCACCATTGAATCAGTGCCACCCCAAGTTGTTGAAGGAGAAAACGTTCTCCTCCGTGTTCATAATCTACCAGAGAATCTTCTAGCCTTTATCTGGCACAAAGGGGCAAGGAATATGAGCCTTAGAATTGCACTGTATTCACTGGCCAAGGATACATGTGTGAAAGGTCCCGTACACAGTGGTAGAGAGACAGTGTACAGCAACGGATCCCTGCAGATCCACAATGTCACCCAGAAGGACATAGGATTCTACACCTTACGAACCATTAATAGACGCATAGGAATTGCATCAATAACAACCAAGTACCTTCACGTGTACA

>Ple_Psg9N1 (Peromyscus leucopus; white-footed mouse) WGS NMRJ02000002.1

CCTCCCTGTTAACCTTCTGGCACCTGTCCACCACTGCCCATGTGACCATTGAATCACTGCCACCTGAAGTGGCTAAAGGAGAAAACGTCCTTTTCATTGTGCGTGATCTTCCAAAGAATATTATCGCCTTTGCTTGGTTCAAAGGGCAAACAAATATGACACAAGGAATTGCATGGTATACACTGGACAACAATTCACGTGGGCCAGGGCCTGTGCACAGTGGTAGAGAGACAGTGTATCGCAATGGATCCCTGCTGCTCCAAAAGGTCACCCAGAAGGACACAGGACCCTATACCCTACAAACTTTTAATAGACACAGAAAAATCATTTCAACAACATCCATTTACCTCCATGTGCATG

>Ple_Psg10N1 (Peromyscus leucopus; white-footed mouse) WGS NMRJ02000002.1

CCTCCATCTTAACCTCCTGGCACCTGTCCACCACTGCCCATGTGACCATTGAGTCACTTCCACTCCAAGTGATTGAGGGAGAAAACGTCCTTTTCGTTGTCCATGATCTCCCAGAGAATCTTGCAACCTTTGCCTGGTTCAAAGGTCTAACAAATATGACACAAAGAATTGCAATGTGTACAACGGACAATTTAAGTGGGCCAGGACATGTGCACAGTGGTAGAGAGACAATATATTGCAATGGATCCCTGTTGCTCAAAAATGTCAACCAGAAGGACACAGAAAATTATACCCTACAAACCTATAATAGACATGGAAAAATCATATCAACAACATCCAAGTACCTCCATGTGTCTG

>Ple_Psg11N1 (Peromyscus leucopus; white-footed mouse) WGS NMRJ02000002.1

CCTGCCTTTTAACCTCCTGGCACCTGTCCACCACTGCCCATGTGAGCACTAAAACAGTGCCACCCCAAGTGGCTGAAGGAGAAAACGTCCTTTTCGAGGTTCATGGTCTTCCAGAGAATACTATGGGCTTTGCCTGGTTCAAAGAGCTAAGAAATATGAAAAAAGCAATTGCAGTATATGGAATGCACATCAATTTAAGTGCACCAGGGCCTGTGCACAGTGGTAGAGAAACATTATATCGCAATGGATCCATGCTGCTTGAAAAAGTCACCAAGAAGGACATAGGATTTTATACCCTGCGGACCTATGACAGAAATGTAAAAATCGTATCAACAACATCCACGTACCTCCATGTGGACA

>Ple_Psg12N1 (Peromyscus leucopus; white-footed mouse) WGS NMRJ02000002.1

CCTCCCTTTTAAGCTGCTGGTATCCACCCACCACTGCCCAAGTCAACATTGAACTCCTGCCGCCCAATGTGTTCGAAGGAGACAATGTCCTTCTACAGGTCCACAATCTGCCAGAGAATCTGCTAGCCTTTGCTTGGTACAAAGGCGTGACAAATTTGAAACGTGGAATTGCCATCTATTCACTGAAATACAATTTAAGTGTCACAGGGCCTGTACACAGTGGAAGAGAGACAGTGTACAGCAATGGATCCCTGCTGCTCCAGAGTGTCACCCACAAGGACACAGGATTCTACACCCTACGAACCATAAGTAGACAAGCAGAAATTGTATCAGTGACATCCATGTACGTCCACGTGCACA

>Ple_Psg13N1 (Peromyscus leucopus; white-footed mouse) WGS NMRJ02000002.1

CCTCCTTTTTAACCTCTTGGCACCTGACCACCACTGCCCAAGTCACCATTGAATTAGTGCCACCCCAAGTGGTTGAAGGAGAAAATGTCATTTTCCTTGTCCGTAATCTTCCAGAGAAGCTTGTGGGCTTATCCTGGTTTAAAGAGGAGACAAATATGAAGTCTAAAATTGCAAGCTATGAAATGGCCTACAGTCGGGATTTTCTGGGGGCAGCACATACTGGTAGAGAGACAGTATACCCTAATGGATCCCTGTGGATTCAAAATGTTACCCAGAGTGACTCAGGAGTCTATATCCTACGAAGCGGTAATAGAGTAAGAATTACATCATCAACATACATATACCTTCACGTGTACA

>Ple_Psg14N1 (Peromyscus leucopus; white-footed mouse) WGS NMRJ02000002.1

TCTCCCTTTTAACCTGTTGGTACCGGCCCACCACTGCCCAAGTCACCATTGAATCAGTGCCTCCCCATGTGGTCGAAGGAGAAAGCAACCTTTTCCTTGTCCACAATCTGCCAGAGAATCTTTTAACCATATCCTGGTTTAAAGAAGAAGCAAATATGGATCATAAAATTATGACCTACACACTGAAATACAATATTGCTCTGCCAGGGCCAGCACACAGTGGTAGAGAGACAGTGTACCCAAATGGATCCCTGTGGATTCAAAATGTCACCCACAAGGACACAGGATTCTATATACTAGAAACCAGAAAAGTAAAAATTATATCAACAATATACATACACCTTCACGTGTACA

>Ple_Psg15N1 (Peromyscus leucopus; white-footed mouse) WGS NMRJ02000002.1

CCTCGCTTTTAACCTGTTGGTACCGCCTCACCACTGCCCAAGTCATCATTGCATCAGTGCCTCTCCATGTGGTCGAAGGAGAAAGCAACCTTTTTCTTGTTCACAATCTTCCAGACAATCTTTTAACCATATCCTGGTTTAAAAAAAGAGCAAATATGGACCATAAAATTGCGACCTACACACTGAAATACAATATTGCTCTGCCGGGGCCAGCACACAGTGGTAGAGAGACAGTGTACGCAAATGGATCCCTGTGGATTCAAAATGTCACCCATAAGGACACAGGAATCTACATACTACAAACCAGAAGTAGACAAGTAAAAACTGTATCAACAAAACATACATACCTTCATGTGTACA

>Ple_Psg16N1 (Peromyscus leucopus; white-footed mouse) WGS NMRJ02000002.1

CCTCCCTTTTAACCTGTTGGTACCGGCCCACCACTGCCCAAGTCACCATTGAATCAGTGCCTCCCCATGTGGTCGAAGGAGAAAGCAACCTTTTCCTTGTCCACAATCTGCCAGAGAATCTTTTATCCATATCCTGGTTTAAAGAGGGAGCAAATATGGACCACAGAATTGTGACCTACATACTGAATTACAACACAGCTCTGCCGGGAACAGCACACAGTGGTAGAGAGACAATGTACCCAAATGGATCCCTGTGGATTCAAAATGTCACCCATAAGGACACAGGATTCTATATACTACAAACCATAAGTACAGAAGTAAAAACTGTATCAAGAACATACTTACACCTTCATGTGTACA

>Ple_Psg17N1 (Peromyscus leucopus; white-footed mouse) WGS NMRJ02000002.1

CCTCCCTTTTCACCTGCTGGCATCTGTCCACCACTGCCCAAGTCACCATTGAATCAGTGCCGCCCCAAGTGGTTGAAGGAGAAAACGTCCTTCTACATGTCAACAATCTGCCAGAGAATCTCCTAGCTTTCTCCTGGTACAAGGAGGTGAGGAATATGAACCTCAGAATTGCACTATTTGCACTGAACACAAATCAAATTGTGATGGGGCCTGAACACACTGACAGAGAAACAGTGTACAGCAATGGATCCCTGTGGCTGAAAAATGTCACCAAGAAGGACACAGGATTCTATACCCTACAAACAGTGAATAGAGCTGGAAAAATTGTGTCTACAACAACCATGTACTTCCATGTGTAC

>Ple_Psg18N1 (Peromyscus leucopus; white-footed mouse) WGS NMRJ02000002.1

CCTCCATTTTCATTTGCTGGCACCTTTCAACCACTGATGATTTTACCATGGAACCTGTCCCACCCCATGTGGCCAATGGAGACAATGTACTTCTTCTTGTCCACAATCTGCCCGAGAATATTATAGCCTTCACCTGGATCAAAGGGGAGATAAGCATGAATCATACAATTGCAATATATATACCAAACAAAAAGTTAAGTGTGCCAGGGCGTTTATATAGTGGTAGAGAGACAGTGTATGGCAATGGATCTCTGCTCCTCCAAAATGTCAATGAGAAGGACACAGGAATTTATACCCTACAAACCTTTAACAGACACACAGATACTGTGTCGCAAACATCCATGTACCTCTATGTTCACA

>Ple_Psg19N1 (Peromyscus leucopus; white-footed mouse) WGS NMRJ02000002.1

CCTCCCTTTTAATCTGCTGTCATTTAGCCACCACTGCTAAAGTCACCATTGAATCAGTGCCACTCAATGTGTTCGAAGGAGACAATGTCCTTCTACATGTCCACAATCTGCCAGAGAATCTTCTAGCCTTTGCTTGGTTCAAAAGGCTGACAAAAACGAAACACAGAATTGCACTCTATGCACTGAACATCAATTTATTTGTGCCAGGGCCTGTACACGTTGGTAGAGAGACAGTGTACCGCAACGGATCCCTGTGGATTCAGAATGTCACCCATAAGGACACAGGATTCTACACCCTAGAGACCATAAATAGACATGGACGAACTGTATCAGTAACAACCATGTACCTCCATGTGTACA

>Ple_Psg20N1 (Peromyscus leucopus; white-footed mouse) WGS NMRJ02000002.1

nctctcTTTTAATTTACTGCCATTCCCCAACCAATGCTGAAGTCACCATTGAATCAGTGCCACTTGATGTGTTCGAAGGAGACAATGTCCTTCTACATGTCCACAATCTGCCAGAGAGTCTTCTATCCTTTGTTTGGTTCAAAGGGCTGACAAATATGAAGCACAGAATTGCGCTCTATGTACTGAACACCAATTTAATTGTGCCGGGGCCTGTGCACAGTGGTAGAGAGACAGTGTACCACAATGGATCCCTGTGGATTCACAATGTCACCCATAAGGACACAGGATTCTACACCCTACAGACCATAAATAGACATGGACGAACTGTATCAATAACAACTATGCACCTCCATGTGCACC

>Ple_Psg21N1 (Peromyscus leucopus; white-footed mouse) WGS NMRJ02000002.1

CCTCCTTTTTAACCTTTTGGCACCTGTCTACAACTGACCATGTGACCATTGAATCACTGCCATCCCAAGTGGCTGAAGGAGAAAATGTCCTTTTCCTTGTCCAGAATCTGCCAGAGAATCTTACAGCCTTTGCTTGGTTCAAAGGGCTCAGATGTATGAAACGAAGAATTTCAATGTATACACTGCACAACAATTTAAGTGGTCCAGGGCCTGTGCACAGCGGTAGAGAGACAATATCTCACAATGGATCCCTGCTGCTCGAAAAGGCCACCCAGAAGGACACAGGATACTATATGCTAAGAACATGTAACAGACAGGGTAAAATCATGTCAACAACATCCATGTACCTCCACGTGCATG

>Ple_Psg23N1 (Peromyscus leucopus; white-footed mouse) WGS NMRJ02000002.1

CCTCCATTTTAACCTGTTGGTACCTACCCACCACTGTCCAAGTCATTATTGAATTAGTGCCACCTGATGTGGTTGAAGGAGACAATGTCCTTCTCCTTGTCCGCAATCTGCCAGAGAATCTTGAAGCCTTTGTCTGGTACAAAGGGGTGACAAATATGAACCTTGGAATTGTACTTTATTCGATGGACACTAATTTAAGAGTGGCAGGGCCTGAATACAGTGGTAGAGAGACAGTGTACAGAAATGGATCCCTGCATCTCCAAGATGTCACCCAGAAGGACACTGGATTCTATACCCTACGATCCATAAACAGACATAAAGAAATCATATCAACAACATCCATATACCTCCACGTGTACT

>Ple_Psg24N1 (Peromyscus leucopus; white-footed mouse) WGS NMRJ02000002.1

CCTCCATTTTAACCTGCTGGCACCTTTCCACCACTGATCGTTTTGCTGTGCAAACTGTCCCACTCTATGTGGCCAACGGAGACAGCATCCTTTTCCTTGTCCGCAATCTGCCAAAGAATCTTCTATCCTTTGCCTGGATCAAAGGGAGAACCAGCATGAATGATGCAATCATAGTATATATACCAAACATAAATTTAAGTGTGCCAGGGCGTTTTCACAGTGGTAGAGAGACAGTGTATGGCAATGGATCCATGTTCATCCAAAATGTCAACCAGAAGGACACAGGATTCTATTCTCTACTAGCTTTTCATAGACACACAGATACTGTGTCACAAATAGGCACATACGTCTACGTGAAGA

>Pma_Ceacam9N (Peromyscus maniculatus bairdii; Northern American deer mouse) WGS RCWR01130653.1

CCTCCCTCTTGACCTGCTGGAATGCACCCACTACAGCCGAGCTCACTATTGAATTAGTGCCTCCCATGGTGGCTGAGGGTGGAAACTCCGTTCTCTTTGTGCACAAAATGCCGTTGAACGTCCAGGCGTTTTACTGGTACAAACAGAAAGACCCGACGAAGAGCTATGAAGTCGCCCGGTACTTAACACCCGACAACACAACTTCGAAGATGCCTCAACAGAGCGGCAGGAAAACGGTGTTCTACAGTGGATCCCTGCTGATCAGAAACGTCACCCAGGCTGACAGCGGATTCTACACCTTACTCACATTTAACACGGAAATGGAAAGTGAACTAACACACGTGTATCTGGAAGTCCACA

>Pma_Ceacam11N1 (Peromyscus maniculatus bairdii; Northern American deer mouse) WGS RCWR01116439.1

CCTCCCTTTTAACCTGCTGGCTGCCTCCTACTGTTGCCCAGCTCACCATTGAATCAGTGCCACCCATTTCTGCTGAAGGGGATAATGTTCTTCTGCTTGTGCACAACCTTCCTGAGAATGGTCAAGCCTTTTCCTGGTACACAGGAGTTATGGTGCTCAAGAGCCGTGAAATTGCAAGATGTGCAATAGCTACCAATTCATGTGTGCTGGGTCCTGCACACAGTGGTAGAGAGACAGCATTCAATAATGGATCTCTGCTGATCAAGAATGTCACCAGGAAGGACTCAGGATACTACATCCTACAGACACTTAATACAAATTCAAGATCTGAAATAACACGTGCGGAATTTTTTGTACACA

>Pma_Ceacam15N (Peromyscus maniculatus bairdii; Northern American deer mouse) WGS RCWR01130661.1

CCTCACTTTTAACCTGCTGGAACTCACCCACTGCGGCGCTAAGATCTACCAAAGAAATGCGGTTTTCTGCAGCTGAAGGGGGGAAGGTTCTTCTCTCTGTTCCTATCCAGGCAGAGAATCTTCTCTCCTTTCACTGGTACAAAGGGAAAGAAGAAGACCAAGATTTTGCAATTGCCCATTATGAAAAGGACACAGATTCACTTAAATTTGGGAATGCAACCAGCGGCAGGGAAGAGGTATATAAGGATGGGTCCATGATGCTCCAGGATGTCACCCAGGAAGATACAGGGATCTACACCCTAGAAGCCATTGGAACACATGATCATATTGAAATAACACATTTCTACCTCCAGGTGTACA

>Pma_Psg1N1 (Peromyscus maniculatus bairdii; Northern American deer mouse) Ensembl ENSPEMG00000026361

CCTCCATTTTCACCTGCTGGCACCTTTCCACCACTGACCATATCACCATTAAATCTTTCCCGTCTCAAGTGGCCAATGGAGACAACGTCCTTCTTCTTGTCAACGATTTGCCCGAAGATCTTCTAACCTTTACCTGGTTTAAAGGCGAGACAGGCATGGACCTTGGAATTGCAAGATATGCACCAGACAGAGATTTAATCATGCAAGGGCCTGGATATAGTGGTAGAGAGACAGTGTACCGCAATGGATCCCTGCTGATCCAAAATGTCAATGAGAAGGACACAGGACTCTACACTCTGCAAACCTTAAATGAACATGGAGATGTTCTGTCAATAACAACTATGCGCCTGCATGTGTACC

>Pma_Psg2N1 (Peromyscus maniculatus bairdii; Northern American deer mouse) Ensembl ENSPEMG00000028933

CCTCCCTTTTATCGTGCTGGCACCTTTCCACCACTGCCCGATTTGCCCTTGAATCTGTCCCACCTGAAGTCATCGAAGGAGAAAATGCCTTTTTCCTTGTCCACAATCTGCCAGAGAATCTTGCAGCTGTAGTCTGGTCCAAAAGGGTGAAAAGTATGAACCATGGAATTGTGACATACGCACTGAACAAAGATTTAAGTGTGCCGGGGCCTCTACACAGTGGCAGAGAGACAGTGTACCGCAATGGATCCCTGCTGCTCAGAAACGTCACCAGGAAGGATACAGGACTTTATACCATAGAGCTCTTAGACAGGCTTGGAGATATTGTGTCAACAATAACCGCTTATCTTCGCGTGCACA

>Pma_Psg3N1 (Peromyscus maniculatus bairdii; Northern American deer mouse) Ensembl ENSPEMG00000025341

CCTCCCTTTTAACCTTCTGGCACCTGTCCACCACTGCGGATGTGACCATTGAATCACTGCCACCCCTAGTGGCCGAAGGAGAAAATGTCCTTTTCCTTGTCCGTGATCTGCCAGAAAATCTTATGGCCTTAGCCTGGTTCAAAGGACTAACAGATATGAAACAAGGAATTGCGGTATATACACTGCACAACAATTTAAGTGTGACAGGGTCTGTGCACAGTGGCAGAGAGACAATATATCACAATGGATCTCTGTTGCTGGAAAACGTCACCCAGAAGGACACAGGATACTACACTCTACGAACCTATAACAGACGTGCAAAAATCGTATCAACAACATCCATGTACCTCCAAGTGCACG

>Pma_Psg4N1 (Peromyscus maniculatus bairdii; Northern American deer mouse) Ensembl ENSPEMG00000026401

CCTCCCTATTAACCTTCTGGCACCGGTCCACCACTGCCCATGTGACCATTGAATCACTGCCACCCCAAGTGGCTGAAGGAGAAAACGTCCTTTTCCTTGTCCATGGTCTGCCAAAGAATCTTATAGCCTTTGCCTGGTACAAAGGGCAAACAAATATGACACACGGAATTGCATGGTATACACTGGACAACAATTTACGTGGACCAGGGCCTTTAAACAGTGGTAGAGAGACAGTGTATCGCAATGGATCCCTGCTGCTTCAAAATATCACCCAGAGGGACACAGGAACCTATACCCTACAAATCTATAATAGACGTGGAAAAATCATATCAGCAACATCCATTAACCTCCATGTGACTG

>Pma_Psg5N1 (Peromyscus maniculatus bairdii; Northern American deer mouse) Ensembl ENSPEMG00000026163

CCTCCCTCTTAACCTCCTGGCACCTGTCCACCATGGCCCATGTGACTATTGAGTCACTTCCACTCCAAGTGGCTGAAGGAGAAAACGTCCTTTTCCTTGTCCATGATCTGCCAAAGAATCTTACAACCTTTGCCTGGTTTAAAGGGCTAACAAATATGACACAAAGAATTGCAATGTATACAATGGACAATACTGTAAGTGGGCCAGGACATGTGCACAGTGATAGAGAGACAATATATCATAATGGATCCCTGTTAATCGAAAATGTCACCCAGAAGGACACAGGAATCTATACCCTACGAACCTATAATAGACATGGAAAAACTGTATCAACAACATCCACGTACTTTCACGTGTCTG

>Pma_Psg6N1 (Peromyscus maniculatus bairdii; Northern American deer mouse) WGS RCWR01116426.1

CCTCCCTGTTAAACTTCTGGCACCTGTCCACCACTGCCCATGTGACCATTGAATCACTGCCACCCCAAGTGACTGAAGGAGAAAGCGTTCTTTTCTTTGTCCATCGTCTGCCAAAGAATCTTAATGCCTTTGCCTGGTTAAAAAGACTAACAAATACGACACGAGGAATTGCATGGTATACATTGGACAATAATTTACGTGGGCCAGGGCCTGGGCACAGTGGTAGAGAGATAGTGTATCACAATGGATCCCTGCTGCTCCAAAATGTTACCCAGAAGGACACAGGAAGCTATATCCTACAAACCTATAATAGACGTAGAAAAATCATATCAACAACAACCATTTACCTCCATGTGCACG

>Pma_Psg7N1 (Peromyscus maniculatus bairdii; Northern American deer mouse) Ensembl ENSPEMG00000025653.1

CCTCCCTGTTAACCTTCTGGCTCCTGTCCACCACTGCCACTGTAAACACTGAATCAATGCCACTCCTTGTGGCTGAAGGAGAAAACATTCTTTTCCCTGTCCAAGATCTTCCAGAGAATATTATGGCCATAGCCTGGTTCAAAGGACGAACAAAGATGACACAAGGAATTGCATTGTATGCACTGCACAGTGATTTAAGTTGCCCAGGTTCTGTGCACAGTGGTAGAGAGACAATATATCGCAATGGATCCCTGCTGCTAGAAAGGGTCACCCAGAATGACACAGGATTTTATACCCTGCGAACCTTTAACAGACACAGGAAAATCATGTCAGCAACATCCATTTACCTCCATGTGCATG

>Pma_Psg8N1 (Peromyscus maniculatus bairdii; Northern American deer mouse) WGS RCWR01116484.1

CCTCCCTTTTCACCTGCTGTCACCTGTCCACCACGGCCCAAGTCACCATTGAATCAGTGCCACCCCAAGTTGTTGAAGGAGAAAACGTCCTCCTCCGTGTTCATAATCTACCAGAGAATCTTCTAGCCTTTATCTGGCACAAAGGGGCAAGGGATATGAGCCTTAGAATTGCACTGTATTCACTGGCCAAGGATACAAGTGTGAATGGTCCCGTACACAGTGGCAGAGAGACAGTGTACAGGAACGGATCCCTGCTGATCCACAATGTCACCCAGAAGGACACAGGATTCTATACCTTACGAACCATTAATAGACGCATAGGAATTGCATCAATAACAACCAAGTACCTTCACGTGTACA

>Pma_Psg9N1 (Peromyscus maniculatus bairdii; Northern American deer mouse) Ensembl ENSPEMG00000025954

CCTCCCTGTTAACCTTCTGGCACCTGTCCACCACTGCCCATGTGACCATTGAATCACTGCCACCTGAAGTGGTTGAAGGAGAAAACGTCCTTTTCATTGTGCGTGATCTTCCAAAGAATATTATCGCCTTTGCTTGGTTCAAAGGGCAAATAAATATGACACAAGGAATTGCATGGTATACATTGGACAACAATTCACGTGGGCCAGGGCCTGTGCACAGTGGTAGAGAGACAGTGTATCGCAATGGATCCCTGCTGTTCCAAAAGGTTACCCAGAAGGACACAGGACCCTATACCCTACAAACTTTTAATAGACACAGAAAAATCATTTCAACAACATCCATTTACCTCCATGTGCATG

>Pma_Psg10N1 (Peromyscus maniculatus bairdii; Northern American deer mouse) Ensembl ENSPEMG00000024336

CCTCCATCTTAACCTCCTGGCACCTGTCCACCACTGCCCATGTGACCATTGAGTCACTTCCACTCCAAGTGATTGAGGGAGAAAACGTCCTTTTTGTTGTCCATGATCTCCCAGAGAATCTTGCAACCTTTGCCTGGTTCAAAGGTCTAACAAATATGACACAAAGAATTGCAATGTGTACAACGGACAATTTAAGTGGGCCAGGACATGTGCACAGCGGTAGAGAGACAATATATTGCAATGGATCCCTGTTGCTCAAAAATGTCAACCAGAAGGATACAGAAAATTATACCCTACAAACCTATAATAGACATGGAAAAATCATATCAACAACATCCAAGTACCTCCATGTGTCTG

>Pma_Psg11N1 (Peromyscus maniculatus bairdii; Northern American deer mouse) WGS RCWR01116432.1

CCTGCCTTTTAACCTCCTGGCACCTGTCCACCACTGCCCATGTGAGCACTAAAACAGTGCCACCCCAAGTGGCTGAAGGAGAAAACGTCCTTTTTGATGTTCATGGTCTTCCAGAGAATATTATAGGCTTTGCCTGGTTCAAAGAGCTAAGAAATATGAAAAAAGCAATTGCAGTATATGGAATGCACATCAATTTAAGTGCACCAGGGCCTGTGCACAGTGGTAGAGAAACATTATATCGCAATGGATCCATGCTGCTTGAAAAAGTCACCAAGAAGGACATAGGATTTTATACCCTGCGGACCTATGACAGAAATGTAAAAATCGTATCAACAACATCCACGTACCTCCATGTGGACA

>Pma_Psg12N1 (Peromyscus maniculatus bairdii; Northern American deer mouse) WGS RCWR01116389.1

CCTCCCTTTTAAGCTGCTGGTATCAACCCACCACTGCCCAAATCACCATTGAACTGCCGCCCAATGTGTTCGAAGGAGACAATGTCCTTCTACAGGTCCACAATCTGCCAGATAATCTGCTAGCCTTTGCTTGGTACAAAGGCGTGACAAATTTGAAACGTGGAATTGCCATCTATTCACTGAAATACAATTTAAGTGTAACAGGGCCTGTACACAGCGGAAGAGAGACAGTGTACAGCAATGGATCCCTGCTGCTCCAGAGTGTCACCCACAAGGACACAGGATTCTACACCTTTCGAACCATAAGTAGACAAGCAGAAATTGTATCAGTGACATCCATGTACCTCCACGTGCACA

>Pma_Psg13N1 (Peromyscus maniculatus bairdii; Northern American deer mouse) WGS RCWR01116389.1

CCTCCTTTTTAACCTCTTGGCACCTGACCACCACTGCCCAAGTCACCATTGAATTAGTGCCACCCCAAGTGGTTGAAGGAGAAAATGTCATTTTCCTTGTCCGTAATCTTCCAGAGAAGATTGTGGGCTTATCCTGGTTTAAAGAGGAGACAAATATGAAGTCTAAAATTGCAAGCTATGAAATGGCCTACAGTCGGGATTTTCTGGGGGCAGCACATACTGGTAGAGAGACAGTGTACCCTAATGGATCCCTGTGGATTCAAAATGTTACCCAGAGTGACTCAGGAGTCTATATCCTACGAAGCGGTAATAGAGTAAGAATTACATCATCAACATACATATACCTTCACGTGTACA

>Pma_Psg14N1 (Peromyscus maniculatus bairdii; Northern American deer mouse) WGS RCWR01116390.1

TCTCCCTTTTAACTTGTTGGTACCGGCCCACCACTGCCCAAGTCACCATTGAATCAGTGCCTCCCCATGTGGTTGAAGGAGAAAGCAACCTTTTTCTTGTCCACAATCTGCCAGACAATCTTTTAACCATATCATGGTTTAAAGAAGAAGCAAATATGGACCATAAAATTACAACCTACACACTGAAATACAATATTGCTCTGCCGGGGCCAGCACACAGTGGTAGAGAGACAGTGTACCCAAATGGATCCCTGTGGATTCAAAATGTCACCCACAAGGACACAGGATTCTATATACTAGAAACCAGAAAAGTAAAAATTATATCAACAATATACATATACCTTCACGTGTACA

>Pma_Psg15N1 (Peromyscus maniculatus bairdii; Northern American deer mouse) WGS RCWR01116392.1

CCTCCCTTTTAGCCTGTTGGTACCACCTCACCACTGCCCAAGTCATCATTGCATCAGTGCCTCTCCATGTGGTCGAAGGAGAAAGCAACCTTTTTCTTGTTCACAATCTTCCAGACAATCTTTTAACCATATCCTGGTTTAAAAAAAGAGCAAATATGGACCATAAAATTGCGACCTACACACTGAAATACAATATAGCTGTGCCGGGGGCAGCACACAGTGGTAGAGAGACTGTGTACCCAAATGGATCCCTGTGGATTCAAAATGTCACCCATAAGGACACAGGAATCTACATACTACAAACCAGAAGTAGACAAGTAAAAATTGTATCAACAAAACATACATACCTTCATGTGTACA

>Pma_Psg16N1 (Peromyscus maniculatus bairdii; Northern American deer mouse) WGS RCWR01116392.1

CCTCCTTTTTACTCTGTTGGTACCAGCCCACCACTGCCCAAGTCACCATTGAATCAGTGCCTCCCCATGTGGTCGAAGGAGAAAGCAACCTTTTCCTTGTCCACAATCTGCCAGAGGATCTTTTATCCATATCCTGGTTTAAAGAGGGAGCAAATATGGACCACAGAATTGCAACCTATATACTGGAATACAACATAGCTCTGCCTGGAACAGCACACAGTGGTAGAGAGACAGTGTACCCAAATGGATCCCTGTGGATTCAAAATGTCACCCATAAGGACACAGGATTCTATATACTACAAACCATAAGTGGAGAAGTAAAAACTGTATCAAGAACATACATACACCTTCATGTGTACA

>Pma_Psg17N1 (Peromyscus maniculatus bairdii; Northern American deer mouse) Ensembl ENSPEMG00000008362

CCTCCCTTTTCACCTGCTGGCATCTGTCCACCACTGCCCAAGTCACCATTGAATCAGTGCCGCCCCAAGTGGTTGAAGGAGAAAACGTCCTTCTACGTGCCAACAATCTGCCAGAGAATCTTCTAGCTTTCTCCTGGTACAAGGAGGTGAGGAATGTGAACCTCAGAATTGCACTATTTGCACTGAACAAGAATCAAATTGTGATGGGGCCTCAACACAGTGACAGAGAAACAGTGTACAGCAATGGATCCCTGTGGCTGAAAAATGTCACCAAGAAGGACACAGGATTCTATACCCTACAAACAGTGAATAGAGCTGGAAAAATTGTATCTACAACAACCATGTACTTCCATGTGTACA

>Pma_Psg18N1 (Peromyscus maniculatus bairdii; Northern American deer mouse) WGS AYHN01025361.1

CCTCCATTTTCATTTGCTGGCACCTTTCAACCACTGATGATTTTACCATGGAATCTGTCCCACCCCATGTGGCCAATGGAGACAATGTACTTCTTCTTGTCCACAATCTGCCCAAGAATATTATAGCCTTCGCCTGGATCAAAGGGGAGATAAGCATGAATCATACAATTGCAATATATATACCAAACAAAAAGTTAAGTGTGCCAGGGCATTTATATAGTGGTAGAGAGACAGTGTATGGCAATGGATCCCTGCTCCTCCAAAATGTCAATGAGAAGGACACAGGAATTTATACCCTACAAACCTTTAATAGACACACAGATACTGTGTCGCAAACATCCATGTACCTCTATGTTCACA

>Pma_Psg19N1 (Peromyscus maniculatus bairdii; Northern American deer mouse) WGS RCWR01152821.1

CCTCCCTTTTAATCTGCTGTCATTTAGCCACCACTGCTAAAGTCACCATTGAATCAGTGCCACTCAATGTGTTCGAAGGAGACAATGTCCTTCTACATGTCCACAATCTGCCAGAGAATCTTCTAGCCTTTGCTTGGTTCAAAAGGCTGACAAAAACGAAACACAGAATTGCACTCTATGCACTGAACATCAATTTATTTGTGCCAGGGCCTGCACATGTTGGTAGAGAGACAGTGTACCGCAACGGATCCCTGTGGATTCAGAATGTCACCCATAAGGACACAGGATTCTATACCCTAGAGACCATAAATAGACATGGACGAACTGTATCAATAACAACCATGTACCTCCATGTGTACA

>Pma_Psg20N1 (Peromyscus maniculatus bairdii; Northern American deer mouse) WGS RCWR01116388.1

CCTCCGTTTTAATTTACTGCCATTCCCCAACCAATGCTGAAGTCACCATTGAATCAGTGCCACTTGATGTGTTCGAAGGAGACAATGTCCTTCTACATGTCCACAATCTGCCAGAGAGTCTTCTATCCTTTGTTTGGTTCAAAGGGCTGACAAATATGAAGCACAGAATTGCGCTCTATGTACTGAACACCAATTTAATTGTGCCGGGGCCTGTACACAGTGGTAGAGAGACAATGTACCGCAATGGATCCCTGTGGATTCACAATGTCACCCATAAGGACACAGGATTCTACACCCTGCAGACCATAAATAGACATGGACGAACTGTATCAATAACAACTATGCACCTCCATGTGCACC

>Pma_Psg21N1_P (Peromyscus maniculatus bairdii; Northern American deer mouse) WGS RCWR01116484.1

CCTCCTTTTTAACCTTTTGGCACCTGTCTACAACTGACCATGTGACCATTGAATCACTGCCATCCCAAGTGGCTGAAGGAGAAAATGTTCTTTTCCTTGTCCAGAATCTGCCAGAGAATCTTACAGCCTTTGCTTGGTTCAAAGGGCTCAGATGTATGAAATGAAGAATTTCAATGTATACACTGCACAACAATTTAAGTGGTCCAGGGCCTGTGCACAGAAGTAGAGAGACAATATCTCACAATGGATCCCTGCTGCTCGAAAAAGTCACCCAGAAGGACACCGGATACTATATGCTAAGAACATGTAACAGATGGGGAAAAATCATGTCAACAACATCCATGTACCTCCACGTGCATG

>Pma_Psg22N1 (Peromyscus maniculatus bairdii; Northern American deer mouse) WGS RCWR01116437.1

CCTCCCTTTTAACCTGTTGGCACCTACCCACCACTGTCCAAGTCATTATTGAATTAGTGCCACCTGATGTGGTTGAAGGAGAAAATGTCCTTCTCCTTGTCCGCAATCTGCCAGAGAATCTTGAAGCCTTTGTCTGGTACAAAGGGGTGACAAATATGAACCTTGGAATTGTACTTTATTCGCTGGACACTAATTTAAGAGTGGAAGGGCCTGAATACAGTGGTAGGGAGACAGTGTACAGAAATGGATCCCTGCATCTCCAAGATGTCACCCAGAAGGACACTGGATTTTATACCCTACGATCAATAAACAGACATAAAGAAATCATATCAACAACATCCATATACCTCCACGTGTACT

>Pma_Psg23N1 (Peromyscus maniculatus bairdii; Northern American deer mouse) WGS RCWR01116409.1

CCTCCATTTTAACCTGCTGGCACCTTTCCACCACTGATCGTTTTGCTGTGCAAACTGTCCCACTCTATGTGGCCAACGGAGACAGCATCCTTTTCCTTGTCCGCAATCTGCCAAAGAATCTTCTATCCTTCGCCTGGATCAAAGGGAAAACCAGCATGAATGATGCAATCATAGTATATGTACCAAACATAAATTTAACTGTGCCAGGGCGTTTTCACAGTGGTAGAGAGACAGTGTATGGAAATGGATCCCTGCTCATCCAAAATGTCAACCAGAAGGACACAGGATTCTATTCTCTACTAGCTTTTCATAGACACACAGATACTGTGTCACAAATAGGCACATACCTCTACGTGAACA

>Pma_Psg24N1 (Peromyscus maniculatus bairdii; Northern American deer mouse) WGS RCWR01116401.1

CCTCCATTTTAACCTGCTGGCACCTTTCCACCACTGATCATTTTGGTGTGGAAGTTGTCCCACCCTATGTGGCCAATGGAGACAGCGTCCTTTTCCTTGTCCGCAATCTGCCAAAGAATCTTCTATCCTTCGCCTGGATCAAAGGGAAAACCAGCATGAATGATGCAATCATAGTATATGTACCAAACATAAATTTAAGTGTGCCAGGGCGTTTTCACAGTGGTAGAGAGACAGTGTATGGAAATGGATCCATGCTCATCCAAAATGTCAACCAGAAGGACACAGGATTCTATTCTCTACTAGCTTTTCATAGACACACAGATACTGTGTCACAAATAGGCACATACCTCTACGTGAACA

>Pma_Psg25N1 (Peromyscus maniculatus bairdii; Northern American deer mouse) WGS AYHN01025347.1

CCTCCATTTTAACCTGCTGGCACCTTTCCACCACTGATCATTTTGGTGTGGAAGTTGTCCCACCCTATGTGGCCAATGGAGACAGCGTCCTTTTCCTTGTCCGCAATCTGCCAAAGAATCTTCTATCCTTTGCCTGGATCAAAGGGGGAACGAGCATGAATGATGCAATCATAGTATATATACCAAACAATAACTTAAGTGTGCCAGGGCGTTTTCACAGTGGTAGAGAGACAGTGTATGGAAATGGATCCCTGCTCATCCAAAATGTCAACCAGAAGGACACAGGAGTCTATTCTCTACGAGCTTTTAATAGACGCACAGATACTGTGTCACAAATAGGCACATACCTCTACGTGAACA

>Pme_Ceacam9N (Peromyscus melanophrys; plateau mouse) WGS CABHPR010089314.1

CCTCCCTCTTGACCTTCTGGAATGCACCTACTACTGCCGAGCTCACTATTGAATTAGTGCCTCCCATGGTAGCTGAAGGTGGAAACTCCGTTCTATTTGTGCACAAAATGCCGTTGAACGTCCAGGCATTTTACTGGTACAAACAGAAAGGTCCAACAAAGAGCTATGAAGTTGCCCGGTACTTAACACCTGATAACACAACTTCGAAGATGCCTCAACACAGCGGCAGGAAAACGGTGTTCTACAGTGGATCCCTGCTGATCAGAAACGTCACCCAGGCTGACAGCGGATTCTACACCTTACTAACATTTAACACAGAAATGGAAAGTGAACTAACACACGTGTATCTGGAAGTCCACA

>Pme_Ceacam11N1 (Peromyscus melanophrys; plateau mouse) WGS CABHPR010118185.1 CCTCCCTTTTAACCTGCTGGCTGCCTCCTACTGTTGCCCAGCTCACCATTGAATCAGTGCCACCCATTTCTGCTGAAGGGGATAATGTTCTTCTGCTTGTGCACAACCTTCCTGAGAATGTTCAAGCCTTTTCCTGGTACACAGGAGTTATGGTGCTCAAGAGCCGTGAAATTGCAAGATGTGCAATAGCTACCAATTCATGTGTGCTGGGGCCTGCACACAGTGGTAGAGAGACAGCATTCAATAATGGATCTCTGCTGATCGAGAATGTCACCAGGAAGGACTCAGGATACTACATCCTACAAACACTTAATACAAATTCGAGATCTGAAATAACACGTGCAGAATTTTTTGTACACA

>Pme_Ceacam15N (Peromyscus melanophrys; plateau mouse) WGS CABHPR010157688.1

CCTCACTTTTAACCTGCTGGAACTCACCCACTGCGGCGCTAAGAACTACCAAAGAAATGAGGTTCTCTGCGGCTGAAGGGGGGAAGGTTCTTCTCTCTGTTCCTATTCAGGCAGAGAATCTCCTCTCCTTTCGCTGGTACAAAGGGAAAGAAGAAGACCAAGATTTTACAATTGCCCATTATGAAAAGGACACAGATTTACTTAAACTTGGGAATGCAACCAGCGGCAGGGAAGAGATATATAAGGACGGATCCATGATGCTCCAGGACGTCACCCAGGAGGACACAGGGATCTACACCCTAGAAACCTTTGGAACACATGATCATATTGAAATAACACATGTCTACCTCCAAGTGTACA

>Pme_Psg1N1 (Peromyscus melanophrys; plateau mouse) WGS CABHPR010127705.1

CCTCCATTTTAACCTGTTGGCACCTTTCCACCACTGACCATATCACCATTAAATCTGTCCCATCCCAAGTGGCCAATGGAGACAATGTCCTTCTTCTTGTCAACAATCTGCCAGAAGATCTTCTAACCTTTGCCTGGTTTAAAGGAGAGACAGGCATGAACGTTGGAATTGCAATATATGCACCGGACAGAGATTTAATCATGCAAGGGCCTGGATATAGCGGTAGAGAGACAGTGTACCGCAATGGATCCCTGCTGATCCAAAATGTCAATGAGAAGGACACAGGACTCTACACCCTGCAAACCTTAAATGAACATGGAGATGTTCTGTCAATAACAACTATGCGCCTGCATGTGTACC

>Pme_Psg2N1 (Peromyscus melanophrys; plateau mouse) WGS CABHPR010140247.1

CCTCCCTCTTATCATGCTGGCACCTTTCTACCACTGCCCAATTTGCCCTTGAGTCTGTCCCACCTGAAGTCATCGAAGGAGAAAATGTCCTTTTCCTTGTCCACAATCTGCCAGAGAATCTTGCAGCCGTAGTCTGGTCCAAAATGGTGAAAAGTATGAACCATGGAATTGTAACATATGCACTGAACAAAGATTTAAGTGTGCCAGGGCCTCTACACAGTGGTAGAGAGACACTGTACCGCAATGGATCCCTGCTGTTCAGAAATGTCACCAGGAAAGATACAGGACTTTATACCATAGAGCTCTTAGACAGACTTGGAGATATTGTGTCAACAATAACCACTTATCTTCGCGTGCACA

>Pme_Psg3N1 (Peromyscus melanophrys; plateau mouse) WGS CABHPR010033318.1

CCTCCCTTTTAACTTTCTGGCACCTGTCTACCACTGCTGATGTGACCATTGAATCACTGCCGCTCCTAGTGGCTGAAGGAGAAAATGTCCTTTTCCTTGTCCATGATCTGCCGGAAAATCTTACGGCCTTAGCCTGGTTCAAAGGACTAACAAATATGAAACAAGGAATTGCAATATATACACTGCACAGAAATTTAAGTGTGACAGGGCCTGTGCACAGTGGCAGAGAGACAATATATCACAATGGATCTCTGTTGCTGGAAAATGTCACTCAGAAGGACACAGGATACTATACTCTACGAACCTATAACAGACGAGCAAAAATCGTATCAACAACATCCATGTACCTCCAAGTGCACG

>Pme_Psg4N1 (Peromyscus melanophrys; plateau mouse) WGS CABHPR010129806.1

CCTCCCTGTTAACCTTCTGGCACCTGTCCACCACTGCCCATGTGACCATTGAATCACTGCCACCCCAAGTGGCTGAAGGAGAAAACGTCCTTTTCCTTGTCCGTGGTCTGCCAAAGAATCTTATAGCCTTTGCCTGGTTGAAAGGGCTAGCAAATATGACACATGGAATTGCATGGTATACACTGGACAAAAATTCACATGGGCCAGGGCCTGTAAACAGTGGTAGAGAGATAGTGTATCGCAATGGATCCCTGCTGCTTCAAAATATCACCCAGAAGGACACAGGAACCTATACCCTACAAATCTATAATAGACGTGGAAAAATCATATCAACAACATCCATTTACCTCAATGTGCCTG

>Pme_Psg5N1 (Peromyscus melanophrys; plateau mouse) WGS CABHPR010128480.1

CCTCCCTCTTAACCTCCTGGCACCTGTTCACCACTGCCCATGTGACCATTGAGTCACTTCCACTCCAAGTGGCTGAAGGAGAAAACGTCCTTTTCCTTGTCCATGATCTGCCAAAGAATCTTATAACCTTTGCCTGGTTTAAAGGGCTAACAAATATGACACAAAGAATTGCAATGTATACAACGGACAGCAATGTAAGTGGGCCAGGACATGTGCACAGTGATAGAGAGAGAATATATCATAATGGATCCCTGTTGATCGAAAATGTCACCCAGAAGGACACAGGAATCTATACCCTACGAACCTATAATAGACATGGAAAAATTGTATCAACAACATCCATGTACTTTCACGTGTCCG

>Pme_Psg6N1 (Peromyscus melanophrys; plateau mouse) WGS CABHPR010118326.1

CCTCCCTGTTAACCTTCTGGCACCTGTCCACCACTGCCCATGTGACCATTGAATCACTGCCACCCCAAGTGACTGAAGGAGAAAACGTCCTTTTCTTTGTCCGTCGGTTGCCAAAGAATCATATTGCCTTTGCCTGGTTCAAAAGGCTAACAGATATGACACGAGGAATTGCATGGTATACATTGGACAATAATTTACGTGGGCCAGGGCCTGGGCACAGTGGTAGAGAAACAGTGTATCACAATGGATCCCTGCTGCTCCAAAAGGTCACCCAGAAGGACACAGGAAGCTATATCCTACAAACCTATAATAGACGTAGAAAAATCATATCAACAACATCCATTTACCTCCACGTGCATG

>Pme_Psg7N1 (Peromyscus melanophrys; plateau mouse) WGS CABHPR010149198.1

CCTCCCTTTTAACCTTCTGGCTCCTGTCCACCACTGCCACTGTAAACACTGAATCAATGCCACTCCTTGTGGCTGAAGGAGAAAGCATCCTTTTCCCTGCCCAAGATCTTCCAGAGAATATTATAGCCTTAGCCTGGTTCAAAGGACTAACAAAGATGACACAAGGAATTGCGTTGTATGCACTGCACAGCAACTTAAGTTGCCCAGGTTCTGTGCACAGTGGTAGAGAGACAATATATCGCAATGGATCCCTGCTGCTGGAAAAGGTCACCCAGAATGACACAGGATTTTATACCCTACGAACCTTTAATAGATACCGAAAAATCATGTCAACAACATCCATTTACCTCCAAGTGCATG

>Pme_Psg8N1 (Peromyscus melanophrys; plateau mouse) WGS CABHPR010075690.1

CCTCCCTTTTAACCTGCTGTCATCTGTCCACCACTGCCCAAGTCACCATTGAATCAGTGCCACCCCAAGTGGTTGAAGGAGAAAACGTCCTCCTCCGTGTTCATAATCTACCAGAGAATCTTCTAGCCTTTATCTGGCACAAAGGGGCAAGGAATATGAGCCTTAGAATTGCACTGTATTCACTGGCCAAGGATGTAAGTGTGAAAGGTCCCGTACACAGCGGTAGAGAGACAGTGTACAGCAACGGATCCCTGCAGATCCACAATGTCACCCAGAAGGACACAGGATTCTATACCTTACGAACCATTAATAGAGGCATAGGAATTGTATCAATAACAACCAAGTACCTTCACGTGTACA

>Pme_Psg9N1 (Peromyscus melanophrys; plateau mouse) WGS CABHPR010118326.1

CCTCCCTGTTAGCCTTCTGGCACCTCTCCACCACTGCCCATGTGACCATTGAATCACTGCCACCCGAAGTGGCTGAAGGAGAAAACGTCCTTTTCCTTGTCAGTGATCTTCCAAAGAATATTATCGCCTTTGCTTGGTTCAAAGGGCAAACAAATATGACACAAGGAATCGCATGGTATACATTGGACAACAATTCACGTGGACCAGGGCCTGTGCACAGTGGTAGAGAGACAGTGTATCGCAATGGATCCCTGCTGCTCCAAAAGGTCACCCAGAAGGACACAGGACCCTATACCCTACAAACTTTTAATAGACACAGAAAAATCATTTCAACAGCATCCATTTACCTCCATGTGCATG

>Pme_Psg10N1 (Peromyscus melanophrys; plateau mouse) WGS CABHPR010152748.1

CCTTCCTCTTAACCTTCTGGCATCTGTCTGCCATGGCCCATGTGACCATTGAGTCATTTCCACTCCAAGTGACTGAAGGAGAAAACGTCCTTTTCGTTGTCCATGATCTCCCGGAGAATCTTACAACCTTTGCCTGGTTCAAAGGTCTAACAAATATGACACAAAGAATTGCAATATGTACAATGGACAACAATTTAAGTGGGCCAGGACATGTGCACAGTGGTAGAGAGACAACATATTGCAATGGATCCCTGTTGCTCGAAAATGTCAACCAGAAGGACACAGAAAATTATACTCTACAAACCTATAATAGACATGGAAAAATCATAACAACAACATCCATGTACCTCCATGTGTCTG

>Pme_Psg11N1 (Peromyscus melanophrys; plateau mouse) WGS CABHPR010154473.1

CCTGCCTTTTAACCTCCTGGCACCTGTCCACCACTGCCTATGTGACCACTAAAACAGTGCCACCCCAAGTGGCTGAAGGAGAAAATGTCCTTTTCCAGGTGCATGATCTTCCAGAGAATATTATAGCCTTTGCCTGGTTCAAAGAGCTAAGAAATATGAACAAAGCAATTGCAGTATATGGACTGCACATCAATTTAAGTGCACCAGGGCCTGTGCACAGTGGTAGAGAAACAGTATATCACAATGGATCCCTGCTGCTTGAAAAAGTCACCCGGAAGGACATAGGATTTTATACCCTGCGGACCTATGATAGAAATGTAAAAATCGTATCAACAACATCCACGTACCTCCATGTGGACA

>Pme_Psg15N1 (Peromyscus melanophrys; plateau mouse) WGS CABHPR010150774.1

TCTCCCTTTTAACCTGTTGGTACCGGCCCACTACTGCCCAAGTCACCATTGAATCAGTGCCTCCCGATGTGGTCGAAGGAGAAAGCAACCTTTTTCTTGTCCACAATTTGCCAGAGCATCTTTTAACCATATCCTGGTTTAAAGAAGAAGCAAATGTGGACCATAAAATTGCGACCTACACACTGAAATACAATATTGCTGTGCCAGGGGCAGCACACAGTGGTAGAGAGACAGTGTACCCAAATGGATCCCTGTGGATTCAAAATGTCACCCATAAGGACACAGGATTCTATATACTACAAACCAGAAGTAGACAAGTAAAAATTATATCAACAATATATACATACCTTCATGTATACA

>Pme_Psg16N1 (Peromyscus melanophrys; plateau mouse) WGS CABHPR010122117.1

TCTCCCTTTTAACCTGTTGGTACCGGCCCACCACTGCCCAAGTCACCATTGAATCAGTGCCTCCCCATGTGGTCGAAGGAGACAGCAACCTTTTCCTAGTCCACAATCTGCCAGAGAACCTTTTAGCCATATCCTGGTTTAAAGAAGGAGCAAATATGGACCATAGAATTGTGACCTACACACTGAAATACAATATATCTCTGCCAGGAACAGCACACAGCGGTAGAGAGACAGTGTACCCAAATGGATCCCTGTGGATTCAAAATGTCACCCATAAGGACACAGGATTCTATATACTACAAACCATAAGCAGACAAGTAAAAACTGTATCAAGAACATACATACACCTTCATGTGTACA

>Pme_Psg17N1 (Peromyscus melanophrys; plateau mouse) WGS fused CABHPR010047767.1 CABHPR010152382.1

CCTCCCTTTTAACCTGCTGGCACCTGTCCACCACTGCCCAAGTTACCATTGAATCAGTGCCGCCCCAAGTGGTTGAAGGAGAAAATGTCCTTCTACnTGCCAACAATCTGCCAGAGAACCTTCTAGCATTTTCCTGGTACAAGGAGGTGAGAAATAGGAACATCAGAATTGCACTATTTGCACTGAACACTAATCAAAGTGTGATGGGGCCTGAACAAAGTGACAGAGAAACAGTGTACAGCAATGGATCCCTGTGGCTGAAAAATGTGACCAAGAAGGACACAGGATTCTATACCCTACAAACAGTAAATAGAGGTGGAAAAATTGTATCTACAACAACCATGTACTTCCATGTGTACA

>Pme_Psg18N1 (Peromyscus melanophrys; plateau mouse) 18.2.21 WGS CABHPR010152748.

CCTCTATTTTAATCTGCTGGCACCTTTCAACCACTGATGATTTTACCATGGAACCTGTCCCACCCCACGTGGCCAGTGGAGACAATGTACTTCTTCTTGTCCACAATCTGCCAGAGAATCTTATAGTCTTTTCCTGGATCAAAGGGGAGATAAGCATGAATCATACAATTGCAATATATATACCAAACAAGAAGTTAAGTGTGCCAGGGCGTTTATATAGTGGTAGAGAGACAGTGTATGGCAATGGATCCCTGCTCCTCCAAAATGTCAATGAGAAGGACACAGGAATTTATACCCTACAAACCTTTAATAGACACACAGATGCTGTGTCGCAAACATCCATGTACCTCTATGTTCACA

>Pme_Psg19N1 (Peromyscus melanophrys; plateau mouse) WGS CABHPR010124040.1

CCTCCCTTTTAATCTGCTGTCATTTAGCCACCACTGCCAAAGTCACCATTGAATCAGTGCCACTCAATGTGTTCGAAGGAGACAATGTCCTTCTACATGTCCACAATCTGCCAGAGAGTCTTCTAGCCTTTGCTTGGTTCAAAAGGCTGACAAAAACGAAGCACAGAATTGCACTCTATGCACTGAACACCAATTTAATTGTGCCGGGGCCTGTACACAGTGGTAGAGAGACAGTGTACCGCAATGGATCCCTGTGGATTCAGAATGTCACCCATAAGGACACAGGATTCTACACCATACAGACCATAAATAGACATGGAAGAACTGTATCAATAACAGCCATGTACCTCCATGTGTACA

>Pme_Psg21N1 (Peromyscus melanophrys; plateau mouse) WGS CABHPR010144011.1

CCTCCTTTTTAACCTTTTGGCACCTGTCTACAACTGACCATGTGACCACTGAATCACTGCCACCCCAAGTGGCTGAAGGAGAAAACATCCTTTTCCTTGTCCAGAATCTGCCAAAGAATCTTATAGCCTTTGCTTGGTTCAAAGGGCTAAGAAGTATGAAACAAGGAATTGCAATGTATACACTGCACAACAATTTAAGTGGTCCAGGGCCTGTGCACAGCGGTAGAGAGACAATATATCACAATGGATCCCTGCTGCTCGAAAAGGTCACCCAGAAGGACACAGGATACTATACGCTAAGAACATATAACAGACGGGGAAAAATCGTATCAACAACATCCATGTACCTCCACGTGCATG

>Pme_Psg22N1 (Peromyscus melanophrys; plateau mouse) WGS CABHPR010139926.1

CCTCCCTTTTAACCTGCTGTCACCTGTCCATCACTGCCCAAGTCACCATTGAATCAGTGCCACCCCAAGTGGTTGAAGGAGAAAACGTCCTCCTCCGTGTTCATAATCTACCAGAGAATCTTCTAGCCTTTGTCTGGCACAAAGGAGTGAGGAATATGAGCCTTGGAATTGCACTGTATTCACTGGCCAAGAATGTGAGTGTGATAGGTCCTGTACACAGTGGTAGAGAGACAGTGTACAGCAACGGATCCCTGCAGATCCACAATGTCACCCAGAAGGACACAGGATTCTATACCTTTCGAACTATAAATGCACATGTAGGAGTTGTATCAATAACGACCACATACCTTCATGTGCACA

>Pme_Psg23N1 (Peromyscus melanophrys; plateau mouse) WGS CABHPR010149793.1

CCTCTCTTTTAACCTGTTGGCACCTACCCACCACTGTCCAAGTCATTATTGAATTAGTGCCACCTGATGTGGTTGAAGGAGAAAATGTCCTTCTCCTTGTCCGCAATCTGCCAGAGAATCTTGAAGCCTTTGTCTGGTACAAAGGGGTGACAAATATGAACCTCGGAATTGTACTTTATTCACTGACCACTAATTTAAGTGTGGCAGGGCCTGAATACAGTGGTAGAGAGACAGTTTACAGAAATGGATCCCTGCGTCTCCAAGATGTCACCCAGAAGGACACAGGATTCTATACCCTACGATCCATAAGCAGACATAAAGAAATCATATCAACAACATCCATATACCTCCACGTGTACT

>Pme_Psg24N1 (Peromyscus melanophrys; plateau mouse) WGS CABHPR010121093.1 -

CCTCCATTTTAACCTGCTGGCACCTTTCCACCACTGATGGTTTTGAGGTGGAAACTGTCCCACGCCATGTAGCCAATGGAGACAACATCCTTTTCCTTGTCCGCAATCTGCCAAAGAATCTTCTATCTTTCGCCTGGATCAAAGGGGCAACAAGCATGAATGATGCAATCATAGTATATATACCAAACAAAAATTTAAGTGTGCCAGGACGTTTTCACAGTGGTAGAGAGACAGTGTATGGCAATGGATCCCTGCTGATCCAAAATGTCAACCAGAAGGACACAGGAATCTATTCTCTACGAGCTTTTCATAGACGCACAGATACTGTGTCACAAATAGGCACATACCTCTACGTGAACA

>Pnu_Ceacam9N (Peromyscus nudipes; naked-footed deer mouse) WGS CABHPH010103538.1

CCTCCCTCTTGACCTGCTGGAATGCACCTACTACTGCCGAGCTCACTATTGAATTAGTGCCTCCCATGGTGGCTGAAGGTGGAAACTCCGTTCTATTTGTGCACAAAATGCCGTTGAACGTCCAGGCATTTTACTGGTACAAACAGAAAGATCCGACGAAGAGCTATGAAGTTGCCCGGTACTTAACACCCGATAACACAACTTCAAAGATGCCTCAACACAGCGGCAGAAAAACGGTGTTCTACAGTGGATCCCTGCTGATCAGAAACGTCACCCAGGCTGACAGCGGATTCTACACCTTACTAACATTTAACACAGAAATGGAAAGTGAACTAACACACGTGTATCTGGAAGTCCACA

>Pnu_Ceacam11N1 (Peromyscus nudipes; naked-footed deer mouse) WGS CABHPH010147038.1

CCTCCCTTTTAACCTGCTGGCTGCCTCCTACTGTTGCCCAGCTCACCATTGAATCAGTGCCACCCATTTCTGCGGAAGGGGATAATGTTCTTCTGCTTGTGCACAACCTTCCTGAGAATGTTCAAGCCTTTTCCTGGTACACCGGAGTTATGGTGCTCAAGAGCCGTGAAATTGCAAGATGTGCAATAGCTACCAATTCATGTGTGCTGGGGTCTGCACACAGTGGTAGAGAGACAGCATTCAATAATGGATCTCTGCTGATCGAGAATGTCACCAGGAAGGACTCAGGATACTACATCCTTCAAACACTTAATACAAATTCTAGATCTGAAATAACACGTGCAGAATTTTTTGTACACA

>Pnu_Ceacam15N (Peromyscus nudipes; naked-footed deer mouse) 15.10.20 WGS CABHPH010098808.1

CCTCACTTTTAACCTGGTGGAACTCCCTCACCCTGGCGCTAAGAACTATTAAAGAAATGCAGTTTTCTGCTGCTGAAGCGGGGACGGTTCTTCTCTCTGTTTCTATTCAGGCAGAGAACCTTCTCTCTTTTCACTGGTACAAAGGGAAAGATGTAAATAACAATTTTACAATTGCCCATTATGAAAAGGACACAGATTTACTTAAACTTGGGATTAAAACCAGCAGCAAGGAAGAGATATATAAGGACAGATCCATGATGCTCCAGGACGTTACCCAGGAAGATACGGGAATCTACACCCTAGAAACCTTTGGAACACATGATCAACATGAAATAACACATGTCTACCTCCAAGTGTACA

>Pnu_Psg1N1 (Peromyscus nudipes; naked-footed deer mousenaked-footed deer mouse) WGS CABHPH010160498.1

CCTCCATTTTAACCTGCTGGCACATTTCCACCTCTGACCATATCACCATTAAATCTGTCCCATCCCAAGTGGCCAATGGAGACAACGTCCTTCTTCTTGTCAACAATCTGCCAGAAGATCTTCTAACCTTAGCCTGGTTTAAAGGAGAGACAGGCATGAACGTTGGAATTGCAATGTATGCACCAGACAGAGATTTAATCATGCAAGGGCCTGGATATAGCGGTAGAGAGACAGTGTACCGAAATGGATCCCTGCTGATCCAAAATGTCAATGAGAAGGACACAGGACTCTACACCCTGCAAACCTTAAATGAACATGGAGATGTTCTGTCAATAACAACTATGCGCCTGCATGTGTACC

>Pnu_Psg2N1 (Peromyscus nudipes; naked-footed deer mouse) WGS CABHPH010160498.1

CCTCCCTCTTATCATGCTGGCACCTTTCTACCACTGCCCAATTTGCCCTTGAGTCTGTCCCACCTGAAGTCATCGAAGGAGAAAATGTCCTTTTCCTTGTCCACAATCTGCCAGAGAATCTTGCAGCCGTAGTCTGGTCCAAAAGGGTGAAAAGTATGAACCATGGAATTGTAACGTATGCACTGAACAAAGATTCAAGTGTGCCAGGGCCTCTACACAGTGGTAGAGAGACACTGTACCGAAATGGATCCCTGCTGTTCAGAAATGTCACCAGGAAAGATACAGGACTTTATACCATAGAGCTCTTAGACAGATTTGGAGATATTGTGTCAACAATAACCACTTATCTTCGCGTGCACA

>Pnu_Psg3N1 (Peromyscus nudipes; naked-footed deer mouse) WGS CABHPH010045148.1

CCTCCCTTTTAACTTTCTGGCACTTGTCCACCACTGCCGATGTGACCATTGAATCACTGCCGCTCCTAGTGGCCGAAGGAGAAAATGTCCTTTTCCTTGTCCACCATCTGCCGGAAAATCTTACGGCCTTAGCCTGGTTCAAAGGACTAACAAATATGAAACAAGGAATTGCAATATATACACTGCACAGAAATTTAAGTGTGACAGGGCCTGTGCACAGTGGCAGAGAGACGATATATCACAATGGATCTCTGTTGCTGGAAAAGGTCCCCCAGAAGGACACAGGATACTATACTCTACGAACCTATAACAGACGTGCAAAAATCGTATCAACAACATCCATGTACCTCCAAGTGCATG

>Pnu_Psg4N1 (Peromyscus nudipes; naked-footed deer mouse) WGS CABHPH010138579.1

CCTCCCTGTTAACCTTCTGGCACCTGTCCACCACTGCCCATGTGACCATTGAATCACTGCCACCCCAAGTGGCTGAAGGAGAAAACGTCCTTTTCCTTGTCTGTGGTCTGCCAAAGAACCTTATAGCCTTTGCCTGGTTGAAAGGGCAAACAAATATGACACATGGAATTGCATGGTATACACTGGACAACAATTCACATGGGCCAGGGCCTGTAAACAGTGGTAGAGAGATAGTGTATCACAATGGATCCCTGCTGCTTCAAAATATCACCCAGAAGGACACAGGAACCTATACCCTACAAATCTATAATAGACGTGGAAAAATCATATCAACAACATCCATTTACCTCAATGTGCCTG

>Pnu_Psg5N1 (Peromyscus nudipes; naked-footed deer mouse) WGS CABHPH010113223.1

CCTCCCTCTTAACCTCCTGGCACCTGTCCACCACTGCCCATGTGACCATTGAGTCACATCCACTCCAAGTGGCTGAAGGAGAAAACGTCCTTTTCCTTGTCCATGATCTGCCAAAGAATCTTATAACCTTTGCCTGGTTTAAAGGGCTAACAAATATGACACAAAGAATTGCAATGTATACAATGGACAGCAATGTAAGTGGGCCAGGACATGTGCACAGTGATAGAGAGAGAATATATCATAATGGATCCCTGTTGATCGAAAATGTCACCCAGAAGGACGCAGGAATCTATACCCTACGAACCTATAATAGACATGGAAAAATTGTATCAACAACATCCATGTACTTTCACGTGTCTG

>Pnu_Psg6N1 (Peromyscus nudipes; naked-footed deer mouse) WGS CABHPH010145792.1

CCTCCCTGTTAACCTTCTGGCACCTGTCCACCACTGCCCATGTGACCATTGAATCACTGCCACCCCAAGTGACTGAAGGAGAAAACGTCCTTTTCTTTGTCCGTCAGTTGCCAAAGAATCATATTGCCTTTGCCTGGTTCAAAAGGCTATCAGATACGACACGAGGAATTGCATGGTATACATTGGACAATAATTTACGTGGGCCAGGGCCTGGGCACAGTGGTAGAGAGATAGTGTATCACAATGGATCCCTGCTGCTCCAAAAGGTCACTCAGAAGGACACAGGAAGCTATATCCTACAAACCTATAATAGACGTAGAAAAATCATATCAACAACATCCATTTACCTCCATGTGCATG

>Pnu_Psg7N1 (Peromyscus nudipes; naked-footed deer mouse) WGS CABHPH010048289.1

CCTCCCTTTTAACCTTCTGGCTCCTGTCCACCACTGCCACTGTAAACACTGAATCAATGCCACTCCTTGTGGCTGAAGGAGAAAACGTCCTTTTCCCTGCCCAAGATCTTCCAGAGAATATTATAGCCTTAGCCTGGTTCAAAGGACTAACAAAGATCACACAAGGAATTGCGTTGTATGCACTGCACAGCAATTTAAGTTGCCCAGGTTCTGTGCACAGTGGTAGAGAGACAATATATCGCAATGGATCCCTGCTGCTGGAAAAGGTCACCCAGAATGACACAGGATTTTATACCCTACGAACCTTTAATAGATACCGAAAAATCATGTCAACAACATCCATTTACCTCCATGTGCATG

>Pnu_Psg8N1 (Peromyscus nudipes; naked-footed deer mouse) WGS CABHPH010102570.1

CCTCCCTTTTAACCTGCTGTCACCTGTCCATCACTGCCCAAGTCACCATTGAATCAGTGCCACCCCAAGTGGTTGAAGGAGAAAACGTCCTCCTCCGTGTTCATAATCTACCAGAGAATCTTCTAGCCTTTATCTGGCACAAAGGGGCAAGGAATATGAGCCTTAGAATTGCACTGTATTCACTGGCCAAGGATGTAAGTGTGAAAGGTCCTGTACACAGTGGTAGAGAGACAGTGTACAGCAACGGATCCCTGCAGATCCACAATGTCACCCAGAAGGACACAGGATTCTATACCTTACGAACCATTAATAGAGGCATAGGAATTGTATCAATAACAACCAAGTACCTTCATGTGCACA

>Pnu_Psg10N1 (Peromyscus nudipes; naked-footed deer mouse) WGS CABHPH010147594.1

CCTTCCTCTTAACCTTCTGGCATCTGTCTACCATGGCCCATGTGACCATTGAGTCATTTCCACTCCAAGTGACTGAAGGAGAAAACGTCCTTTTCATTGTCCATGATCTCCCAGAGAATCTTACAACCTTTGCCTGGTTCAAAGGTCTAACAAATATGACACAAAGAATTGCAATATGTACAATGGACAACAATTTAAGTGGGCCAGGACATGTGCACAGTGGTAGAGAGACAATATATTGCAATGGATCCCTGTTGCTCAAAAATGTCAACCAGAAGGACACAGAAAATTATACCCTACAAACCTATAATAGACATGGAAAAATCGTATCAACAACATCCATGTACCTCCATGTGTCTG

>Pnu_Psg11N1 (Peromyscus nudipes; naked-footed deer mouse) WGS CABHPH010159348.1

CCTGCCTTTTAACCTCCTGGCACCTGTCCACCACTGCTCATGTGACCACTAAAACAGTGCCACCCCAAGTGGCTGAAGGAGAAAACGTCCTTTTCCTGGTGCATGATCTTCCAGAGAATATTATAGCCTTTGCCTGGTTCAAAGAGCTAAGAAATATGAAAAAAGCAATTGCAGTATATGGACTGCACGTCAATTTAAGTGCACCAGGGCCTGTGCACAGTGGTAGAGAAACAGTATATCATAATGGATCCCTGCTGCTTGAAAAAGTCACCCGGAAGGACATAGGATTTTATACCCTGCGGACCTATGATAGAAATGTAAAAATCGTATCAACAACATCCACGTACCTCCATGTGGACA

>Pnu_Psg12N1 (Peromyscus nudipes; naked-footed deer mouse) WGS CABHPH010178618.1

CCTCCCTTTTAAGCTGCTGGTATCTACCCACCGCTGCCCAAATCACCATTGAACTACTGCCGCCCAATGTGTTCGAAGGAGACAATGTCCTTCTACAGGTCCACAATCTGCCGGAGAATCTTTTAGCCTTTGCTTGGTTCAAAGGGGTGACACATTTGAAACGTGGAATTGCCATCTATTCACTGAAATACAATTTAAGTGTCACGGGGCCTGTACACAGCGGAAGAGAGACAGTGTACAGCAATGGATCCCTGCTGCTCCAGCATGTCACCCACAAGGACACAGGATTCTACACCCTACGAACCATAAATAGACAAGCAGAAATTGTATCAATGACATCCATGTACCTCCACGTGCACA

>Pnu_Psg13N1 (Peromyscus nudipes; naked-footed deer mouse) WGS CABHPH010178617.1

CCTCCTTTTTAACCTGTTGGTACCTGACCACCACTGCCCAAGTCACCATTGAATTAGTGCCACCCCAAGTGGTTGAAGGAGAAAATGTCATTTTCCTTGTCCGTAATCTTCCAGAGAAGCTTTTTGGCTTATCCTGGTTTAAAGAGGAGACAAATATGAAGTATAGAATTGCAAGCTATGAAATGGCCTACAGTCAGGATTTTCTGGGTGCAGCACACACTGGTAGAGAGACAGTGTACCCCAACGGATCCCTGTGGATTCAAAATGTCACCCAGAGTGACTCAGGAGTCTATATCCTACGAAGCAATAATAGAGTAAGAATTGTATCGTCAACATACATATACCTTCACGTGTACA

>Pnu_Psg14N1 (Peromyscus nudipes; naked-footed deer mouse) WGS CABHPH010150976.1

TCTCCCTTTTAACCTGTTGGTACCGGCCCACTACTGCCCAAGTCACCATTGAATCAGTACCTCCCCATGTGGTCGAAGGAGACAGCAACCTTTTCCTAGTCCACAATCTGCCAGAGAACCTTTTAGCCATATCCTGGTTTAAAGAAGGAGCAAATATGGACCATAGAATTGTGACCTACACACTGAAATACAATATATCTCTGCCAGGAACAGCACACAGCGGCAGAGAGACAGTGTACCCAAATGGATCCCTGTGGATTCAAAATGTCACCCATAAGGACACAGGATTCTATATACTACGAACCATAAGTAGACAAGTAAAAACTGTATCAAGAACATACATACACCTTCATGTGTACA

>Pnu_Psg15N1 (Peromyscus nudipes; naked-footed deer mouse) WGS CABHPH010162487.1, CABHPH010038327.1

TCTCCCTTTTAACCTGTTGGTACCGGCCCACTACTGCCCAAGTCACCATTGAATTAGTGCCTCTCCATGTGGTCGAAGGAGAAAGCAACCTTTTTCTTGTCCACAATCTGCCAGAGAATCTTTTAACCATATCCTGGTTTAAAGAAGAAGCAAATGTGGACCATAAAATTGCGACCTACACACTGAAATACAATATAGCTGTGCCGGGGGCAGCACACAGTGGTAGAGAGACAGTGTACCCAAATGGATCCCTGTGGATTCAAAATGTCACCCATAAGGACACAGGAATCTATATACTACAAACCAGAAGTAGACAAGTAAAAATTGTATCAACAAAATATACATACCTTCACGTGTACA

>Pnu_Psg16N1 (Peromyscus nudipes; naked-footed deer mouse) WGS CABHPH010150976.1

TCTCCCTTTTAACCTGTTGGTACCGGCCCACTACTGCCCAAGTCACCATTGAATCAGTACCTCCCCATGTGGTCGAAGGAGACAGCAACCTTTTCCTAGTCCACAATCTGCCAGAGAACCTTTTAGCCATATCCTGGTTTAAAGAAGGAGCAAATATGGACCATAGAATTGTGACCTACACACTGAAATACAATATATCTCTGCCAGGAACAGCACACAGCGGCAGAGAGACAGTGTACCCAAATGGATCCCTGTGGATTCAAAATGTCACCCATAAGGACACAGGATTCTATATACTACGAACCATAAGTAGACAAGTAAAAACTGTATCAAGAACATACATACACCTTCATGTGTACA

>Pnu_Psg17N1_partial (Peromyscus nudipes; naked-footed deer mouse) WGS CABHPH010165506.1

CCTCCCTTTTAACCTGCTGGCACCTGTCCACCACTGCCCAAGTTACCATTGAATCAGTGCCGCCCCAAGTGGTTGAAGGAGAAAATGTCCTTCTACGTGCCAACAATCTGCCAGAGAACCTTCTAGCTTTTTCCTGGTACAAGGAGGTGAGAAATAGGAACCTCAGAATTGCACTATTTGCACTGAACACTAATCAAAGAGTGATGGGGCCTGAAGAAAGTGACAGAGGAACAGTGTACAGCAATGGATCCCTGTGGCTGAAAAATGTGACCAAGAAGGACACAGGATTCTACACCCTACGAACAGTAAATAGAGGTGGAAAAATTGTATCTACAACAACCATGTACTnnnnnnnnnnnn

>Pnu_Psg18N1 (Peromyscus nudipes; naked-footed deer mouse) WGS CABHPH010038353.1

CCTCCATTTTAATCTGCTGGCACCTTTCAACCACTGATGATTTTACCATGGAACCTGTCCCACCCCACGTGGCCAATGGAGACAATGTACTTCTTCTTGTCCACAATCTGCCAGAGAATCTTATAGTCTTTGCCTGGATCAAAGGGGAGATAAGCATGAATCATACAATTGCAATATATATACCAAACAAGAAGTTAAGTGTGCCAGGGCGTTTATATAGTGGTAGAGAGACAGTGTATGGCAATGGATCCCTGCTCCTCCAAAACGTCAATGAGAAGGACACAGGAATTTATACCCTACAAACCTTTAATAGACGCACAGATACTGTGTCGCAAACATCCATGTACCTCTATGTTCACA

>Pnu_Psg19N1 (Peromyscus nudipes; naked-footed deer mouse) WGS CABHPH010162369.1

CCTCCCTTTTAATCTGCTGTCATTTAGCCACCACTGCCAAAGTCACCATTGAATCAGTGCCACTCAATGTGTTCGAAGGAGACAATGTCCTTCTACATGTCCACAATCTGCCAGAGAGTCTTCTAGCCTTTGCTTGGTTCAAAAGGCTGACAAAAACGAAGCACAGAATTGCGCTCTATGCACTGAACACCAATTTGATTGTGCCGGGGCCTGTACACAGTGGTAGAGAGACAGTGTACCGCAATGGATCCCTGTGGATTCAGAATGTCACCCATAAGGACACAGGATTCTACACCATACAGACCATAAATAGACATGGAAGAACTGTATCAATAACAGCCATGTACCTCCATGTGTACA

>Pnu_Psg21N1 (Peromyscus nudipes; naked-footed deer mouse) WGS CABHPH010045451.1

CCTCCTTTTTAACCTTTTGGCACCTGTCTACAACTGACCATGTGACCATTGAATCACTGCCACCCCAAGTGGCTGAAGGAGAAAACATCCTTTTCCTTGTCCAGAATCTGCCAAAGAATCTTATAGCCTTTGCTTGGTTCAAAGGGCTAAGAAGTATGAAACGAGGAATTGCAATGTATACACTGCACAACAATTTAAGTGGTCCAGGGCCTGTGCACAGCGGTAGAGAGACAATATATCACAATGGATCCCTGCTGCTTGAAAAGGTCACCCAGAAGGACACAGGATACTATACGCTAAGAACATATAACAGACGGGGAAAAATCGTATCAACAACATCCATGTACCTCCACGTGCATG

>Pnu_Psg22N1 (Peromyscus nudipes; naked-footed deer mouse) WGS CABHPH010122830.1

CCTCCCTTTTAACCTGCTGTCACCTGTCCATCACTGCCCAAGTCACCATTGAATCAGTGCCACCCCAAGTGGTTGAAGGAGAAAATGTCCTCCTACGTGTTCATAATCTACCAGAGAATCTTCTAGCCTTTGTCTGGCACAAAGGAGTGAGGAATATGAGCCTTGGAATTGCACTGTATTCACTGGCCAAGGATGCGAGTGTGATAGGTCCTGTACACAGCGGTAGAGAGACAGTGTACAGCAACGGATCCCTGCAGATCCACAATGTCACCCAGAAGGACACAGGATTCTATACCTTACGAACCATTAATAGAGGCATAGGAATTGTATCAATAACAACCACATACCTTCATGTGCACA

>Pnu_Psg23N1 (Peromyscus nudipes; naked-footed deer mouse) WGS CABHPH010150081.1

CCTCTCTTTTAACCTGTTGGCACCTACCCACCACTGTCCAAGTCATTATTGAATTAGTGCCACCTGATGTGGTTGAAGGAGAAAATGTCCTTCTCCTTGTCCGCAATCTGCCAGAGAATCTTGAAGCCTTTGTCTGGTACAAAGGGGTGACAAATATGAACCTCGGAATTGTACTTTATTCGCTGACCACTAATTTAAGCGTGGCAGGACCTGAATACAGTGGTAGAGAGACAGTTTACAGAAATGGATCCCTGCGTCTCCAAGATGTCACCCAGAAGGACACAGGATTCTATACCCTACGATCCATAAGCAGACATAAAGAAATCATATCAACAACATCCATATACCTCCATGTGTACT

>Pnu_Psg24N1 (Peromyscus nudipes; naked-footed deer mouse) WGS CABHPH010078053.1

CCTCCATTTTAACCTGCTGGCACCTTTCCACCACTGATGGTTTTGCTGTGGAAACTGTCCCACCCCATGTGGCCAATGGAGACAGCATCCTTTTCCTTGTCCGCAATCTGCCAAAGAATCTTCTATCTTTCGCCTGGATCAAAGGGGCAACAAGCATGAATGATGCAATCATAGTATATATACCAAACAAAAATTTAAGTGTGCCAGGACGTTTTCACAGTGGCAGAGAGACAGTGTATGGCAATGGATCCCTGCTGATCCAAAATGTCAACCAGAAGGACACAGGAATCTATTCTCTACGAGCTTTTCATAGACGCACAGATACTGTGTCACAAATAGGCACATACCTCTACGTGAACA

>Pob_Ceacam9N_P (Psammomys obesus; fat sand rat) WGS CAJQZG010007229.1

CTTCCCTCTTAACCTACTGGCCCACACCCGCCACTGCCGAGCTCACTATTGAACTGGTGCCACCCGTGGTTGCTGAAGGTGGAAACTCCATTCTGTTTGTGCATGAAATGCCGTTGAACGTCCAGGCATTTTACTAGTACAAACAGGAAGATCCCCCCCAAGAGCTACGAAGGGGCACCACGGTACTTAACGCCCACTAATACAGCTTCCCAGATGCCCCAACACAGCGCAGGAAACCGTGCTCTGCAGTGGATCCCTGCTGACCAGAGGCGTCACCCAGGCTGACGGTGAAGTCTACACCTTACTAACATTTAACCCAGAAATGGAAAGCGAATGAACACAAGTGCATTGGGAAGTACACAG

>Pob_Ceacam15N (Psammomys obesus; fat sand rat) WGS NESX02000058.1

CCTCACTTTTAACCTGCTGGAACTCACCTGCCGCTGCAGCCCTACGAACAACTAAAGAAATGCGGTTCTCTGCTGCCGAAGGGGCAAAGGTTCTTCTCCATGTTCCTAACCAGGAAGAGAATCTTCTCTCCTTTTCCTGGTACAAAGGGAAAAATGAACATGAAAATTTTACAATTGCACATTATGAAAAGGCCACAGATGTACTTAAACTTGGAGATAAAACCAGCGGCAGAGAGGACATATATAAGGATGGATCCATGATGCTCCGGTCCGTCACCCAGGAAGACTCAGGATTCTACACTTTAGAAACCTTTGAAGCACACAATCAGCGTGAAATAACATATGTCCACCTCCAAGTGTACA

>Pob_Psg1N1 (Psammomys obesus; fat sand rat) WGS NESX02000058.1

CCTCCCTTTTAGCCTGCTGGCACCTGCTCACCACTGCTGAAGTCACCATTGAATTAGTGCCGCCCCACGCGGTTGAAGGAGAAAATGTTGTGTTCCTTGTTCACAATCTGCCAGAGAAGGCTGTAGGACTCGTCTGGTTCAAAGGAAGAACAAACAGGAGCCTTGGAATTGCAGTGTACTCTCTGACCACTAAAGTGGGTGTGGAGGGGCCTCTCTACAGTGGGAGAGAGAGGGTGTACAGCAACGGATCTCTGCAGATTGACAATGTCACCTGGGGGGACACGGGATACTACACCCTCCAAACCTTCAATAGACAATCCGAAACCGAGTCAATAGTGTCTACGTACCTCCACGTGAACA

>Pob_Psg2N1 (Psammomys obesus; fat sand rat) WGS NESX02000058.1

CCTCCCTTTTAACCTGCTGGCACCTGTCCACCACTGCCGACGTCATCATTGAATTAGTTCCACCTCAAGTTGTGGAAGGAGAAAATGTCCTTCTCCTCGTCCACAATCTTCCAAGGAATCTTATAGCCCTGGCCTGGTACAAAGGGACGACGACGAGTGTGAGCACAGAAATTGTACTGTATGCACTGAACACTGACGTAAGTGTGCCGGGGCCTGTACACAGCAGCAAAGAGAGCATGTACAGGAACGGATCTCTGCGGATTGACAATGTCACCCGGGGGGACACGGGATACTACACCCTCCGAACCTTCAATAGACAAGTAGAAATGGTGTCAGTGACATCTACCTACCTCCACGTCAACA

>Pob_Psg3N1(Psammomys obesus; fat sand rat) WGS NESX02000058.1

CTTGCATTCACCCTTCTGCCACTGACCAGCTCACTATCGAGCCAATGCCACCCAATGTTGCTGAAGGGAAAAATGCTCTCCTACTTGTTCATAATATCCCAGAGAACCTTCGGTCCTTTTCCTGGTACAAAGGGGTAGCCACTCTCAAGAGACATGAGATCGCGCGGAATGTCATAAAAACTAACAAGAGTAGTCTGGGACCTGCATACACCGGCAGACAGACAGTATACACTAATGGATCCCTGCTGCTGCACAATGCCACCCGGGAGGACGCTGGATTCTACACCCTACGAACCCTAAATACACGGCGTGAAAGTCAAGAAACACACGTGTACCTCCATATATACG

>Ppo_Ceacam9N (Peromyscus polionotus subgriseus; oldfield mouse subspecies) WGS RCWS02242687.1

CCTCCCTCTTGACCTGCTGGAATGCACCCACTACAGCCGAGCTCACTATTGAATTAGTGCCTCCCATGGTGGCTGAGGGTGGAAACGCCGTTCTCTTTGTGCACAAAATGCCGTTGAACATCCAGGCGTTTTACTGGTACAAACAGAAAGACCCGACGAAGAGCTATGAAGTCGCCCGGTACTTAACACCTGACAACACGACTTCGAAGATGCCTCAACAGAGCGGCAGGAAAACGGTGTTCTACAGTGGATCCCTGCTGATCAGAAACGTCACCCAGGCTGACAGCGGATTCTACACCTTACTCACATTTAACACGGAAATGGAAAGTGAACTAACACACGTGTATCTGGAAGTCCACA

>Ppo_Ceacam11N1 (Peromyscus polionotus subgriseus; oldfield mouse subspecies) WGS RCWS02242413.1

CCTCCATTTTAACCTGCTGGCTGCCTCCTACTGTTGCCCAGCTCACCATTGAATCAGTGCCACCCATTTCTGCTGAAGGGGATAATGTTCTACTGCTTGTGCACAACCTTCCTGAGAATGGTCAAGCCTTTTCCTGGTACACAGGAGTTATGGTGCTCAAGAGCCGTGAAATTGCAAGATGTGCAATAGCTACCAATTCATGTGTGCTGGGGCCTGCACACAGTGGTAGAGAGACAGCATTCAATAATGGATCTCTGCTGATCAAGAATGTCACCAGGAAGGACTCAGGATACTACATCCTACAGACACTTAATACAAATTCAAGATCTGAAATAACACGTGCGGAATTTTTTGTACACA

>Ppo_Ceacam15N (Peromyscus polionotus subgriseus; oldfield mouse subspecies) WGS RCWS02242702.1

CCTCACTTTTAACCTGCTGGAACTCACCCACTGCGGCGCTAAGATCTACCAAAGAAATGCGGTTTTCTGCAGCTGAAGGGGGGAAGGTTCTTCTCTCTGTTCCTATCCAGGCAGAGAATCTTCTCTCCTTTCACTGGTACAAAGGGAAAGAAGAAGACCAAGATTTTGCAATTGCCCATTATGAAAAGGACACAGATTCACTTAAATTTGGGAATGCAACCAGCGGCAGGGAAGAGGTATATAAGGATGGGTCCATGATGCTCCAGGACGTCACCCAGGAAGATACAGGGATCTACACCCTAGAAGCCTTTGGAACACATGATCATATTGAAATAACACATTTCTACCTCCAGGTGTACA

>Ppo_Psg1N1 (Peromyscus polionotus subgriseus; oldfield mouse subspecies) WGS RCWS02242249.1

CCTCTATTTTCACCTGCTGGCACCTTTCCACCACTGACCATATCACCATTAAATCTGTCCCATCTCAAGTGGCCAATGGAGACAACGTCCTTCTTCTTGTCAACGATTTGCCTGAAGATCTTCTAACCTTTACCTGGTTTAAAGGCGAAACAGGCATGGACCTTGGAATTGCAAGATATGCACCAGACAGAGATTTAATCATGCAAGGGCCTGGATATAGTGGCAGAGAGACAGTGTACCGAAATGGATCCCTGATGATCCAAAATGTCAATGAGAAGGACACAGGACTCTACACCCTGCAAACCTTAAATGAACATGGAGATGTTCTGTCAATAACAACTATGCGCCTGCATGTGTACC

>Ppo_Psg2N1 (Peromyscus polionotus subgriseus; oldfield mouse subspecies) WGS RCWS02242265.1

CCTCCCTTTTATCGTGCTGGCACCTTTCTACCACTGCCCGGTTTGCCCTTGAATCTGTCCCACCTGAAGTCATCGAAGGAGAAAATGCCTTTTTCCTTGTCCACAATCTGCCAGAGAATCTTGCAGCTGTAGTCTGGTCCAAAAGGGTGAAAAGCATGAACCATGGAATTGTAACATATGCACTGAACAAAGATTTAAGTGTGCCGGGGCCTCTACACAGTGGCAGAGAGACAGTGTACCGCAATGGATCCCTGCTGCTCAGAAACGTCACCAGGAAGGACACAGGACTTTATACCATAGAGCTCTTAGACAGGCTTGGAGATATTGTGTCAACAATAACCGCTTATCTTCGCGTGCACA

>Ppo_Psg3N1 (Peromyscus polionotus subgriseus; oldfield mouse subspecies) WGS RCWS02242274.1

CCTCCCTTTTAACTTTCTGGCACCTGTCCACCACTGCCGATGTGACCATTGAATCACTGCCACCCCTAGTGGCCGAAGGAGAAAATGTCCTTTTCCTTGTCCATGATCTGCCAGAAAATCTTATGGCCTTAGCCTGGTTCAAAGGACTAACAGATATGAAACAAGGAATTGCGATATATACACTGCACAACAATTTAAGTGTGACAGGGTCTGTGCACAGTGGCAGAGAGACAATATATCACAATGGATCTCTGTTGCTGGAAAACATCACCCAGAAGGACACAGGATACTACACTCTACGAACCTATAACAGACGTGCAAAAATCGTATCAACAACATCCATGTACCTCCAAGTGCACG

>Ppo_Psg5N1 (Peromyscus polionotus subgriseus; oldfield mouse subspecies) WGS RCWS02242463.1

CCTCCCTCTTAACCTCCTGGCACCTGTCCACCATGGCCCATGTGACTATTGAGTCACTTCCACTCCAAGTGGCTGAAGGAGAGAACGTCCTTTTCCTTGTCCATGATCTGCCAAAGAATCTTACAACCTTTGCCTGGTTTAAAGGGCTAACAAATATGACACAAAGAATTGCAATGTATACAATGGACAATACTGTAAGTGGGCCAGGACATGTGCACAGTGATAGAGAGACAATATATCATAATGGATCCCTGTTAATCGAAAATGTCACCCAGAAGGACACAGGAATCTATACCCTACGAACCTATAATAGACATGGAAAAACTGTATCAACAACATCCACGTACTTTCACGTGTCTG

>Ppo_Psg6N1 (Peromyscus polionotus subgriseus; oldfield mouse subspecies) WGS RCWS02242435.1

CCTCCCTGTTAACCTTCTGGCACCTGTCCACCACTGCCCATGTGACCATTGAATCACTGCCACCCCAAGTGATTGAAGGAGAAAGCGTTCTTTTCTTTGTCCATCGTCTGCCAAAGAATCTTAATGCCTTTGCCTGGTTAAAAAGGCTAACAAATACGACACGAGGAATTGCATGGTATACATTGGACAATAATTTACGTGGGCCAGGGCCTGGGCACAGTGGTAGAGAGATAGTGTATCACAATGGATCCCTGCTGCTCCAAAATGTTACCCAGAAGGACACAGGAAGCTATATCCTACAAACCTATAACAGACGTAGAAAAATCATATCAACAACAACCATTTACCTCCATGTGCACG

>Ppo_Psg7N1 (Peromyscus polionotus subgriseus; oldfield mouse subspecies) WGS RCWS02242457.1

CCTCCCTGTTAACCTTCTGGCTCCTGTCCACCACTGCCACTGTAAACACTGAATCAATGCCACTCCTTGTGGCTGAAGGAGAAAACATTCTTTTCCCTGTCCAAGATCTTCCAGAGAACATTATGGCCGTAGCCTGGTTCAAAGGACGAACAAAGATGACACAAGGAATTGCATTGTATGCACTGCACAGTGATTTAAGTTGCCCAGGTTCTGTGCACAGTGGTAGAGAGACAATATATCGCAATGGATCCCTGCTGCTAGAAAGGGTCACCCAGAATGACACAGGATTTTATACCCTGCGAACCTTTAATAGACACAGAAAAATCATGTCAGCAACATCCATTTACCTCCATGTGCATG

Ppo_Psg9N1 (Peromyscus polionotus subgriseus; oldfield mouse subspecies) WGS RCWS02242432.1

CCTCCCTGTTAACCTTCTGGCACCTGTCCACCACTGCCCATGTGACCATTGAATCACTGTCACCTGAAGTGGTTGAAGGAGAAAACGTCCTTTTCATTGTGCGTGATCTTCCAAAGAATATTATCGCCTTTGCTTGGTTCAAAGGGCAAATAAATATGACACAAGGAATTGCATGGTATACATTGGACAACAATTCACGTGGGCCAGGGCCTGTGCACAGTGGTAGAGAGACAGTGTATCGCAATGGATCCCTGCTGCTCCAAAAGGTCACCCAGAAGGACACAGGACCCTATACCCTACAAACTTTTAATAGACACAGAAAAATCATTTCAACAACATCCATTTACCTCCATGTGCATG

>Ppo_Psg10N1 (Peromyscus polionotus subgriseus; oldfield mouse subspecies) WGS RCWS02242453.1

CCTCCATCTTAACCTCCTGGCACCTGTCTACCACTGCCCATGTGACCATTGAGTCACTTCCACTCCAAGTGATTGAGGGAGAAAACGTCCTTTTCGTTGTCCATGATCTCCCAGAGAATCTTGCAACCTTTGCCTGGTTCAAAGGTCTAACAAATATGACACAAAGAATTACAATGTGTACAACGGACAATTTAAGTGGGCCAGGACATGTGCACAGCGGGAGAGAGACAATATATTGCAATGGATCCCTGTTGCTCAAAAATGTCAACCAGAAGGACACAGAAAATTATACCCTACAAACCTATAATAGACATGGAAAAATCATATCAACAACATCCAAGTACCTCCATGTGTCTG

>Ppo_Psg11N1 (Peromyscus polionotus subgriseus; oldfield mouse subspecies) WGS RCWS02242429.1

CCTGCCTTTTAACCTCCTGGCACCTGTCCACCACTGCCCATGTGAGCACTAAAACAGTGCCACCCCAAGTGGCTGAAGGAGAAAACGTCCTTTTTGAGGTTCATGGTCTTCCAGAGAATATTATAGGCTTTGCCTGGTTCAAAGAGCTAAGAAATATGAAAAAAGCAATTGCAGTATATGGAATGCACATCAATTTAAGTGCACCAGGGCCTGTGCACAGTGGTAGAGAAACATTATATCGCAATGGATCCATGCTGCTTGAAAAAGTCACCAAGAAGGACATAGGATTTTATACCCTGCGGACCTATGACAGAAATGTAAAAATCGTATCAACAACATCCACGTACCTCCATGTGGACA

>Ppo_Psg12N1 (Peromyscus polionotus subgriseus; oldfield mouse subspecies) WGS RCWS02242513.

CCTCCCTTTTAAGCTGCTGGTATCCACCCACCACTGCCCAAATCACCATTGAACTGCCGCCCAGTGTGTTCGAAGGAGACAATGTCCTTCTACAGGTCCACAATCTGCCAGAGAATCTACTAGCCTTTGCTTGGTACAAAGGCGTGACAAATTTGAAACGTGGAATTGCCATCTATTCACTGAAATACAATTTAAGTGTAACAGGGCCTGTACACAGCGGAAGAGAGACAGTATACAGCAATGGATCCCTGCTGCTCCAGAGTGTCACCCACAAGGACACAGGATTCTACACCTTTCGAACCATAAGTAGACAAGCAGAAATTGTATCAGTGACATCCATGTACCTCCACGTGCACA

>Ppo_Psg13N1 (Peromyscus polionotus subgriseus; oldfield mouse subspecies) WGS RCWS02242512.1

CCTCCTTTTTAACCTCTTGGCACCTGACCACCACTGCCCAAGTCACCATTGAATTAGTGCCACCCCAAGTGGTTGAAGGAGAAAATGTCATTTTCCTTGTCCGTAATCTTCCAGAGAAGATTGTGGGCTTATCCTGGTTTAAAGAGGAGACAAATATGAAGTCTAAAATTGCAAGCTATGAAATGGCCTACAGTCGGGATTTTCTGGGGGCAGCACATACTGGTAGAGAGACAGTGTACCCTAATGGATCCCTGTGGATTCAAAATGTTACCCAGAGTGACTCAGGAGTCTATATCCTACGAAGCGGTAATAGAGTAAGAATTACATCATCAACATACATATACCTTCACGTGTACA

>Ppo_Psg14N1 (Peromyscus polionotus subgriseus; oldfield mouse subspecies) WGS RCWS02242509.1

TCTCCCTTTTAACTTGTTGGTACCGGCCCACCACTGCCCAAGTCACCATTGAATCAGTGCCTCCCCATGTGGTTGAAGGAGAAAGCAACCTTTTTCTTGTCCACAATCTGCCAGACAATCTTTTAACCATATCATGGTTTAAAGAAGAAGCAAATATGGACCATAAAATTACAACCTACACACTGAAATACAATATTGCTCTGCCGGGGCCAGCACACAGTGGTAGAGAGACAGTGTACCCAAATGGATCCCTGTGGATTCAAAATGTCACCCACAAGGACACAGGATTCTATATACTAGAAACCAGAAAAGTAAAAATTATATCAACAATATACATATACCTTCACGTGTACA

>Ppo_Psg15N1 (Peromyscus polionotus subgriseus; oldfield mouse subspecies) WGS RCWS02242504.1

CCTCCCTTTTAACCTGTTGGTACCGCCTCACCACTGCCCAAGTCATCATTGCATCAGTGCCTCTCCATGTGGTCGAAGGAGAAAGCAACCTTTTTCTTGTTCACAATCTTCCAGACAATCTTTTAACCATATCCTGGTTTAAAAAAAGAGCAAATATGGACCATAAAATTGCGACCTACACACTGAAATACAATATAGCTGTGCCGGGGGCAGCACACAGTGGTAGAGAGACAGTGTACCCAAATGGATCCCTGTGGATTCAAAATGTCACCCATAAGGACACAGGAATCTACATACTACAAACCAGAAGTAGACAAGTAAAAATTGTATCAACAAAACATACATACCTTCATGTGTACA

>Ppo_Psg16N1 (Peromyscus polionotus subgriseus; oldfield mouse subspecies) WGS RCWS02242496.1

CCTCCTTTTTACTCTGTTGGTACCAGCCCACCACTGCCCAAGTCACCATTGAATCAGTGCCTCCCCATGTGGTCGAAGGAGAAAGCAACCTTTTCCTTGTCCACAATCTGCCAGAGGATCTTTTATCCATATCCTGGTTTAAAGAGGGAGCAAATATGGACCACAGAATTGCGACCTATATACTGGAATACAACATAGCTCTGCCGGGAACAGCACACAGTGGTAGAGAGACAGTGTACCCAAATGGATCCCTGTGGATTCAAAATGTCACCCATAAGGACACAGGATTCTATATACTACAAACCATAAGTGGAGAAGTAAAAACTGTATCAAGAACATACATACACCTTCATGTGTACA

>Ppo_Psg17N1 (Peromyscus polionotus subgriseus; oldfield mouse subspecies) WGS RCWS02242426.1

CCTCCCTTTTCACCTGCTGGCACCTGTCCACCACTGCCCAAGTCACCATTGAATCAGTGCCGCCCCAAGTGGTTGAAGGAGAAAATGTCCTTCTACGTGCCAACAATCTGCCAGAGAATCTTCTAGCTTTCTCCTGGTACAAGGAGATGAGGAATATGAACCTCAGAATTGCACTATTTGCACTGAACACAAATCAAATTGTGATGGGGCCTGAACACAGTGACAGAGAAATGGTGTACAGCAATGGATCCCTGTGGCTGAAAAATGTCACCAAGAAGGACACAGGATTCTATACCCTACAAACAGTGAATAGAGCTGGAAAAATTGTATCTACAACAACCATGTACTTCCATGTGTACA

>Ppo_Psg18N1 (Peromyscus polionotus subgriseus; oldfield mouse subspecies) WGS RCWS02242451.1

CCTCCATTTTCATTTGCTGGCACCTTTCAACCACTGATGATTTTACCATGGAATCTGTCCCACCCCATGTGGCCAATGGAGACAATGTACTTCTTCTTGTCCACAATCTGCCCAAGAATATTATAGCCTTCGCCTGGATCAAAGGGGAGATAAACATGAATCATACAATTGCAATATATATACCAAACAAAAAGTTAAGTGTGCCAGGGCATTTATATAGTGGTAGAGAGACAGTGTATGGCAATGGATCCCTGCTCCTCCAAAATGTCAATGAGAAGGACACAGGAATTTATACCCTACAAACCTTTAATAGACACACAGATACTGTGTCGCAAACATCCATGTACCTCTATGTTCACA

>Ppo_Psg19N1 (Peromyscus polionotus subgriseus; oldfield mouse subspecies) WGS RCWS02242573.1

CCTCCCTTTTAATCTGCTGTCATTTAGCCACCACTGCTAAAGTCACCATTGAATCAGTGCCACTCAATGTGTTCGAAGGAGACAATGTCCTTCTACATGTCCACAATCTGCCAGAGAATCTTCTAGCCTTTGCTTGGTTCAGAAGGCTGACAAAAACGAAACACAGAATTGCACTCTACGCACTGAACACCAATTTATTTGTGCCGGGGCCTGTACATGTTGGTAGAGAGACAGTGTACCGCAATGGATCCCTGTGGATTCAGAATGTCACCCATAAGGACACAGGATTCTACACCCTAGAGACTATAAATAGACATGGACGAACTGTATCAATAACAACCATGTACCTCCATGTGTACA

>Ppo_Psg20N1 (Peromyscus polionotus subgriseus; oldfield mouse subspecies) WGS RCWS02242518.1

CCTCCCTTTTAATTTACTGCCATTCCCCAACCAATGCTGAAGTCACCATTGAATCAGTGCCACTTGATGTGTTCGAAGGAGACAATGTCCTTCTACATGTCCACAATCTGCCAGAGCGTCTTCTATCCTTTGTTTGGTTCAAAGGGCTGACAAATATGAAGCACAGAATTGCTCTCTATGTACTGAACACCAATTTAATTGTGCCGGGGCCTGTACACAGTGGTAGAGAGACAGTGTACCACAATGGATCCCTGTGGATTCACAATGTCACCCATAAGGACACAGGATTCTACACCCTACAGACCATAAATAGACATGGACGAACTGTATCAATAACAACTATGCACCTCCATGTGCACC

>Ppo_Psg21N1 (Peromyscus polionotus subgriseus; oldfield mouse subspecies) WGS RCWS02242283.1

CCTCCTTTTTAACCTTTTGGCACCTGTCTACAACTGACCATGTGACCACTGAATCACTGCCATCCCAAGTGGCTGAAGGAGAAAATGTCCTTTTCCTTGTCCAGAATCTGCCAGAGAATCTTACAGCCTTTGCTTGGTTCAAAGGGCTCAGATGTATGAAACAAAGAATTTCAATGTATACACTGCACAACAATTTAAGTGGTCCAGGGCCTGTGCACAGCGGTAGAGAGACAATATCTCACAATGGATCCCTGCTGTTCGAAAAAGTCACCCAGAAGGACACAGGATACTATACACTAAGAACATATAACAGACGGGGTAAAATTATGTCAACAACATCCATGTACCTCCACGTGCATG

>Ppo_Psg23N1 (Peromyscus polionotus subgriseus; oldfield mouse subspecies) WGS RCWS02242422.

CCTCCCTTTTAACCTGTTGGCACCTACCCACCACTGTCCAAGTCATTATTGAATTAGTGCCACCTGATGTGGTTGAAGGAGAAAATGTCCTTCTCCTTGTCCGCAATCTGCCAGAGAATCTTGAAGCCTTTGTCTGGTACAAAGGGGTGACAAATATGAACCTTGGAATTGTACTTTATTCGCTGGACACTAATTTAAGAGTGGAAGGGCCTGAATACAGTGGTAGGGAGACAGTGTACAGAAATGGATCCCTGCATCTCCAAGATGTCACCCAGAAGGACACTGGATTCTATACCCTACGATCGATAAACAGACATAAAGAAATCATATCAACAACATCCATATACCTCCACGTGTACT

>Ppo_Psg24N1 (Peromyscus polionotus subgriseus; oldfield mouse subspecies) WGS RCWS02242471.1

CCTCCATTTTAACCTGCTGGCACCTTTCCACCACTGATCGTTTTGCTGTGCAAACTGTCCCACTCTATGTGGCCAACGGAGACAGCATCCTTTTCCTTGTCCGCAATCTGCCAAAGAATCTTCTATCCTTCGCCTGGATCAAAGGGAAAACCAGCATGAATGATGCAATCATAGTATATGTACCAAACATAAATTTAACTGTGCCAGGGCGTTTTCACAGTGGTAGAGAGACAGTGTATGGAAATGGATCCCTGCTCATCCAAAATGTCAACCAGAAGGACACAGGATTCTATTCTCTACTAGCTTTTCATAGGCGCACAGATACTGTGTCACAAATAGGCACATACGTCTACGTGAACA

>Psu_Ceacam9N (Phodopus sungorus; Dzhungarian hamster) WGS MCBN011273945.1

CCATCCTCTTAACGTGCTGGAATACACCCACCACCGCCGAGCTCACTATCGAATTGGTGCCCCCCATGGTGTCTGAAGGCGGAAACTCCATTCTATTTGTGCATAAAATGCCACTGAACGTCCAGGCACTTTACTGGTTCAAACGGAAAAATCCAACCAAGAGCTACGAAGTCGCTCGGTTCTTAACGCCCAATAACACAACGTCGAAGATGCCCCAACACAGCGGTAGGAAAACAGTATTCTACAGTGGATCCCTGCTGATCAGAAATGTTACCCAGGCTGACAGTGGATTCTACACCTTACTGACTTTAAACACAGAAATGGAAAGTGAGCTAACACACGTGCAGCTGGAAGTACACA

>Psu_Ceacam11N1 (Phodopus sungorus; Dzhungarian hamster) WGS MCBN011404886.1

CTTCCCTTTTAATTGTTTGGCTACCTTCTACTATTGCCCAGCTCACCATTGAATCAGTGCCGCCAATTTCCGCTGAAGGGGATAATGTTCTATTATTTGTGCATAATCTGCCTGAGAATGTTCAAGCCTTTTCCTGGTACACAGGAATTATGGTACTCAAGAGCCGTGAGATTGCAAGATATGACATAGCTACCAAATCATGTGTTCTGGGGCGTGCACACAGTGGTAGAGAGACAGCATTCAGTAATGGATCTCTGCTGATCAGTAACGTCACCAGGAAAGACTCAGGATACTACATCCTACAAACGCTTGATACAAATTTGAGATCTGAAGTAACACGTGTGGAATTTTTTGTACACA

>Psu_Ceacam15N (Phodopus sungorus; Dzhungarian hamster) WGS MCBN010680318.1

CCTCACTTTTAACCTGCTGGACCTCACCTGCCGTGGCAGAAAGAAGTACTAAAGAAATCCAGGTCTCTGTTGCCAAAGGAGCAAGGGTTCTTCTCTCTGTTCCTAATCTGGTAGAGAGCATCCTCTCCTTCAACTGGTACAAAGGGAGAGAAGAACACAATGATTTTATGATTGCCCACTATGAAAAGGACCAGGCAGTTAAACTCGGGAATAATTCCAGCGGCAGGGAAGAAATATATTTGGATGGATCCATGATGATCAAGAATGTCATCCAGGAAGACACCGGGATCTACACCCTAGAAATCTTTGGAACAGATGATCAATATGAAATAACACATTTTCACCTCCAAGTGTACA

>Psu_Psg1N1 (Phodopus sungorus; Dzhungarian hamster) WGS MCBN011180894.1

CCTCCTTTTTAACCTTCTGGCTCCTGTCTACCACTGCCCATGTGACCACTACATTAGTGCCACCTCAAGTGGCTAAAGGAGAAAACGTCCTCTTTCCTGTACACAATCTTCCTGAAAATCTTACAGTCTTTGCCTGGTTCAAAGGGCTAAGAAACAAGAAACAAAGAATTGCAATGTACTTACTGCACCACAATTTAAGTGGGCCCGGGCCTGTGTACAGCAGTAGAGAGACCATATATTGGAATGGATCCCTGCTGCTGGAAAAGGTCACCCAGAAGGACTCAGGATTCTATACCCTACGAACCTATAATAGATGTGGAAAAGTCATATCAATCACATCTATATACCGTCATGTGCATG

>Psu_Psg2N1 (Phodopus sungorus; Dzhungarian hamster) WGS MCBN011433720.1

ACTCCCTTTTAACCTTCTGGCACCTGCCCACCACTGCCCAAATGGCCACTGAGTTAGTGCTACCCCTAGTGGCTGAAGGAGATGATGTCCTTGTCCTTGTCCACAATCTGCCAGAGAATCTTCTAGCCTTAGCCTGGTTCAAAGGACTAACAAACATGAAACAAGAAATTGCAATATATGTACTTCACAAAAATTTAAGTGCAACAGGGCCTGTGCACAGTGGTAGAGAGACCATATATCGCAATGGATCCCTGCTGTTTGAAAAGGTCACCCAGAAGGACACAGGATTCTATACCCTAAGAACCTATAACAAACTTGGAAAAATCGTATCAACAATATCCATGTACCTCCATGTGCAGG

>Psu_Psg3N1 (Phodopus sungorus; Dzhungarian hamster) WGS MCBN011448782.1

CCTGCCTTTTAACCTCCTGTCACCTGTCCACCACTGCTCATGTGACCACTGAATCAGTACCACGCCTAGTTGCCGAAGGAGATAACGTCATTTTCCTTGTCCATGATCTGCCTGAGAATATTGTAGTCTTAGCTTGGTTCAAAGGGCTAAGAACTATGAAAGAAGGAATTGCAATATACTCACTGAACAATAATTTAAGTCAGCCAGGGCCTGTGCACAGTGGTCGAGAGACACTATATCACAATGGATCCCTGCAGCTCAAAAATGTCAACCATAAGGATGCAGGATTCTATACTTTACGAACCTATGATAAAAGAGGAAAAATTGTATCAACCACATCCACGTATCTCCATGTGCAGG

>Psu_Psg4N1 (Phodopus sungorus; Dzhungarian hamster) WGS MCBN010736417.1

CCTCCCTTTTAAGCTTCTGCCACCTGGCCACCACTGACTATGTAATCATTGAATCAGTGCCACCCCTAGTGGCCAAAGGAGATGATGTCCTTTTCCTTGTCTCCAGTCTGCCAGAGAATATTCAAGCCATAGCCTGGTTCAAAGGGCGAAAAAATGAGAAAGACTCAATCGCAGTATATGACCTGCAAAACAATACTAGTGCGCTGGAGGCTGTGCACAGTGGTAGAAATACAATATATCACAATGGATCCATGCTGATCAAAAAGACCACGGAGGAGGACGAAGGATACTATACCATGCGAACCTATGATGAACATGCAGAAATTGTATCAACAACAATAACATACCTCCACGTGCAGA

>Psu_Psg5N1 (Phodopus sungorus; Dzhungarian hamster) WGS MCBN011460612.1

TCTCCTTTTTAACCTCCTGGCACCTGTTCGCAACTGCCCAAGTGATCACTGAAGCAGTACCACCCTTAGTGGCTGAAGGAGATAACGTCCTTTTCCTAGTCCACAATCTGCCAAAGAAAATTAAATCCTTAACCTGGTTCAAAGGTCTAACAAATACGACAGAAGACATTGCAACATATGCACTGCACAACAGTTTCAGTCAGCCAGGGTCCATGCACAGTGGTAGAGAGACAATATATCACAATGGATCCCTGCTGCTTGAAAAGGCTAACCTGAAGGACACAGGATTCTATACCCTACGAACCTACAATAGACGTGGAAAAATCTTAACAACAGCATTCCTGTACCTCCGCGTGCACA

>Psu_Psg6N1 (Phodopus sungorus; Dzhungarian hamster) WGS MCBN010695550.1

CCTCCCTTTTAAACTTCTGCCACCTGGCCACCACTGCCTATGTGACCGTGACCACTGAGTCAGTGCCACCCCTGGTGGCCAAAGGAGATGACGTCGTTTTCCTTGTCCACAATCTGCCAGAGAAAACTCAAATCTTAGCCTGGTTCAAAGGTCTAACAAACATGAAAGATGCCATTGCATTATACGGACTGTTCAACAATGTTAGTGGGCCGGGGCCTGCGCACAGTGGTAGAGAGACCATATATCGCAACGGATCCCTGCTGATTGAAAATCTCACGGAGAAGGACACAGGATTCTACACCCTGCGGACCTATAATGAATACATAAAAGTTGTAACGACAACATCCACGTACCTCCACGTGCAGG

>Psu_Psg7N1 (Phodopus sungorus; Dzhungarian hamster) WGS MCBN010793913.

CCTTCCTTCTAACCTGCTGCCACCTCTCCTTCACTGACTATATCACCGTTCGAACTGTTCCATCCCAAGTGGCCAGTGGAGAAAACGTCCTTCTGTCTGTCCACAATCTGCCAGAGGATATTCTAGCCTTCGCCTGGTTCAAAGGTGCGAAAAGCATGAAACATGGAATTGCAATATATGGACTGCACAAGAATCTAAGTGCGACAGGGCCTGCACACAGCGGAAGAGAGACAATATATCGCAACGGGTCCCTGCTCCTCGAGTGTGTCACCGAGAAGGACTCAGGATTCTATACCCTACGATCCATAGATGAACATGGAGAGATTGTGTCAACAACAACCATGAAGCTTCACGTGTACC

>Psu_Psg8N1 (Phodopus sungorus; Dzhungarian hamster) WGS MCBN011304450.1

CATCCATTTTAACATGCTGGCACCTCTCCACCACCACTGCTCACGTCACCATTGACTTTGTACCGCCCCATGTGGTCGAAGGTGAAAACGTCCTTCTCCATGTCCAAGATCTGCCAGAGAATCTCGTAGCCTTAGCCTGGTTCAAAGAGGTGAAAACTATACACCATATAATAGCAATATACTCACTGAACAATGATTTAAGTGTTCCAGGGCCTCTGCACAGTGGTAGAGAGACAGTGTACCGCAATGGATCTCTGCTGCTCAGAAATGTCACAAAGAAGGACATAGGAACTTATACCCTACAAACCATGGATAGACTTTCAAATACCTTGTCAATAACATCCATGTACCTTCTTGTGTACA

>Psu_Psg9N1 (Phodopus sungorus; Dzhungarian hamster) WGS MCBN011441595.1

CCTTCCTTTTAATCTGTTGTTATTCGGCCATCACTGCTGAAGTCACCATCGAATCAGTGCCACTCGATGTGTTCGAAGGAGACAATGTCCTTCTCCATGTCCACAATCTTCCAGAAAATCTTCTAGCCTTTGCTTGGTTCAAAGGGGTGACAAATATGAAACGAGAAATTGCACTCTATGAGTTGAACAACAACGTAAGTGTGCCGGGGCCCTTACACAGTGGTAGAGAGACATTGTACCACAATGGATCCCTGTGGATTTCCAACGTCACCCACAAGGACACAGGGCTCTATACCCTACGGACCATAAATAAGCATGCAAGAATTGTATCCATGACAACCATTCATCTCCACGTGTACA

>Psu_Psg10N1 (Phodopus sungorus; Dzhungarian hamster) WGS MCBN011376055.1

CCTCCCTTTTAACCTGCTGGCATCTGTCCACCACTGTCAATATCACCATTGAGTCAGTGCCACCTCAAGTGATTGAGGGAGAAAATGTTCTTCTACGTGCCAACAATCTCCCAGAGAATCTTCTAGCTTTCGCCTGGTTCAAAAGAGTGAATAAAGTGAGGCGTCGAATTGCACTATATTCACTGACTGCTAATCTAAATGTGACAGGGCCTCAATACAGTGGTAGAGAAACTTTGTATAGCAATGGATCCCTGTGGATCAAAAATGTCACCCAGAAAGACAGAGGATTATACACCCTATGGACAATAAAAAGAAGTGGAAAAAATGTATCTACAACAACCATGTACCTCCGAGTGTACT

>Psu_Psg11N1 (Phodopus sungorus; Dzhungarian hamster) WGS MCBN011300351.1

CCTCCCTTATAACCTGCTGGCACCTCTCTACCACAACCCTTGTCACCAGTGAATCGGTCCCTGTCACCATTGAATCTGTCCCGCCCCATGTGGTTGAAGGAGAAACTGTCCTCTTCCTTGCCCACAACCTGCCAGATAATATTCACTCCATATTTTGGGCGAAAGGGGTGACTACTATGAACCTTGGAATTGCAACATATACACTAAGCGAAAATACGAGTGTGCCAGGGACTGTTAACAGTGGTAGAGAGACAGTGTACCCCAATGGATCCCTGCTGATCAGAAACGTCACAAAGAAAGACACAGGATTCTACACCCTACGAACCTTAACCAAGCCTTTTAGTATTCTGTCAACAACATCCATTTACCTTCATGTGCACA

>Psu_Psg14N1 (Phodopus sungorus; Dzhungarian hamster) WGS MCBN011404670.1

CTTCCCTTTTATCTTTCTGGCACCTACCCACCACCGCCAAGTTGATCAGTGATTCAATCCCAACGCTAGTGGCTGAAGGAGATAATGTTCTTCTCCCTGTACATAATCTTCCAAAGAAAATTAAATCTATAACCTGGTACAAACATGTAAGAAATGAGACAAAAGTAATTGCAACGTATGTACTGCACAAGAATGTAAGTCAACCAGGACCTGCAAACAGCGGTAAAGAGATAATATATCGCAATGGAACCCTGTTTCTTGAAAAGGTGAACATAAAGAATGAAGGATTTTATACTCTACGCACCCATAATAGACATGGAAACGTCGTATCCACAACCATCATGTACCTCAATGTCATCC

>Rdi_Ceacam9N (Rhabdomys dilectus; mesic four-striped grass rat) WGS JADRCG010009641.1

CCTCCCTCTTAACCTGCTGGAATGCACCCGCTGCTGCCGAGCTCACTATTGAGTTAGTGCCACCCATGGTTGCGGAAGGCGGAAACTCTGTTCTGTTTGTGCACGAAATGCCGCTGAATGTCCAGGCGTTTTACTGGTACAAACAGAGAGGTCCAACCAAGAGCTATGAAGTCGCGCGGTACTTAACACCCACTAACGAAAGTTCGAAGATGCCTCAGCACAGTGATAGGAAAACCGTATTCTACAGTGGATCCCTGCTGATCAGAAATGTCACCCAGGCCGACAGTGGAGTCTACACCTTACTAACATTTAACACAGAAATGGAAAGCGAATTAACACACGTGCATCTGGAAGTACGCG

>Rdi_Ceacam11N1 (Rhabdomys dilectus; mesic four-striped grass rat) WGS JADRCG010008142.1

TTTCCCTTTTAACCTGCTGGCTGCTTCCCACTACTGCCAAGATCACCATTGAATCAGTGCCTCCCATTGCTGTTGAAGGGGAAGATGTTTTTCTGTTTGTGCATAACTTGCCAAAGAAGGTTAAAGCCCTTTCCTGGTACACAGGAGTTGCAGCGCTCAAGAGTTGTGAAATTGCAAGACATGAGATAGCTACCAATTCTAGTGTGGTGGGACTTGCGCACAGTGGTAGAGAGACAGTATTCAACAATGGATCTCTGATGATCAAAAGTGTCACCAGAAAAGACTCAGGATACTACACCCTACAAATACTTGATTCAACCTCAAGACCTAAAATAATACATGCAGAATTCTTTGTACACA

>Rdi_Ceacam12N1 (Rhabdomys dilectus; mesic four-striped grass rat) WGS JADRCG010007845.1

TTTCCCTTTTTACCTGCTGGCTGCTTCCCACTACTACTCAACTCACCATTGAATCAGTGCCTCTCATTGCTGTTGAAGGGGAAAATGTTCTTTTGTTAGTGCATAATTTGACAAAGAAGATTGAAGCCCTTTCCTGGTACACAGGAGATAAAGTGCTCAAGAGTTGTGAAATTGCAAGACATATGATAGCTATCAATTCTAGTGTGGTGAGACTTGCACACAGTGGTAGAGAGACAGTACTCAACAATGGATCTCTGCTGATTAAGAGTGTCACCAGAAAAGACTCAGGATATCACACCCTAGAAATACTTGATTCAACCTCAAGACTTGAAATAATACATGCAGAATTATATGTACACA

>Rdi_Ceacam13N1 (Rhabdomys dilectus; mesic four-striped grass rat) WGS JADRCG010008142.1

TTTCTCTTTTAACTTGCTGGCTGCTTTCCACTACTGCCCAGCTCACCATTGAATCAGTGCCTCCCATTGCTATTGAAGGGGAAAATGTTCTTCTGTTTGTGCAAAACCTGCCGAAAAATATTGAAGCCCTTTCCTGGTACAGAGGAGCTAAACCACTCAAGGCTTTTGAAATTGCAAGACATGATATAGCTACTAATTCTAGTGTGGTAGGGCTTGCAAACAGTCATAGAGAGACAGTACTCAACAGTGGATCTCTGCTGATCAAGAGTGTAACCAGAAAAGACTCTGGATACTACACCCTACAAATACTTCATACAACCTCAAGACCTGAAATAATGCGTGCAGAATTCTTTGTACAGA

>Rdi_Ceacam15N (Rhabdomys dilectus; mesic four-striped grass rat) WGS JADRCG010009641.1

CCTCACTTTTAACCTGCTGGAACTCGCCCACCGCAGCACTGCTAACATCTAAGGAAATGCGGTTCTCGGCTGCTGAAGGGGCAAAGGTTCTTCTCTATGTTCCTGACCAGGAAGAGAACCTTATCTCTTTTTCCTGGTACAAAGGGAAAGATGTAAATGAAAATTTTACAATTGCACATTATAAAAAATCCAGCGATTCACTTCAACTTGGAAAGAAAGTCAGCGGCAGGGAAGAAATCTATAAGGACGGCTCCATGATGCTCCAGGCTGTCACCCAGGAAGACACGGGATTCTACACTTTAAAAACCTTTAAAACACATGATCAACAGGAAATAACATATGCTCATCTCCAAGTATATA

>Rdi_Psg36N1 (Rhabdomys dilectus; mesic four-striped grass rat) WGS JADRCG010008190.1

CCTCCCTCTTAACCTGCTGGTTTCTGCCCACCACTGCCCAAGTTGCCATCGAATCCTTACCTCCTCAAGTGGTTGAAGGAGAAAATGTTCTTCTACGTGTTGACAATCTGCCAGAGGATCTTCTAGCCTTTGTCTGGTATAGAGGGGTGACAAATATGAGTCTCGGAATTGCACTGTATTCACTGACCTACAGCGTAATTGTGACGGGGCCCATGCACAGCGGTAGAGAGACGTTGTACAGCAATGGGTCCCTGTGGATCCACAATGTCACCCAGAAGGACACAGGATTCTACACTTTTCGAACCATAAGTAAACGTGGAGAAATTGTATCAAATACAACAATGCATCTTCAAGTGTACT

>Rdi_Psg37N1_P (Rhabdomys dilectus; mesic four-striped grass rat) WGS JADRCG010009061.1

CCTCCCTCTTAACCTGCTGGCTTTTGCCCACCACTGCTGGAGTCACCATCGAATCCTTACCATCCAAAGTGGTTGAAGGAGAAAATATTCTTCTACATGTTGACAATATGCCAGAGAATCTTCTAATGTTTGCCTGGTACAGAGGGGTGAAAAATTTGACACATGCAATTGCATTCTATTCACTGCACCATAGCACAAGTGTGAAGGGGCTGACCCACAGTGGTAGAGAGACATTGTACAGCAATGGGTCCTTGTGGATCCCAAATGTCACCCGGAAGGACACAGGATTCTACACTTTTCAAACCATAAGTAGACATGGAGAAATTGTATCAAATACATGAATGTTCCTTCTCGTGTACT

>Rdi_Psg38N1 (Rhabdomys dilectus; mesic four-striped grass rat) WGS JADRCG010009641.1

CCTCCCTTTTAATCTGCTGGCTCCTGCCCGCCACTACCCAGGTCACCATTGACTTGGTGCCATCCAATGTGGTTGAAGGAGAAAATGTCCTTCTTCTTGTTGACAATCTGCCAGAGGATCTTATGGCCTTAGCCTGGTTCAGAGGACTGAAGAAAATTGTCGTATACATACTGAACACTAAAGTAAGTGTGACAGGGTCCATGTACAGTGGTAGAGAGACAATGTCCAGCAACGGGTCCCTGTGGATCCACAATGTCACTCAGAAGGACACAGGATTCTACACCCTACGAACTGTAAATAGACATGGAGAAATTGCATCAACAACATCCACGTATCTCTACGTGTACA

>Rdi_Psg39N1 (Rhabdomys dilectus; mesic four-striped grass rat) WGS JADRCG010009061.1

CCTCCCTCTTAACCTGCTGGTTTCTGCCAACCACTGCCCAAGTCACCATTGAATCTTTACCGCTCAAAGTGGTTGAAGGAGAAAATGTTCTTTTACGCGTGGACAATCTGCCAGAGAATCTTCTAGGCTTTGCCTGGTACAGAGGGGTGAAAAATTTGAAGCTCGGAATTTCACTGTATTCACTGACCTATAGCAAAAATGTGGAAGGGCCTACACACAGCGGTAGAGAGATATTGTACAGCAACGGGTCCCTGTGGATACAAAATGTCACCAAGCAGGACACAGGATATTACACCTTTCGAACCATAAGTAGACGTGGAGAAATTATATCAAATACATCCCTCCAACTTCGTGTGTACT

>Rdi_Psg39bN1 (Rhabdomys dilectus; mesic four-striped grass rat) WGS JADRCG010009061.1

CCTCACTTTTAACCTGCTGGTTTCTGCCCACCATTGCCCGAGTCACCATTGAATCCTTTCTGATCAAGGTGGTTGAAGGAGAAAATGTTCTTCTACGTGTTGACAATATGTCAGAAAATCTTCTAGGCTTTGCCTGGTATAAAGGGTTGTCAAATATGAGGCTTGGAATTGTACTATATTCTCTGGTCTATAGCATAAGTGTAACAGGGCCCAAGCACAGTGGTCGAGAGATATTGTACAGCAACGGGTCCCTATGGATCCCAAATGTCACCCGGAAGGACACAGGATTCTACACTTTTCGAACCATAAGTAGACGTGGAGAAATTGTATCAAATACATCAATGTTTCTTCTTGTGTACT

>Rdi_Psg40N1 (Rhabdomys dilectus; mesic four-striped grass rat) WGS JADRCG010008805.1

CCTCCCTTTTAACCTGCTGGCACCTGTCTACCACTTCTCAAGTCACCATTGAATTAGTGCCACCCCAAGTGGTTGAAGGAGAAGATGTCCTATTCCTTGTCCACAAACTGCCAGAAAATCTTGTGGCCTTAGTCTGGTACAAAGGGTTGGCAGTTGCAAAACATGCAATTGCACGGTATGCAACAGACACTAGACAAAGTGTAATGGGGCTCAAGTACAGTGCTAGAGAGACCTTGTACAGAAATGGGTCCCTGTTGATCCGCAGTGTCACCCAGAAGGACACAGGATTCTACATCCTACGAACATTAAATAGAATTGGAACTGCTATGTCAACATCACTGTTCCTCCATGTGAACC

>Rdi_Psg42N1 (Rhabdomys dilectus; mesic four-striped grass rat) WGS JADRCG010008190.1

CCTCCCTTTTAACCTGTTGGCACCTGTCTACCACTTCCAAAGTCACCATTGAATTAGTGCCACCACATGTGGTTGAAGGGGAAGATGTTCTTTTCCTTGTTCATAATCTGCCAGAGAATCTTACAGCCTTTGCTTGGTTTAAAGGCAGGACAAATATGAAACGAGGAATTGCACTGTATGCATTGGTTTCTGACATACATGTACACAGTGATAGAGAGACACTGTATAGCAATGGATCCCTGATGATCCACAATATCACCCAGAAGGACAGAGATTATTATACCCTACGAACCTTCAATGGACATGCAAAGACTGTATCAACAACAACCACATTCCTCCATGTGAACC

>Rdi_Psg43N1_P (Rhabdomys dilectus; mesic four-striped grass rat) WGS JADRCG010008805.1

CTTTTCTTTTAACCTTCTGGTTCCTTCCCACCACTACACAGGTTACCATTAAATTAGTGCCACCCCAAGTGGCTGAAGAAAAAAACGTCCTTTTTCCTGTTTACAATCTGCCAGAGAATCATATAGGCACAGCCTGGTTTAAAGGAGTGACAAATATGAACCTTGGAATTGCAATGTATGCACTGGCCTTTAACATAAGTTTTTTAGGGCCTGAACACAGTGTAGAGAGACAGTGTACAGAAATGGATCCCTGGTGCTCTACAATGTCACCCAGAAGGACACAGGATTTTATACTCTACCAACCATAAATAGACATAAAAACTTGTATCAACAACATCCATATATCTACATATGTACA

>Rdi_Psg44N1 (Rhabdomys dilectus; mesic four-striped grass rat) WGS JADRCG010012859.1

CCTTTTTTTTAACCTGCTGGTACCTGCCTACCACTGCACAAATAACCATTGAATTAGTGCCACCCCAAGTGATTGAAGGAGAAAATGTTCTCATAAGTATTGACAATCTGCCAGAGAATCTTGTAACCTTAGCCTGGTTCAGAGGAACAAGGATTAAGAGCCCTCAAATTGGACAATATACACTGGCCACTAATGCTATTGTGCTGGGACCTGGTCACACTGGTAGAGAGACTTTGTACAGCAATGGATCCCTGCAGATCTACAACGTCAACCAGGAGGATACAGGGTTCTACAGCCTACGAATCATAAACAGACATGCAGAAATTGTATCAATAACATCCACATATCTCAACGTGTACT

>Rno_Ceacam9N (Rattus norvegicus; Norway rat) WGS JAKEKU010000001.1

CTTCCTTCTTAACCTGCTGGAATGCACCCGCCGCTGCCGAGCTCACTATTGAATTAGTGCCACCCATGGTTGCTGAAGGCGGAAACTCCGTTTTGTTTGTGCATGAAATGCCATTGAATGTCCAGGCGTTTTACTGGTACAAACAGAGAGATCCGACGAAGAGCTATGAAGTCGCGCGGTACTTAACACCCACCAACGAAAGTTCGAAGATGCCTCAGCACAGCGGCCGGAAAACCGTATTCTACAGTGGATCCCTGCTGATCAGAAACGTCACCCAGGCCGACAGTGGAGTCTACACCTTACTAACATTTAACACAGAAATGCAAAGCGAATTAACACATGTGCATCTGGAAGTACGCG

>Rno_Ceacam11N1 (Rattus norvegicus; Norway rat) WGS JAKEKU010000001.1

TCTCCCTTTTAACCTCCTGGCTGCTTCCCACCACTGCCCAAGTCACCATTGAAGCAGTGCCACCCATTGCTGTTGAAGGGGAAACTGTTCTTCTGTTTGTGCATAACCTGCCAGAGAATGTTCAAGTCCTTTCCTGGTACACAGGACTTGCAGCACTCAAGAGTTGTGAAATTGAAAGATATGTGATAGCTACCAAGTCTCATGAGGTGGGACCGGCATACAGTAGTAGAGAGACAATACTACGCAATGGATCTCTGATGATCAAAAGTGTCAACAAAAAAGACTCAGGATACTACACGCTAAAAATACTTAGTACAACCTCAAGTTCTGAAATAATACATGCAGAATTCTTTGTACACA

>Rno_Ceacam12N1 (Rattus norvegicus; Norway rat) WGS JAKEKU010000001.1

CCTACCTTTTAACCTGCTGGCTGCTTCCCACTACTACCCAGGTCACCATCGAATCAGTGCCATCCATTGCTGTTGAAGGGGAAACAGTTCTTCTATTTGTGCATAGCCTGCCACCTAATATTCTTGCCTTTTACTGGTACAGAGGGGTCAGAGCTCTCAGGAGTTTTCAAATTGCAGAATATGTGATAGCTACCAAGTCTTGTGTGGAGGGACCTTCACACCGTGGTAGAGAGACAGTACTCAGCAATGGATCTCTGCTGATCAAGAGTGTCACCAGACAAGACTCCGGACACTATACTCTACAAATAATCACTACAAATGCAAGACCTGAAATAATACGTGCAGAATTCTTTGTACACA

>Rno_Ceacam15 (Rattus norvegicus; Norway rat) WGS JAKEKU010000001.1

CCTCACTTTTAACCTTCTGGGACTCGCCTGCTGCAGCACTGCTGACAACCAAAGAAATGCGGTTCTCAGCCGCCGAGGGGGCGAAGGTTCTTCTCCATGTCCCTGACCAGGAAGAGGACCTCGTCTCCTTTTCCTGGTACAAAGGGAAAGATGTAAATGAAAATCTCACAATTGCACATTACGAAAAGTCCAGCGACTCACTTCAAATTGGAAAGAAAGTCAGCGGCAGGGAAGAAATCTATAAGGATAGCTCCATGATGCTCTGGGCTATCACCCAGAAGGACACGGGATTCTACACTTTACAGACCTTTAAAGCACATGATCAACAGGAAATAACGTACGTCCATCTCCAAGTATACA

>Rno_Psg36N1 (Rattus norvegicus; Norway rat) WGS JAKEKU010000001.1

CCTCCCTCTTAACCTGCTGGCTCCTGCCCACCACTGCCCAAGTCTCCATTGAATCCTTACCACCCCAGGTGGTTGAAGGAGAAAATGTTCTTCTACGTGTTGACAATTTGCCAGAGAATCTCATAGCCTTTGTCTGGTACAAAGGGCTGACAAACATGAGCCTCGGAGTTGCACTGTATTCACTAACCTATAACGTAACTGTGACGGGACCTGTGCACAGTGGTAGAGAGACATTGTACAGCAATGGGTCCCTGTGGATCCAAAATGTCACCCAGAAGGACACAGGATTCTACACCCTACGAACCATAAGTAATCATGGAGAAATTGTATCAAATACATCCCTGCACCTTCATGTGTACT

>Rno_Psg37N1 (Rattus norvegicus; Norway rat) WGS JAKEKU010000001.1

CCTCACTTTTAACCTGCTGGCTCCTGCCCACCACTGCCCAAGTCACCCTCAAGTCCTCACCGCCCCAGGTGGTTGAAGGAGAAAACGTTCTTCTAAGTGCTGACAATCTGCCAGAGAACATTATAGCTTTCGCCTGGTACAAAGGGGAGACCGACATGAACCGTGGAATTGCACTGTATTCACTGAGGTATACTGTAAGTTTGACGGGGCCTGTGCACAGTGGTCGAGAGACATTGTACAGCGACGGGTCCCTGTGGATCAAAAATGTCACCCAGGAGGACACAGGATTTTATACCTTTCGAATCATAAATAATCATGGAAAAATTCAATCAAATACAACCCTGTTCCTTCACGTGAAAT

>Rno_Psg38N1 (Rattus norvegicus; Norway rat) WGS JAKEKU010000001.1

CCTCCCTTCTAACCTGTTGGCTCCTGACTACTGCCCAGGTCAACATTGAATCGGTGCCATTCAATGTGGTTGAAGGGGAAAACGTCCTTCTTCTTGTCCACAATCTGCCAGAGAATCTCATAGCCTTTGCCTGGTATAGAGGGCTGAGGAAAATTGGAGTATACATACTGAACACTGAAGTAAGTGTGACGGGGCCAATGTACAGCGGTAGAGAGACAGTGTACAGCAATGGTTCCCTGTGTATCCGCAATGTCACCCAGAAGGACACAGGATTCTACACTCTACGAACAGTCAACACACGTGGAGAAACTGTATCAACAACATCCTTGTACCTCTATGTGTACA

>Rno_Psg39N1 (Rattus norvegicus; Norway rat) WGS JAKEKU010000001.1

CCTCCCTTTTAACCAGCTGGCTCTTGCCCACCACTGCCCAAGTCACCCTCGAGTCCTCACCGCCCCAAGTGGTTGAAGGAGAAAACGTTCTTCTAAGTGTTGACAATATGCCAGAGAATATTATAGGTTTTGGCTGGTTCAAAGGGGAGACCGACATGAACCGTGGAATTGCACTGTATTCACTGAGGTATACTATAAGTTTGATGGGGCCCGTGCACAGTGGTCGAGAGACATTGTACGGCAACGGGTCGCTGTGGATCAAAAATGTCACCCAGGAGGACACAGGATTCTACACCTTTCGAATCATAAATAAACATGGAAAAATTCAATCAAATACAACCCTGTTCCTTCACGTGAAAT

>Rno_Psg40N1 (Rattus norvegicus; Norway rat) WGS JAKEKU010000001.1

CCTCCTTCTTAACCTGCTGGCACCTGTCCACCACTTCCCAAGTCACCATTGAACTAGTGCCACCTCAAGTGGTTGAAGGAGAAGATGTCCTATTCCTTGTTCACAAACTGCCAGAAAACCTTATTGGCTTCTGCTGGTACAAAGGGTCACCAATTATAAAACATGGCATTGCACTGTATGAAGCAAACACTAAAGTAGGTGTGGCAGGGCCCATGTACAGTGCTAGAGAGACATTGTACAGAAACGGGTCCATGTTGATCCACAATGTCACCCAAAAGGACACAGGATTCTACAGACTACGAATCTTTAATAGACATCGAGATACTATGTTAACATCCACATTCCTCCACGTGAATC

>Rno_Psg41N1 (Rattus norvegicus; Norway rat) WGS JAKEKU010000001.1

CCTCCCTTTTAACCTGTTTGCTCCTGACCACTGCCCAAGTCACCATTGAATCAGTGCCATCCCAAGTAGTTGAAGGAGACAACGTCCTTCTTCTTGTCCACAATCTGCCAGAGAACATTATAGTCTTAGCCTGGTTCAGAAGGCTAAGGAATATGAACCGTGCAATTGGCCTATACGCACTGAACATTAAAGTAAGCATGATGGGGCCAGTGCACAGCGGTAGAGAGACAGTGTACAGCAATGGTTCCCTGTGGATCTGCAATGTCACCCAGAAGGACACAGGATTCTACATCCTACAAACCTTAAATAGACATGGAGAAATGGTATCAAGAACATCCATATATCTCTACGTGTACA

>Rno_Psg42N1 (Rattus norvegicus; Norway rat) WGS JAKEKU010000001.1

CCTCCCTTTTAACCTGCTGGCACCTGTCTACCACTTCCCAAGTCACTATTGAATTAATGCCACCACAAGTAGTTGAAGGGGAAGATGTCCTTTTCCTCGTCCATAATCTGCCAGAGAATGTTACAACCTTTGTCTGGTTCAAAGGGAGGAAAAATATGAAACGTGGAATTGCACTGTATACAGTGGCCTCTGACCTACTTGTACACAGTGATAGAGAGACACTGTACAGCAATGGATCTCTGATGATCCACAATATCACCCAGAAAGACAGAGAATATTACACCCTACGAACCTTCAATAAACGTTCAGAAACTGTATCAACAACATCCACATTCCTCCATGTGAATG

>Rno_Psg43N1 (Rattus norvegicus; Norway rat) WGS AAHX01004148.1

CTTTTCTTTTAACCTCCTGGCTCCTGCCCACCACTCTCCAAGTCACCATAGAATTAGTGCCACCCCAAGTGGCTGAAGGAGAAAATGTCCTTTTTCTTGCTCACAATCTGCCAGAGAATCTTATAGCCATAGCCTGGTTCAAGGGAGTGACAAATATGAATCTTGGAATTGCACTGTATGCACTGGCCTCTAACATAAGTGTGACAGGGCCTGAACACAGTGGTAGAGAGACGGTGTACAGCAATGGATCCCTGCTACTTCAAAATGTCACCCAGAAGGACACAGAATTCTATACTCTACGAACGTTAAATAGACATGGAGAAATTGTATCAACAACATCCATATACCTCCATGTGTACA

>Rno_Psg44N1 (Rattus norvegicus; Norway rat) WGS JAKEKU010000001.1

CCTCCCTTTTAACCTACTGGCACTTGCCTACCACTGCTCAAATAACCATTGAATTAGTGCCACCCCAAGTGGTTGAAGGAGAAAATGTTCTCATACGTATTGACAATCTGAGAGAGAATAATATAACCTTGGCCTGGTATAGAGGAATGAGTATTAAAAGCCCTCAAATTGGACAATATACACTGGCCACTAATGTTACTGAGCTGGGGCCTGGTCATAGTGGTAGAGAGACTTTGTACAGCAACGGATCCCTGCAGATCTACAATGTCACCCAGGAGGATATAGGATTCTACAGCCTACGGATCATAAACAGTCATGCAGAAATTGTATCAATAGCATCTATATACCTCAACGTGTACT

>Rop_Ceacam9N_P (Rhombomys opimus; great gerbil) WGS REGO01000645.1

CCTCCCTCTTAACCTACTGGACCACACCCGCCACTACCGAGTTCACTATTGAACCGGTGCCACCCGTGGTTGCTGAAGGTGGAAACTCCTTTCTGTTTGTGCATAAAATGCCGTTGAACGCCCAGGCATTTTACTAGTACAAACAGGAAGATCCCCCAAAGAGCTACGAAGGGGCACCACGGTACTTAACGCCCACTAATACAGCTTCCCAGATGCCCCAACACAGCGCAGGAAACCGGTTCTGCAGTGGATCCCTGCTGACCAGAGACGTCACCCAGGCTGATGGTGGAGTCTACACCTTACTAACATTTAACCCAGAAATGGAAAGCAAATTAGCACAAGTGCCTTGGGAAGTACACG

>Rop_Ceacam15N (Rhombomys opimus; great gerbil) WGS REGO01000645.1

CCTCACTTTTAACCTGCTGGAACTCACCTGCCGCTGCAGCCCGACTAACTAAAGAAATGCGGTTCTCTGCTGCCGAAGGGGCAAAGGTTCTTCTCCATGTTCCTAACCAGGAAGAGAACCTTCTCTCCTTTTCCTGGTACAAAGGGAAAAATGAACATGAAAATTTTACAATTGCACATTATGAAAAGGCCACAGGTGTACTTAAACTTGGAGATAAAACCAGTGGCAGAGAAGACATATATAAGGATGGATCCATGATGCTCCGGTCCGTCACCCAGGAAGACACAGGATTCTACACTTTCAAAACGTTTGAAGCACACAATCATCGTGAAATAACATATGTCCACCTCCAAGTGTACA

>Rop_Psg1N1 (Rhombomys opimus; great gerbil) WGS REGO01000645.1

CCTCCCTTTTAGCCTGCTGGCACCTGCTCACCACTGCCGAAGTCACCATCGAATTAGTGCCGCCCCACGCGGTGGAAGGAGAAAATGTCGTATTCCTTATTCACAATCTGCCAGAGAAGGCTGTAGGCCTCGCCTGGTTCAAAGGAAGAACAAACAGGAGCCTTGGAATTGCAGTGTACTCTCTGACCGCTAAAGTGGGTGTGGAGGGGCCTCTGTACAGTGGGAGAGAGAGGGTGTACAGCAACGGATCTCTGCGGATTGACAATGTCACCCAGGGGGACACGGGATACTACACCCTCCAAACCTTCAATAGACAATCCGAAACTGAGTCAATAGCGTCTACGTACCTCCACGTGAACA

>Rop_Psg2N1_P (Rhombomys opimus; great gerbil) WGS REGO01000645.1

CCTCCCTTTTAACCTGCTGGCACCTGTCCACCACTGCCGACGTCATCATTGAATTAGTTCCACCTCAAGTGGTGGAAGGAGAAAATGTCCTTCTTCTCGTCCACAACCTTCCAAGGAATCTTATAGCCCTAGCCTGGTACAAAGGGACGACGAGTGCGAGCATGGAAATTGTACTGTATGCACTGAACACCGATGTAAGTGTGCTGAGGCCAGTACACAACAGCAAAGAGAGCATGTACAGGAGCGGATCCCTGCGGATTGACAATGTCACCTGGGGGACACAGGACGCTACACCCTCCGAACCTTCAATAGACAAGTAGAAACTGTATCAGTGACATCCACGTACCTCCACGTAAACA

>Rop_Psg3N1 (Rhombomys opimus; great gerbil) WGS REGO01000645.1

CTTGCATTCACCCTTCTGCCACTGACCAGCTCACTATCGAACCAATGCCACCCAATGTTGCTGAAGGGAAAAACGCTCTCCTACTGGTTCATAACATCCCAGAGAACCTTCGATCCTTTTCCTGGTACAAAGGGGTAGCCACTGTCAAGAGACATGAGATTGCACGGAATGTCATAAAAACTAACAAGAGTGTTCTGGGACCTGCATACACCGGCAGACAGACAGTGTACACTAATGGATCCCTGCTACTGCACAATGCCACCCGGGAGGATGCTGGATTCTACACCCTACGAACCCTAAATACACGGCGTGAAAGTCAAGAAACACACGTGTACCTCCATATATACG

>Rra_Ceacam9N (Rattus rattus; black rat) WGS JAAIVD010000001.1

CTTCCATTCTAACCTGCTGGAATGCACCCGCCGCTGCCGAGCTCACTATTGAATTAGTGCCACCCATGGTTGCCGAAGGCGGAAACTCCGTTCTGTTTGTGCATGAAATGCCATTGAATGTCCAGGCGTTTTACTGGTACAAACAGAGAGATCCGACGAAGAGCTATGAAGTCGCGCGGTACTTAACACCCACCAACGAAAGTTCGAAGATGCCTCAGCACAGCGGCCGGAAAACCGTATTCTACAGTGGATCCCTGCTGATCAGAAATGTCACCCAGGCCGACAGTGGAGTCTACACCTTACTAACATTTAACACAGAAATGCAAAGCGAATTAACACATGTGCATCTGGAAGTACGCG

>Rra_Ceacam11N1 (Rattus rattus; black rat) WGS JAAIVD010000001.1

TCTCCCTTTTAACCTCCTGGCTGCTTCCCACCACTGCCCAAGTCACCATTGAAGCAGTGCCACCCATTGCTGTTGAAGGGGAAACTGTTCTTCTGTTTGTGCATAACCTGCCAGAGAATGTTCAAGTCCTTTCCTGGTACACAGGACTTGCAGCACTCAAGAGTTGTGAAATTGAAAGATATGTGATAGCTACCAAGTCTCATGAGGTGGGACCGGCATACAGTAGTAGAGAGACAATACTACGCAATGGATCTCTGATGATCAAAAGTGTCAACAAAAAAGACTCAGGATACTACACGCTAAAAATACTTAGTACAACCTCAAGTTCTGAAATAATACATGCAGAATTCTTTGTACACA
[truncated: 21,599 more chars]
